# Recurrent evacuation of mantle mush in ocean islands revealed by clinopyroxene from La Palma

**DOI:** 10.1038/s41467-026-77213-9

**Published:** 2026-09-24

**Authors:** Alberto Caracciolo, Teresa Ubide, Mónica Ágreda-López, Raquel Herrera, Alvaro Marquez, Diego González-García, María José Huertas, Eumenio Ancochea, Nicolás Chicharro, Juan Jesús Coello-Bravo, Maurizio Petrelli

**Affiliations:** 1 University of Perugia, Department of Physics and Geology, Perugia, Italy; 2 University of Iceland, Institute of Earth Sciences, Reykjavík, Iceland; 3 The University of Queensland, School of the Environment, Brisbane, Australia; 4 Universidad Rey Juan Carlos, ESCET, Área de Geología, Tecvolrisk Research Group; Móstoles, Madrid, Spain; 5 Universidad Complutense, Área de Petrología y Geoquímica, Madrid, Spain; 6 Instituto Geográfico Nacional. Centro Geofísico de Canarias, Santa Cruz de Tenerife, Tenerife, Spain; 7 Fundación Telesforo Bravo - Juan Coello; Puerto de la Cruz, Tenerife, Spain

**Keywords:** Volcanology, Geochemistry, Petrology

## Abstract

Temporal variations in magma plumbing influence eruption priming and the interpretation of unrest signals, yet remain poorly constrained in low-flux ocean island volcanoes. Here, we examine temporal changes in the active volcanic system beneath La Palma, Canary Islands, using clinopyroxene zoning from the 1712, 1971, and 2021 eruptions. Combining quantitative trace element mapping, thermobarometry, and cluster analysis, we show that preceding all eruptions, an evolved phonolitic crystal mush resided in the upper mantle (∼18–25 km depth) under relatively cool (1000–1025 °C) conditions. At least one week before eruption, basanitic recharge (1100–1150 °C) recycled this mush and promoted magma ascent, potentially with brief stalling in the crust (∼5–10 km). Mafic recharge was critical in unlocking the mush, yet may not represent the immediate eruption trigger. Globally, upper mantle evolved mushes may be common in ocean island basalt volcanoes, and their occurrence is likely controlled by the island evolutionary stage and magma flux.

## Abstract (web summary)

Historical eruptions at La Palma repeatedly involved hot magma mobilizing a deep, cooler crystal-rich reservoir, highlighting a process potentially common to ocean island basalt systems.

## Introduction

The dynamics of magma plumbing systems regulate not only the timing, composition, and style of volcanic eruptions, but also key subsurface processes such as magma recharge, mixing, and crystal mobilization (refs. ^1, 2^). Temporal changes in the configuration and behaviour of magma feeding and storage systems can influence eruption priming and the run-up to volcanic activity, with implications for interpreting monitoring signals and improving eruption forecasts. Temporal changes may be influenced by variations in magma flux, which represents the rate at which magma enters the lithosphere at a specific volcano. Magma flux modulates the depth and physical characteristics of magma storage, with higher magma flux promoting shallow crustal storage^3–5^. Despite variations in volcanic plumbing systems over time being recently explored in high-flux basaltic systems characterized by frequent volcanic activity (inter-eruption intervals <5–10 years, such as in Iceland and Hawaii^6–9^), temporal variations remain largely underexplored in low-flux basaltic systems where inter-eruption times are longer^3^, translating into monitoring challenges and increased risk potential^3^.

Magmatic processes occurring within a magma plumbing system cannot be directly observed, yet they can be inferred from the chemical zoning and textural features of erupted minerals^2^. This is because growing magmatic crystals can record the thermodynamic and chemical conditions of the magma from which they crystallize, preserving detailed archives of magma evolution and dynamics in their zoning patterns (refs. ^2, 10^). In particular, clinopyroxene crystals are ideal recorders of magmatic processes, as they crystallize over a wide range of depths^11^. and preserve trace element zoning due to slow diffusion rates^10^.

The Cumbre Vieja volcanic system at La Palma, the most volcanically active island of the Canary Islands in historical time, is a great candidate for studying the temporal evolution of magma plumbing characteristics at low-flux ocean island basalt (OIB) systems via clinopyroxene records, as (1) the eruptive products contain abundant clinopyroxene crystals, (2) eruptions are historically documented ^12,13^, with recent paleomagnetic work indicating nine eruptions in the last 1100 years^14^, (3) recent eruptions have shown petrological variations reflecting remobilization of mushes of basanite to phonolitic compositions^15–21^ and (4) recent magmatic reactivation led to the 2021 Tajogaite eruption with major economic and societal consequences^22^. A study focusing on the temporal evolution of the magma plumbing system feeding historical eruptions at Cumbre Vieja is urgent and lacking to date. Specifically, magma storage is considered to take place dominantly at upper mantle depths^19,21,23^. However, potential temporal changes in storage configuration, transient crustal storage^24,25^, and the links between erupted lithologies and storage depths, all of which are essential for monitoring efforts, remain unclear.

**Fig. 1:**
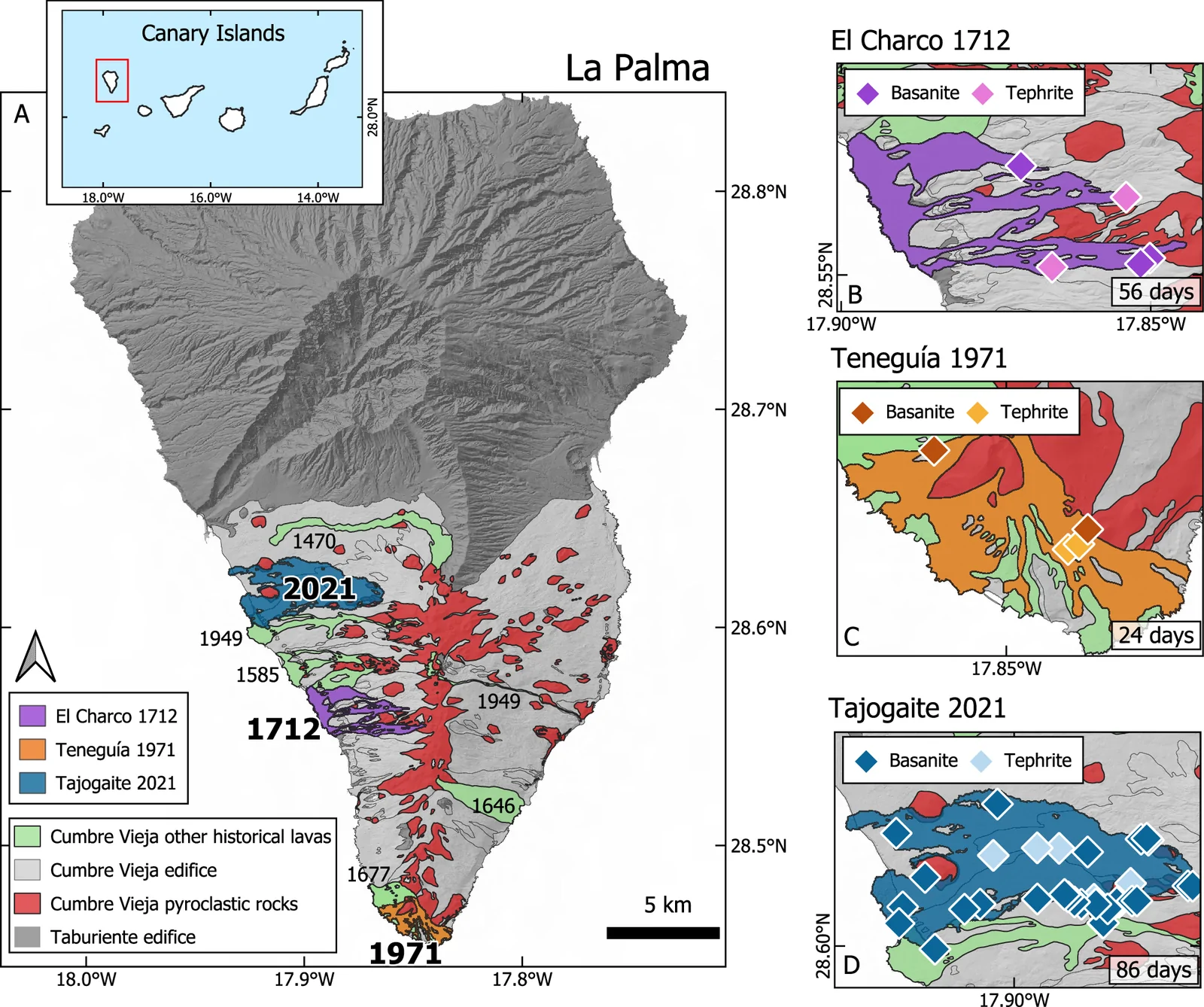
Geological map of La Palma and sample localities of studied eruptions. **A** Simplified geological map of La Palma island, with inset showing the location of the island in the Canaries. The Cumbre Vieja edifice shows historical eruptions that have occurred since the 15th century. **B**–**D** Zoom of historical eruptions studied in this work and sample localities of (**B**) El Charco 1712, (**C)** Teneguía 1971, and (**D**) Tajogaite 2021 eruptions. Sample location is indicated by colored diamonds, distinguishing between amphibole-bearing tephrite and olivine-bearing basanite samples. The duration of each eruption is shown in the bottom right corner. Geological data are from the Cartográfica de Canarias (https://www.grafcan.es/).

In this work, we interrogate the zoning records preserved in clinopyroxene crystals from three historical eruptions at Cumbre Vieja, La Palma: El Charco 1712, Teneguía 1971 and Tajogaite 2021 (Fig. 1). All three erupted early tephrite followed by later basanite lavas^21,26,27^ and some lavas hold evidence of phonolitic mush at depth, despite not being erupted^18,28^. We integrate major and trace element data across clinopyroxene textures with quantitative trace element mapping, pressure–temperature constraints, and cluster analysis of pyroxene populations. We combine the mineral dataset with major and trace element geochemistry of the associated carrier melts (microcrystalline rock matrix). By merging our results with previous work on the 2021 Tajogaite eruption and earlier historical eruptions, we find a striking consistency in the magma chemistry and plumbing system anatomy during historical time, highlighting the key role of mafic recharge in evacuating mantle-seated phonolitic mushes, a process likely occurring at low-flux OIB volcanoes worldwide.

## Results

### Petrography and clinopyroxene textures

**Fig. 2:**
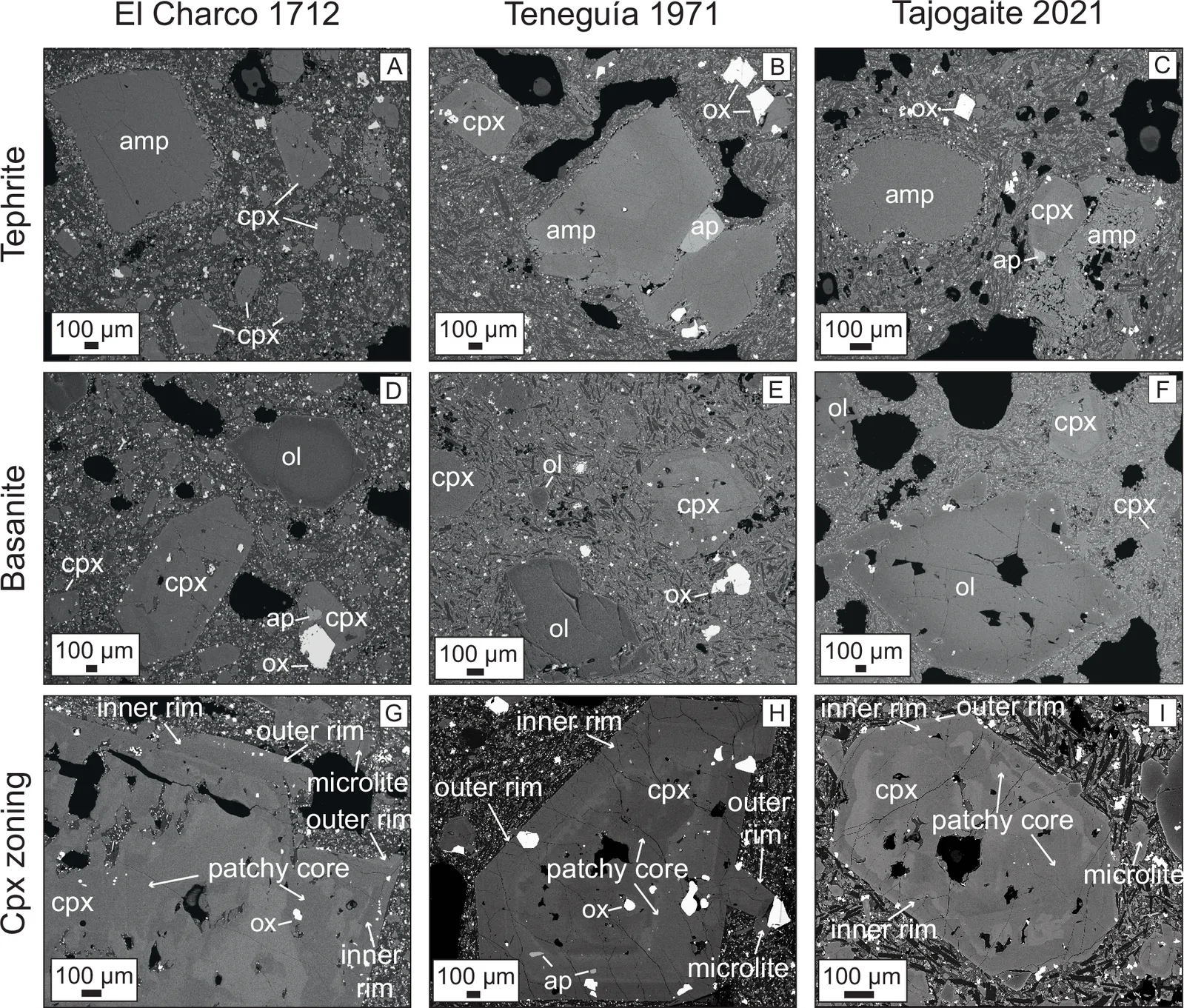
Petrographic features of tephrite and basanite samples from the three La Palma eruptions studied in this work in backscattered electron images (BSE). **A**–**C** Representative BSE images of tephrite samples, showing a mineral assemblage characterized by crystals of clinopyroxene and amphibole. **D**–**F** Representative BSE images of basanite samples, showing a mineral assemblage dominated by crystals of clinopyroxene and olivine. **G**–**I** BSE images of clinopyroxene crystals, highlighting mineral zoning and different textural positions studied in this work. cpx: clinopyroxene; ol: olivine; amp: amphibole; ox: oxide; ap: apatite.

Lava samples in this study classify as tephrites and basanites based on petrographic observations, with tephrites erupted earlier than basanites within each eruption (see geological setting in the Supplementary Information). Both rock types are porphyritic and contain large crystals of clinopyroxene with inclusions of Fe–Ti oxides. Tephrites are distinguished by the presence of amphibole (kaersutite) and absence of olivine, while basanites contain olivine and lack amphibole (Fig. 2).

Clinopyroxene forms large crystals in all three eruptions (up to 5 mm in size), together with microlites smaller than 100 µm. Clinopyroxene crystals can also be found as mineral inclusions in rare plagioclase crystals^18^. Across all eruptions, large clinopyroxene crystals can host inclusions of apatite, Fe–Ti oxides, and glassy to microcrystalline melt inclusions (Fig. 2). The groundmass is microcrystalline and predominantly composed of plagioclase, clinopyroxene, and Fe–Ti oxides, as well as olivine in the basanites and amphibole in the tephrites (Fig. 2).

Clinopyroxene crystals can be broadly divided into three main textural zones across the three eruptions: core, inner rim, and outer rim. The cores represent the innermost parts of the crystals and are texturally complex, characterized by patchy or more rarely sector zoning in back-scattered electron (BSE) images (Fig. 2G-I). Under the petrographic microscope, clinopyroxene cores are green or light brown in plane polarized light, surrounded by brown rims that are typically sector zoned. In BSE, the inner rims appear as darker zones than the cores, suggesting an increase in Mg (reverse zoning; Fig. 2G–I). The outermost zone, in contact with the groundmass, is the outer rim, usually forming a relatively thin (5–30 µm) layer that returns to lighter BSE contrast (Fig. 2G–I).

### Clinopyroxene major elements

**Fig. 3:**
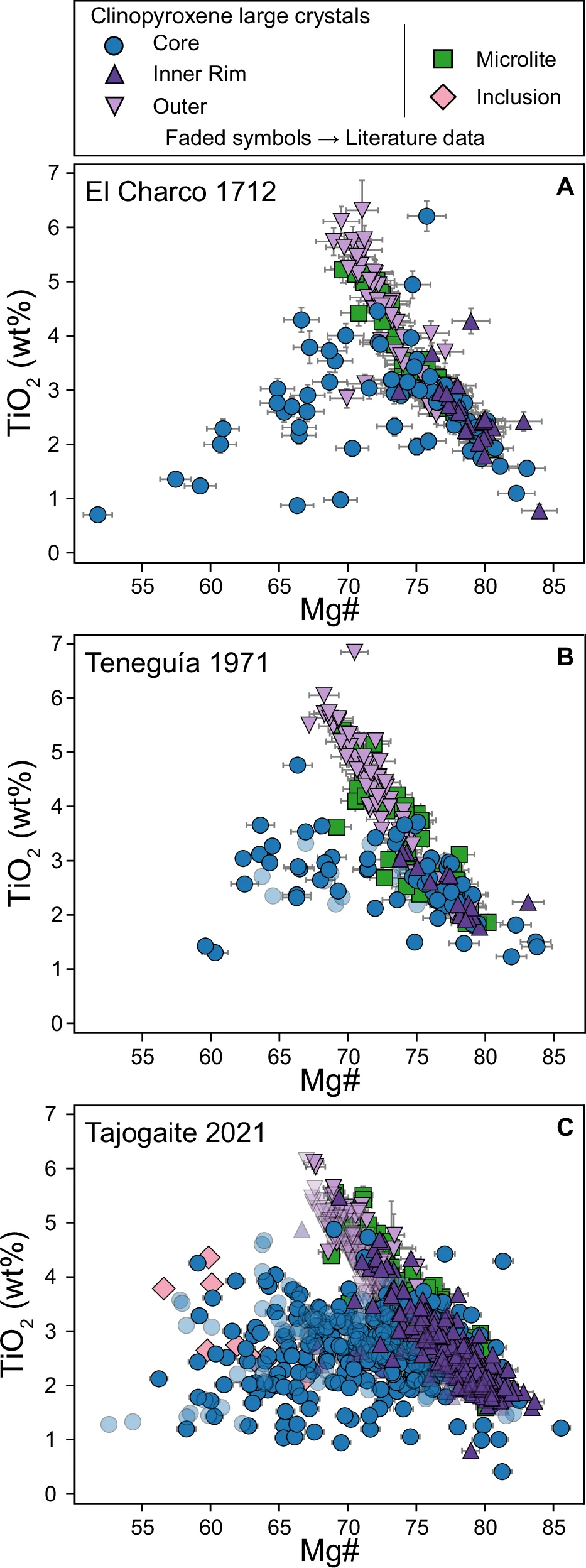
Major element compositions of clinopyroxene crystals. Variation in Mg# as a function of TiO₂ for samples from (**A**) El Charco 1712, (**B**) Teneguía 1971, and (**C**) Tajogaite 2021. Data are categorized by textural position: core, inner rim and outer rim of large crystals and microlite and clinopyroxene inclusions. Literature data for Teneguía 1971^15,44^ and Tajogaite 2021^23^ are plotted as faded filled symbols according to textural positions. Inner rim and microlite compositions from the 2021 Tajogaite eruption^21^ are here plotted using the same color scheme as our dataset, as they were collected from the same crystals and complement our study. Error bars indicate 1σ uncertainties derived from EMPA counting statistics.

Clinopyroxene crystals from all eruptions are diopside in composition and do not display geochemical differences in terms of En [Mg / (Ca + Mg + Fe) × 100]_mol_, Fs [Fe / (Ca + Mg + Fe) × 100] _mol_, Wo [Ca / (Ca + Mg + Fe) × 100] _mol_ components (Supplementary Fig. 1) and Mg# values ([Mg / (Mg + Fe)] × 100, molar basis), which range from 52 to 86 (El Charco 1712, Mg#52-84, *n* = 213; Teneguía 1971, Mg#56-86, *n* = 164; Tajogaite 2021, Mg#56-86, *n* = 786). However, there are consistent Mg# variations from cores to inner and outer rims in large crystals. Cores show the broadest range of Mg# values within each eruption (El Charco 1712, Mg#52-83; Teneguía 1971, Mg#60-84; Tajogaite 2021, Mg#56-86), with compositions Mg#<67 showing green color in plane polarized light. Inner rims consistently have higher Mg# values defining reverse zoning (El Charco 1712, Mg#74-84; Teneguía 1971, Mg#74-83; Tajogaite 2021, Mg#69-84), whereas outer rims have lower Mg# 68–78 across all eruptions. Groundmass microlites span a compositional range that overlaps both inner and outer rims, encompassing the full range defined by these two textural zones (El Charco 1712, Mg#70-80; Teneguía 1971, Mg#69-80; Tajogaite 2021, Mg#69-82) (Fig. 3 and Supplementary Fig. 2).

Bivariate plots of Mg# versus major and minor elements show that clinopyroxene inner rims, outer rims, and microlites generally align along a coherent negative trend in diagrams such as Mg# vs TiO₂, Al₂O₃, and Na₂O, or a subtle positive trend with CaO or Cr_2_O_3_ (Fig. 3 and Supplementary Fig. 3). Inner rims define the primitive end of the trend, showing higher Mg#-Cr_2_O_3_ and lower TiO₂-Al₂O₃ contents compared to outer rims (Fig. 3 and Supplementary Fig. 3) and microlites. In contrast, clinopyroxene cores are scattered over a broad compositional range, including along the main trend but mostly outside of it (Fig. 3 and Supplementary Fig. 3). Core compositions span from primitive, Mg#-rich and TiO₂-poor types, to more evolved, visually green, Mg#-poor and Na_2_O-rich compositions, which in Tajogaite 2021 are occasionally found with samples containing rare large crystals of plagioclase^18,21^.

Overall, clinopyroxene compositions do not systematically vary with host rock type. Crystals from both tephrites and basanites span the full compositional range, though the most evolved cores, with the lowest Mg#, occur mainly in basanites (Supplementary Fig. 4).

### Clinopyroxene trace elements

**Fig. 4:**
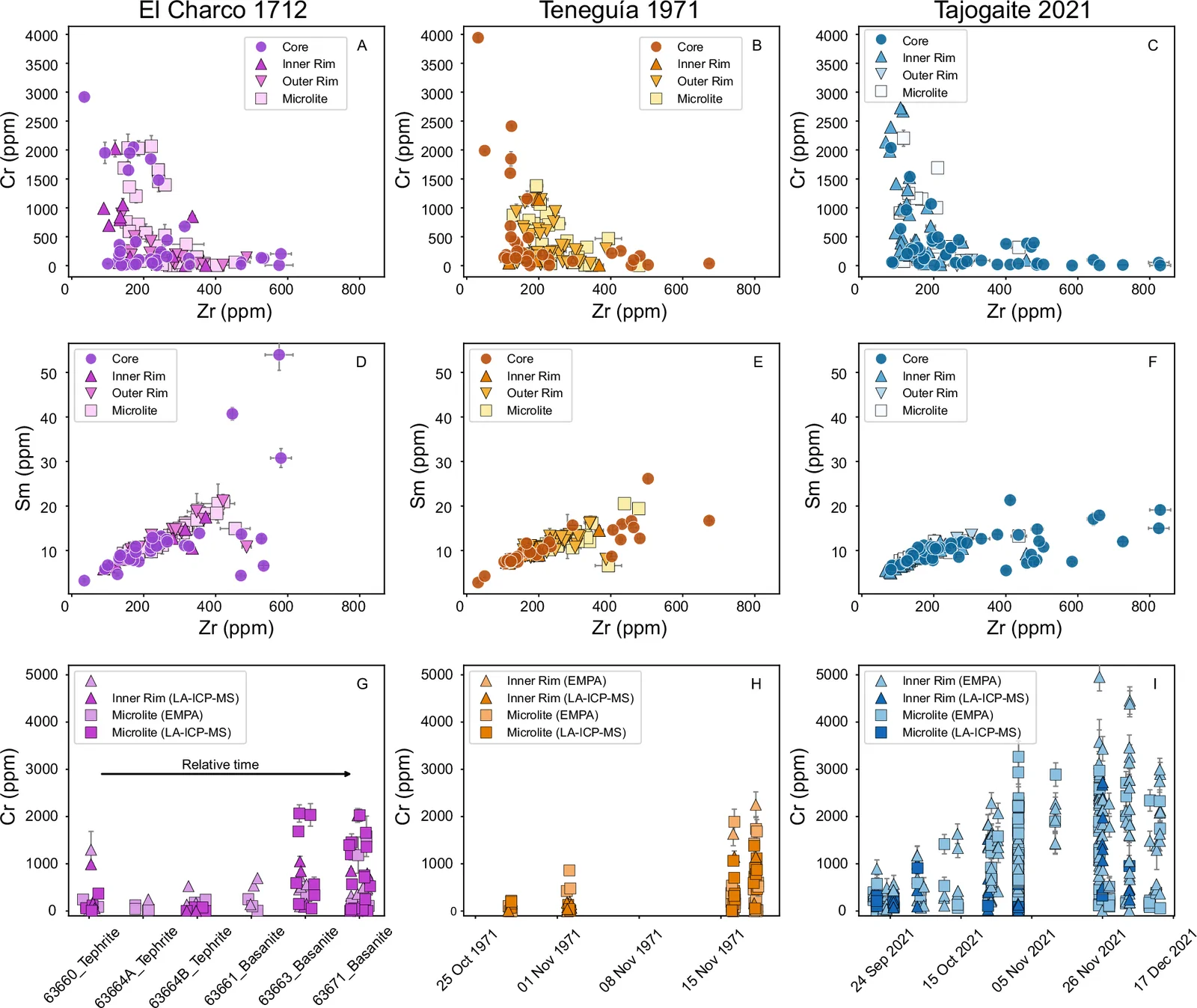
Trace element composition of clinopyroxene crystals. Trace element characteristics of different clinopyroxene textural positions, showing the LA-ICP-MS concentration of (**A**–**C**) Cr and (**D**–**F**) Sm in the different eruptions. Symbols and colors represent different textural positions. **G**–**I** Temporal variation of Cr concentration in inner rim and microlite compositions measured using both EMPA and LA-ICP-MS. For El Charco 1712, samples are ordered from early-erupted tephrite to late-erupted basanite to provide temporal context. For Teneguía 1971 and Tajogaite 2021, samples are plotted according to eruption age. LA-ICP-MS error bars indicate the internal 2SE analytical uncertainty, whereas EMPA error bars reflect 1σ analytical uncertainty based on counting statistics.

Compatible elements in clinopyroxene such as Cr, Sc, and Ni exhibit high concentrations and broad variability at low Zr contents (<200 ppm), reflecting primitive compositions. In contrast, high-Zr (>400 ppm) evolved compositions are characterized by consistently low concentrations of compatible metals (Fig. 4A–C and Supplementary Fig. 5). Within each eruption, Cr concentration in clinopyroxene inner rims and microlites increases with time, from early-erupted tephrites to late-erupted basanites (Fig. 4G–I).

Incompatible trace elements in clinopyroxene correlate positively with Zr, with data from all eruptions aligning along a common trend (Fig. 4D–F and Supplementary Figs. 6 and 7). At Zr ~400 ppm, some clinopyroxene compositions diverge from this main trend, especially for middle and heavy rare earth elements (MREE-HREE) like Sm, Nd, Eu, Gd, Dy and Y (Fig. 4D–F and Supplementary Figs. 6 and 7). High-Zr compositions (>400 ppm) are mostly observed in evolved clinopyroxene cores, which are slightly enriched in HREE (Tm, Yb, Lu) compared to other core compositions (Supplementary Fig. 8). Normalized clinopyroxene rare earth element (REE) patterns are broadly similar across eruptions, without notable differences between tephrite and basanite samples (Supplementary Fig. 9). Clinopyroxene cores display both enriched and depleted compositions, while inner rims are generally more depleted than outer rims and microlites. Overall, REE patterns are parallel, with consistent trace element abundances across the eruptions (Supplementary Figs. 9 and 10).

**Fig. 5:**
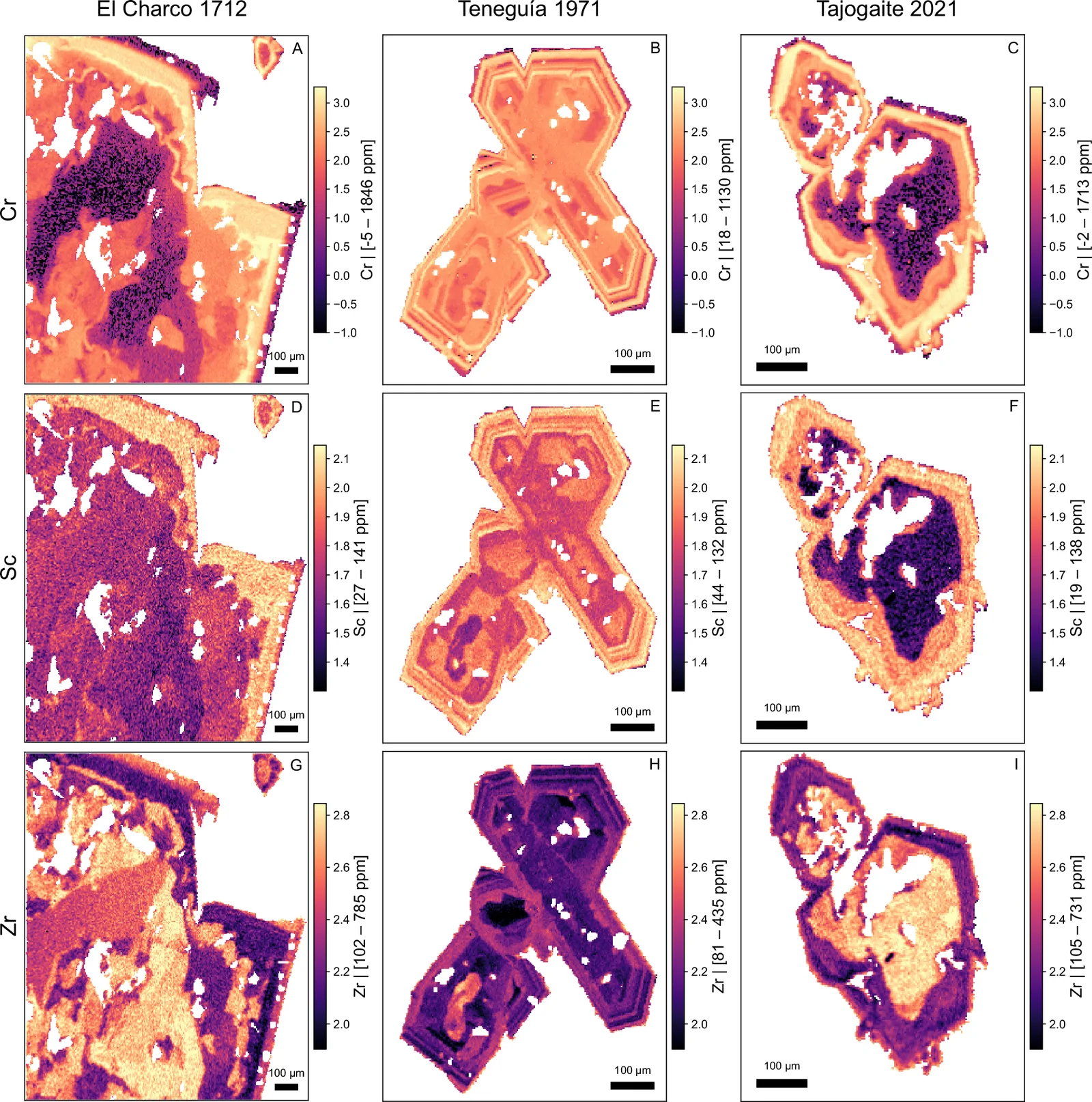
Trace element maps of clinopyroxene crystals. Representative basanite-hosted clinopyroxene crystal maps from El Charco 1712, Teneguía 1971, and Tajogaite 2021, showing the spatial distribution of Cr (**A**–**C**), Sc (**D**–**F**), and Zr (**G**–**I**) concentrations. Maps are visualized using a logarithmic scale and scaled to the maximum common range of Cr (0.1–2000 ppm; log₁₀ = −1–3.3), Sc (20–140 ppm; log₁₀ = 1.3–2.15) and Zr (80–700 ppm; log₁₀ = 1.9–2.85) observed across the three maps to allow direct comparison of variations among samples and eruptions. Color bars specify the element and the corresponding concentration range in ppm for each sample. The Python script used to visualize maps and produce this figure is provided in the Zenodo repository^73^.

Clinopyroxene trace-element maps reveal chemical complexities not visible in BSE images (Fig. 5). The cores often exhibit extreme heterogeneity with patchy domains, subgrains and/or sector zoning (Fig. 5). Core compositions span from primitive (high Cr, Ni, Sc; low Zr, Mn) to evolved (low Cr, Ni, Sc; high Zr, Mn), mirroring single-spot analyses. Contacts between cores and surrounding crystal portions are usually rounded, particularly around evolved cores (low Cr, Sc, Ni; high Zr, Mn). A key feature is the high Cr, Ni and Sc concentration of clinopyroxene inner rims, consistently present across all mapped clinopyroxene crystals, reaching up to 3500 ppm of Cr (Fig. 5 and Supplementary Fig. 11) and increasing from tephrite to basanite as observed for individual eruptions (Fig. 4G–I). The inner rims are either in contact with the groundmass directly or, more frequently, surrounded by thin outer rims characterized by a drop in Cr, Sc, and Ni and higher Zr, Ti, and Mn relative to inner rims (Fig. 5). Multiple high-Cr zones are observed in the zoning record of tephrite- and basanite-hosted clinopyroxene crystals from Teneguía 1971 and Tajogaite 2021, whereas in El Charco 1712 they are restricted to single inner rims (Supplementary Fig. 11).

### Matrix major and trace element composition

**Fig. 6:**
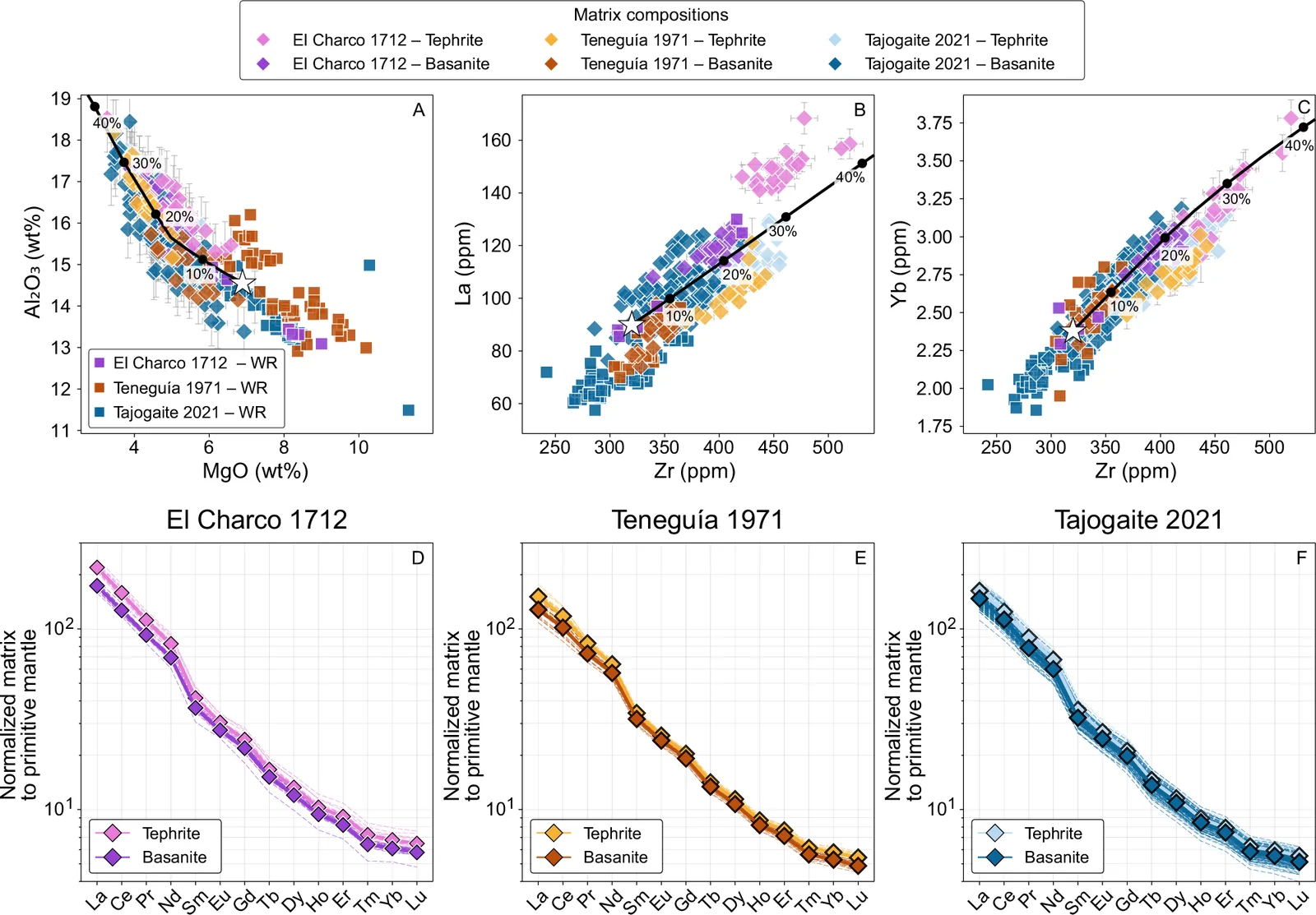
Major and trace element composition of the microcrystalline matrix from El Charco 1712 and Teneguía 1971 (this study) and Tajogaite 2021^21^. **A** Variation of MgO vs Al_2_O_3_. **B** Variation of Zr vs La and (**C**) Zr vs Yb concentrations. Matrix data are compared to recent whole-rock (WR) major and trace element data from the literature published after 2000 for El Charco 1712^50,84^, Teneguía 1971^15,44,50,84^ and Tajogaite 2021^21,81^. The black line in A-C shows the MAGEMin^35^ thermodynamic-based FC model at 10% increments, starting from a primitive matrix composition (white star). See Supplementary Table 1 for modeling details. **D**–**F** REE patterns of matrix composition, normalized to the composition of primitive mantle^85^. Symbols are color-coded according to tephrite and basanite samples. For major elements, error bars represent 2σ accuracy estimated from BHVO-2G replicate analyses; for trace elements, error bars denote the internal 2SE analytical uncertainty.

The microcrystalline matrix from the Teneguía 1971 and El Charco 1712 eruptions ranges in composition from basanite to tephrite, overlapping matrix results from the Tajogaite 2021 eruption^21^ (Supplementary Fig. 12). The mean MgO content of the matrix is 4.9 ± 0.7 wt% and 4.8 ± 0.9 wt% in El Charco 1712 and Teneguía 1971, respectively, similar to the matrix from the 2021 Tajogaite eruption of 4.7 ± 0.7 wt%^21^ (Fig. 6A).

Tephrite matrices display slightly higher Zr and incompatible trace element concentrations than basanites, consistent with a more evolved character (Fig. 6B, C). Basanite compositions from the Tajogaite 2021 and Teneguía 1971 eruptions are similar and define overlapping fields (Fig. 6B, C). In contrast, basanites from El Charco 1712 are more evolved, plotting close to the tephrite fields of the other eruptions. The El Charco 1712 tephrite matrix is also the most evolved, reaching the highest Zr and incompatible element concentrations (Fig. 6B, C), consistent with its most enriched REE patterns (Fig. 6D-F).

### Pressure and temperature of clinopyroxene crystallization

**Fig. 7:**
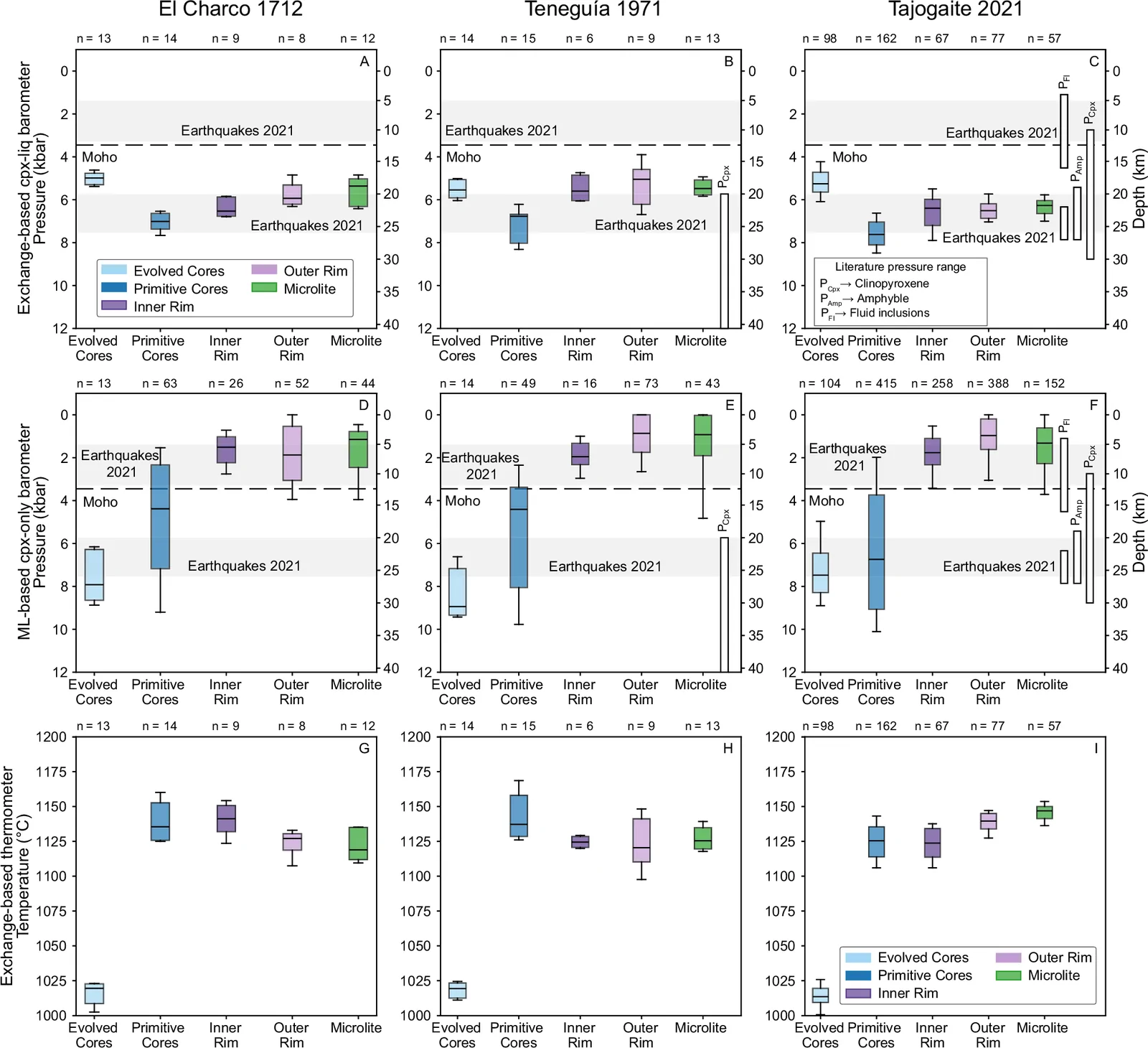
Clinopyroxene thermobarometry results. **A**–**C** Exchange-based clinopyroxene-liquid barometry, (**D**–**F)** ML-based clinopyroxene-only barometry and (**G**–**I**) Exchange-based clinopyroxene-liquid thermometry results for the different eruptions illustrated as box plots. Data are grouped in boxplots and color-coded according to individual textural positions. The box represents the interquartile range (25th to 75th percentile), the horizontal line indicates the median, and the whiskers extend to the 10th and 90th percentiles. The number of estimates that pass the equilibrium criteria is indicated for each textural category. Gray fields in (**A**–**F)** panels indicate the location of crustal and upper mantle seismicity before the 2021 Tajogaite eruption^32^. White rectangles indicate literature pressure ranges based on clinopyroxene (P_Cpx_), amphibole (P_Amp_) and fluid inclusions (P_FI_). Pressures were converted into depths using a crustal density of 2800 kg/m^3^ and 3100 kg/m^3^ above and below the Moho, respectively^31,86^.

Clinopyroxene–liquid exchange-based barometers^29,30^ (see “Methods”) indicate that primitive clinopyroxene cores exhibit the greatest pressure variability within individual eruptions, extending down to 9.4 kbar (32 km) (Fig. 7A–C and Supplementary Fig. 13). Most primitive core pressures, hereafter defined as within the 10th–90th percentile, range from 6.4–7.7 kbar (22.2–26.5 km) for El Charco 1712, 6.2–6.9 kbar (21.5–23.9 km) for Teneguía 1971, and 6.6–8.6 kbar (22.9–29.4 km) for Tajogaite 2021. Evolved cores (Mg# <67) record lower pressures, mostly 4.3–6.1 kbar (15.3–21.2 km), than primitive cores, with no systematic differences across eruptions (Fig. 7A–C). Inner rims, outer rims, and microlites show broadly similar pressure distributions across all eruptions, indicating crystallization in the pressure range 5.0–7.0 kbar (17.6–24.2 km) (Fig. 7A–C). Overall, clinopyroxene–melt barometry indicates upper mantle storage (below the local Moho at 10–14 km^31,32^). No pressure differences are observed across eruptions or clinopyroxene textural positions beyond calibration uncertainties, except for some primitive cores which likely crystallized deeper (>7 kbar) than other textural categories (Fig. 7A–C).

Machine-learning (ML)-based clinopyroxene–only barometry^33^ supports crystallization of evolved and primitive cores predominantly in the upper mantle, with evolved cores recording similar pressures within uncertainty, or recording deeper levels, and primitive cores spanning a broader pressure range (Fig. 7D–F). However, ML barometry also suggests differences in crystallization depth across textural positions. Specifically, it indicates that inner rims, outer rims, and microlites crystallized within the crust across eruptions, within the pressure range 1.0–2.5 kbar (3.6–9 km; Fig. 7D–F).

In terms of crystallization temperature, clinopyroxene-liquid thermometry^29,30^ returns comparable results across eruptions for primitive cores (1107-1145 °C), inner rims (1129-1155 °C), outer rims (1114–1147 °C) and microlites (1118–1153 °C). The evolved cores record the lowest temperatures (1000–1025 °C), with no significant differences across the three eruptions (Fig. 7G–I and Supplementary Fig. 14). The same pattern is recorded by ML-based thermometry^33^, with evolved cores extending to lower temperatures than other textural positions (Supplementary Fig. 15).

## Discussion

### Limited temporal variation of tephrite-basanite carrier melts

The microcrystalline matrix represents a crystal-free proxy for carrier melts, unlike whole-rock compositions, which are commonly influenced by recycled crystals^21,34^. Major element homogeneity over time (~5 wt% MgO, in agreement with the filtering of OIB melts to eruptible liquids^34^) is mirrored in the trace elements, as matrix compositions from all three eruptions define a single array in incompatible trace element space (Fig. 6). The only notable difference is the more evolved trace element character of tephrite melts from the El Charco 1712 eruption. REE patterns are parallel across all eruptions, indicating compositions controlled by fractional crystallization (FC). Major and trace element FC thermodynamic-based modeling using MAGEMin^35^ (see Supplementary Information) shows that within each eruption, the transition from basanite to tephrite requires only ~10–20% fractionation (Fig. 6A–C), consistent with previous Rayleigh trace element modeling at Tajogaite^21^. The more evolved El Charco 1712 matrices require slightly higher degree of fractionation (~20–30% fractionation; Fig. 6A–C) which does not appear to correlate with pre-eruption repose time. Only 35 years separated El Charco 1712 from the previous eruption (San Antonio 1677), less than the 50-year break preceding Tajogaite 2021 and more than the 22 years preceding Teneguía 1971. Instead, the more evolved character of El Charco 1712 carrier melts may reflect limited mafic recharge into the system, allowing greater crystal fractionation and melt differentiation. This is supported by the comparatively limited evidence for high-Cr recharge in mapped clinopyroxenes from El Charco 1712, in contrast to the repeated high-Cr recharge signatures identified in clinopyroxenes from Teneguía 1971 and Tajogaite 2021 (Supplementary Fig. 11).

### Temporal persistence of upper mantle magma storage and transient crustal stalling

Thermobarometers applied on our dataset and published compositions return consistent clinopyroxene crystallization depths and temperatures for each textural group within individual eruptions and across historical time, within uncertainties of the applied methods (Fig. 7). Regardless of the calibration used, clinopyroxene cores consistently record crystallization in the upper mantle beneath Cumbre Vieja (Fig. 7 and Supplementary Fig. 13), at ~18–25 km depths. These depths are consistent with the location of the deep seismicity recorded before and during the 2021 Tajogaite eruption at 20–25 km depth^32^. Crystallization temperatures are consistently around 1100–1150 °C, except for the evolved cores which record lower temperatures of 1000–1025 °C (Fig. 7 and Supplementary Fig. 14).

Our results broadly align with previous barometry on the 2021 Tajogaite eruption. Application of a range of exchange-based calibrations on clinopyroxenes^21,23^ and amphiboles^16,23^ supports upper mantle extraction depths of crystals, broadly spanning 10–27 km, regardless of the textural position. Romero et al.^36^ report higher clinopyroxene crystallization pressures between 7.6–11.7 kbar (26-40 km), similar to those calculated by Castro and Feisel^37^, in the range 7–10 kbar (24–34 km) and to those obtained by Chamberlain et al.^16^, where mean pressures for clinopyroxene cores, rims and microlites are between 7.3–8.6 kbar (25–29 km). These studies used the calibration of Neave and Putirka^38^, which is not specifically designed for mafic alkaline magmas and is noted here to yield higher pressure estimates for Tajogaite 2021 compared to barometers calibrated for such compositions (Supplementary Fig. 13).

Upper mantle extraction depths are corroborated by olivine-hosted melt and fluid inclusion studies during the 2021 Tajogaite eruption, which record magma storage depths between 15–30 km^39,40^. Similarly, microthermometry on fluid inclusions from Tajogaite 2021 indicates magma storage at depths of 22 to 27 km, in addition to a shallower transient zone at 4 to 16 km depth^24^. Interestingly, the application of ML-based clinopyroxene-only thermobarometry^33^ (Fig. 7D–F) provides a complementary perspective, possibly resolving differences in crystallization pressures among textural domains. Unlike clinopyroxene-liquid exchange-based methods, the ML approach does not require an equilibrium liquid composition, allowing pressure estimates for individual textural domains where the equilibrium liquid is unavailable or must be assumed. Specifically, ML barometry indicates that evolved and primitive cores predominantly crystallized in the upper mantle, broadly in line with clinopyroxene–liquid exchange-based barometers. In contrast, inner rims formed mainly within the crust (∼5–10 km), possibly during transient crustal storage suggested by fluid inclusion data^24^, together with experimental constraints^41,42^ and crustal seismicity at 4–12 km depth recorded during the 2021 Tajogaite eruption^32,43^. Outer rims and microlites record similar or slightly shallower depths, potentially indicating crystallization upon final ascent and eruption (∼0–7 km) (Fig. 7D–F).

Previous studies using clinopyroxene–liquid exchange-based methods on the Teneguía 1971 eruption report storage depths of 20–45 km^15,44^. These estimates are deeper than those obtained in our study, likely because they used whole-rock compositions instead of matrix or glass, which typically result in overestimated pressures and temperatures^45^. Additionally, we note that the Teneguía 1971 clinopyroxene compositions for these studies^15,44^, acquired using the same EMPA instrument, show systematically lower CaO and slightly higher Na₂O contents compared to our data (Supplementary Fig. 3). These differences may reflect a calibration issue affecting CaO and Na₂O measurements (Supplementary Fig. 3), with lower CaO and higher Na₂O in those datasets potentially explaining the higher-pressure estimates. The 1949 San Juan eruption was also fed by a magma reservoir located at 21-27 km or deeper, according to clinopyroxene-liquid barometry^28^. Fluid inclusions in olivine and clinopyroxene crystals from the 1949 San Juan eruption record entrapment pressures of 6.0–6.8 kbar (21–24 km) and 2–3.4 kbar (8–12 km)^46^. Overall, published clinopyroxene, fluid and melt inclusion thermobarometry on historical eruptions is consistent with findings from the 2021 Tajogaite eruption and our work, revealing temporally consistent upper mantle magma storage and transient crustal processing of eruption-feeding magmas under Cumbre Vieja.

### Clinopyroxene records of magma history: Coupling clustering with texture, chemistry, and thermobarometry

**Fig. 8:**
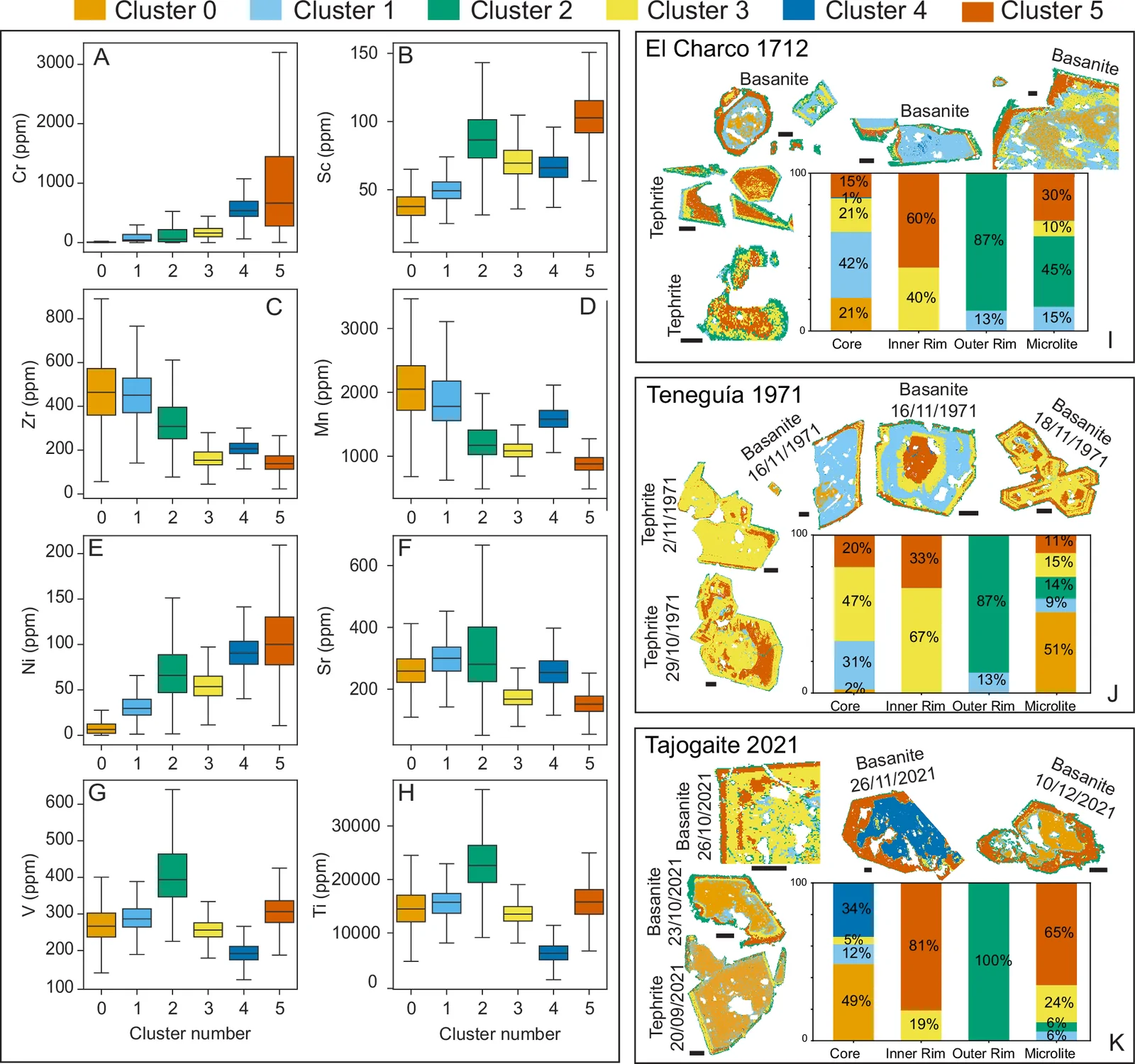
Clinopyroxene trace element clustering results and cluster-colored maps. **A**–**H** Boxplots showing the clustering results for Cr, Sc, Zr, Mn, Ni, Sr, V and Ti. The boxes represent the interquartile range (25th–75th percentile), the horizontal line indicates the median, and the whiskers extend to the most extreme data points within 1.5 times the interquartile range from the lower and upper quartiles. Each cluster is assigned a distinct color, which is used to distinguish clusters in panels **I**–**K** and in Fig. 9. **I**–**K** summary and visualization of the clustering results for (**I**) El Charco 1712, (**J)** Teneguía 1971, and (**K**) Tajogaite 2021. Each panel includes: (1) cluster-colored maps of clinopyroxenes, indicating whether they are hosted in tephrite or basanite samples and, where applicable, the eruption date. The black bar in each map indicates a scale bar of 100 µm; (2) stacked bars displaying, for each textural position, the proportions of the different clusters.

**Fig. 9:**
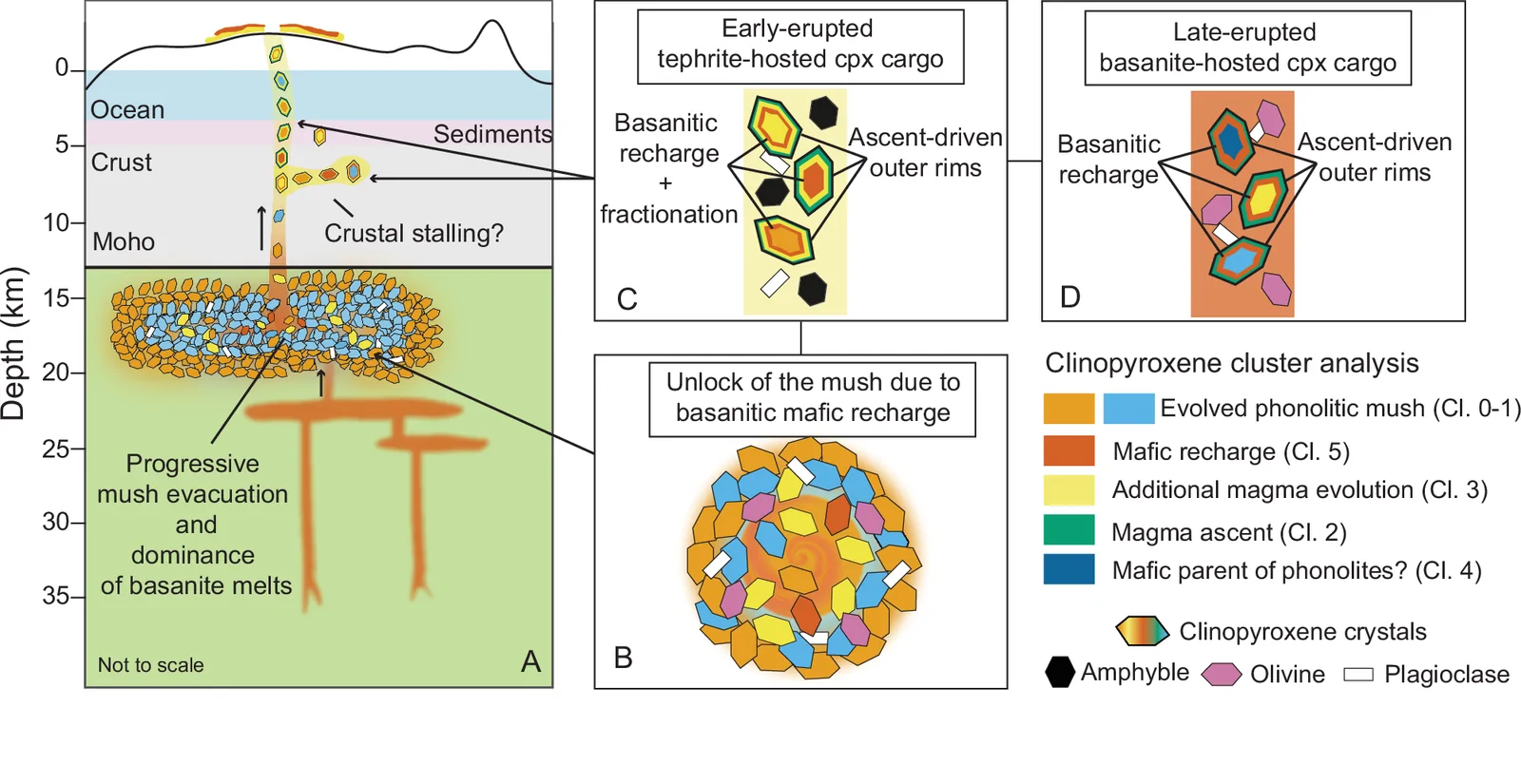
Conceptual cartoon of the La Palma plumbing system, summarizing the main findings of this study. **A** Schematic illustration of the magmatic plumbing system beneath Cumbre Vieja at La Palma prior to and during the El Charco 1712, Teneguía 1971 and Tajogaite 2021 eruptions. **B** Basanite melts recharge a tephri-phonolitic to phonolitic crystal mush reservoir in the upper mantle, leading to partial disaggregation and recycling of the resident antecrystic mush. This recharge process rejuvenates the mush system and produces resorbed clinopyroxene cores in the zoning record. **C** Following mush recycling, the magma may undergo transient stalling in the crust, modulating crystallization of the inner rims. At this depth, magma fractionates into tephrite that erupts first and creates specific zoning patterns reflected in the early-erupted clinopyroxene record, characterized by tephritic inner rims (Cluster 3) preceded by basanite compositions (Cluster 5). During ascent and/or surface cooling, outer rims (Cluster 2) crystallize. **D** As the eruption proceeds, an increasing contribution of mafic magma drives evacuation of  the upper mantle mush. Cl: Cluster number.

In the following sections, we apply cluster analysis, an unsupervised ML technique, to clinopyroxene zoning to identify distinct geochemical groups that guide subsequent petrological interpretation. These clusters are integrated with textural observations, major and trace element chemistry, and thermobarometric constraints, aiding the linkage between compositional domains, crystallization conditions, and magmatic processes. Among the clustering solutions explored, the six-cluster solution was selected as the most interpretable and geologically meaningful representation of the dataset (see “Methods” and Supplementary Information). Accordingly, clinopyroxene trace element compositions are subdivided into six clusters (Cl 0 to 5; Fig. 8), which record distinct pre-eruptive histories from magma storage to ascent and eruption (Fig. 9). In summary, clinopyroxene Clusters 0 and 1 have the highest Zr and Mn and the lowest Cr, Sc, and Ni in the dataset, along with the most enriched REE patterns. Cluster 2 is defined by elevated Ti, V, Al_2_O_3_, and Sc, with low Cr. Cluster 3 broadly resembles Cluster 5 but shows lower Cr, Sc, and Ni and a slightly more fractionated REE signature. Cluster 4 is rare in the dataset and stands out for its uniquely low Ti, V, and Al_2_O_3_, paired with moderately high Cr and Ni. Cluster 5 represents the most primitive group, with the highest Cr, Ni, and Sc, the lowest Zr and Mn, and the most depleted REE patterns.

### Evolved, cold tephri-phonolitic to phonolitic mushes in the upper mantle

Clinopyroxene clusters 0 and 1 are characterized by the highest Zr and Mn contents and the lowest Cr, Sc, and Ni concentrations (Fig. 8A–H). Cluster 1 differs from Cluster 0 by slightly higher Cr, Sc, and Ni. Both clusters show the most enriched REE patterns (Supplementary Figs. 16 and 17). They occur exclusively in clinopyroxene cores and often form patchy domains (Fig. 8I–K). When present in other textural positions, they are restricted to microlite cores (Fig. 8I–K), likely representing core remnants of larger crystals. The exclusive occurrence of Clusters 0 and 1 in mineral cores and their resorbed textures suggest crystallization at depth, from melts no longer present at the time of eruption. We interpret these clusters as representing evolved and fractionated melt compositions growing patchy cores with relatively low Mg# values (Supplementary Fig. 18). These clinopyroxene compositions are in major- and trace element disequilibrium with the tephrite-basanite carrier melts (Supplementary Figs. 2 and 19) and define a separate lineage in the Mg–Na–Fe2 + (+Mn) diagram^47^ compared to tephrite- and basanite-related compositions, suggesting a different origin (Supplementary Fig. 21). We therefore infer that Clusters 0 and 1 represent clinopyroxene antecrysts that did not crystallize from carrier liquids but instead formed in evolved melt pockets within a crystal mush (Fig. 9).

To investigate the nature of these evolved melt pockets, we calculate the trace element composition of melts in equilibrium with clinopyroxene, using partition coefficients (Kd) derived from multiple parameterizations^48^ (Supplementary Table 1). The reconstructed melt compositions define a trend broadly consistent with fractional crystallization, with Clusters 0 and 1 representing the most differentiated compositions (Supplementary Fig. 19D–F). Despite uncertainties from using fixed Kd values, our fractional crystallization modeling, starting from a primitive basanite matrix composition, indicates that up to 80–90% crystallization is required to produce the most evolved reconstructed melt compositions represented by Clusters 0 and 1 (Supplementary Fig. 19). This aligns with the ~80-85% crystallization estimated to generate phonolitic magmas from basanitic magmas beneath La Palma^49,50^ and OIB settings in general^51^. This is also supported by our thermodynamic-based FC model, which indicates the generation of tephri-phonolite and phonolite compositions after 80-90% fractional crystallization from a basanitic melt (Supplementary Fig. 20). We also observe fractionation of MREE (Y) and, to a lesser extent, HREE (Yb) at fixed Zr in Clusters 0 and 1, with these elements showing lower concentrations in reconstructed melt compositions relative to LREE (La, Supplementary Fig. 19). This likely reflects increased partitioning of MREE and HREE into amphibole and clinopyroxene during fractionation of evolved alkali melts, depleting the residual melts in these elements^52^. Additionally, most of the REE patterns of clinopyroxene from Clusters 0 and 1 show an upward inflection of HREE (Tm, Yb, Lu) compared to light REE. This is a typical behaviour of high-Na clinopyroxene crystallizing from phonolitic melts, where HREE preferentially partition into the M1 site, whereas other REE remain mostly distributed in the M2 site (refs. ^18, 53^). Altogether, these observations support the occurrence and recycling of a tephri-phonolitic to phonolitic mush under Cumbre Vieja at La Palma. This is consistent with the occurrence of plagioclase antecrysts in a 2021 basanite sample, interpreted to be recycled from evolved mushes^18^, as well as tephriphonolite xenoliths in previous eruptions^19^.

Understanding the depth of crystallization of phonolitic-derived Clusters 0 and 1 clinopyroxene cores is challenging, as the exact composition of the melts from which they crystallized is unknown. Using exchange-based calibrations specific to phonolitic magmas^54^, Jegal et al.^18^ estimated pressures of 5.4 ± 0.8 kbar and temperatures of 1021 ± 6 °C for a green evolved clinopyroxene included in a plagioclase antecryst. Building on this approach, we obtain crystallization depths for evolved clinopyroxene from El Charco 1712, Teneguía 1971 and Tajogaite 2021 of 4.8 ± 0.8 kbar (17 ± 2.9 km), 5.1 ± 0.4 kbar (18 ± 1.5 km) and 5.0 ± 0.8 kbar (18 ± 2.9 km). Mean temperatures of evolved clinopyroxene for the same eruptions are 1021 ± 13 °C, 1018 ± 8 °C and 1019 ± 12 °C, respectively (Fig. 7). Similar estimates are obtained when using ML-based thermobarometers on evolved cores (Supplementary Fig. 15), strengthening our results and placing evolved clinopyroxene crystallization in cold upper mantle storage regions (Fig. 9), as also inferred for green, evolved clinopyroxene cores at other OIB volcanoes^55^.

### Mafic magma recharge and subsequent differentiation

Cluster 5 displays the highest Cr, Ni, and Sc and the lowest Zr and Mn concentrations (Fig. 8A–H). It is the most depleted in REE and the least fractionated, with Mg#>67 (Supplementary Figs. 16 and 18A). Cluster 3 is similar to Cluster 5 but is distinguished by lower Cr, Sc, and Ni concentrations (Fig. 8) and a slightly more fractionated character (Supplementary Fig. 16). In general, Clusters 3 and 5 are found in primitive cores, inner rims, and microlites, all with Mg# >67 (Supplementary Fig. 18A). Their major and trace element compositions are in chemical equilibrium with carrier melts, suggesting that they are true phenocrysts (Supplementary Figs. 2 and 19).

In basanite-hosted clinopyroxenes, Cluster 5 consistently characterizes the inner rims across all eruptions, typically forming a homogeneous euhedral zone (Fig. 8I–K). In contrast, in earlier tephrite-hosted clinopyroxenes, inner rims are characterized by the more fractionated Cluster 3, preceded by Cluster 5 compositions (Fig. 8I–K). The pattern that emerges from these observations is that inner rims are commonly associated with clusters 3 and 5, which define reverse zoning relative to cores.

We estimate inner rim crystallization timescales using measured rim thicknesses and clinopyroxene growth rates for mafic alkaline systems. In the absence of experimental constraints for Cumbre Vieja, we adopt a growth rate of ~10⁻⁸ cm/s from Mt Etna (Italy)^56^, which has similar mafic alkaline compositions. Inner rims display consistent thicknesses across eruptions (48 ± 10 µm in 2021; 41 ± 8 µm in 1971; 48 ± 8 µm in 1712), corresponding to crystallization timescales of ~4–7 days. These timescales reflect the interval from inner rim formation to eruption only if outer rims crystallized immediately thereafter. Because this relationship is not known and inner rims may have been stored under near-equilibrium conditions prior to formation of outer rims, we interpret these estimates as minimum timescales^16,56^. Hence, the dominance of primitive inner rims suggests mafic magma recharge occurred at least 4–7 days prior to crystallization of outer rims, similar to observations at other alkaline basaltic volcanoes (e.g., Etna^10,57^).

We interpret Cr-rich Cluster 5 as representing pre-eruptive basanitic recharge that disrupts and resorbs the resident mantle-seated crystal mush (Fig. 9). Following mush disaggregation, the recharging basanitic melt carrying antecrysts from the evolved mush crystallizes to form inner rims, either at upper mantle conditions as suggested by clinopyroxene (this study and refs. ^16–21^) and amphibole^23^ exchange-based barometers and syn-eruptive deep seismicity^43^ or ascending into the crust as suggested by ML-based barometers (this study), syn-eruptive shallow seismicity^43^ and experimental constraints^41^. The lack of correlation between Cr and slowly diffusing elements such as Al argues against a boundary-layer origin. Instead, Cr enrichment coupled with elevated Mg# supports crystallization from a mafic recharge melt, with Cr zoning preserving the recharge signal^10,58^. As for Cluster 3, its trace element composition suggests crystallization from a magma that fractionated following mafic recharge. It thus represents a slightly more fractionated flavor of Cluster 5, probably linked to the tephritic magma (Fig. 9).

A key observation is that in early-erupted tephrite-hosted clinopyroxene, Cluster 3 dominates inner rims, which consistently overgrow Cluster 5 compositions, suggesting fractionation and cooling, or mixing with resident tephrite, following mafic recharge prior to eruptions. In contrast, in late-erupted basanite-hosted clinopyroxene, Cluster 3, when present, generally precedes the final mafic recharge event in the zoning record (Fig. 8I-K). Although we recognize the importance of mafic recharge in recycling mantle-seated crystal mushes, the zoning patterns of early-erupted clinopyroxenes suggest that recharge did not act as the immediate eruption trigger in the eruptions studied here. Cluster 5 signatures precede Cluster 3 in inner rims, suggesting that recharge was followed by cooling. Hence, mafic recharge unlocked the mush in the upper mantle but did not act as an immediate eruption trigger. Instead, eruption onset was probably associated with progressive pressurization of the reservoir during subsequent magma evolution, most plausibly as volatile exsolution accompanying fractionation increased internal overpressure until failure was reached^1^. This interpretation is consistent with observations from the 2021 Tajogaite eruption, for which phase-equilibrium experiments and olivine zoning indicate pre-eruptive cooling of the tephrite magma^16,41^.

Despite lacking precise temporal constraints for the El Charco 1712 samples, tephrite samples erupted earlier than the basanites^27^, similar to the 1971 Teneguía^26^ and 2021 Tajogaite eruptions^21^. To explore the temporal variation of mafic recharge in the three eruptions, we examined Cr concentrations in inner rims and microlites (Fig. 4G–I). Cr concentrations are significantly lower in the early-erupted tephrite-hosted clinopyroxene inner rims and microlites compared to those in basanite-hosted clinopyroxene. This pattern is consistent with observations from the 2021 Tajogaite eruption, where there is evidence for a progressive and gradual increase in Cr concentration of recharge melts over the course of the eruption (Fig. 4I)^21^, and other recent basaltic eruptions where similar datasets are available (e.g., Mt Etna 2021 paroxysms^57^). Hence, considering similarities with the 2021 Tajogaite eruption, we argue that also during El Charco 1712 and Teneguía 1971, there was a progressive input of mafic magma that gradually evacuated and recycled a tephri-phonolitic to phonolitic crystal mush in the upper mantle (Fig. 9).

### Cryptic primitive seed of the phonolitic lineage

Cluster 4 is characterized by the lowest Ti, V and Al_2_O_3_ concentrations, coupled with relatively high Cr and Ni contents, although lower than those of Cluster 5 (Fig. 8 and Supplementary Fig. 18). Cluster 4 is rare, occurring as single-spot data in one core from Teneguía 1971 and one core from Tajogaite 2021. In mapped clinopyroxenes, it appears as a large, resorbed core in one crystal from Tajogaite 2021 and as patches within a Cluster 1 core from El Charco 1712. Despite its sporadic occurrence and geochemical characteristics, Cluster 4 is consistently confined to mineral cores and likely represents a melt composition that crystallized at upper mantle depth. The lower Cr concentrations relative to Cluster 5 and the highly resorbed core mapped in Tajogaite 2021 (Fig. 8K) suggest crystallization from a less mafic melt than the recharge magma, followed by partial dissolution during interaction with the recharging melt. The low Ti, V and Al_2_O_3_ contents of Cluster 4 suggest crystallization at low degrees of undercooling, in a relatively stable magmatic environment, because increasing undercooling would favor incorporation of Al, Ti and possibly V due to crystallization kinetics^59,60^.

Importantly, the major element composition of Cluster 4 clinopyroxene cores is distinctly Mg#-rich and TiO_2_-Al_2_O_3_-poor, plotting below the rim-microlite trend in bivariate plots (Fig. 3 and Supplementary Fig. 3). The transitional nature of Cluster 4 between basanitic (Cluster 3 and 5) and phonolitic (Cluster 0) clinopyroxene is confirmed in the Mg–Na–Fe²⁺(+Mn) ternary diagram^47^, which is particularly appropriate for clinopyroxene from evolved alkaline magmas^61^ (Supplementary Fig. 21). This highlights that Cluster 4 cores fall at the start of the phonolitic trend, distinct from the basanite-tephrite trend. Textural, major and trace element results therefore suggest that Cluster 4 records a distinct primitive composition, possibly capable of evolving into phonolitic melts.

### Magma ascent through the plumbing system

Cluster 2 is marked by the highest Ti, V and Al_2_O_3_ concentrations, along with elevated Sc levels, while Cr is low, similar to Cluster 1 (Fig. 8 and Supplementary Fig. 18). Cluster 2 is predominantly found in outer rims, together with a minor proportion of Cluster 1 in El Charco 1712 and Teneguía 1971 samples. Microlites also show Cluster 2, but only in their outermost portions (Fig. 8I-K). Therefore, Cluster 2 is related to the very final crystallization process, and its increased uptake of Al-Ti is consistent with crystallization at high degrees of undercooling during magma ascent^10,60^. Outer rims are thinner than inner rims, with average thicknesses of 11 ± 3 µm (2021), 13 ± 3 µm (1971), and 24 ± 7 µm (1712). Assuming constant growth rates as discussed above, these thicknesses correspond to crystallization timescales in the order of 1–3 days, immediately preceding the eruption and following reservoir failure. The Cr-poor, Al-Ti-rich character of Cluster 2, its textural dominance in outer rims and microlite rims, and its crystallization at shallow depths according to ML-based barometry, are consistent with formation during magma ascent and/or surface crystallization upon lava emplacement (Fig. 9), as also suggested at Etna volcano^10,57^.

### Implications for low-flux OIBs worldwide

Our findings from historical eruptions at Cumbre Vieja in La Palma underscore two key aspects with implications for low-flux ocean island basalt volcanoes globally: (1) the temporal consistency of geochemical characteristics and magma storage depths across different eruptions, and (2) the recycling of mantle-seated tephriphonolitic to phonolitic crystal mushes by mafic recharge magmas.

First, cluster analysis identifies recurrent geochemical groups preserved across the three Cumbre Vieja eruptions studied here, indicating stable magmatic differentiation pathways over several centuries. Recharge–fractionation trends in major and trace element space are also constant through time (Figs. 3 and 4). Magma storage pressures and temperatures remain largely invariant across the recent Cumbre Vieja eruptions, supporting persistent temporal magma storage (Fig. 9). Similar observations have been reported for other low-flux OIB volcanoes^55,62–66^, supporting the view that magma accumulation and differentiation in such systems predominantly occur in the upper mantle. This is consistent with the broader pattern that low-flux OIB volcanoes (e.g., Cape Verde, Canary Islands, Azores, Galapagos) tend to exhibit deeper, upper-mantle storage, as opposed to high-flux systems (e.g., Kilauea, Piton de la Fournaise, Iceland) which commonly develop shallower crustal reservoirs^3^. As a consequence, a significant portion of magma evolution in low-flux OIB systems may occur at depths that are poorly resolved by conventional geophysical monitoring.

**Fig. 10:**
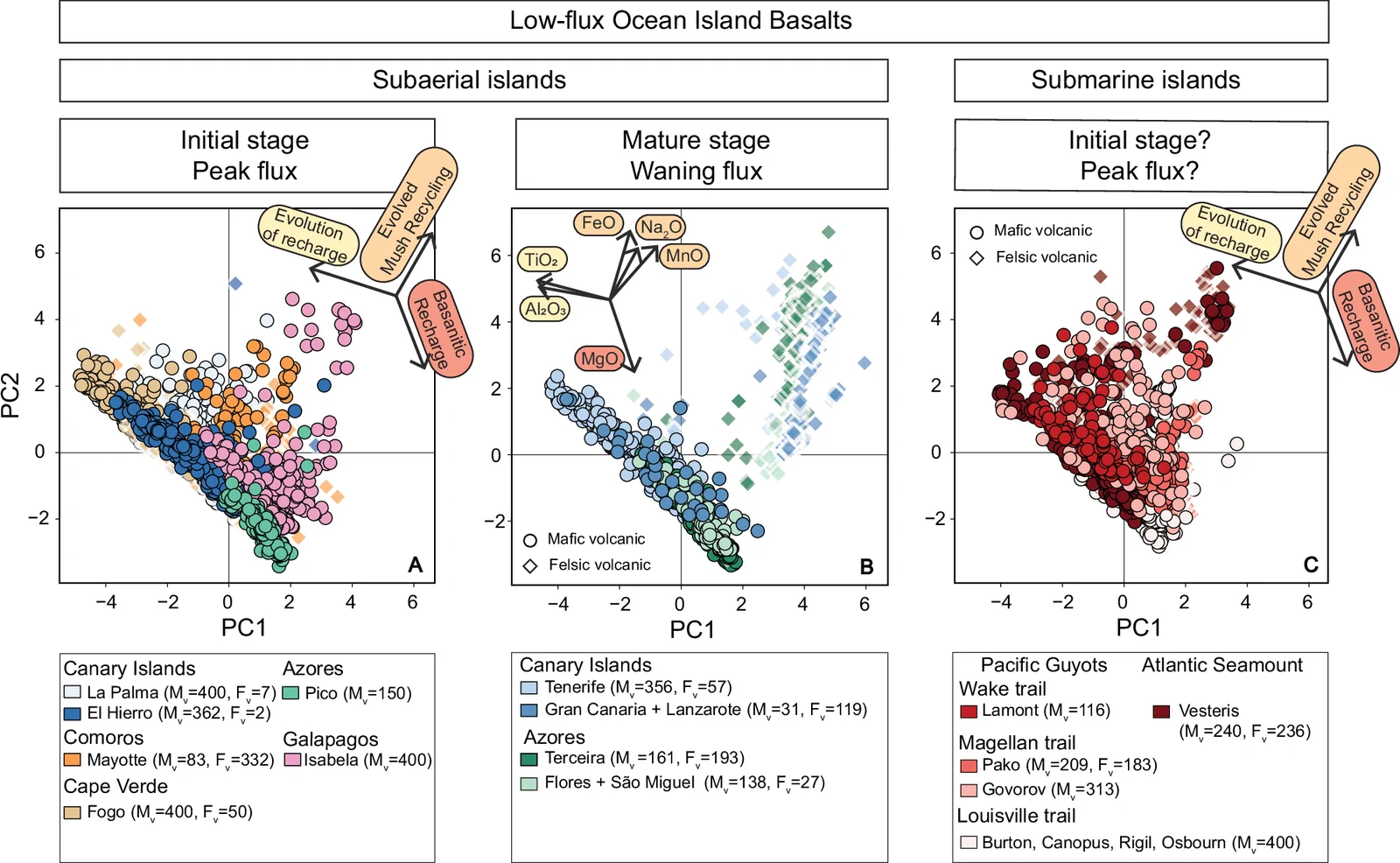
Comparison of clinopyroxene major element compositions from low-flux OIB systems worldwide in principal component analysis (PCA) space. Clinopyroxene data are grouped according to the evolutionary stage of the volcanic island and flux regime: (**A**) subaerial islands in their initial stage, including La Palma, (**B)** subaerial islands in the mature stage and (**C**) large submarine islands. The principal vectors of compositional variability across the dataset define three main trends in PCA space: (i) increasing MgO, interpreted as basanitic mafic recharge; (ii) high Al₂O₃ and TiO₂, reflecting magma ascent; (iii) high FeO, Na₂O, and MnO, associated with recycling of clinopyroxene antecrysts from evolved mushes. Filled circles with black outlines represent clinopyroxene from mafic rocks, whereas faded filled diamonds with white outlines indicate clinopyroxene from felsic rocks. In panels A and B, to reflect the current evolutionary stage of each volcanic system, only clinopyroxene from Holocene mafic volcanic (M_v_) rocks are shown, while clinopyroxene from felsic volcanic (F_v_) rocks are included for comparison regardless of age (see Supplementary Information for more details on data source and filtering).

Second, our data show that tephri-phonolitic to phonolitic crystal mushes can develop in the upper mantle beneath Cumbre Vieja at La Palma and subsequently be recycled by mafic recharge. To assess whether this process also occurs in other OIB systems, we compiled clinopyroxene major-element data from well-characterized low-flux OIB localities (Fig. 10). We focused on clinopyroxene data from Holocene eruptions and examined clinopyroxene hosted in both mafic and felsic lithologies (Fig. 10, and Supplementary Table 2). Within low-flux OIBs, we broadly categorized the data according to the evolutionary stage of the volcanic island and its relative flux regime^67^: (1) subaerial islands in their initial evolutionary stage, characterized by peak magma flux conditions (e.g., Isabela, Galápagos; Fogo, Cape Verde; La Palma and El Hierro, Canary Islands; Pico Island, Azores); (2) mature subaerial systems characterized by waning magma flux conditions (e.g., Gran Canaria and Tenerife, Canary Islands; Terceira, Flores and Sao Miguel, Azores); (3) large submarine seamounts, possibly in their initial stage. Given the high dimensionality of the dataset, we used Principal Component Analysis (PCA) to identify the main vectors of compositional variability (see Methods and Supplementary Information). Similar compositional distributions in PCA space may arise from a range of magmatic processes, including variations in melt compositions, crystallization conditions, and crystal recycling. With this in mind, we use PCA to compare first-order compositional relationships among OIB systems and explore whether the process documented at Cumbre Vieja may operate more broadly. In line with our findings at Cumbre Vieja, in PCA space, high MgO is consistent with clinopyroxene crystallizing from basanitic recharge melts, whereas high TiO_2_ and Al_2_O_3_ are consistent with fractionation of the recharge magma during magma ascent. In contrast, high FeO, Na_2_O and MnO are consistent with evolved mush antecrysts crystallized from differentiated melts that were subsequently recycled by recharge magmas (Fig. 10).

We find that most global OIBs in the initial stage, corresponding to peak magma flux, preserve clinopyroxene compositions in mafic lithologies that are consistent with, and may reflect recycling of an evolved mush, expressed as deviations from the main recharge–fractionation trend (Fig. 10A). Although we cannot exclude that these deviations reflect other processes, one interpretation is that mafic recharge may play a role in recycling and evacuating evolved mushes in low-flux OIBs in initial evolutionary stages, in which magma storage is often attributed to the lower crust^68^ and uppermost mantle^21,34,55,64,66,69^, broadly consistent with our findings of a tephri-phonolitic to phonolitic mush beneath Cumbre Vieja. In contrast, OIBs in a more mature stage, characterized by waning magma flux, lack this evolved-mush signature in mafic rocks, suggesting that the maturity of the system may regulate the development and/or incorporation of deep evolved mushes by mafic recharge. This raises the possibility that evolved melts may be widespread in the deep portions of OIB volcanoes worldwide, with flux regime likely imparting a first-order control on their formation and recycling.

For seamount settings, the lack of historical records makes it challenging to constrain the evolutionary stage and flux regime at the time of eruption. Clinopyroxene data from seamounts define a PCA distribution very similar to clinopyroxene compositions from peak flux OIBs (Fig. 10), supporting the idea that seamounts may represent initial stages of ocean volcanic islands. Despite this, while the evolutionary stage and flux regime appear to be a first-order control, we cannot exclude the possibility that the formation of evolved mushes in OIB systems may also be modulated by additional factors that merit further exploration.

In our model, the preservation of evolved mush signatures in mafic lithologies appears to be linked to early evolutionary stages and peak flux regimes of volcanic islands. Hence, we propose that during the initial stage, magma input might be sufficiently frequent to generate and maintain an upper mantle evolved mush that can be disaggregated by subsequent mafic recharge, yet the system has not yet developed the extensive shallow crustal plumbing that characterizes high-flux OIBs^3^. As the system matures, declining magmatic flux may allow the evolved mush to fully solidify between recharge events, erasing its signature in mafic lithologies (Fig. 10B). In such mature systems, shallow regions in the island edifice seem to be favored for the formation of evolved lithologies, which can reach high volumes (e.g., Tenerife, Azores). Similar links between waning magma flux favoring crustal magma storage and differentiation have been drawn in intraplate continental volcanoes^70^.

Regardless of the mechanisms controlling the occurrence of evolved mushes, our observations have important implications for OIB systems worldwide. We suggest that evolved melts can be generated and stored in the deeper volcanic system during initial evolutionary stages without requiring prolonged shallow crustal accumulation. Continued production and accumulation of such melts may eventually contribute to felsic eruptions tapped directly from near-Moho depths^51^. The cryptic nature of deep evolved mushes warrants further study, as it may pose challenges for volcanic hazard assessment: eruptions tapping these deep melts may occur with little or no precursory signals detectable at those depths by current geophysical monitoring methods.

## Methods

### Sampling and electron microprobe (EMPA)

Samples from the 1712 El Charco, 1971 Teneguía and 2021 Tajogaite eruptions consist of fresh lavas that transition from tephrites to basanites throughout the eruption (Fig. 1 and Supplementary Information). Samples from Tajogaite 2021 correspond to the same sample set published in Ubide et al.^21^ (Supplementary Data 1).

Clinopyroxene crystals from the different eruptions were analysed using three different electron microprobe analysers (EMPA). Data are reported in Supplementary Data 2. Tajogaite 2021 crystals were analysed at the Centro Nacional de Microscopía Electrónica of the Complutense University in Madrid, Spain, using a JEOL JXA-8900M electron microprobe equipped with four wavelength-dispersive spectrometers (WDS). Measurements were conducted using a beam current of 20 nA, an accelerating voltage of 15 kV and a beam diameter of 5 μm. Elemental counting times were 10 s on the peak and 5 s on background positions. Calibration standards included: albite for Na and Si; sillimanite for Al; microcline for K; almandine for Fe and Mn; kaersutite for Mg, Ca, and Ti; and pure metal elements for Ni and Cr. Data were corrected using the ZAF matrix correction procedure. The analyses were conducted in 2021 and 2022, following the same procedure of Ubide et al. (2023), as the clinopyroxene data presented here were acquired during the same analytical sessions. The EMPA data presented in this work complement the Ubide et al.^21^ dataset by adding crystal core and outer rim compositions to the previously reported crystal inner rim and microlite analyses.

El Charco 1712 crystals were analysed at the Unidad de Microscopía Electrónica of the University of Huelva, Spain, using a JEOL JXA-8200 electron microprobe, equipped with four WDS and one energy-dispersive X-ray spectrometer (EDS). Measurements were conducted using a beam current of 20 nA, an accelerating voltage of 15 kV and a beam diameter of 5 μm. Elemental counting times were set at 10 seconds on peak and 5 seconds on background for each element. Calibration standards included: K-feldspar for Al, Na, and K; wollastonite for Ca and Si; fayalite for Fe; forsterite for Mg; manganosite for Mn; chromite for Cr; rutile for Ti. Data were corrected using the ZAF matrix correction procedure.

Teneguía 1971 crystals were analysed at the Centro Nacional de Microscopía Electrónica of the Complutense University in Madrid, Spain, using a JEOL Field Emission Gun (FEG) JXA-iHP200F electron microprobe, equipped with four WDS. Measurements were conducted using a beam current of 10 nA, an accelerating voltage of 15 kV and a beam diameter of 5 μm or a focused (1 μm) beam. Elemental counting times were 10 s on the peak and 5 s on background positions. Calibration standards and data correction procedure are the same as for the JEOL JXA-8900M microprobe described above.

To assess data consistency across three microprobe instruments, we re-analyzed selected clinopyroxene crystals of the 1712 (30 analyses) and 2021 (38 analyses) samples using the JEOL JXA iHP200F electron microprobe at the Centro Nacional de Microscopía Electrónica (Madrid). Re-analyses were conducted at the same locations as the original analyses, identified visually using BSE images. Overall, re-analyses are consistent with the original measurements within analytical uncertainty (based on counting statistics), confirming the robustness and inter-instrument reproducibility of the dataset (Supplementary Figs. 22 and 23). Minor offsets observed in some analyses likely reflect minor positional offsets or patchy crystal domains. Data quality was monitored by analyzing clinopyroxene secondary standards from the Smithsonian Institution of Washington (Kakanui augite NMNH 122142, Cr-augite NMNH 164905 and Diopside NMNH 117733). Accuracy and precision determined using the Cr-augite standard NMNH 164905, which closely matches the composition of our samples, are better than 1–6% and 1–3% for major elements, respectively. For minor elements, accuracy ranges from 2–7% and precision from 1–8%, except for Mn, which exceeds 10%. Data of secondary standards are reported in Supplementary Data 3.

### Laser ablation-inductively coupled plasma mass spectrometry (LA-ICP-MS)

LA-ICP-MS was used to determine trace element compositions of clinopyroxene crystals via single-spot analyses (following Petrelli et al. (2016)^71^) (Supplementary Data 4), to acquire quantitative trace element maps of clinopyroxene (following Ágreda-López et al.^72^) (Zenodo repository https://doi.org/10.5281/zenodo.21081067^73^) and to measure major and trace element compositions of the microcrystalline matrix (following the rastering method of Ubide et al. 2023^21^) (Supplementary Data 6). LA-ICP-MS analyses were done at the Dipartimento di Fisica e Geologia, University of Perugia, using a 193 nm Analyte G2 laser ablation system operated with Chromium software coupled to a quadrupole iCAP-Q ICP-MS (Thermo Fisher Scientific) operated with Qtegra software. For all analyses, helium carrier gas was set to 0.6 and 0.3 L min⁻¹ for the ablation cell and cup, respectively.

Matrix compositions were analysed following the method described in Ubide et al.^21^, consisting of laser rastering over equigranular microcrystalline areas via overlapping spots. We used a raster spot size 50 μm diameter, with a repetition rate of 10 Hz and a horizontal scanning rate of 5 μm/s. Each raster consisted of five parallel horizontal transects, each 200–300 μm long, producing an ablated rectangular area. In total, 10 rasters were acquired per thin section in four thin sections (two basanite and two tephrite samples) from the El Charco 1712 and Teneguía 1971 eruptions. These data are complemented by published matrix analyses from Tajogaite 2021^21^, to which the reader can refer for full details on analytical conditions and matrix data reduction, which uses BCR-2G glass reference material as external standard and normalization to total 100 wt.% oxides as internal standard. For major elements, accuracy and precision were assessed using glass reference materials BHVO-2G and GSD-1G, resulting mostly in values < 5% and always better than 8% (Supplementary Data 7). For trace elements, accuracy was monitored using glass reference materials BHVO-2G, GSD-1G, NIST-610 and NIST-612 (Supplementary Data 8). Accuracy for BHVO-2G and GSD-1G is within 5–10% relative to accepted values, except for Ta which was ~13%. Precision was always <3% for both major and trace elements (Supplementary Data 7 and 8).

Trace element compositions of clinopyroxene single spots were acquired using a spot size of 30 μm and repetition rate of 10 Hz^71^. Data reduction was performed using Iolite^74^, using a 3D trace element calibration including NIST-SRM610, NIST-SRM612, GSD-1G and BHVO-2G as the calibrators, Ca as measured by EMPA as the internal standard, and USGS-BCR2G as the quality control. This configuration ensured precision and accuracy within ±5% for most analysed trace elements, with all elements exhibiting accuracy better than ±10% (Supplementary Data 5).

Compositional maps of clinopyroxene were acquired using oversampling, by overlapping laser squares to generate subsequent ablation lines that build the mapped area^72^. We used a spot size of 10 μm, resulting in a resolution of 5 μm^72^. Dosage was set at 10, scan speed at 100 µm/s, and the repetition rate at 100 Hz. We analysed 10 elements (Ca, Sc, Ti, V, Cr, Mn, Ni, Sr, Zr, Ce). NIST-SRM610 was used as a calibration standard and Ca was used as internal standard. Measurements of the secondary standard USGS-BCR2G alongside the unknown maps yielded accuracy better than 10% relative to preferred values for all elements, except Sc which was within 15%. Precision was better than 10% relative standard deviation, with most elements showing precision better than 5% (Supplementary Data 5). Raw data of clinopyroxene maps can be found in the Zenodo repository at (https://doi.org/10.5281/zenodo.21081067).

Data processing and image reconstruction of chemical maps were performed using HDIP software (Teledyne Photon Machines). HDIP was used to align and calibrate raw LA-ICP-MS signal intensities, reconstruct the spatial distribution of each element, and export fully calibrated concentration matrices (in ppm) of clinopyroxene crystals. This was done by manually segmenting clinopyroxene crystals within each map and removing the surrounding microcrystalline groundmass, mineral oxides, mineral inclusions, large cracks, and voids (Supplementary Fig. 26). Clinopyroxene matrices were subsequently processed and visualized using a Python script, archived in Zenodo at (https://doi.org/10.5281/zenodo.21081067).

### Principal component analysis (PCA)

Principal component analysis (PCA) is a multivariate dimensionality reduction technique that transforms correlated variables into orthogonal axes, capturing the dominant structure of variance in the dataset^75^; (1):

1 $$\sum w=\lambda w$$

where ∑ is the covariance matrix, w are the eigenvectors defining the principal components, and l are the corresponding eigenvalues representing the variance explained. PCA was applied in two parts. First, clinopyroxene major and minor element compositions (SiO₂, TiO₂, Al₂O₃, FeO, MgO, CaO, Na₂O, and MnO) from low flux OIB systems were projected into PCA space to reduce dimensionality and resolve the principal axes of compositional variation (Fig. 10). Second, PCA projections were used to evaluate the clustering structure and assess the optimal number of clusters for the clinopyroxene trace elements dataset (Supplementary Fig. 24).

### Clustering

Clustering is an unsupervised ML technique used to partition complex, high-dimensional datasets into groups (“clusters”) based on similarity in their multivariate features (refs. ^72, 76, 77^). This approach can identify similar chemical features by grouping together data that exhibit comparable elemental compositions. This can reveal patterns in large geochemical datasets that may correspond to distinct crystal zones, growth stages, or textural domains, without prior labels, thereby facilitating the subsequent petrologic interpretation^78^. Here, we applied k-means clustering to trace element concentrations in clinopyroxene from the three studied eruptions. The method partitions observations into *k* clusters by minimizing within-cluster variance (2):

2 $${\min }_{\{{C}_{k}\}}{\sum }_{k=1}^{K}{\sum }_{{xi}\,{\in C}_{k}}{{{{\rm{||}}}}x}_{i}-{{{{\rm{\mu }}}}}_{k}{{{{\rm{||}}}}}^{2} $$

where ${C}_{k}$ are clusters and ${\mu }_{k}$ their centroids, such that within-cluster variance is minimized under Euclidean distance. The input matrix combined single-spot analyses (*n* = 316) and quantified pixels from compositional maps (*n* = 398,776). We considered Sc, Ti, V, Cr, Mn, Ni, Sr, and Zr; Ce was excluded due to localized artifacts associated with cracks. Before clustering, concentrations were log-transformed and standardized using the StandardScaler function in scikit-learn, version 1.7.2 (3):

3 $$z=\frac{x-{{{\rm{\mu }}}}}{\sigma }$$

where x is the original variable, µ is its mean, and σ is its standard deviation, resulting in zero mean and unit variance. This ensures that elements with larger absolute concentrations did not dominate Euclidean distances. Values outside the 0.1–99.9 percentile range were excluded to remove analytical outliers and boundary artifacts. To objectively evaluate the number of clusters (k), we used PCA, t-distributed stochastic neighbor embedding (t-SNE), and evaluated solutions from k = 2 to 10 using the Elbow method^76^ (Supplementary Fig. 24). The Elbow method identifies a clear inflection at k = 6, indicating that reductions in within-cluster variance become progressively smaller beyond this point (Supplementary Fig. 24A). Projection of the cluster assignments into PCA space shows that the k = 6 solution resolves distinct compositional groupings along the principal axes of geochemical variability (Supplementary Fig. 24C), whereas visualization in t-SNE space reveals coherent local neighborhoods that are consistently recovered across the dataset (Supplementary Fig. 24D). Furthermore, single-spot analyses and pixel-based map data occupy the same compositional domains in both PCA and t-SNE space, indicating that the identified clusters reflect intrinsic geochemical populations rather than artefacts arising from differences in sampling density. While no single cluster number can be considered uniquely correct, the combined statistical analyses, together with petrographic and textural constraints, indicate that k = 6 provides the most interpretable and geologically meaningful representation of the dataset among the solutions tested (Supplementary Fig. 24).

To assess the influence of the imbalance between pixel-based map data and single-spot analyses, we created a balanced dataset combining all single-spot analyses and randomly downsampled map pixels (*n* = 1000), clustered it independently, and compared the result against the six-cluster solution from the full dataset via nearest-centroid matching. The results show good agreement, indicating that the identified clusters and their petrological interpretations are robust and not artifacts of the pixel imbalance (Supplementary Fig. 25). We therefore retain the six-cluster solution, obtained from the full dataset for subsequent petrological interpretation. The Python script used for clustering and evaluation of quantitative methods for the selection of k is provided in the Zenodo link (https://doi.org/10.5281/zenodo.21081067)^73^.

### Thermobarometry

Magma storage pressures were estimated using exchange-based clinopyroxene-liquid barometers following guidelines for clinopyroxene-liquid equilibrium and selection of calibrations provided by MacDonald et al.^79^, who found that thermobarometers calibrated on isothermal–isobaric experimental datasets^11,30^ yield greater accuracy under low undercooling conditions, whereas those incorporating decompression and undercooling experiments^29^ perform better at higher degrees of undercooling. Low undercooling is typically associated with the formation of crystal cores and sector zoned compositions, while high undercooling occurs during magma decompression and degassing, promoting the crystallization of crystal rims and microlites^79^. Hence, we used the barometer from Putirka et al.^30^ (SEE = ± 1.7 kbar, 5.6 km) coupled with the thermometer from Putirka (2008)^11^ (Eq. 33, SEE = ± 45 °C) for clinopyroxene cores and inner rim compositions, whereas for clinopyroxene outer rims and microlites we used the thermobarometer by Mollo et al. (2018)^29^ (SEE = ± 1.5 kbar, 4.9 km and ± 28 °C). These thermobarometers are calibrated for mafic alkaline magmas. For evolved clinopyroxene core compositions, here defined as cores with Mg#<67, we employed a thermobarometer specific for differentiated alkaline magmas by Masotta et al. (2013)^54^ (Palk2012, SEE = ± 1.15 kbar, ± 3.8 km and Talk2012, SEE = ± 18.2 °C).

We applied an eruption-specific clinopyroxene–melt matching approach, pairing clinopyroxene compositions with melt compositions from the same eruption. We used clinopyroxene and melt compositions from this study (Supplementary Data 2 and 6) and from the literature (Supplementary Data 9 and 10). For Tajogaite 2021, we used 422 clinopyroxene compositions from this study and 904 from the literature^21,23^. Melt compositions for Tajogaite 2021 included groundmass and tephra glass (*n* = 708), microcrystalline matrix (*n* = 176), and melt inclusions (MI, *n* = 72) from the literature^17,21,80,81^. For the 1971 Teneguía eruption, clinopyroxene compositions come exclusively from this study (*n* = 164). Published data from Teneguía 1971^15,44^ were not included as they show systematically lower CaO and higher Na₂O contents compared to our data (Supplementary Fig. 3). Melt compositions comprised matrix glass data from this study (*n* = 40) and one glass composition from the literature^82^. For the El Charco 1712 eruption, we used 213 clinopyroxene compositions from this study. Melt compositions included matrix glass (*n* = 40) from this study and one published glass analysis^82^.

Evolved clinopyroxene core compositions (Mg#<67) were paired with one titanite-hosted, tephriphonolite melt pocket composition found in a plagioclase antecryst from the 2021 Tajogaite eruption^18^. To increase the number of equilibrium pairs for evolved clinopyroxene cores, we performed a Monte Carlo simulation, varying the starting melt pocket composition by ±5%, generating 1000 compositions to capture analytical variability. Equilibrium between clinopyroxene and putative melts was evaluated by selecting clinopyroxene–melt pairs that met four criteria based on the agreement between measured and predicted components: DiHd (diopside + hedenbergite) within ±10%, EnFs (enstatite + ferrosilite) within ±5%, CaTs (calcium–tschermak component) within 6%, and CaTi (calcium–titanium component) within ±2%^79^. Melt H_2_O concentration was set at 1 wt% for the tephrite-basanite magma, as suggested for the 2021 Tajogaite primitive magma^41^, and to 3 wt% for evolved core compositions^19^. However, we note that H_2_O has little effect on thermobarometry calculations^19,21^. We also tested other clinopyroxene-liquid exchange-based calibrations, which show little difference in pressure and temperature results (Supplementary Figs. 13 and 14). All calculations were performed with the Thermobar Python tool^83^ and the scripts are included in the Zenodo repository (https://doi.org/10.5281/zenodo.21081067).

Given the challenge in identifying equilibrium liquids for some textural positions of clinopyroxene crystals (e.g., mineral cores), we additionally estimated crystallization pressures and temperatures using the recent machine-learning (ML) clinopyroxene-only thermobarometer from Ágreda-López et al.^33^, which showed improved outcomes compared to previous ML thermobarometers and is expected to perform well for sector zoned clinopyroxene crystallizing from mafic alkaline magmas^79^. This method predicts pressure and temperature directly from clinopyroxene composition using a machine-learning algorithm trained on experimental datasets; the reported value for each input is the median of a 1000-iteration Monte Carlo simulation, with error bars given by the 16th–84th percentile range of that distribution^33^.

The ML-based clinopyroxene-only thermobarometer offers two main methodological advantages over exchange-based clinopyroxene-liquid thermobarometers. First, it does not require an equilibrium liquid composition, avoiding the uncertainty that would otherwise be introduced by assuming one for textural domains where it is not directly constrained (e.g., clinopyroxene cores). Second, exchange-based thermobarometers require pressure and temperature to be solved iteratively from one another, so uncertainty in one estimate propagates into the other. The ML approach avoids this circularity, as pressure and temperature are estimated independently from clinopyroxene composition alone.

## Supplementary information

Supplementary Information

Supplementary Data 1–11

Transparent Peer Review file

## Data Availability

All data included in this article are included in the Supplementary Data in the online version of the article. The Supplementary includes a PDF file with Supplementary Information and Figures. Python scripts used for cluster analysis, map visualization, and thermobarometric calculations, along with all data generated in this study have been deposited in the GitHub repository (https://github.com/AlbCaracc/Caracciolo_et_al_2026_CumbreVieja) and archived through Zenodo at (https://doi.org/10.5281/zenodo.21081067^73^). Samples from the 2021 Tajogaite, 1971 Teneguía and 1712 El Charco eruptions are curated at Litoteca de Petrología at UCM and are available for sharing and collaboration upon request to Dr. A. Márquez.
