# Supplementary material for: Recurrent evacuation of mantle mush in ocean islands revealed by clinopyroxene from La Palma
Source: Nat Commun. 2026 Sep 24;17:9362. doi: 10.1038/s41467-026-77213-9 (PMC13612566; doi:10.1038/s41467-026-77213-9)
Supplement: Supplementary file 1 [file 41467_2026_77213_MOESM1_ESM.pdf]

Recurrent evacuation of mantle mush in ocean islands revealed by clinopyroxene from La Palma

Alberto Caracciolo, Teresa Ubide, Mónica Ágreda-López, Raquel Herrera, Alvaro Marquez, Diego González-García, Maria Jose Huertas, Eumenio Ancochea, Nicolás Chicharro, Juan Jesús Coello-Bravo, Maurizio Petrelli

Supplementary Information and Figures

Corresponding author: Alberto Caracciolo, (alberto@hi.is)

Table of Contents

1. Geological setting 3

2. Clinopyroxene major elements 5

Supplementary Figure 1. Classification of Cpx 5

Supplementary Figure 2. Cpx – melt equilibrium... 6

Supplementary Figure 3. Cpx major element. 8

Supplementary Figure 4. Tephrite and Basanite-hosted Cpx. 8

3. Clinopyroxene trace elements 9

Supplementary Figure 5. Cpx compatible TE composition (NI, Sc, V). 9

Supplementary Figure 6. Cpx incompatible TE composition (Sr, Y, Nb). 10

Supplementary Figure 7. Cpx incompatible TE composition (La, Ce, Nd). 11

Supplementary Figure 8. Cpx core REE patterns. 11

Supplementary Figure 9. Rock-grouped cpx REE patterns. 12

Supplementary Figure 10. Texture-grouped cpx REE patterns 13

Supplementary Figure 11. Cr chemical maps 14

4. Geochemistry of the matrix...15

Supplementary Figure 12. TAS diagram 15

5. Thermobarometry 16

Supplementary Figure 13. Comparison of P results... 17

Supplementary Figure 14. Comparison of T results. 19

Supplementary Figure 15. Comparison of ML- and exchange-based temperatures..19

6. Clustering results 20

Supplementary Figure 16. Cluster-colored cpx REE patterns..................................20

Supplementary Figure 17. Evolved cpx REE patterns...21

Supplementary Figure 18. Cluster-colored cpx composition, P and T 21

7. Equilibrium melt composition and trace elements modelling 22

Supplementary Table 1. 22

Supplementary Figure 19. Equilibrium melt composition and FC model............24

Supplementary Figure 20. TAS FC model. 25

Supplementary Figure 21. Mg–Fe^{2+}(+Mn)–Na Cpx classification 26

8. Compilation of the OIB database and PCA 27

Supplementary Table 2. 28

9. Comparison of electron microprobe instruments..................29

Supplementary Figure 22. EMPA Comparison El Charco 1712...................29

Supplementary Figure 23. EMPA Comparison Tajogaite 2021....................30

10. Clustering methods...............31

Supplementary Figure 24. Quantitative methods for selection of k......................32

Supplementary Figure 25. Clustering data imbalance. 33

11. Individual cpx maps 33

Supplementary Figure 26. LA-ICP-MS chemical maps.....................................33

12. Supplementary References...49

1. Geological setting

The Canary Islands are an example of intraplate volcanism, commonly attributed to a hotspot beneath Jurassic oceanic lithosphere[1], although alternative plate-related models have also been proposed[2]. La Palma is one of the westernmost and youngest islands of the archipelago. It is composed of the older, now extinct Garafía and Taburiente shield volcanoes in the north, and the younger, historically active Cumbre Vieja ridge in the south (Fig. 1). La Palma is the most volcanically active island in the Canaries during historical times, having experienced eight eruptions since the late 15th century, including the most recent Tajogaite eruption in 2021[3,4]. Historical eruptions occurred in 1470, 1585, 1677-78, 1712, 1949, 1971 and 2021, with return periods varying between 22 and 237 years[5]. Eruptions at Cumbre Vieja are typically strombolian, producing highly alkaline magma series, ranging from basanitic to tephritic lavas[6] although more evolved phonolites are occasionally erupted [7]. In this work, we focus on the 1712 El Charco, 1971 Teneguía and 2021 Tajogaite eruptions (Fig. 1), for which we have well characterized samples across the tephrite and basanite stages.

The 1712 El Charco eruption is the least documented eruption of the Canaries and the available information is described by Romero Ruiz (1990) [8]. The 1712 eruption occurred in the central west part of Cumbre Vieja and began on October 9, with the opening of a main cone and multiple secondary vents along a 3 km-long fracture, trending SE–NW. Although there was some phreatomagmatic activity, the eruption was essentially strombolian, producing lava flows approximately 5 km in length that reached the sea, forming several lava deltas[8]. The eruption lasted for 56 days, from October 9 to December 3, 1712 [8]. This event produced tephrite to basanite lavas [9], with an estimated total lava volume of 36 x 10^6 m^3 [5]. Samples for El Charco 1712 were collected from different parts of the lava field, and amphibole-bearing tephrites are consistently found at the bottom of the olivine-bearing basanites (Supplementary Data 1).

The 1971 Teneguía eruption took place at the southern tip of La Palma, near the site of the 1677–1678 eruption. Activity began on October 26 with the opening of an eruptive fissure that produced pyroclastic material and lava flows, which rapidly reached the coast. Several vents were active during the eruption. The eruption continued for 24 days, ending on November 18, 1971. This event produced tephrite to basanite lavas and the total eruption volume is estimated to be 40 x 10^6 m^3 [5,10]. Samples from the 1971 Teneguía eruption were collected during the eruption, allowing the construction of a time series of samples (Supplementary Data 1).

The Tajogaite 2021 eruption began on 19 September 2021, at Cabeza de Vaca locality, in the middle part of Cumbre Vieja ridge. The eruption began with Strombolian activity and lava fountaining, transitioning to more intense lava and pyroclastic emissions from multiple vents in October–November 2021. Early tephritic magmas shifted to basanitic compositions over the first few days, with the eruption ending on 13 December

2021. The minimum erupted volume is estimated at 170 ± 85 x10^6 m^3 [11]. For this most recent eruption, modern geophysical and petrological monitoring provide a more detailed eruption evolution. The first signs of volcanic unrest preceding the 2021 Tajogaite eruption occurred in 2017, interpreted as related to magmatic intrusions [12,13]. After eight days of seismic swarms, the eruption began on 19 September 2021, producing tephritic magma rich in amphibole and clinopyroxene until the first break on 27 September [14,15]. Following the few-hours long break, the eruption continued emitting more fluid basanite magmas, dominated by olivine and clinopyroxene crystals, until the end of the eruption, on 13 December 2021 [15,16]. Temporal changes are observed during the 2021 Tajogaite eruption, the most significant being a shift towards more mafic melts over the course of the eruption, followed by a reversal in that trend since the end of November to the end of the eruption, as observed in clinopyroxene, plagioclase and olivine rims, microlites, matrix and whole rock compositions [15–19]. The eruption is overall interpreted as a result of a mafic basanite magma gradually invading a tephrite reservoir [15,16], with the basanite sourced from the upper mantle and interacting with a tephrite reservoir located in the upper mantle [15,19] or in the middle to lower crust [20,21]. Samples from Tajogaite 2021 eruption were collected both during and after the eruption and correspond to the same sample set published in Ubide et al. (2023)[15] (Supplementary Data 1).

2. Clinopyroxene major elements

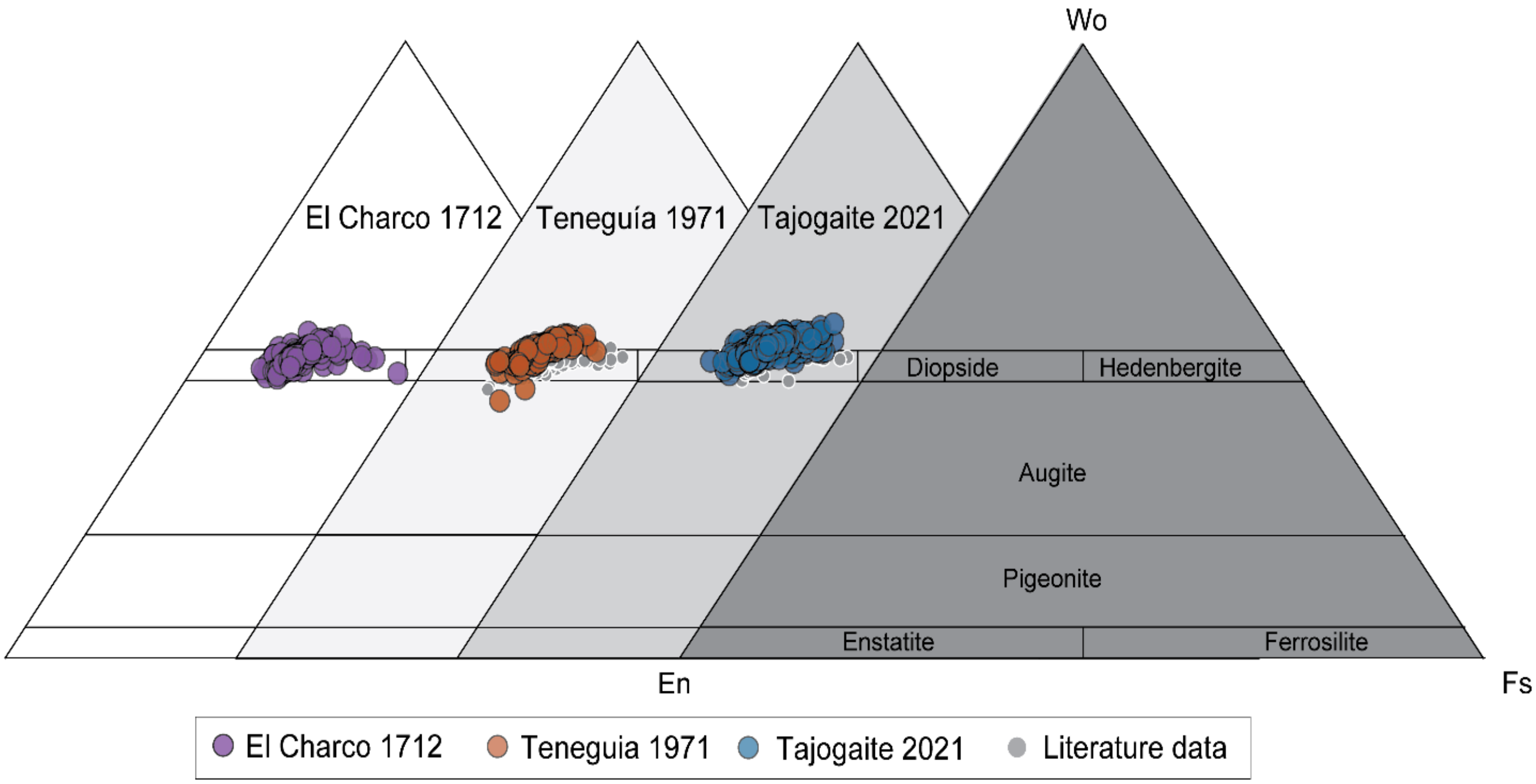

Supplementary Figure 1*. Classification of Cpx. Composition of clinopyroxene crystals in the En-Fs-Wo ternary diagram. All compositions fall within the diopside field, with no differences between eruptions. Gray symbols represent clinopyroxene data from the literature* [15,22–24].

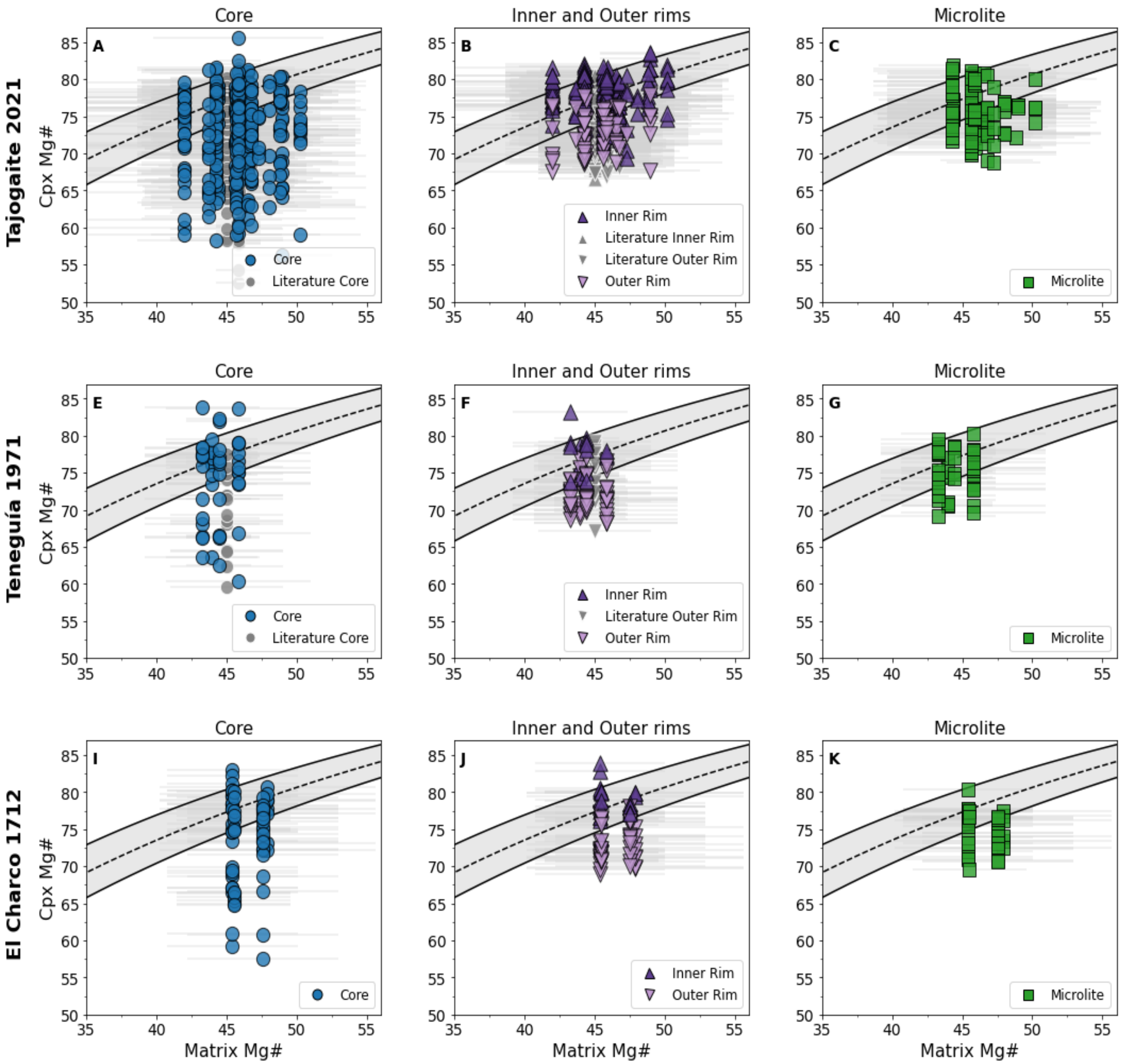

***Supplementary Figure 2**. Cpx – melt equilibrium. Matrix Mg# vs clinopyroxene Mg# for (A-C) Tajogaite 2021 (E-G) Teneguía 1971 and (I-K) El Charco 1712. Each symbol and color represent a different textural position (Core, Inner and Outer rims, Microlites, and Cpx inclusions), as indicated in the legend. Gray symbols correspond to literature data [22–24]. Inner rim and microlite compositions from the 2021 Tajogaite eruption are from Ubide et al. (2023)[15]. The gray field in each panel indicates the $KD(Fe–Mg)_{cpx–melt}$ of 0.28 ± 0.08[25], which is used to assess major element equilibrium between clinopyroxene and the matrix composition. Horizontal error bars reflect the matrix Mg# 2σ variability.*

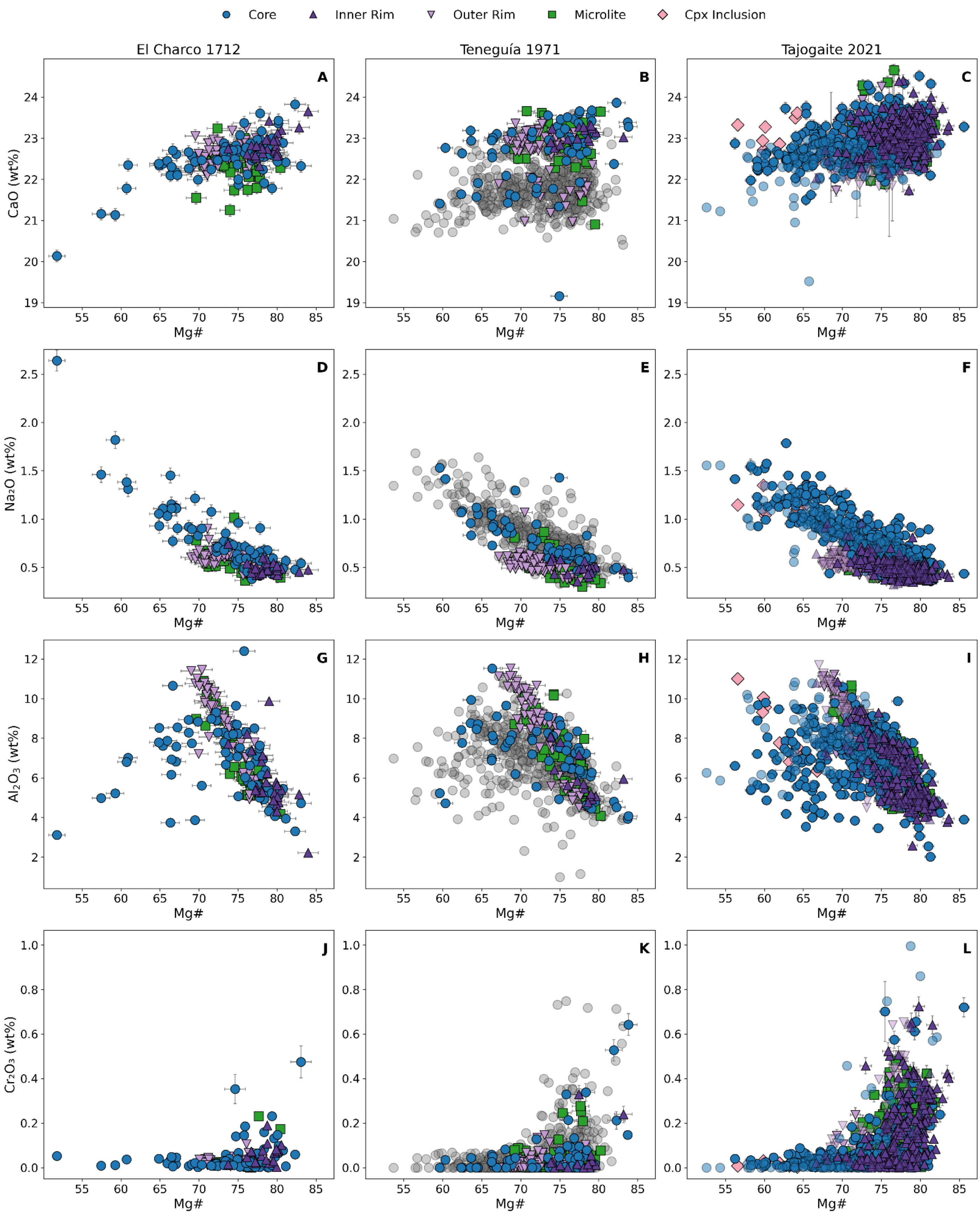

Core
Inner Rim
Outer Rim
Microlite
Cpx Inclusion
El Charco 1712
Teneguía 1971
Tajogaite 2021
CaO (wt%)
Na₂O (wt%)
Al₂O₃ (wt%)
Cr₂O₃ (wt%)
Mg#
A
B
C
D
E
F
G
H
I
J
K
L

Supplementary Figure 3. *Cpx major element. CaO, Na_2O, Al_2O_3 and Cr_2O_3 variation in clinopyroxene from the different eruptions studied in this work, with symbols and colors based on different textural positions. Literature data for Teneguía 1971[22,24] are not grouped into textures and are plotted as filled faded grey circles. For Tajogaite 2021, literature data [23] are shown with faded colours according to textural positions. We observe an offset in CaO contents between our data and previously published data from Teneguía, and a minor offset also in Na_2O and Al_2O_3. We consider our measurements of good quality, as they closely match concentrations in clinopyroxenes from other eruptions, which were analyzed using various microprobes. The data quality is further supported by our cross-validation across instruments (Supplementary Figure 1 and 2). In contrast, the literature data from Teneguía[22,24] were acquired using the same instrument (JEOL JXA-8530F at Uppsala University), and the observed discrepancy may reflect a calibration issue specific to CaO and on that instrument. Error bars indicate 2σ uncertainties derived from EMPA counting statistics.*

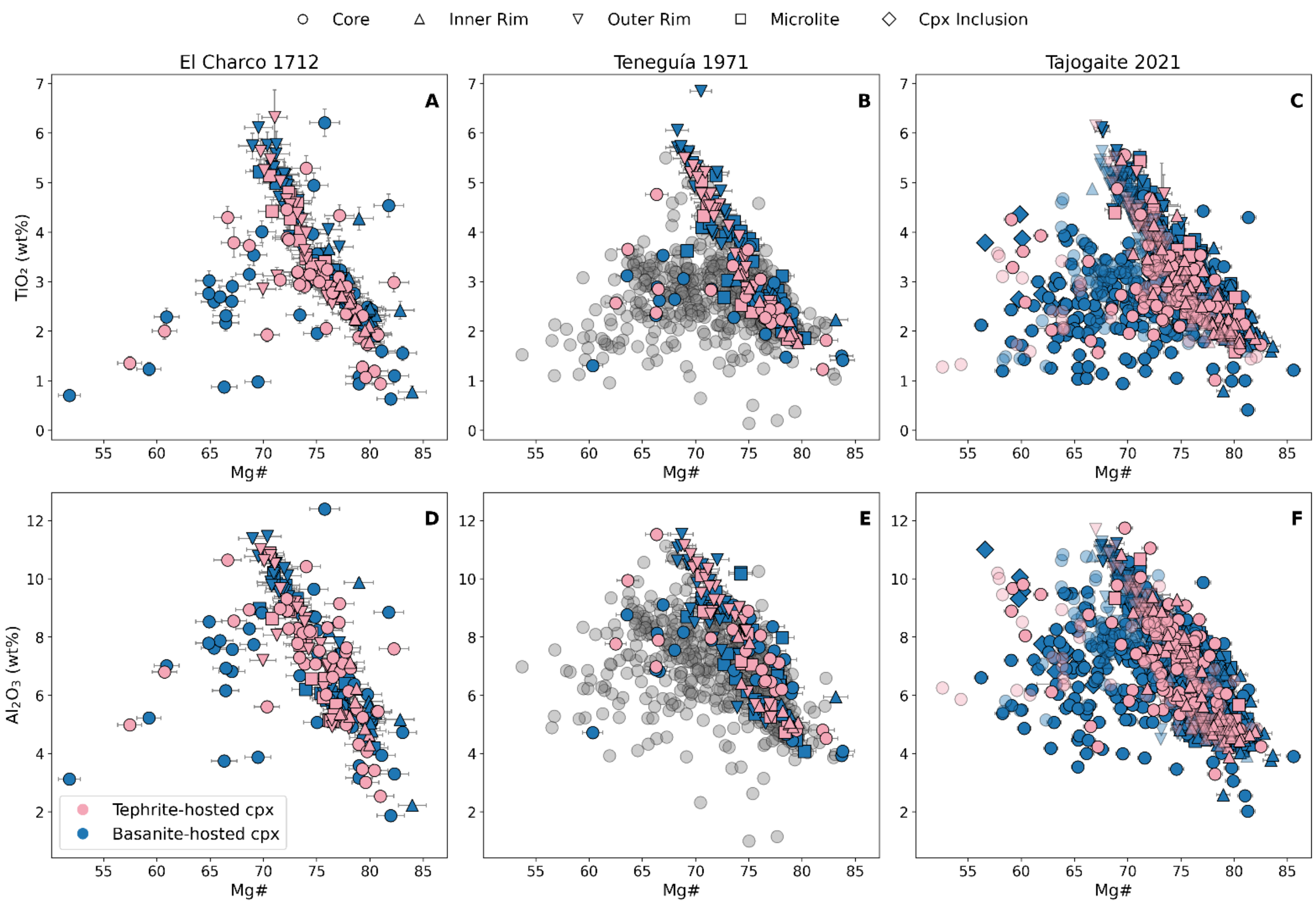

Supplementary Figure 4. *Tephrite and Basanite-hosted Cpx. (A–C) TiO_2 and (D–F) Al_2O_3 variations as a function of Mg# in clinopyroxene, color-coded based on whether they occur in amphibole-bearing tephrite (pink) or olivine-bearing basanite (blue) rocks. No clear relationship is observed between clinopyroxene composition and rock type. Literature data for Teneguía 1971 [22,24] are plotted as faded filled grey circles. For Tajogaite 2021, literature data[23] are shown with faded colors. Error bars indicate 2σ uncertainties derived from EMPA counting statistics.*

3. Clinopyroxene trace elements

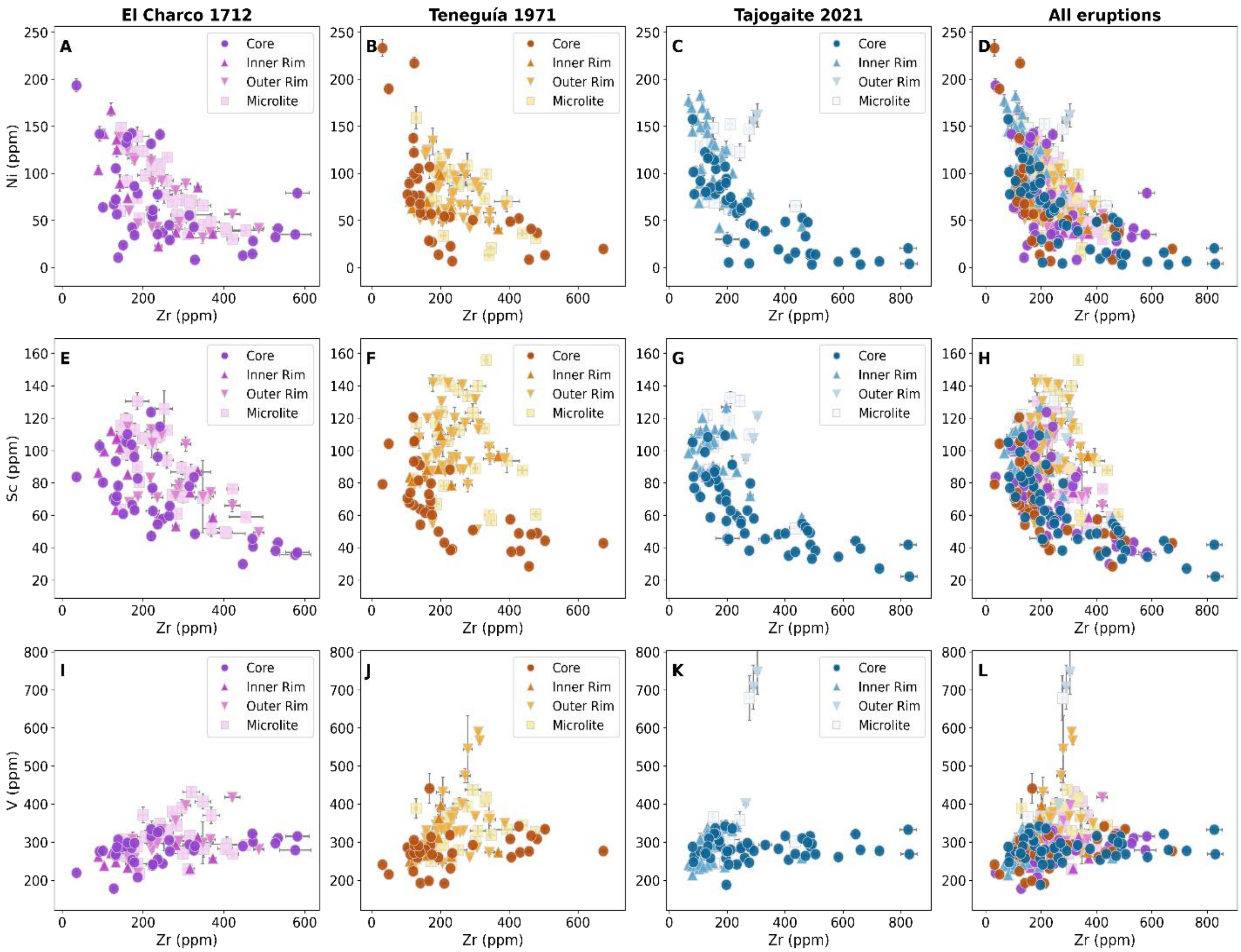

***Supplementary Figure 5**. Cpx compatible TE composition (NI, Sc, V). Clinopyroxene trace element bivariant plots of (A-D) Ni (E-H) Sc and (I-L) V vs Zr. Each column corresponds to a different eruption, as indicated by the column titles, with the final column showing data from all eruptions combined. Each symbol represents a different clinopyroxene textural position. Error bars indicate the internal 2SE analytical uncertainty.*

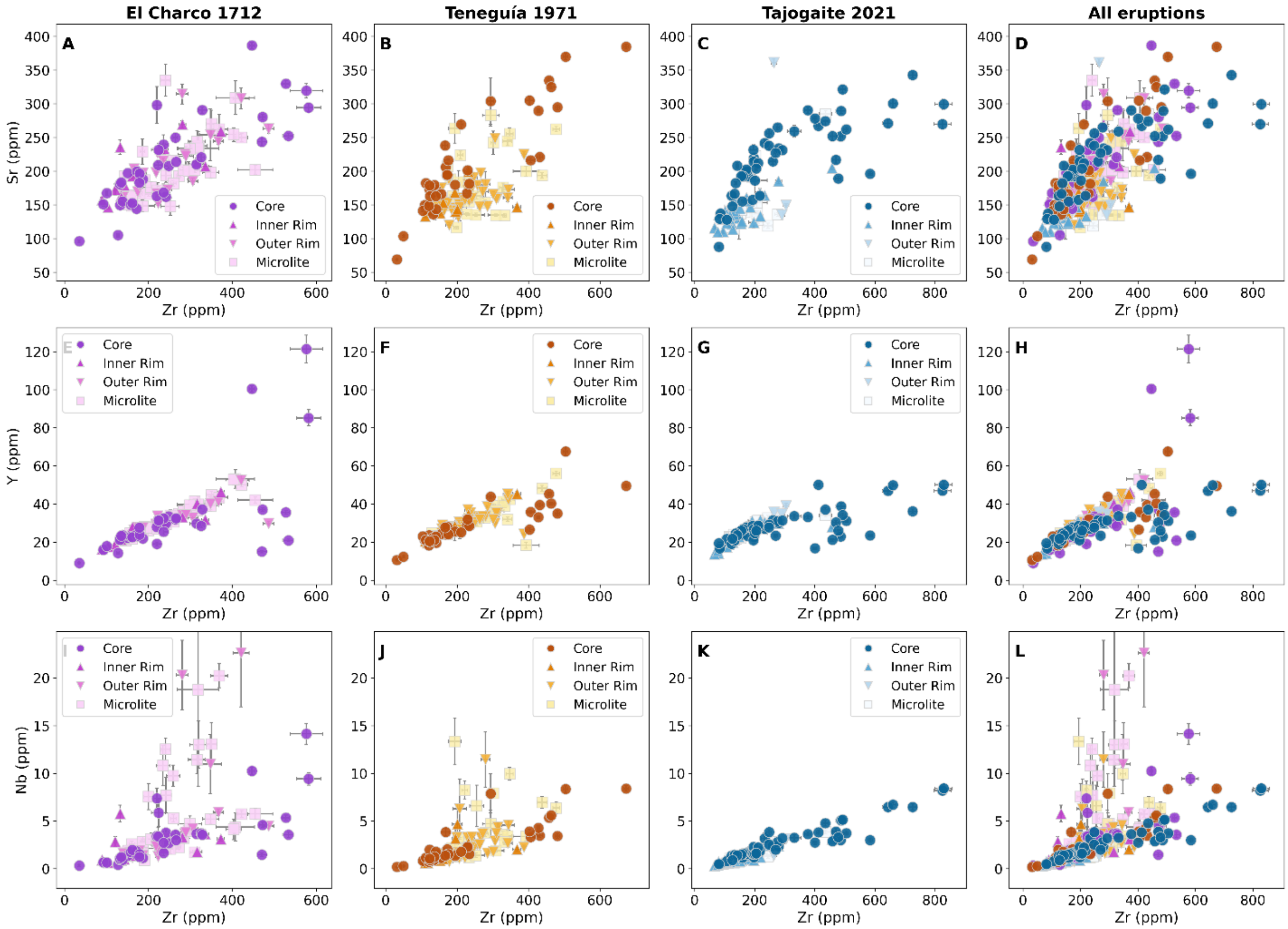

Supplementary Figure 6*. Cpx incompatible TE composition (Sr, Y, Nb). Clinopyroxene trace element bivariant plots of (A-D) Sr (E-H) Y and (I-L) Nb vs Zr. Each column corresponds to a different eruption, as indicated by the column titles, with the final column showing data from all eruptions combined. Each symbol represents a different clinopyroxene textural position. Error bars indicate the internal 2SE analytical uncertainty.*

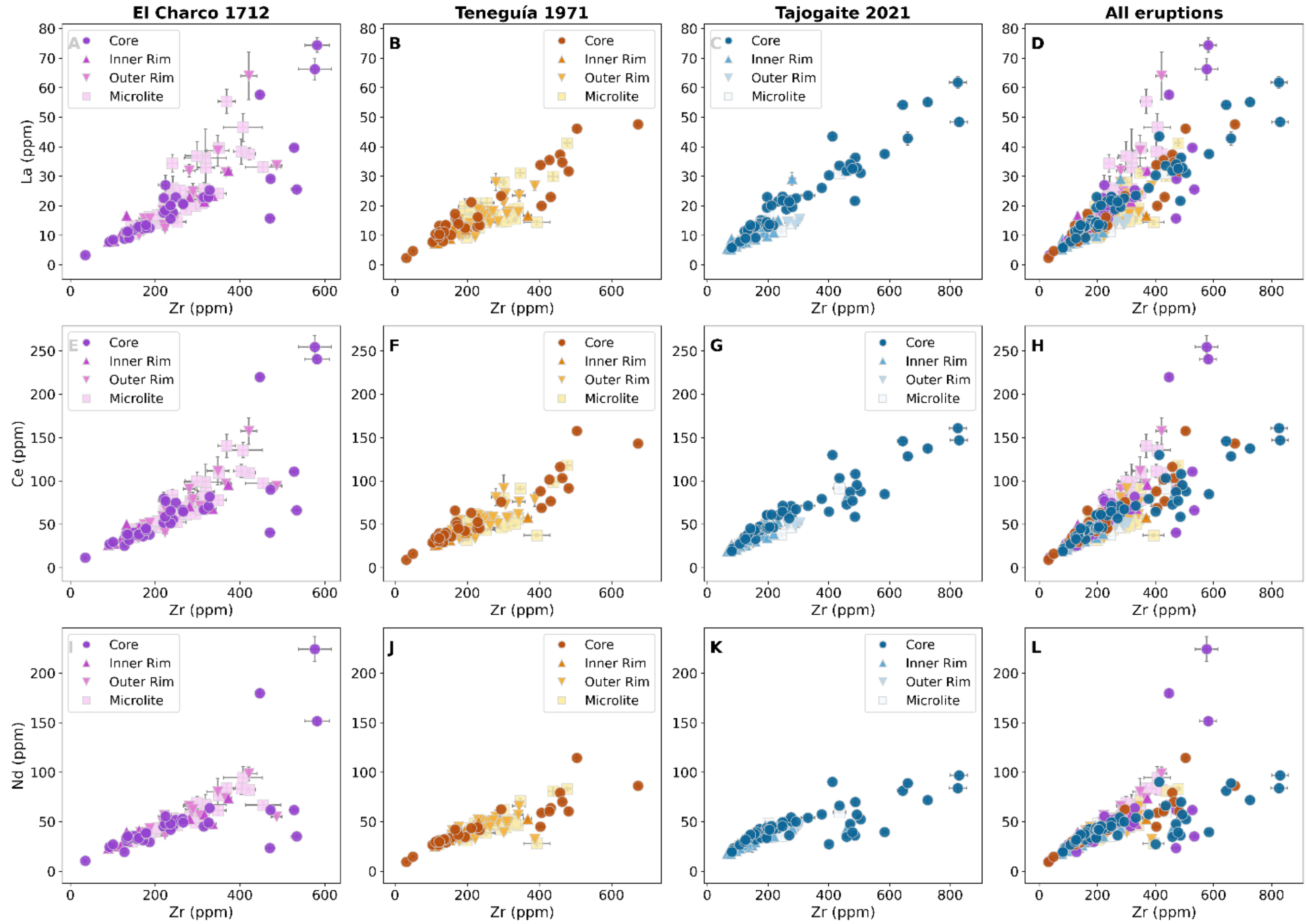

Supplementary Figure 7. *Cpx incompatible TE composition (La, Ce, Nd). Clinopyroxene trace element bivariant plots of (A-D) La (E-H) Ce and (I-L) Nd vs Zr. Each column corresponds to a different eruption, as indicated by the column titles, with the final column showing data from all eruptions combined. Each symbol represents a different clinopyroxene textural position. Error bars indicate the internal 2SE analytical uncertainty.*

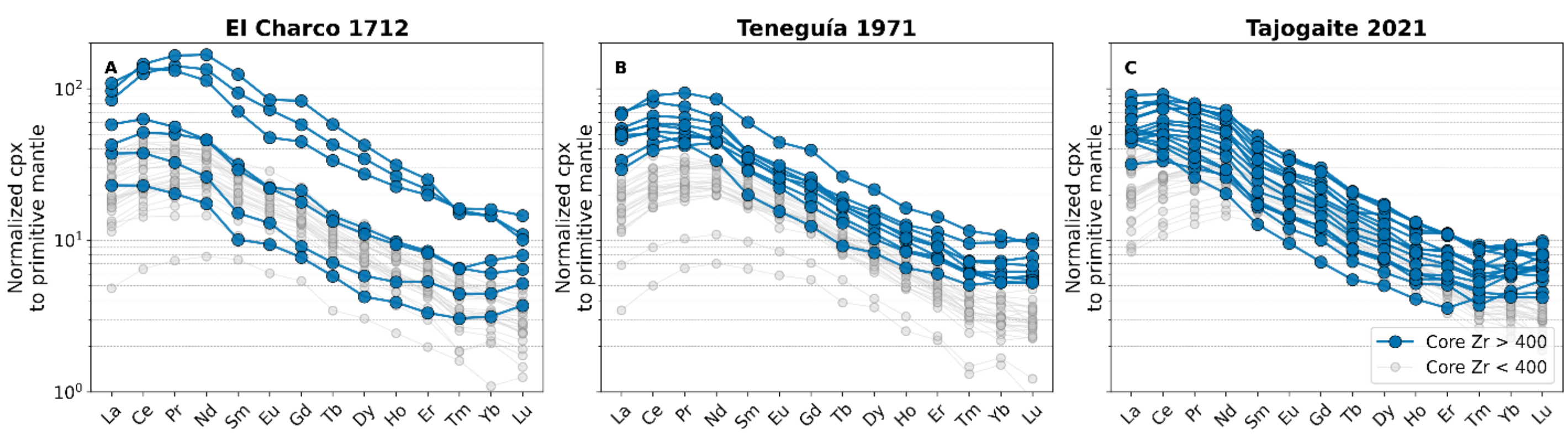

Supplementary Figure 8*. Cpx core REE patterns. Trace element pattern of clinopyroxene cores, color-coded based on Zr concentrations, used as a proxy for magma evolution. Evolved core compositions (Zr>400 ppm), marked in blue, are the most fractionated and show a slight enrichment in heavy REE elements Tm, Yb and Lu compared to less evolved core compositions (Zr<400 ppm).*

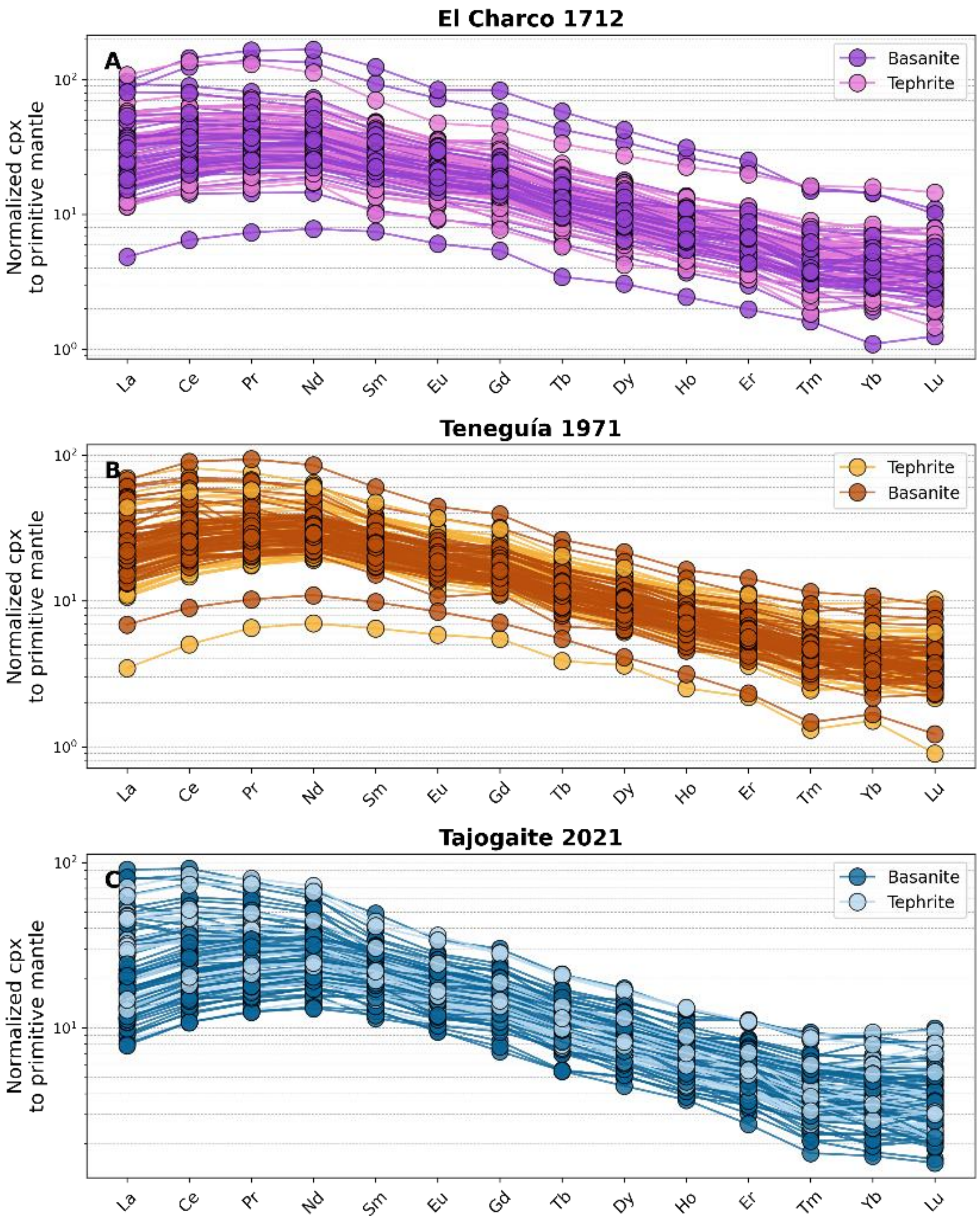

***Supplementary Figure 9**. Rock-grouped cpx REE patterns. Clinopyroxene trace element patterns colored by host rock type, tephrite and basanite.*

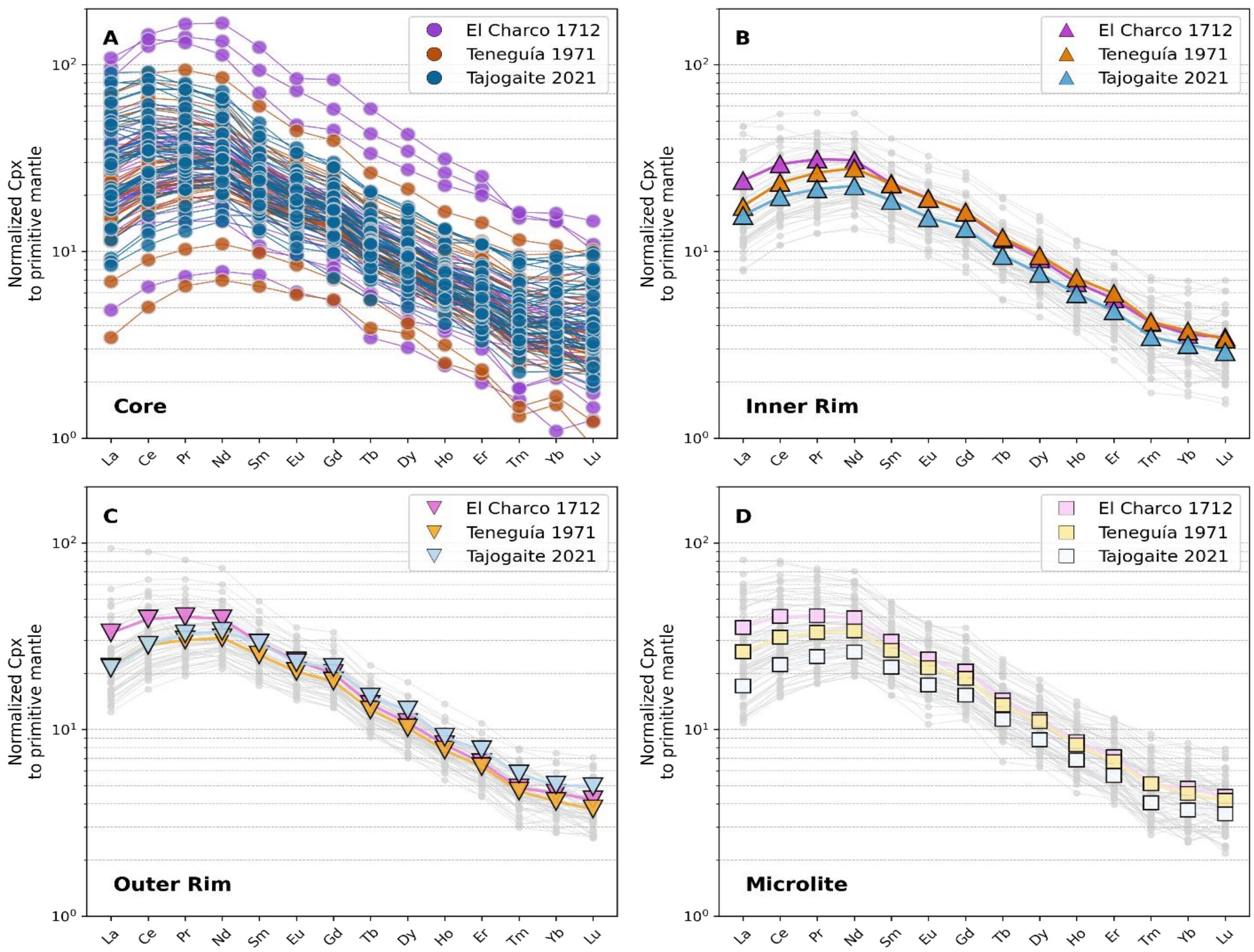

Supplementary Figure 10. *Texture-grouped cpx REE patterns. Rare earth element (REE) patterns of clinopyroxene, normalized to the composition of primitive mantle[26]. REE patterns of cores are shown as individual data, whereas for inner rims, outer rims, and microlites we show the average for each eruption, with individual patterns displayed in light grey.*

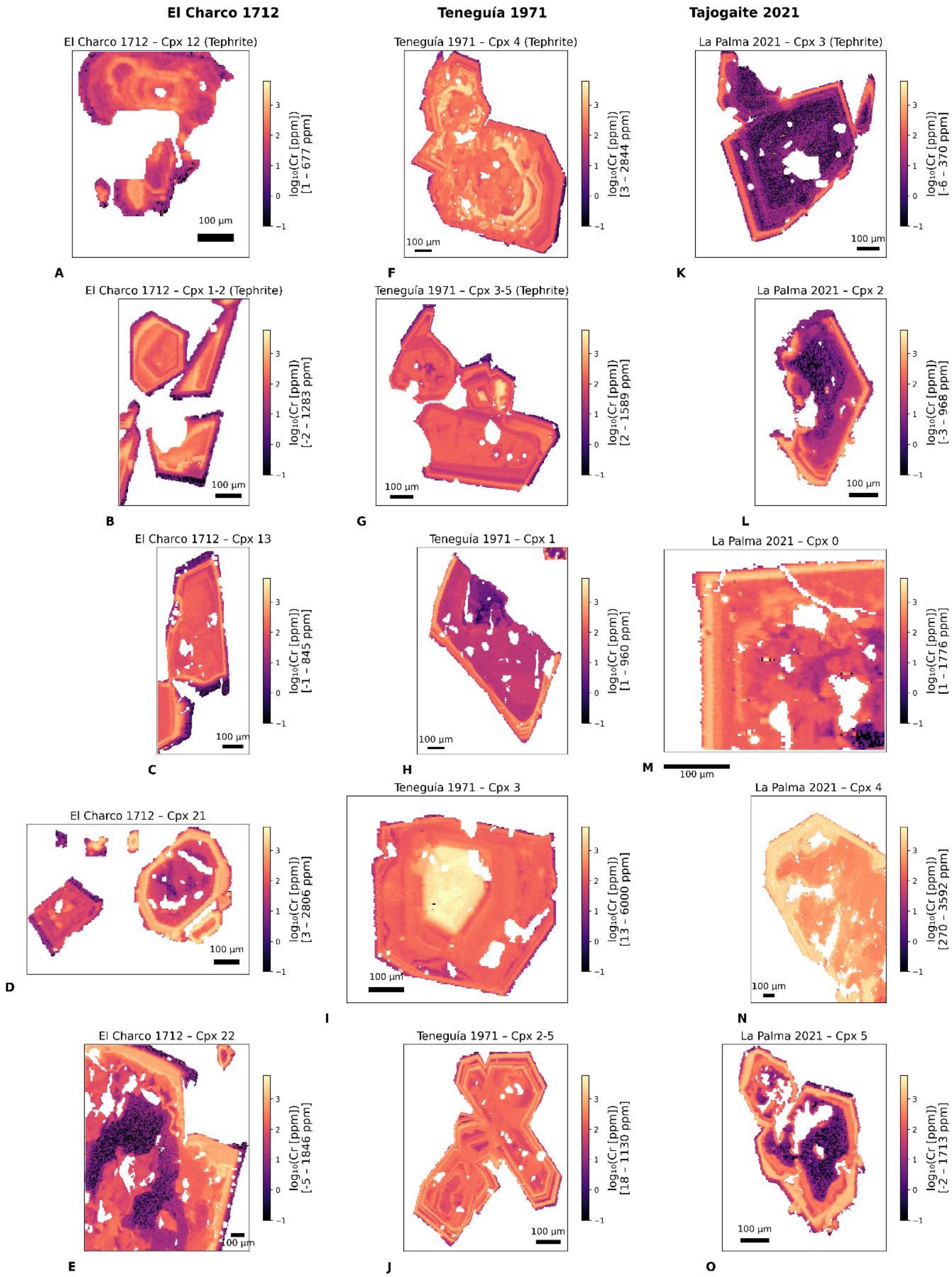

Supplementary Figure 11*. Cr chemical maps. LA-ICP-MS maps of Cr in clinopyroxene from El Charco 1712, Teneguía 1971 and Tajogaite 2021. Maps are visualized using a logarithmic scale and scaled to the maximum common range observed across all maps (0.1–6000 ppm Cr; log_{10} = -1–3.8) to allow direct comparison of Cr variations among samples and eruptions.*

4. Geochemistry of the matrix

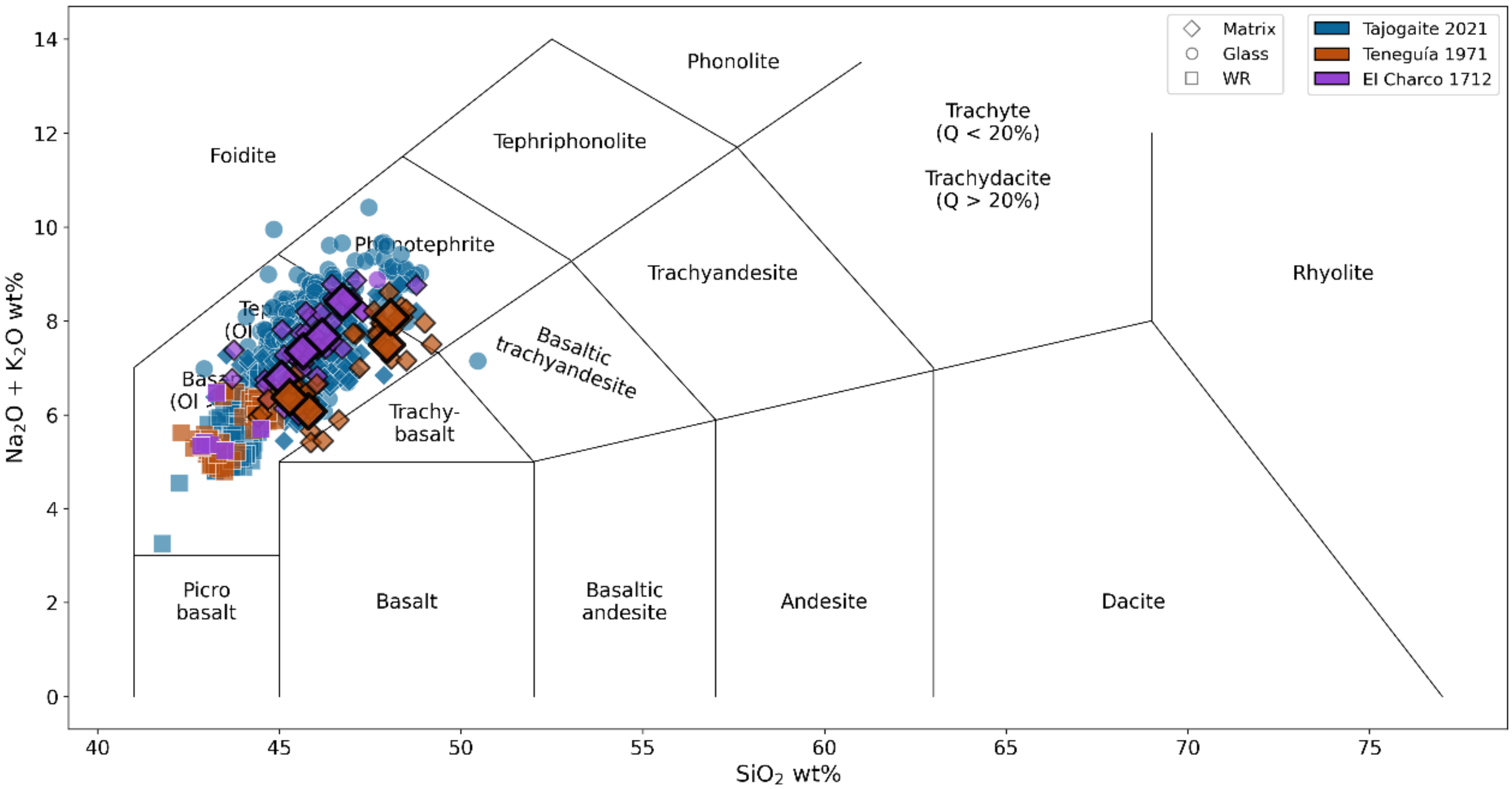

Supplementary Figure 12. *TAS diagram. TAS diagram showing the matrix compositions studied in this work, compared with literature data. Large diamonds for Teneguía 1971 and El Charco 1712 represent the average matrix compositions based on 10 analyses from this study. Matrix data for Tajogaite 2021 Ubide et al. (2023)[15]. Whole-rock (WR) data for Tajogaite 2021 [14–16]. WR data for El Charco 1712 [27–30] and Teneguía 1971 [22,24,27,30–32] were retrieved from the GEOROC database.*

5. Thermobarometry

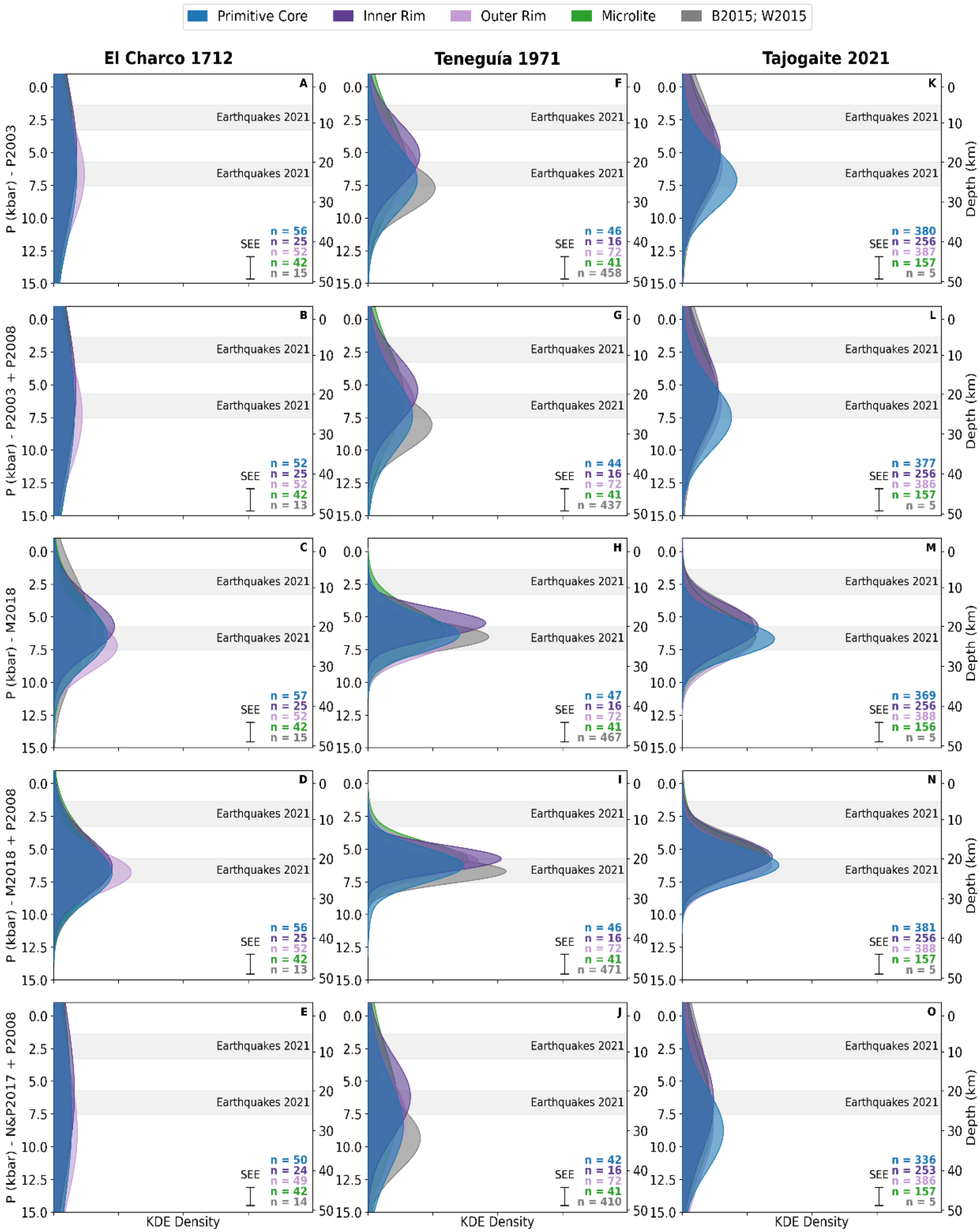

Supplementary Figure 13. *Comparison of P results. Comparison of clinopyroxene – melt magma storage depths using different temperature-dependent exchange-based barometers coupled with different thermometers. For this comparison we used more relaxed equilibrium criteria. These required differences between measured and predicted clinopyroxene components to be within 12% for DiHd, 10% for EnFs, and 6% for CaTs, within 2σ of the thresholds proposed by Neave et al. (2019)[33], and a ($K_D(Fe–Mg)_{cpx–melt}$) of 0.28 ± 0.08 [25]. P2003: barometer and thermometer of Putirka et al. (2003)[34]. P2003 + P2008: barometer of Putirka et al. (2003)[34], coupled with the thermometer (eq. 33) of Putirka (2008)[25]. M2018: barometer and thermometer of Mollo et al. (2018)[35]. M2018 + P2008: barometer of Mollo et al. (2018)[35], coupled with thermometer (eq. 33) of Putirka (2008)[25]. N&P2017 + P2008: barometer of Neave & Putirka (2017)[36] coupled with thermometer (eq. 33) of Putirka (2008)[25]. Gray fields indicate the location of seismicity during the 2021 Tajogaite eruption [37]. B2015: Barker et al. (2015)[22]. W2015: Weis et al. (2015)[24].*

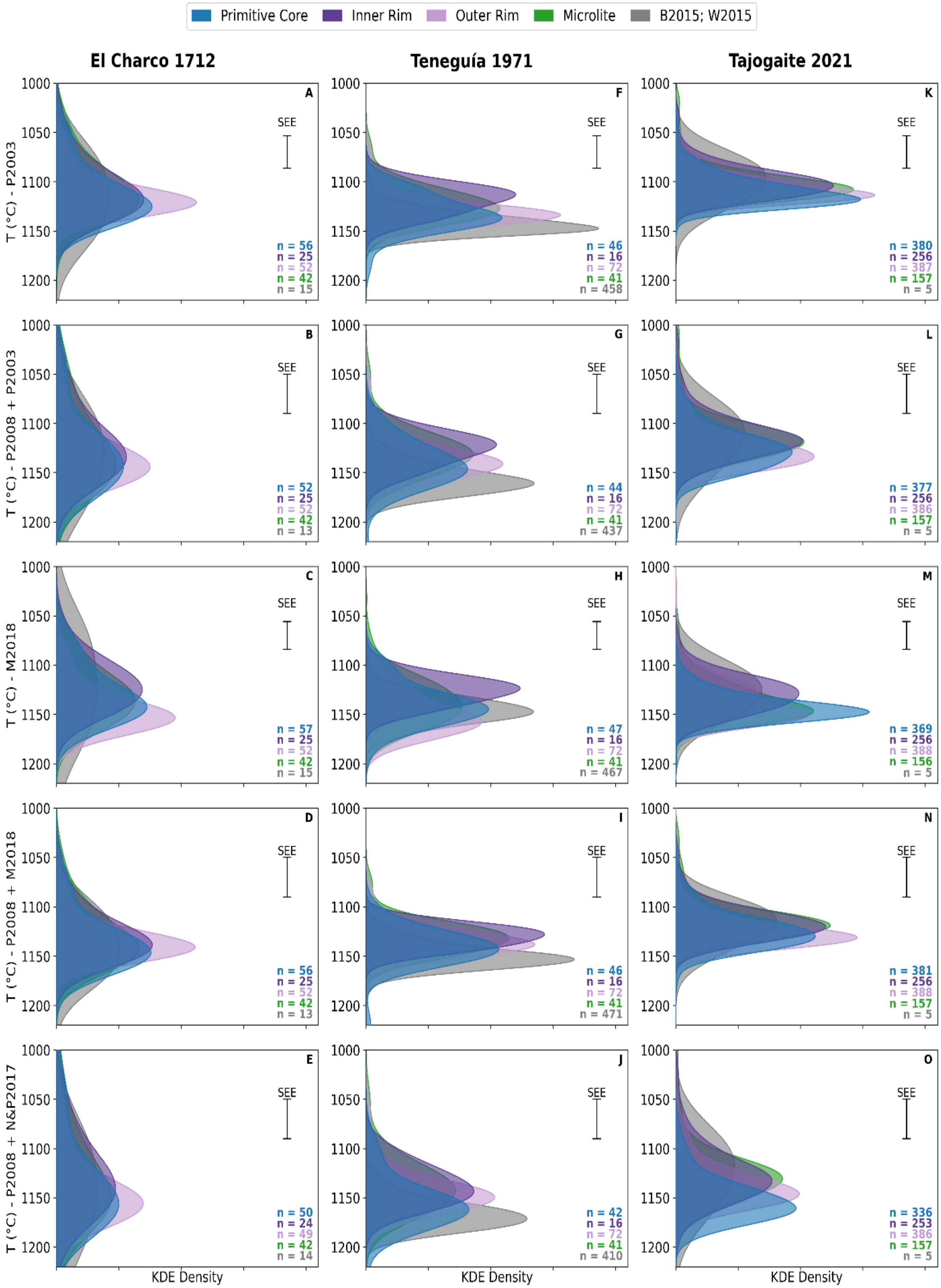
Primitive Core
Inner Rim
Outer Rim
Microlite
B2015; W2015
El Charco 1712
Teneguía 1971
Tajogaite 2021
T (°C) - P2003
T (°C) - P2008 + P2003
T (°C) - M2018
T (°C) - P2008 + M2018
T (°C) - P2008 + N&P2017
SEE
KDE Density
A
n = 56
n = 25
n = 52
n = 42
n = 15
F
n = 46
n = 16
n = 72
n = 41
n = 458
K
n = 380
n = 256
n = 387
n = 157
n = 5
B
n = 52
n = 25
n = 52
n = 42
n = 13
G
n = 44
n = 16
n = 72
n = 41
n = 437
L
n = 377
n = 256
n = 386
n = 157
n = 5
C
n = 57
n = 25
n = 52
n = 42
n = 15
H
n = 47
n = 16
n = 72
n = 41
n = 467
M
n = 369
n = 256
n = 388
n = 156
n = 5
D
n = 56
n = 25
n = 52
n = 42
n = 13
I
n = 46
n = 16
n = 72
n = 41
n = 471
N
n = 381
n = 256
n = 388
n = 157
n = 5
E
n = 50
n = 24
n = 49
n = 42
n = 14
J
n = 42
n = 16
n = 72
n = 41
n = 410
O
n = 336
n = 253
n = 386
n = 157
n = 5

Supplementary Figure 14*. Comparison of T results. Comparison of clinopyroxene – melt magma storage temperatures using different exchange-based pressure-dependent thermometers coupled with different barometers. P2003: thermometer of Putirka et al. (2003) [34], coupled with the barometer of Putirka et al. (2003) [34]. P2003 + P2008: thermometer of Putirka et al. (2003) [34], coupled with the barometer (eq. 33) of Putirka (2008) [25]. M2018: thermometer of Mollo et al. (2018) [35], coupled with barometer of Mollo et al. (2018) [35]. M2018 + P2008: thermometer of Mollo et al. (2018) [35], coupled with barometer (eq. 33) of Putirka (2008) [25]. N&P2017 + P2008: thermometer of Neave & Putirka (2017) [36] coupled with barometer (eq. 33) of Putirka (2008) [25]. B2015: Barker et al. (2015) [22]. W2015: Weis et al. (2015) [24].*

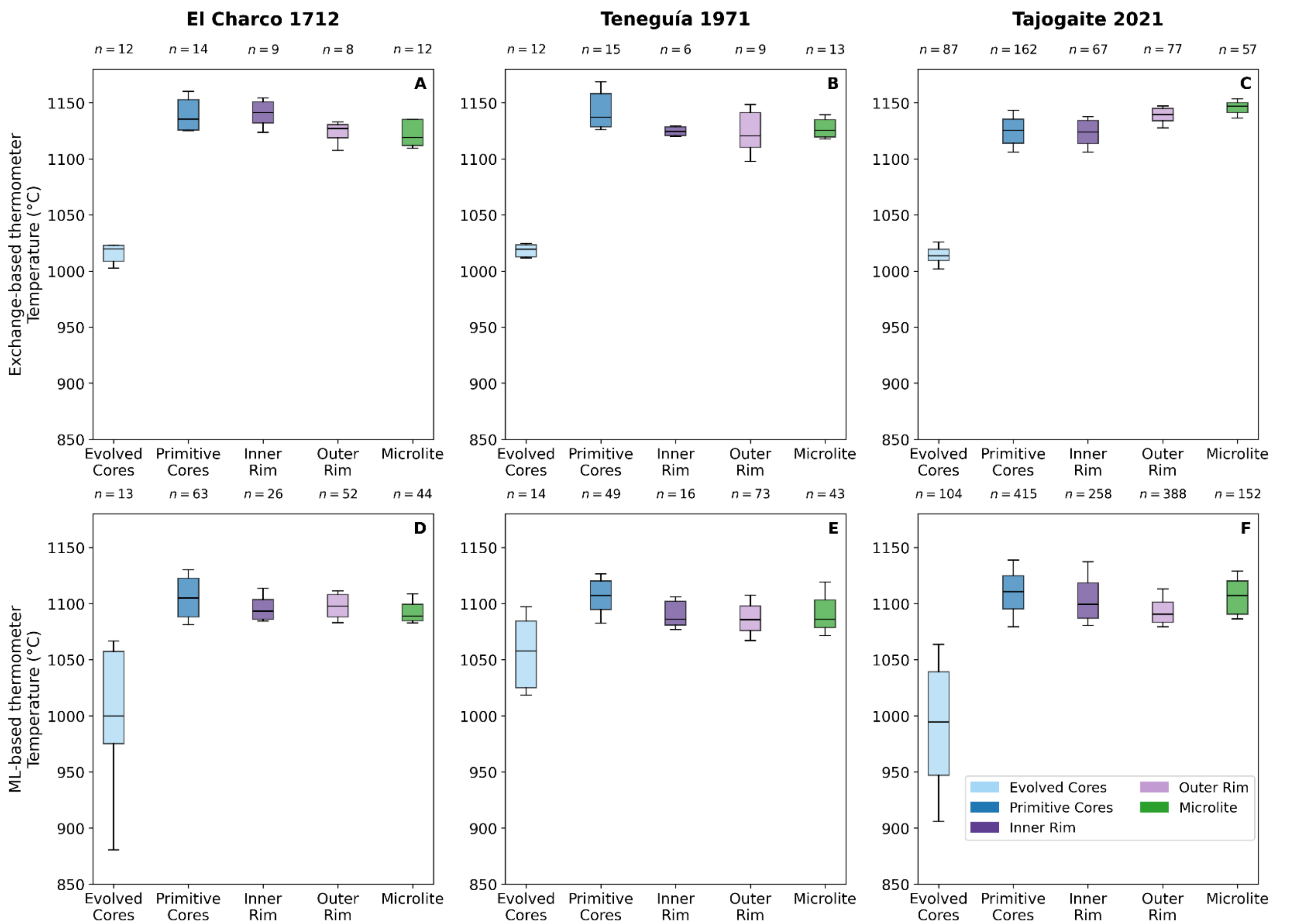

Supplementary Figure 15*. Comparison of ML- and exchange-based temperatures. (A-C) Temperature results from exchange-based thermometers, following the approach described in the main text. (D-F) Temperature results using ML-based thermometer[38]. Both methods yield similar temperatures and, importantly, consistently indicate lower temperatures for evolved cores. The box represents the interquartile range (25th to 75th percentile), the horizontal line indicates the median, and the whiskers extend to the to the 10th and 90th percentiles*

6. Clustering results

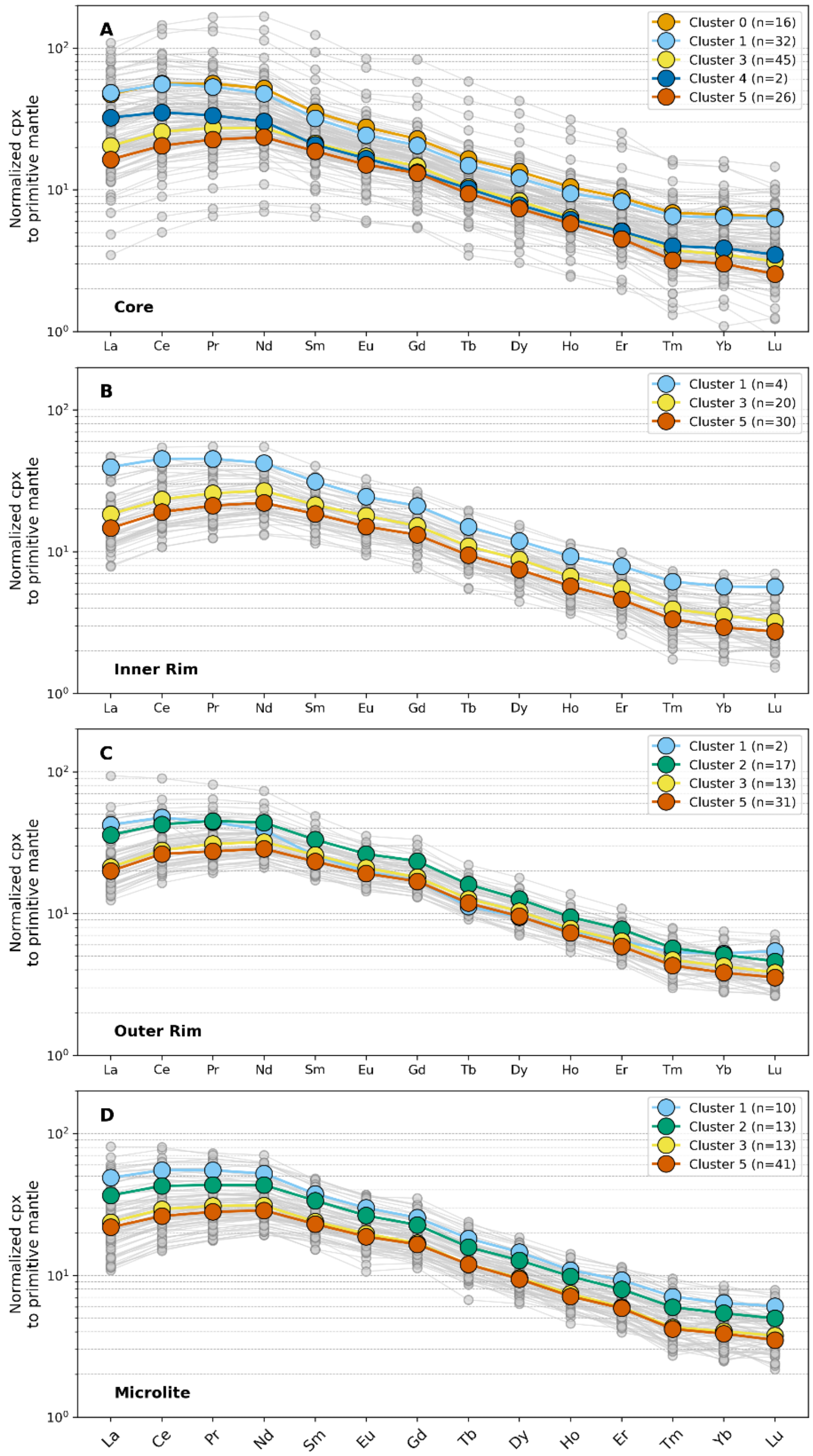

Supplementary Figure 16. *Cluster-colored cpx REE patterns. Trace element patterns are color-coded based on clusters. Grey lines and points represent individual measurements, while colored lines show the average for each cluster group. Each panel corresponds to a specific textural position: (A) core (B) inner rim (C) outer rim and (D) microlite.*

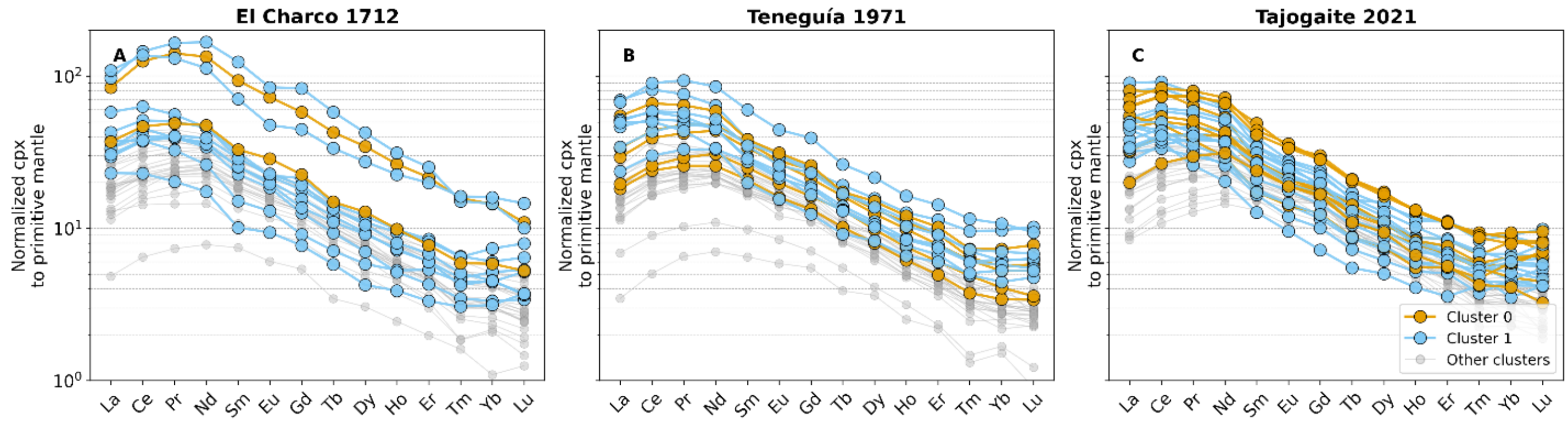

Supplementary Figure 17*. Evolved cpx REE patterns. Trace element pattern of evolved clinopyroxene cores belonging to Cluster 0 and 1. These compositions are the most differentiated, with patterns showing an enrichment in heavy REE elements Tm, Yb and Lu, typical of phonolite-derived compositions. Grey lines represent patterns of clinopyroxenes belonging to Cluster 2, 3, 4 and 5.*

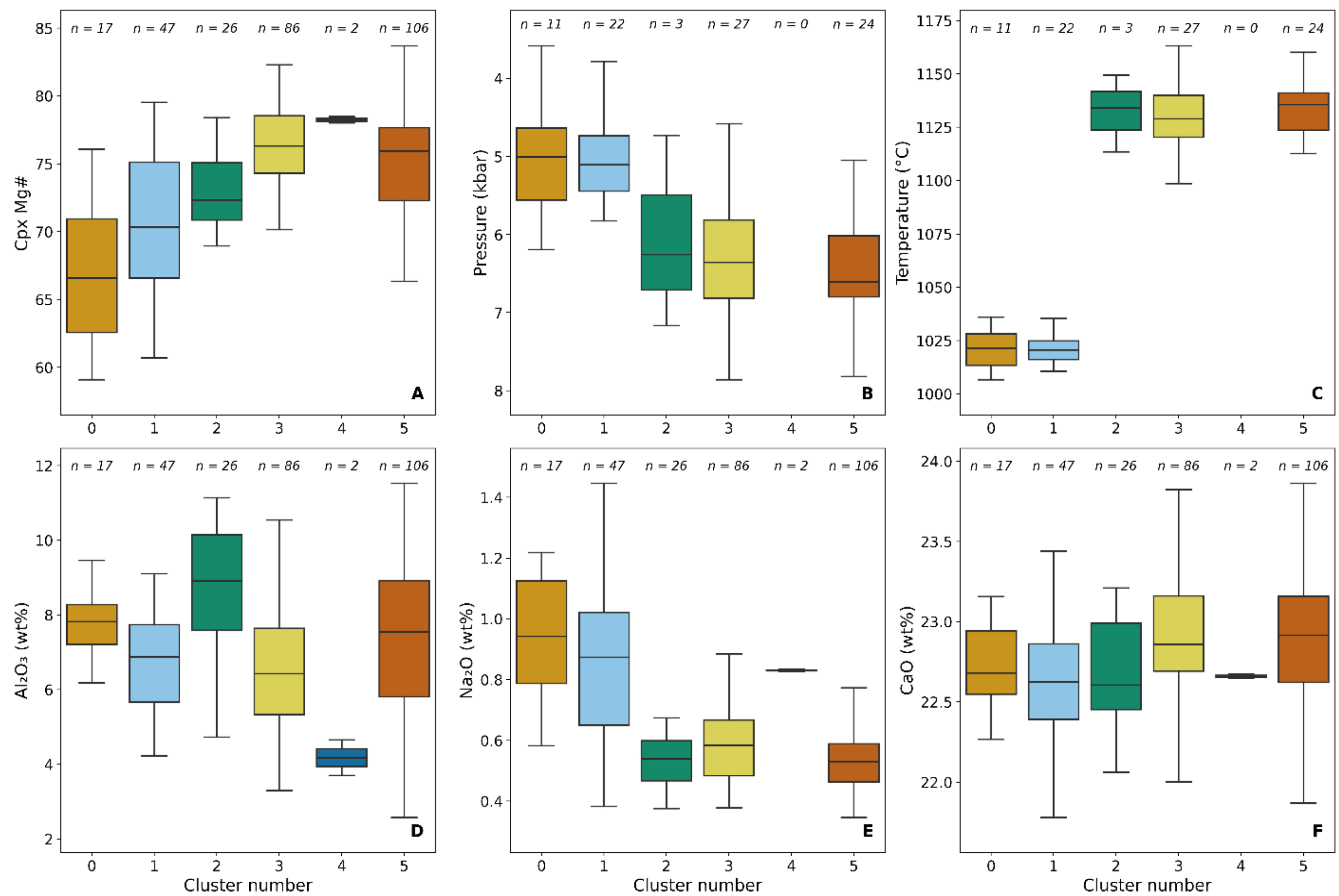

Supplementary Figure 18*. Cluster-colored cpx composition, P and T. Box plots show the distribution of clinopyroxene (A) Mg# for each cluster. Panels (B) and (C) show exchange-based clinopyroxene–melt pressures and temperatures, respectively, for each cluster. Only data that passed the equilibrium criteria following the approach of MacDonald et al. (2023)[39] are included, as described in the method section. (D-F) Distribution of Al_2O_3, Na_2O and CaO for each cluster. In this figure, only analyses with both EMPA and LA-ICP-MS single-spot data are plotted.*

7. Equilibrium melt composition and trace elements modelling

We reconstructed the trace element melt composition in equilibrium with each measured clinopyroxene composition to compare these with the trace element composition of the matrix, interpreted here as representative of the carrier melt, and to model fractional crystallization (FC).

Equilibrium melt compositions were calculated using partition coefficients (KDs) derived from multiple parametrizations published in Bédard (2014). Specifically, for each element, we applied several parameterizations calibrated for terrestrial systems (TE), mafic terrestrial systems (MTS), and low-pressure MTS as reported in Bédard (2014). The resulting KDs were averaged, and uncertainties were estimated based on the 1σ variability across parameterizations. Using these fixed KDs for each trace element, we reconstructed the equilibrium melt composition for each clinopyroxene, incorporating the uncertainty from the different parameterizations. Overall, the calculated KDs are of the same order of magnitude as those reported for alkali basalts in the GERM database.

Major-element fractional crystallization was modeled starting from a primitive matrix composition with the following oxide concentrations (wt%): SiO_2 = 45.21, TiO_2 = 3.64, Al_2O_3 = 14.29, FeOt = 12.05, MnO = 0.22, MgO = 6.82, CaO = 10.22, Na_2O = 4.53, K_2O = 1.86, and P_2O_5 = 1.17. We used the web app of MAGEMin, a Gibbs free-energy minimization solver that computes thermodynamically stable phase assemblages for a given thermodinamic database, melt composition, pressure and temperature conditions [40]. Fractional crystallization of major elements was modeled using the Igneous thermodynamic database [41,42], assuming a constant pressure of 5 kbar, consistent with our clinopyroxene–melt barometry results. The crystallization path was simulated between 1250 °C and 900 °C. The model provides the evolution of major element compositions, as well as the stable mineral phase assemblage and corresponding phase proportions at each step of crystallization in the investigated temperature range. The fractionating phases, together with their respective temperatures of first appearance and the corresponding remaining melt fraction are summarized in Table 1.

**Mineral Phase**	**T (°C)**	**F**	**Kd_{La}**	**Kd_{Zr}**	**Kd_{Yb}**	**Kd_{Y}**
Spinel	1234	0.985	0.002	0.008	0.010	0.002
Olivine	1218	0.964	0.0001	0.005	0.050	0.001
Ilmenite	1187	0.901	0.000003	0.29	0.17	0.0045
Clinopyroxene	1107	0.737	0.19	0.15	0.75	0.65
Nepheline	1028	0.490	0.01	0.005	0.01	0.01
Plagioclase	1012	0.388	0.20	0.10	0.006	0.020

Supplementary Table 1. *Summary of crystallizing phases, their temperatures of first appearance along the liquidus, and the corresponding remaining melt fraction (F), based on the MAGEMin model results. For each phase we indicate the KD used in the modelling, taken from the GERM database.*

Trace element modeling was subsequently performed using the phase proportions obtained from the MAGEMin major element output. At each fractionation step, the bulk partition coefficient (D_{bulk}) was calculated from the proportions of crystallizing phases according to:

$$D_{\text{bulk}} = \sum_{i} X_i \, D_i$$

where X_i is the mass fraction of phase *i* crystallizing at a given step, and D_i is the mineral–melt partition coefficient for that phase.

Trace element concentrations in the residual melt were then calculated using the Rayleigh fractional crystallization equation:

$$C_l = C_0 \, F^{(D_{\text{bulk}}-1)}$$

where C_l is the trace element concentration in the melt after fractionation, C_0 is the initial concentration, F is the remaining melt fraction, and D_{bulk} is the bulk partition coefficient at that step. Because phase proportions vary during crystallization, D_{bulk} was recalculated at each incremental step along the modeled crystallization path.

The starting trace element composition of the matrix was obtained by averaging the least differentiated matrix compositions with Zr concentrations between 300–330 ppm. The starting composition has the following trace element concentrations (in ppm): La = 90, Zr = 320, Yb = 2.38, and Y = 33.

For clinopyroxenes, we used a fixed Kd for each trace element, calculated as the median of individual values estimated from the Bédard (2014) parameterizations described above. For the other mineral phases, Kd values were taken from the GERM database. Specifically, Kds for olivine are from Adam and Green (2006); for plagioclase, from Schnetzler and Philpotts (1970) and Villemant et al. (1981); for spinels, from Elkins et al. (2008) and McKenzie and O'nions (1991); for ilmenite, from Nielsen et al. (1992) and Zack and Brumm (1998); and for nepheline, from Larsen (1979).

Our modeling can broadly reproduce both the major element trends (Fig. 6) and the trace element variations observed in the matrix (Supplementary Figure 19). Tephritic compositions for each eruption can be generated by ~10–20% fractional crystallization of a basanitic parental melt from the same eruption.

When considering the reconstructed equilibrium melt compositions (Supplementary Figure 19D-F), the most differentiated melts, represented by Clusters 0 and 1, can be reproduced by ~80–90% fractional crystallization of a basanitic melt. At this stage of evolution, the modeled melt composition reaches tephriphonolitic to phonolitic compositions, as indicated by the MAGEMin major element model in the TAS diagram (Supplementary Figure 20). According to the model, tephri-phonolitic and phonolitic melts are generated between ~900 and 1000 °C, consistent with our exchange-based and ML-based thermometry results, which constrain the crystallization temperatures of evolved clinopyroxene cores to ~1000–1020 °C. These correspondences reinforce the

interpretation that Clusters 0 and 1 clinopyroxene core compositions are associated with evolved tephri-phonolitic melts stored at depth.

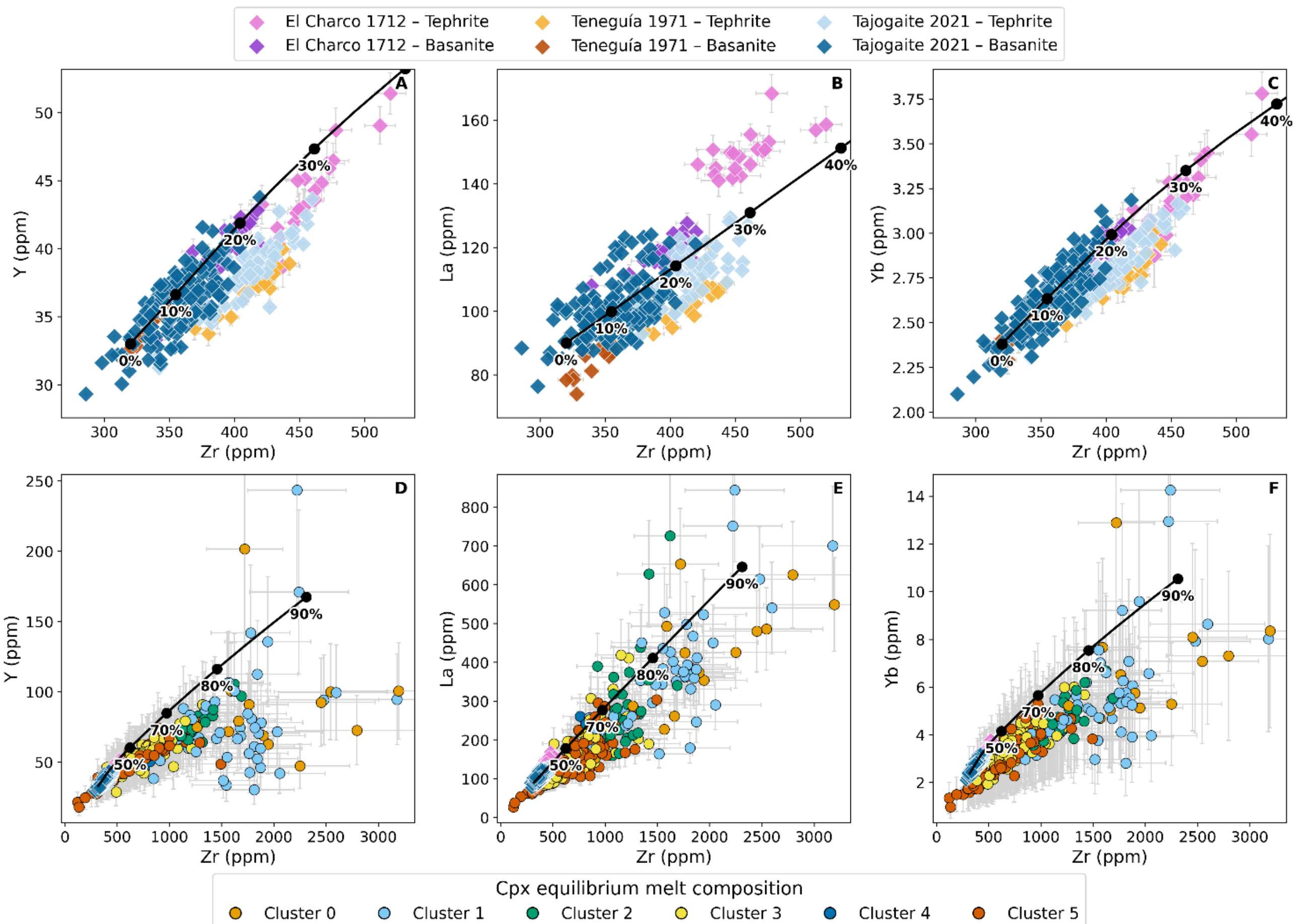

Supplementary Figure 19. *Equilibrium melt composition and FC model. (A–C) Trace element composition of the matrix and FC results from the thermodynamic-based MAGEMin model. Each black filled circle represents 10% increments of fractionation starting from 100% melt phase. Note that the tephrite compositions for each eruption can be reproduced by ~10-20% fractionation from a basanite composition of the same eruption. (D–F) Reconstructed clinopyroxene equilibrium melt compositions, colored according to clustering. The black line represents the thermodynamic-based MAGEMin FC model. Note that Clusters 0 and 1 compositions are consistent with 80–90% fractionation.*

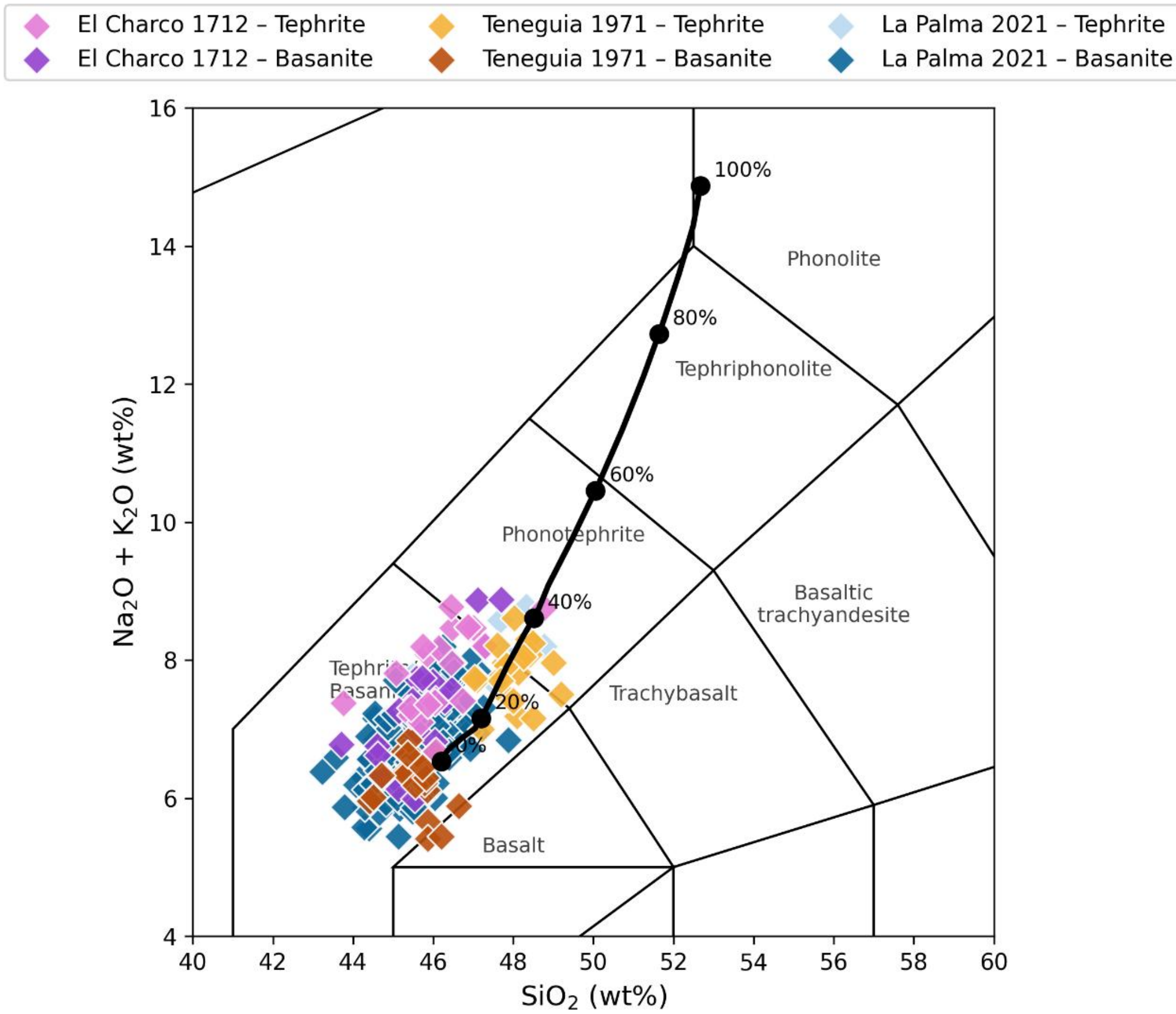

Supplementary Figure 20. *TAS FC model. Total alkali–silica (TAS) diagram showing matrix glass compositions together with the MAGEMin fractional crystallization model. The model indicates that tephriphonolite to phonolite compositions are generated after ~ 80–90% fractional crystallization of an initial basanite composition.*

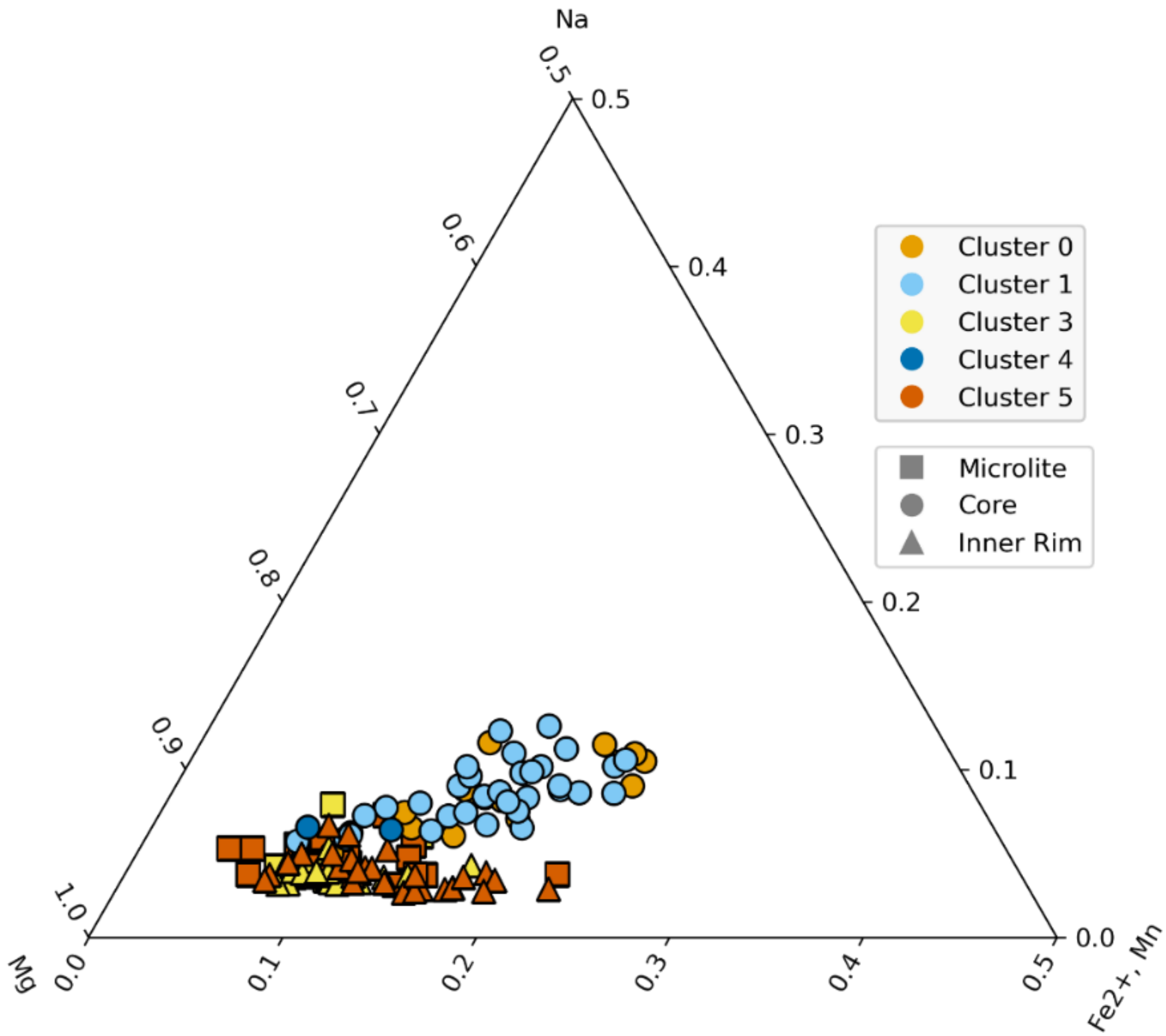

Supplementary Figure 21. *Mg–Fe²⁺(+Mn)–Na Cpx classification. Clinopyroxene compositions are plotted on the Mg–Fe²⁺(+Mn)–Na atoms per formula unit diagram after Larsen (1976). We show Cluster 0 and 1 core compositions, Cluster 4 core compositions (n=2), and Cluster 3 and 5 inner rim and microlite compositions to compare evolutionary trends of the phonolite-derived (Clusters 0 and 1) and tephrite–basanite (Clusters 3 and 5) recharge compositions relative to Cluster 4. Notably, Clusters 0 and 1 cores define a distinct lineage from the Cluster 3 and 5 recharge compositions, while Cluster 4 lies at the beginning of the phonolite lineage, potentially representing a composition capable of evolving into phonolitic melts[52]. Cluster 2 compositions are not included in this plot as they are related to magma ascent and surface cooling.*

8. Compilation of the OIB database and PCA

In the discussion, we extend the results of this study to other well-studied ocean island basalt (OIB) systems by linking the occurrence of evolved tephri-phonolite to phonolite mushes to the evolutionary stage of volcanic islands and to magma flux regime. To this end, we compiled published Holocene clinopyroxene major-element data from OIB systems, starting from the GEOROC mineral database [53] and supplementing it with data from recent literature. We filtered the data to include only clinopyroxene compositions with totals between 98 and 102 wt% (Supplementary Data 11).

We focused on datasets from Holocene eruptions in order to focus on the current evolutionary stage of volcanic islands and on localities for which large clinopyroxene datasets are available. Data from seamounts were plotted independently of age, as they represent rocks erupted millions of years ago. Given that our goal is to explore the occurrence of evolved antecrysts in mafic magmas, as identified at Cumbre Vieja in La Palma, we restricted the dataset to clinopyroxene hosted in mafic volcanic lithologies. These include the following rock types: Basanite, Tephrite, Alkali basalt, Basalt, Trachybasalt, Picrite, Picrobasalt, Trachybasalt and Transitional basalt. For comparison, we also plotted clinopyroxene compositions from felsic rocks using a different symbol to illustrate the distribution of these compositions. Felsic rocks were included regardless of age and comprise lithologies of Benmoreite, Ignimbritic tuff, Nephelinite, Phonolite, Phonotephrite, Pumice and Trachyte.

Given the unequal number of analyses among localities (e.g., 2458 datapoints from mafic rocks from La Palma versus 356 from Tenerife and 83 from Mayotte), we applied a Monte Carlo weighted bootstrap resampling approach to reduce sampling bias. Specifically, datasets containing more than 400 analyses were randomly resampled and downsampled to obtain datasets of comparable size across volcanoes, thereby preventing large datasets from disproportionately influencing the statistical distribution.

To reduce the multidimensionality of the dataset and identify the main vectors of compositional variability, we performed principal component analysis (PCA) on the major and minor element concentrations (SiO_2, TiO_2, Al_2O_3, FeO, MgO, CaO, Na_2O, and MnO). Prior to PCA, the variables were standardized to zero mean and unit variance to ensure that all elements contributed equally to the analysis despite differences in absolute concentration ranges. PCA was then applied to the standardized dataset, and the first two principal components (PC1 and PC2), which capture the largest proportion of the total variance (70%) were retained for visualization and interpretation. We then examined the orientation of the geochemical vectors in PC1–PC2 space to identify the main compositional trends in the dataset.

The PCA groups the data along three dominant compositional trends as shown by the direction of the vectors: Mg, Al–Ti and Fe–Na–Mn. These trends are interpreted to reflect basanitic magma recharge (high MgO), post recharge melt evolution during ascent (high TiO_2 and Al_2O_3), and recycliing evolved antecrysts (high FeO, Na_2O, and MnO), consistent with the presence of evolved mushes as observed in our study.

The source of literature clinopyroxene data is reported in Supplementary Data 11 and summarized in Supplementary Table 2 here below:

OIB Stage	Archipelago/chain	Locality	Data source
Seamounts	-	Vesteris seamount	[54]
Seamounts	Magellan seamounts	Pako Guyot	[55]
Seamounts	Magellan seamounts	Govorov Guyot	[56]
Seamounts	Wake seamounts	Lamont Guyot	[57,58]
Seamounts	Louisville seamounts	Burton, Canopus, Rigil, Osbourn	[59–61]

Initial stage	Canary Islands	La Palma	[15,22–24,62–66]
Initial stage	Canary Islands	El Hierro	[67–71]
Initial stage	Azores	Pico	[72,73]
Initial stage	Comoros	Mayotte	[74,75]
Initial stage	Galapagos	Isabela	[76–79]
Initial stage	Cape Verde	Fogo	[80–83]
Mature stage	Canary Islands	Tenerife	[84–90]
Mature stage	Canary Islands	Gran canaria + Lanzarote	[91–97]
Mature stage	Azores	Terceira	[98–101]
Mature stage	Azores	Flores + Sao Miguel	[102–104]

Supplementary Table 2. *OIB cpx data. List of OIB localities included in the Figure 10 compilation and their data sources.*

9. Comparison of electron microprobe instruments

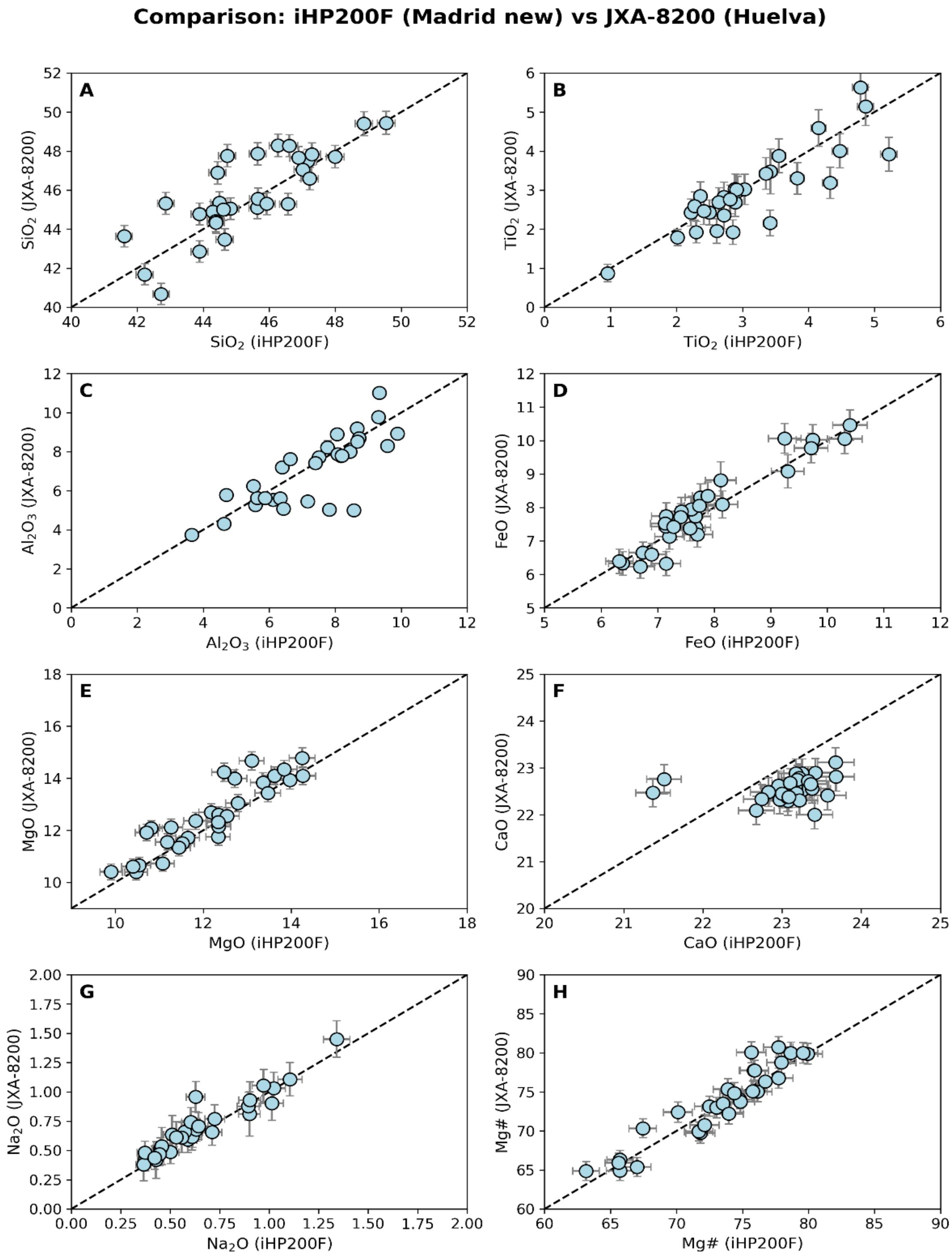

***Supplementary Figure 22**. EMPA Comparison El Charco 1712. Comparison of major and minor element data for samples from El Charco 1712 collected with the new iHP200F microprobe in Madrid and JXA-8200 microprobe in Huelva. Error bars indicate 2σ uncertainties derived from EMPA counting statistics.*

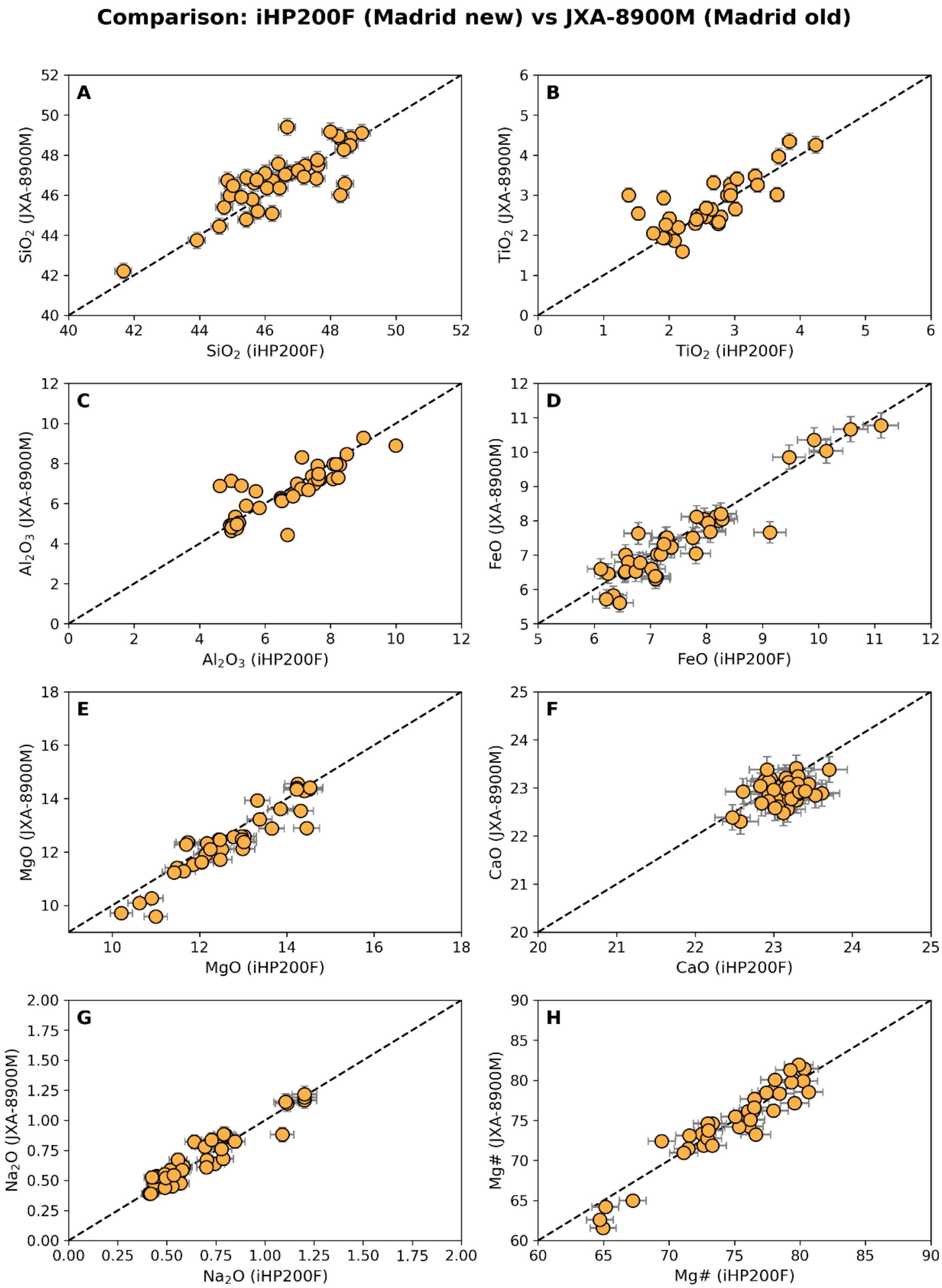

Supplementary Figure 23. *EMPA Comparison Tajogaite 2021. Comparison of major and minor element data for samples from Tajogaite 2021 collected with the iHP200F microprobe in Madrid and the old JXA-8900M microprobe in Madrid. Error bars indicate 2σ uncertainties derived from EMPA counting statistics.*

10. Clustering methods

The clustering was performed on a single matrix containing single-spot analysis (n=316) and pixel-based map data (n= 398,776), which represent complementary observations of the same underlying clinopyroxene chemical variability.

To objectively evaluate the number of clusters (k) for the trace-element clinopyroxene dataset, we applied the elbow method as a quantitative approach, complemented by PCA and t-SNE as statistical tools. The elbow method evaluates clustering performance by examining the total within-cluster sum of squares (WSS) as a function of k. Increasing k reduces WSS, but the rate of decrease diminishes as clusters become more refined[105,106]. The optimal number of clusters is identified at the inflection point where additional clusters yield only marginal improvements in cluster compactness. We evaluated solutions for k ranging from 2 to 10, and the WSS curve shows a clear inflection at k = 6, indicating that the reduction in within-cluster variance decreases markedly beyond this point. This identifies six clusters as the highest-resolution partition that yields substantial improvements in cluster compactness, with further subdivision providing diminishing returns (Supplementary Figure 24).

Projection of the k = 6 assignments into PCA space defines distinct groupings along the principal axes of compositional variance, indicating that clusters correspond to structured variation in the dataset rather than random partitioning. In t-SNE space, the same clusters form well-defined local neighborhoods. t-SNE is a nonlinear dimensionality reduction technique that maps high-dimensional data into a lower-dimensional space while preserving local similarity relationships between observations, making it well-suited for visualizing cluster structure and identifying coherent neighborhoods within complex geochemical datasets. The coherent groupings observed in t-SNE space therefore demonstrate that the six clusters represent genuine compositional populations rather than arbitrary subdivisions of a continuous gradient (Supplementary Figure 24).

Strong textural constraints exist in our dataset, and clustering is expected to reflect texturally defined domains within crystals, including cores and their patchy regions, and concentric zoning such as inner and outer rims (Supplementary Figure 24B). We therefore inspected cluster maps generated for k ranging from 2 to 10 (Supplementary Figure 24A). Comparison with BSE images and compositional maps indicates that k = 6 provides the most interpretable and petrographically meaningful representation of the observed zoning patterns among the solutions tested. We therefore retain k = 6 as a statistically supported, texturally consistent partition of the dataset for subsequent petrological interpretation. We note that no universally correct value of k exists in k-means clustering; the choice is inherently dataset- and context-dependent. However, the agreement between quantitative, visual, and textural criteria gives us confidence that k = 6 is a well-supported and defensible choice for the subsequent petrological interpretation.

Finally, we tested the sensitivity of cluster centroids to dataset composition, given that the combined matrix used for clustering is strongly dominated by pixel-based map data (n = 398,776) relative to single-spot analyses (n = 316). To do so, we use the K-Means model fitted on the full dataset (pixels + spots) as a reference, and separately fitted an independent K-Means model on a balanced dataset combining all single-spot analyses (n = 316) with a random subsample of 1000 pixels. The balanced model's six clusters were matched to the reference clusters by nearest-centroid distance. To visualize this, boxplots show the chemical distribution of each cluster from the full-dataset clustering, with white triangles overlaid representing the median chemical composition of the corresponding matched cluster from the balanced-dataset clustering (Supplementary Figure 25).

Five of the six clusters show close agreement with the full-dataset solution across all eight trace elements. Only cluster 2 shows a comparatively larger deviation in Sr, though this value still falls within the overall range of Sr

observed for that cluster in the full dataset. Overall, this indicates that the six-cluster solution is robust to the pixel:spot imbalance in the full dataset. The Python script used for clustering and evaluation of quantitative methods for the selection of k is provided in the Github repository (https://github.com/AlbCaracc/Caracciolo_et_al_2026_CumbreVieja) and archived in the Zenodo link (https://doi.org/10.5281/zenodo.21081067)[107].

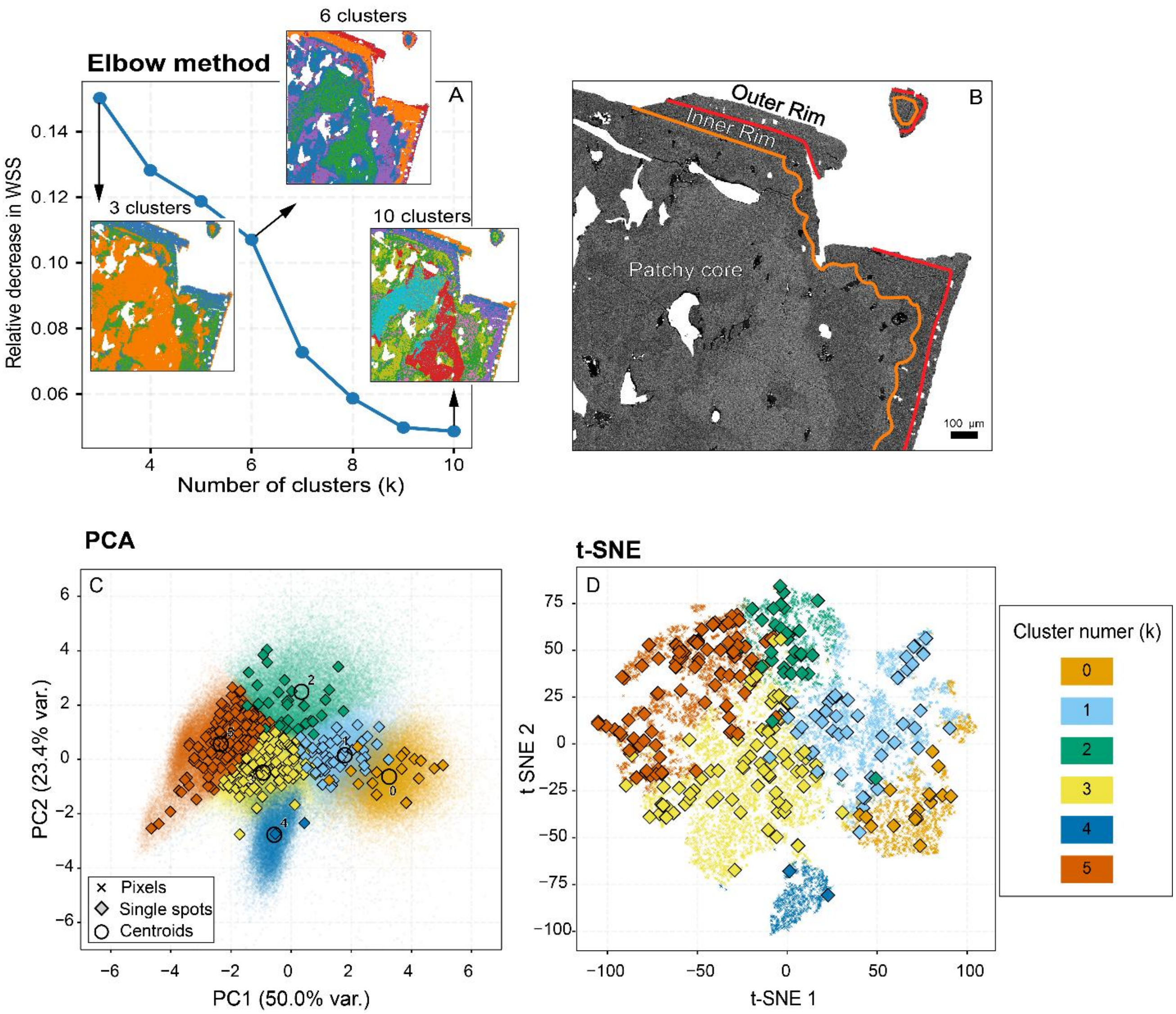

***Supplementary Figure 24**. Quantitative methods for selection of k. (A) Elbow method, showing the total variance within each cluster, expressed as within sum of squares (WSS), and cluster-coloured maps of a representative crystal for k = 3, 6, and 10. (B) Backscattered electron (BSE) image of the same crystal, highlighting texturally defined domains (outer rim, inner rim, and patchy core). (C) Single spots and pixel-data from maps in the principal component analysis (PCA) space. (D) t-SNE projection of the dataset coloured by cluster number, showing the formation of distinct compositional groups for both single spots and map pixel-data.*

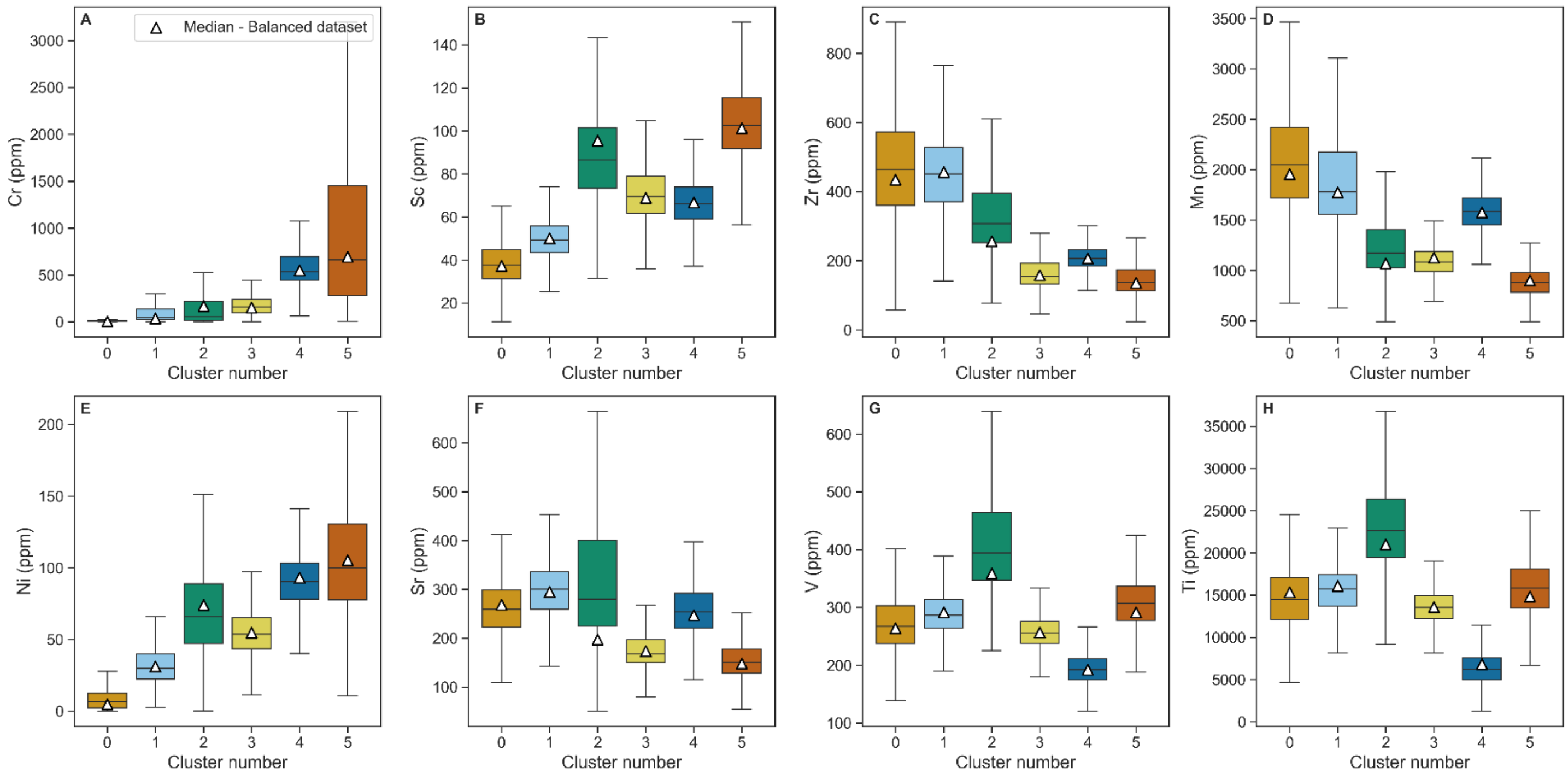

Supplementary Figure 25. *Clustering data imbalance**.** Boxplots show the distribution of trace-element concentrations for the six clusters obtained from the K-means model fitted to the full dataset (LA-ICP-MS map pixels and single-spot analyses). Elements shown are Cr, Sc, Zr, Mn, Ni, Sr, V, and Ti, similarly to Fig. 8A-H. White triangles indicate the median composition of the corresponding clusters produced by an independent K-means model fitted to a balanced dataset composed of all single-spot analyses (n = 316) and a random downsample of 1000 map pixels. The clusters from the balanced model were matched to the reference full-dataset clusters by nearest-centroid distance in the scaled eight-element compositional space. The close agreement between the balanced-cluster medians and the full-dataset boxplots indicates that the main six-cluster structure is reproduced when clustering is rerun on a dataset less dominated by map pixels, supporting the robustness of the solution to the pixel:spot imbalance.*

11. Individual cpx maps

Supplementary Figure 26*. LA-ICP-MS chemical maps. We present 15 figures of clinopyroxene trace element maps for Cr, Mn, Ni, Sc, Sr, Ti, V, and Zr. For each element and each sample, the optimal visualization method, either logarithmic scaling, histogram equalization (HE), or adaptive histogram equalization (AHE), was selected to enhance chemical contrast, depending on the statistical distribution and dynamic range of each element (Panels A–H). Note that colors are not comparable between images, as this approach is designed to enhance zoning contrast within each map. Logarithmic transformation was applied when elemental concentrations spanned several orders of magnitude, helping to highlight subtle variation in low-concentration domains that would otherwise be suppressed by linear rescaling. Histogram equalization, a global contrast enhancement technique, was applied to datasets with moderate skew to redistribute pixel intensities and make use of the full dynamic range of the display. For highly skewed or spatially heterogeneous data, adaptive histogram equalization was employed. Unlike the HE method, AHE operates on small image regions to enhance local contrast and resolve fine-scale compositional structures. The quantitative range for each element (in ppm) is shown in the title of each color bar. In each figure, Panel I displays the BSE image of the mapped area. BSE images were acquired after crystal mapping and gentle repolishing, which may result in minor differences between the crystals shown in the trace element maps and those in the BSE image. The eruption, clinopyroxene name, and rock type (basanite or tephrite) are indicated at the top of each figure.*

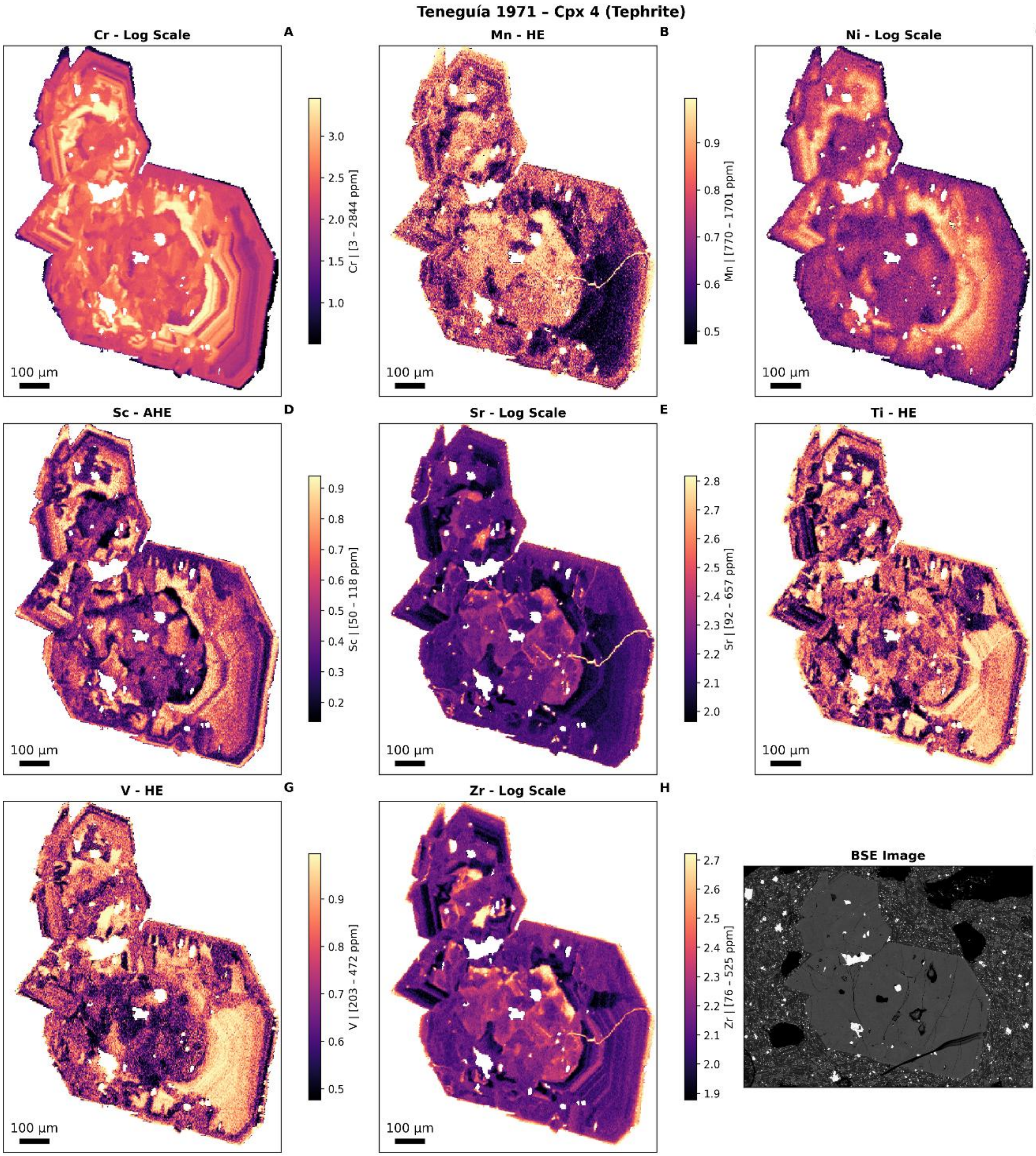

Teneguía 1971 – Cpx 4 (Tephrite)
Cr - Log Scale
A
Cr | [3 – 2844 ppm]
Mn - HE
B
Mn | [770 – 1701 ppm]
Ni - Log Scale
C
Ni | [30 – 142 ppm]
Sc - AHE
D
Sc | [50 – 118 ppm]
Sr - Log Scale
E
Sr | [92 – 657 ppm]
Ti - HE
F
Ti | [9900 – 27547 ppm]
V - HE
G
V | [203 – 472 ppm]
Zr - Log Scale
H
Zr | [76 – 525 ppm]
BSE Image
I
100 µm

Teneguía 1971 – Cpx 3-5 (Tephrite)

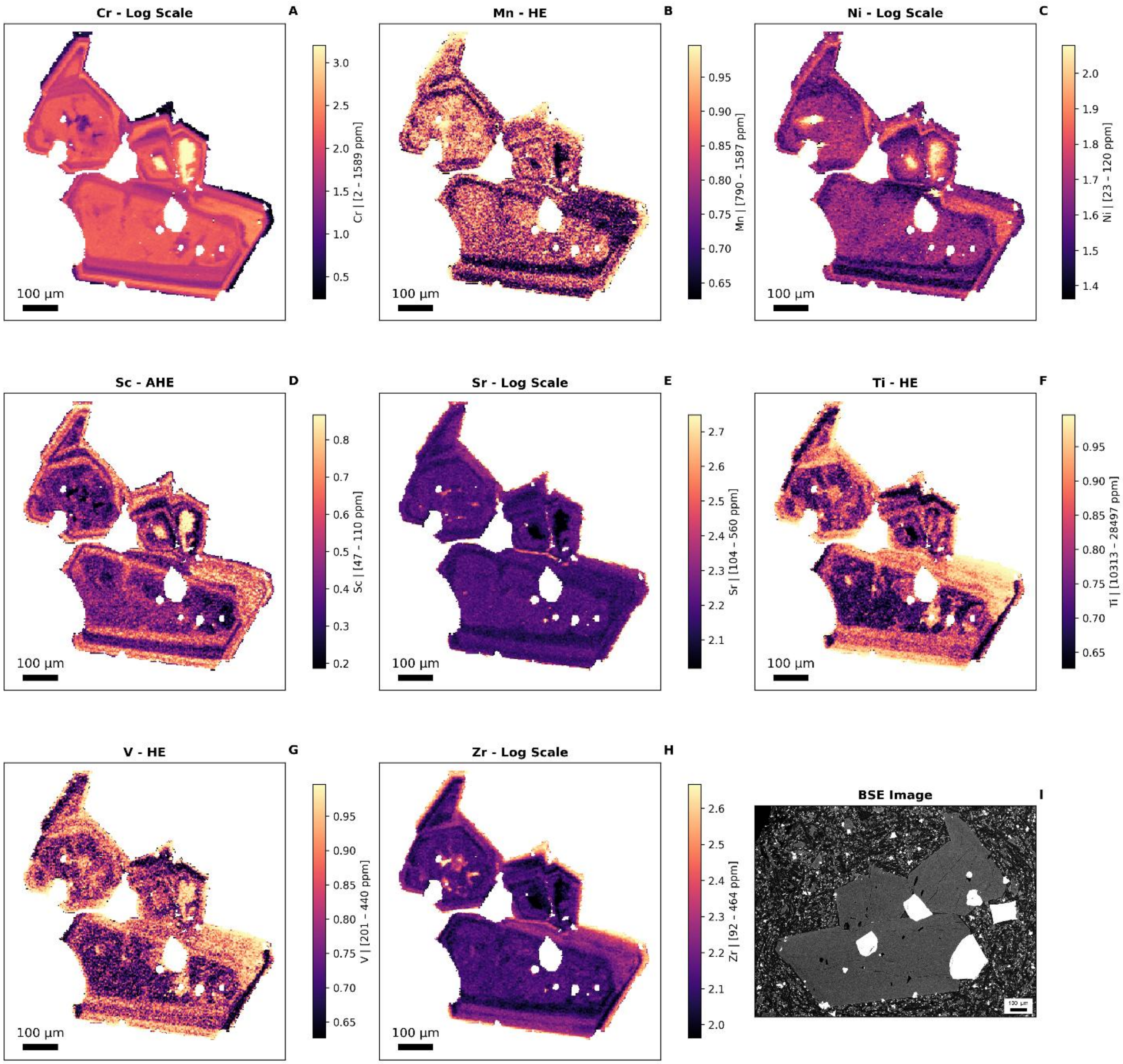

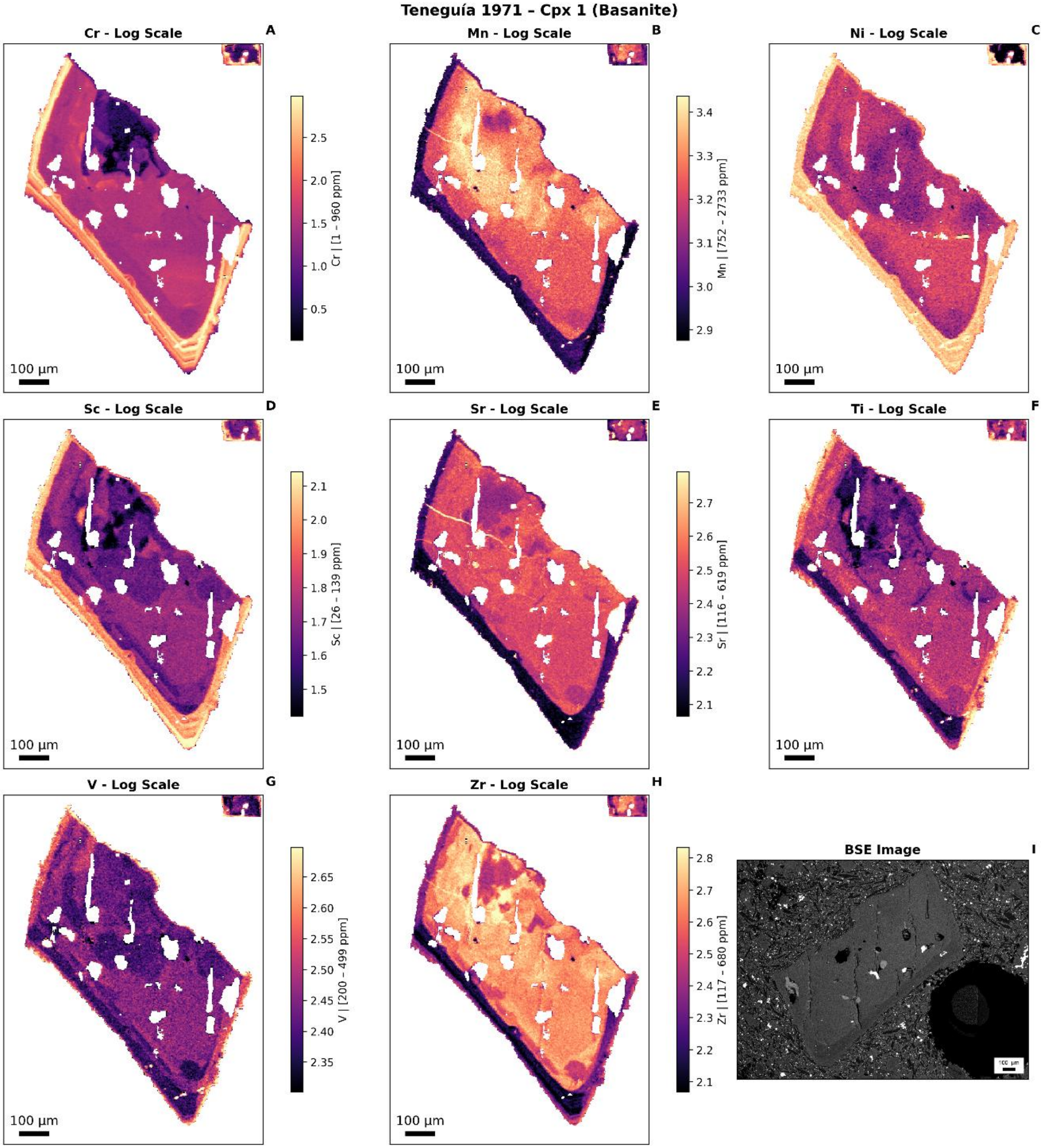
Teneguía 1971 – Cpx 1 (Basanite)
Cr - Log Scale
A
Cr | [1 – 960 ppm]
Mn - Log Scale
B
Mn | [752 – 2733 ppm]
Ni - Log Scale
C
Ni | [6 – 102 ppm]
Sc - Log Scale
D
Sc | [26 - 139 ppm]
Sr - Log Scale
E
Sr | [116 – 619 ppm]
Ti - Log Scale
F
Ti | [10086 – 29311 ppm]
V - Log Scale
G
V | [200 – 499 ppm]
Zr - Log Scale
H
Zr | [117 – 680 ppm]
BSE Image
I
100 µm

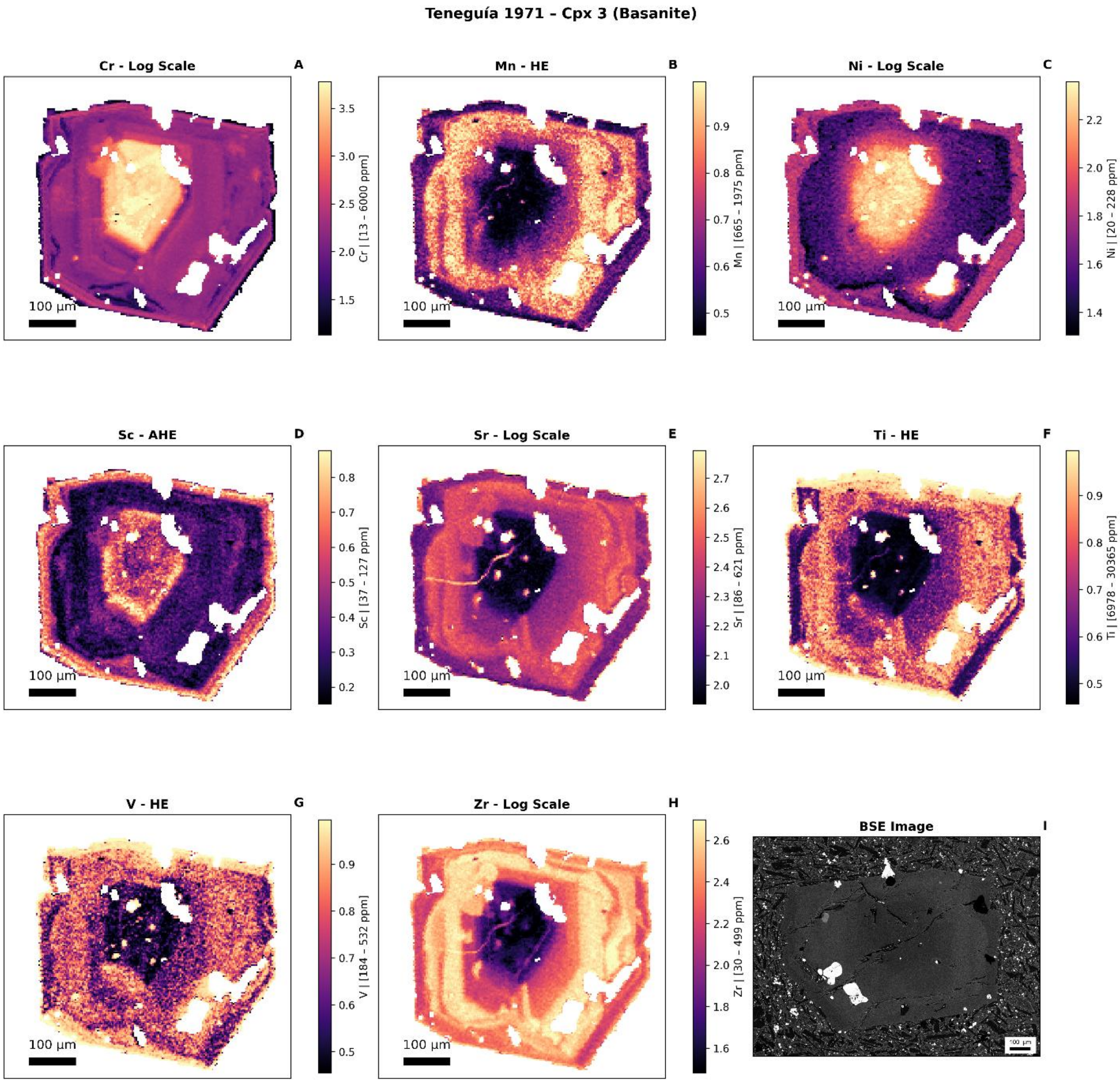

Teneguía 1971 – Cpx 3 (Basanite)
Cr - Log Scale
A
Cr | [13 – 6000 ppm]
Mn - HE
B
Mn | [665 – 1975 ppm]
Ni - Log Scale
C
Ni | [20 – 228 ppm]
Sc - AHE
D
Sc | [37 – 127 ppm]
Sr - Log Scale
E
Sr | [86 – 621 ppm]
Ti - HE
F
Ti | [6978 – 30365 ppm]
V - HE
G
V | [184 – 532 ppm]
Zr - Log Scale
H
Zr | [30 – 499 ppm]
BSE Image
I
100 µm

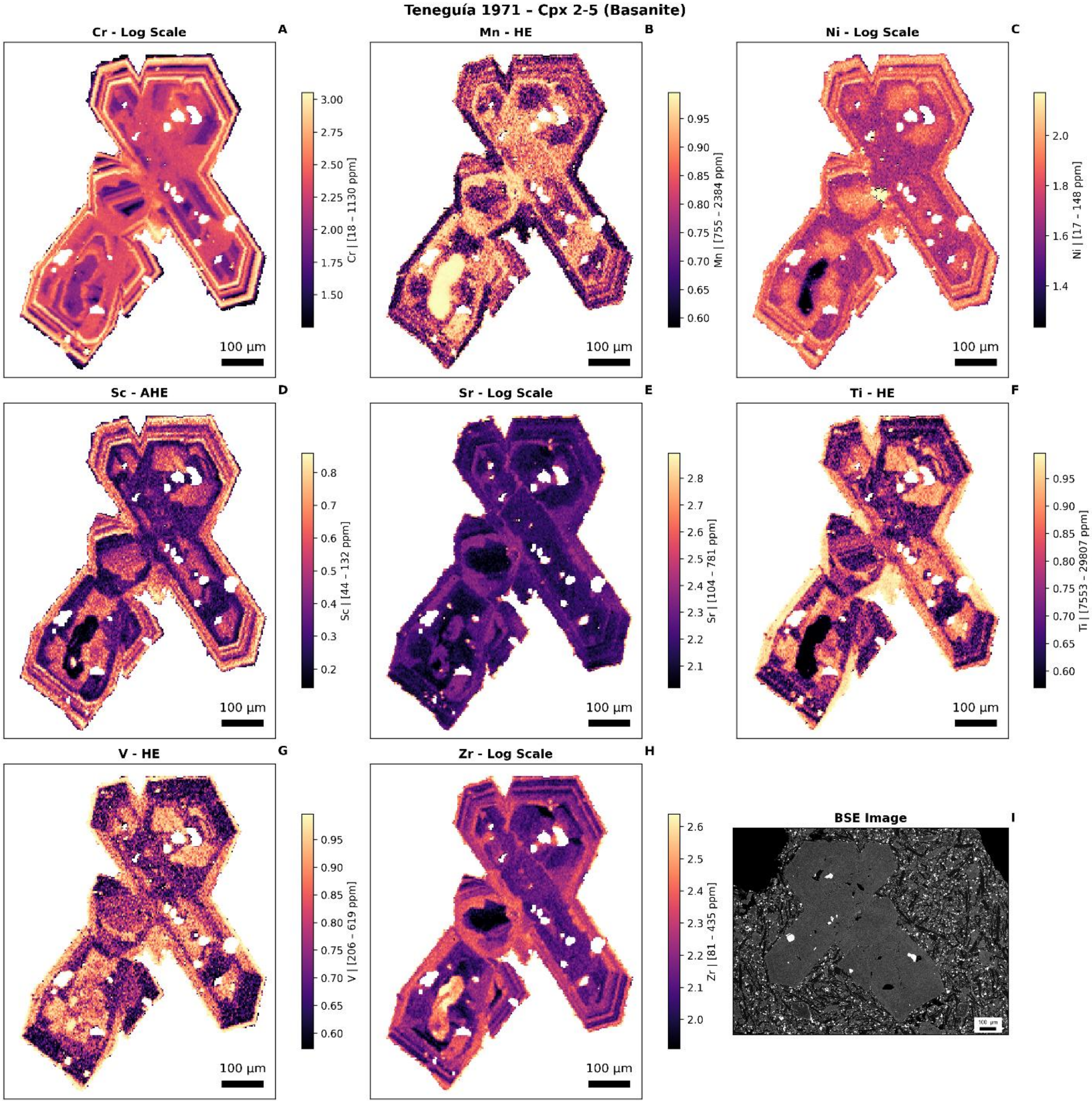
Teneguía 1971 – Cpx 2-5 (Basanite)
Cr - Log Scale
A
Cr | [18 – 1130 ppm]
Mn - HE
B
Mn | [755 – 2384 ppm]
Ni - Log Scale
C
Ni | [17 – 148 ppm]
Sc - AHE
D
Sc | [44 – 132 ppm]
Sr - Log Scale
E
Sr | [104 – 781 ppm]
Ti - HE
F
Ti | [7553 – 29807 ppm]
V - HE
G
V | [206 – 619 ppm]
Zr - Log Scale
H
Zr | [81 – 435 ppm]
BSE Image
I
100 µm

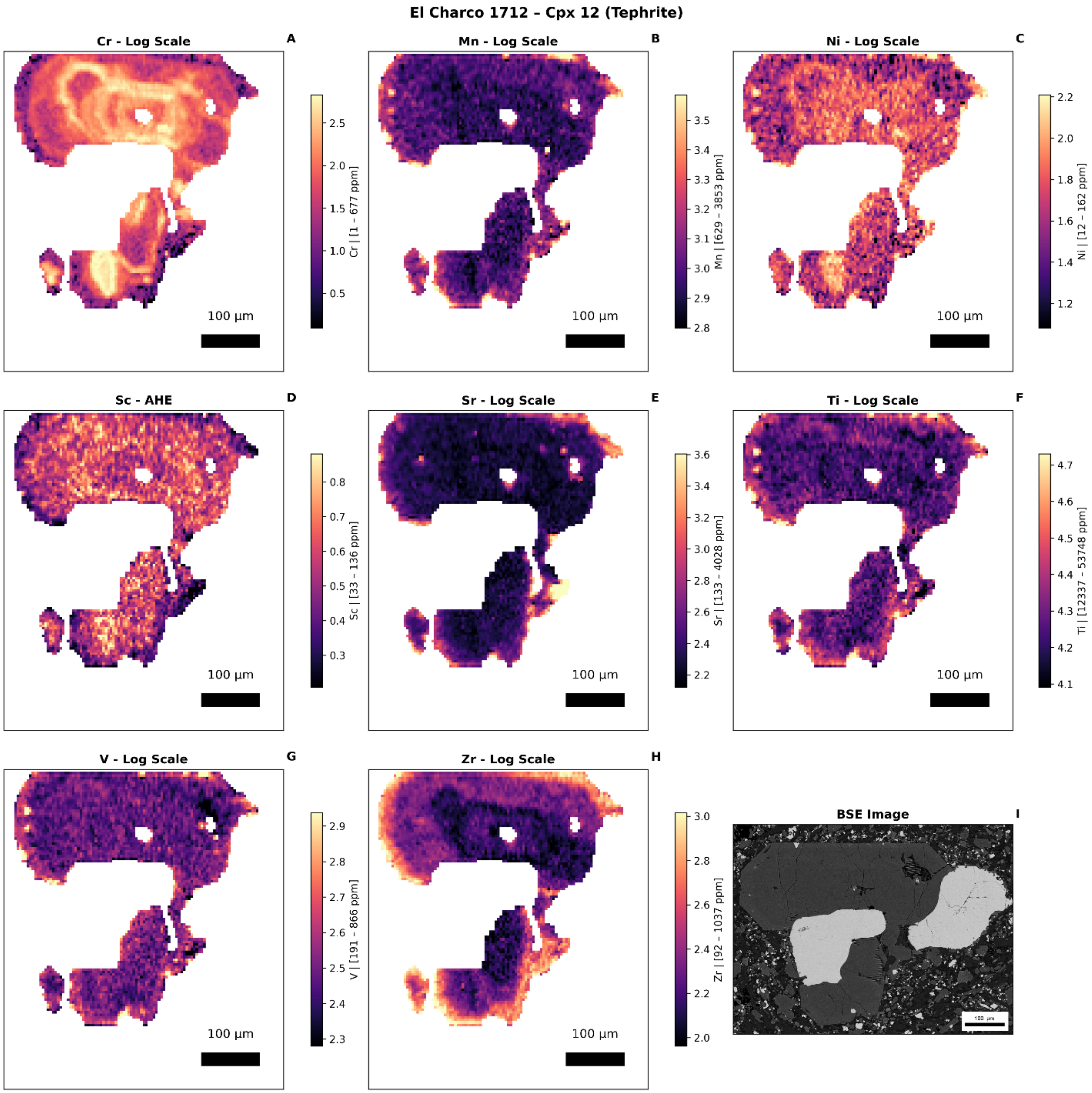
El Charco 1712 – Cpx 12 (Tephrite)
Cr - Log Scale
A
Cr | [1 – 677 ppm]
100 µm
Mn - Log Scale
B
Mn | [629 – 3853 ppm]
100 µm
Ni - Log Scale
C
Ni | [12 – 162 ppm]
100 µm
Sc - AHE
D
Sc | [33 – 136 ppm]
100 µm
Sr - Log Scale
E
Sr | [133 – 4028 ppm]
100 µm
Ti - Log Scale
F
Ti | [12337 – 53748 ppm]
100 µm
V - Log Scale
G
V | [191 – 866 ppm]
100 µm
Zr - Log Scale
H
Zr | [92 – 1037 ppm]
100 µm
BSE Image
I

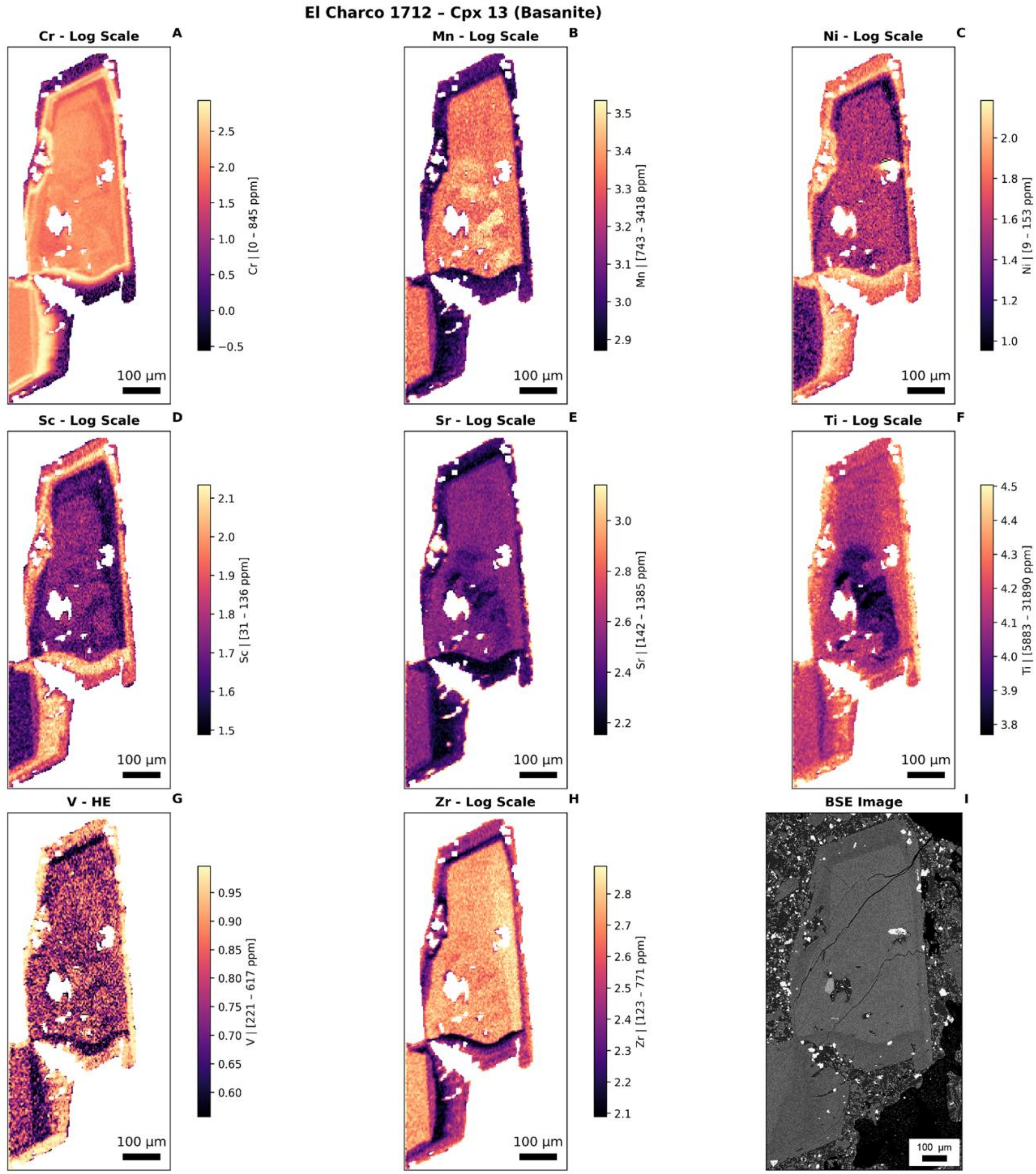
El Charco 1712 – Cpx 13 (Basanite)
Cr - Log Scale
A
Cr | [0 – 845 ppm]
Mn - Log Scale
B
Mn | [743 – 3418 ppm]
Ni - Log Scale
C
Ni | [9 – 153 ppm]
Sc - Log Scale
D
Sc | [31 – 136 ppm]
Sr - Log Scale
E
Sr | [142 – 1385 ppm]
Ti - Log Scale
F
Ti | [5883 – 31890 ppm]
V - HE
G
V | [221 – 617 ppm]
Zr - Log Scale
H
Zr | [123 – 771 ppm]
BSE Image
I
100 µm

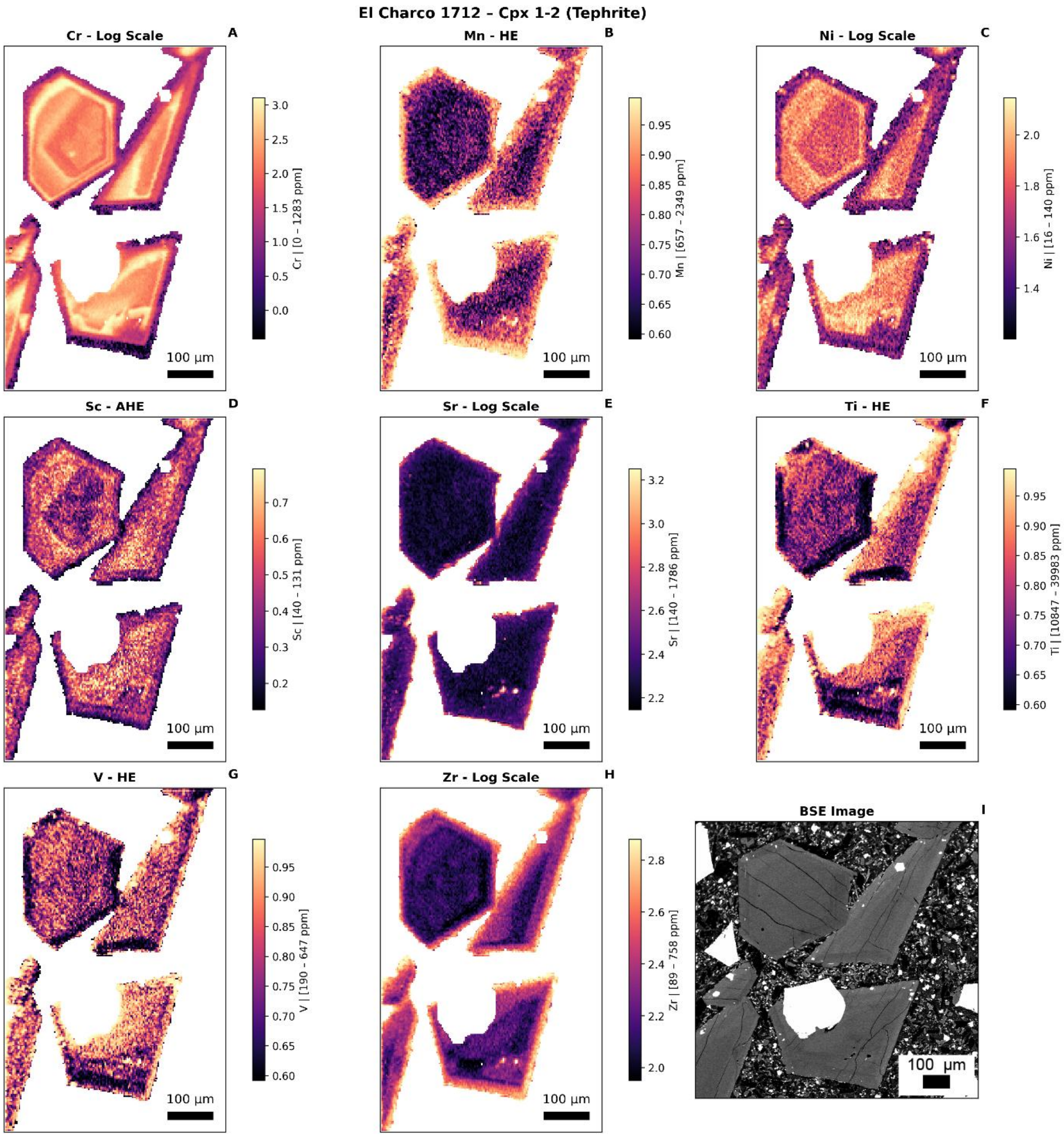
El Charco 1712 – Cpx 1-2 (Tephrite)
Cr - Log Scale
A
Cr | [0 – 1283 ppm]
Mn - HE
B
Mn | [657 – 2349 ppm]
Ni - Log Scale
C
Ni | [16 – 140 ppm]
Sc - AHE
D
Sc | [40 – 131 ppm]
Sr - Log Scale
E
Sr | [140 – 1786 ppm]
Ti - HE
F
Ti | [10847 – 39983 ppm]
V - HE
G
V | [190 – 647 ppm]
Zr - Log Scale
H
Zr | [89 – 758 ppm]
BSE Image
I
100 µm

El Charco 1712 – Cpx 21 (Basanite)

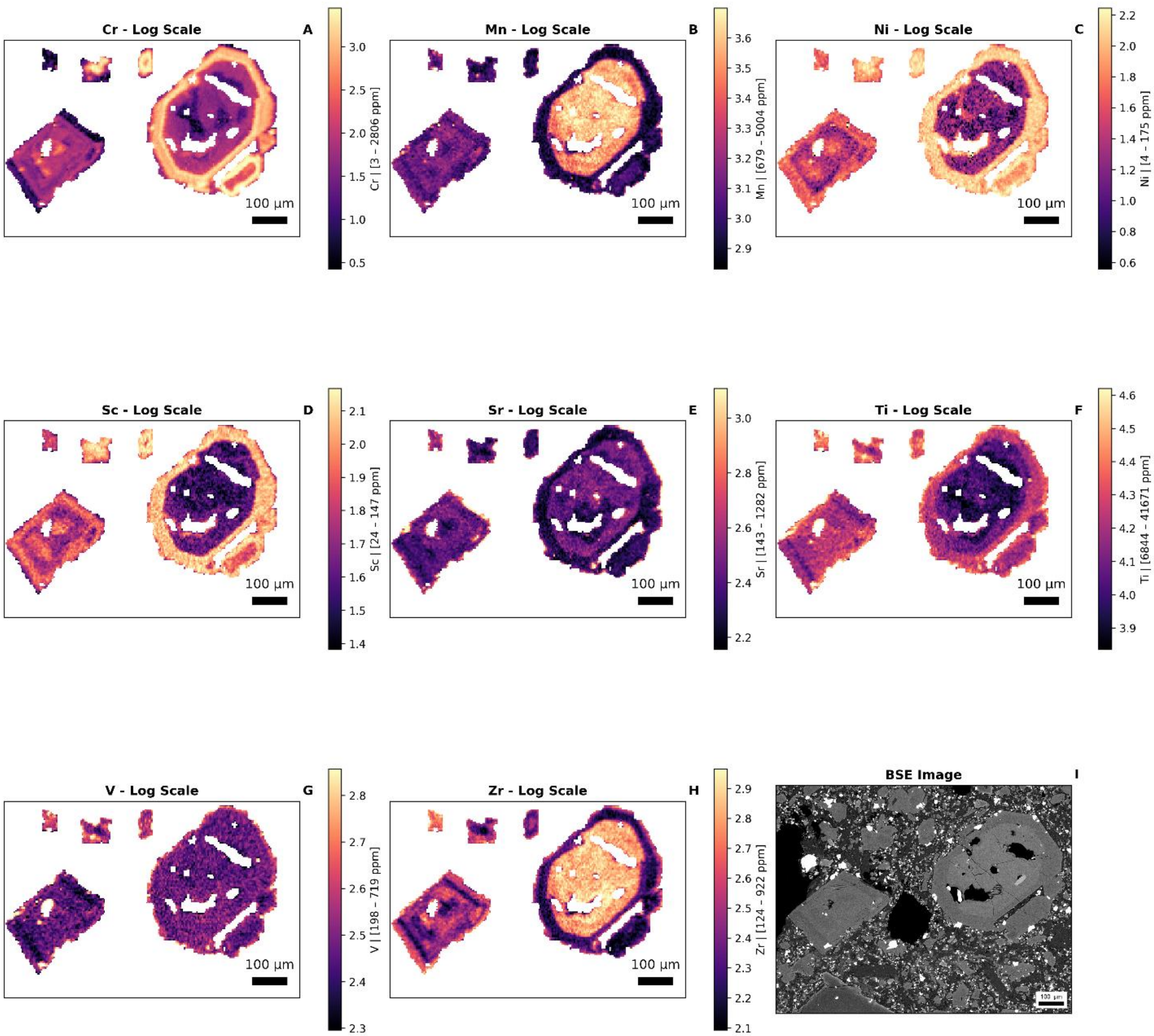

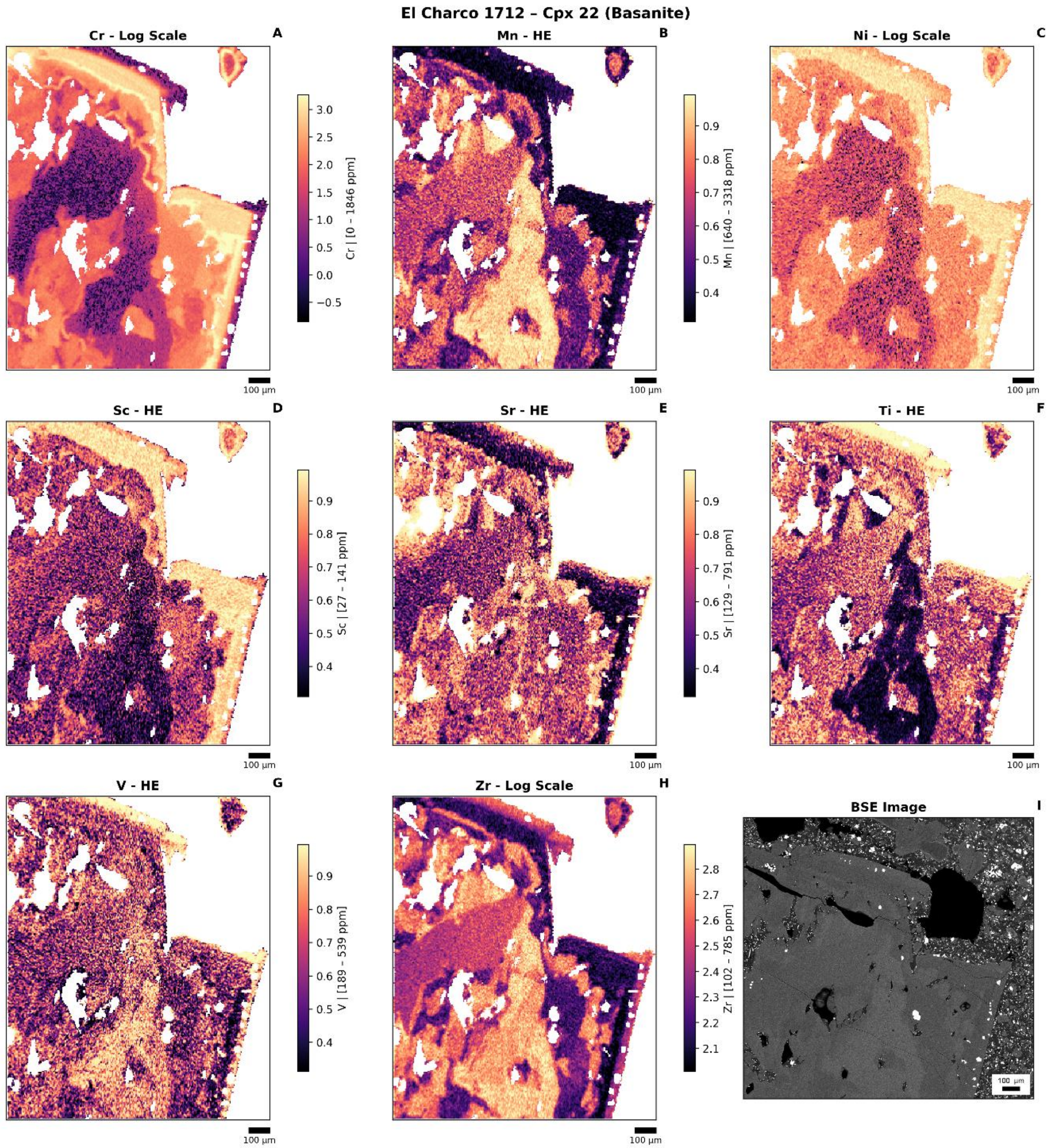
El Charco 1712 – Cpx 22 (Basanite)
Cr - Log Scale
A
Cr | [0 - 1846 ppm]
Mn - HE
B
Mn | [640 - 3318 ppm]
Ni - Log Scale
C
Ni | [0 - 177 ppm]
Sc - HE
D
Sc | [27 - 141 ppm]
Sr - HE
E
Sr | [129 - 791 ppm]
Ti - HE
F
Ti | [7105 - 29056 ppm]
V - HE
G
V | [189 - 539 ppm]
Zr - Log Scale
H
Zr | [102 - 785 ppm]
BSE Image
I
100 µm

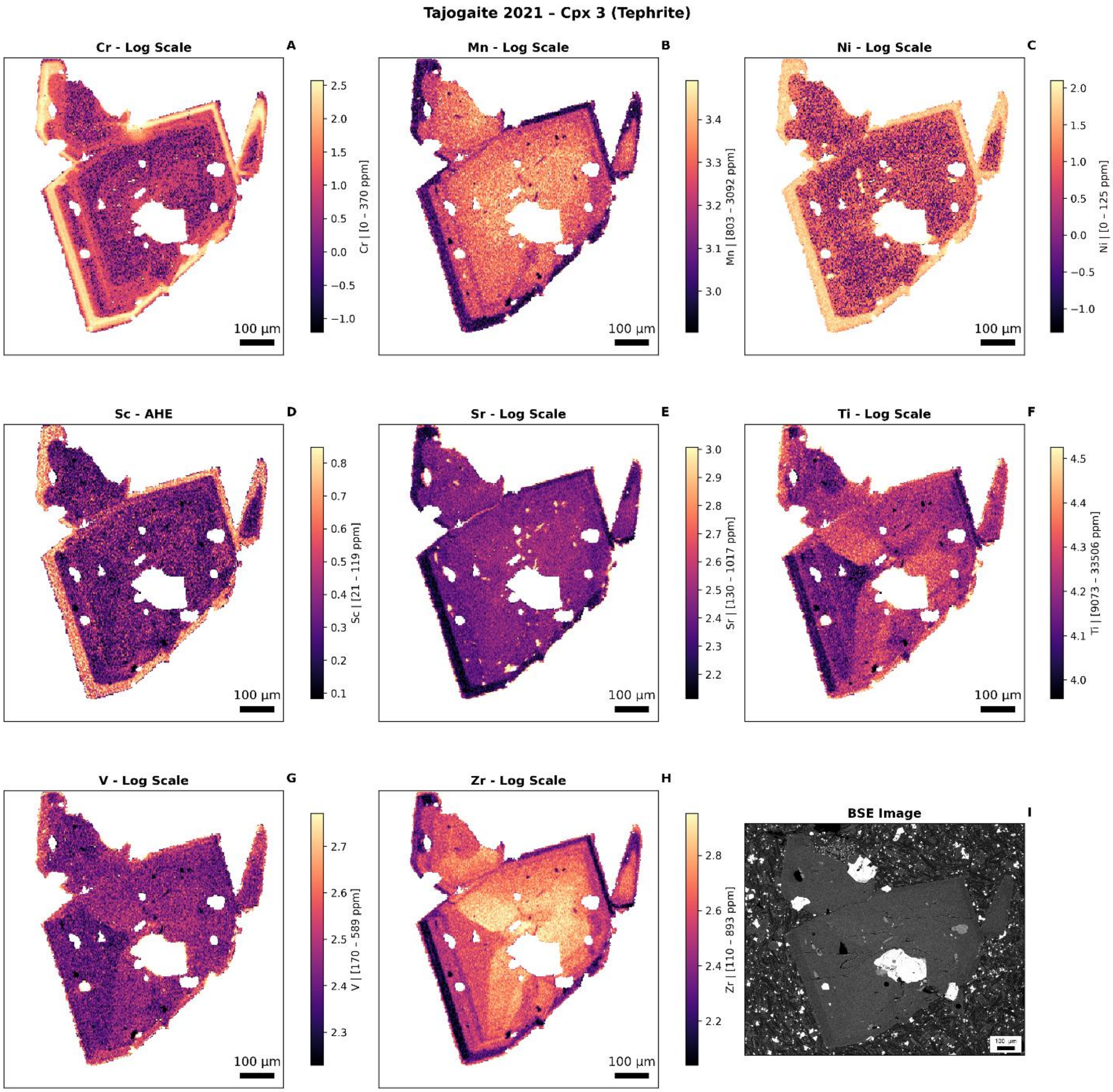
Tajogaite 2021 - Cpx 3 (Tephrite)
Cr - Log Scale
A
Cr | [0 - 370 ppm]
Mn - Log Scale
B
Mn | [803 - 3092 ppm]
Ni - Log Scale
C
Ni | [0 - 125 ppm]
Sc - AHE
D
Sc | [21 - 119 ppm]
Sr - Log Scale
E
Sr | [130 - 1017 ppm]
Ti - Log Scale
F
Ti | [9073 - 33506 ppm]
V - Log Scale
G
V | [170 - 589 ppm]
Zr - Log Scale
H
Zr | [110 - 893 ppm]
BSE Image
I
100 µm

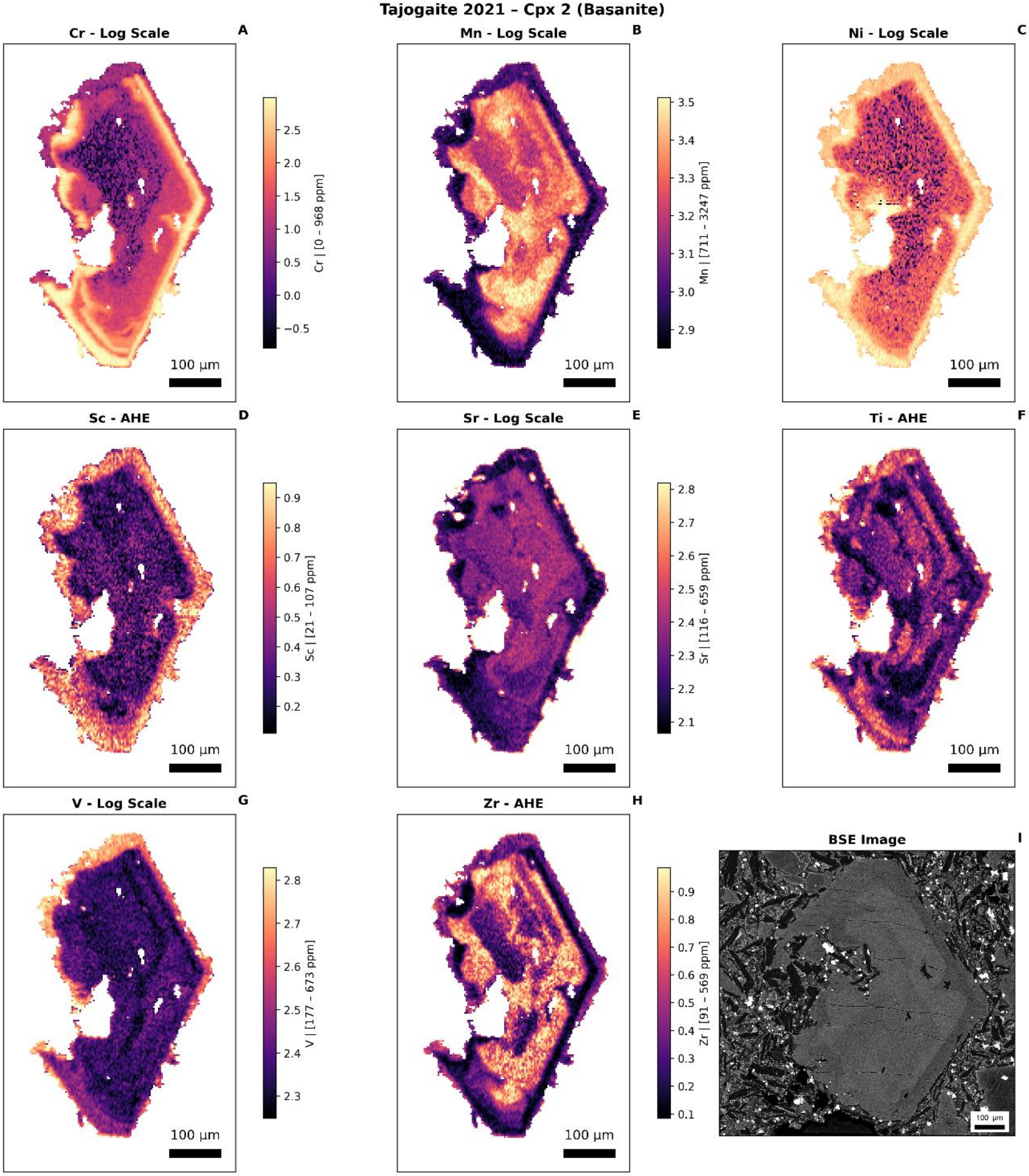
Tajogaite 2021 – Cpx 2 (Basanite)
Cr - Log Scale
A
Cr | [0 – 968 ppm]
Mn - Log Scale
B
Mn | [711 – 3247 ppm]
Ni - Log Scale
C
Ni | [0 – 151 ppm]
Sc - AHE
D
Sc | [21 – 107 ppm]
Sr - Log Scale
E
Sr | [116 – 659 ppm]
Ti - AHE
F
Ti | [10410 – 38846 ppm]
V - Log Scale
G
V | [177 – 673 ppm]
Zr - AHE
H
Zr | [91 – 569 ppm]
BSE Image
I
100 µm

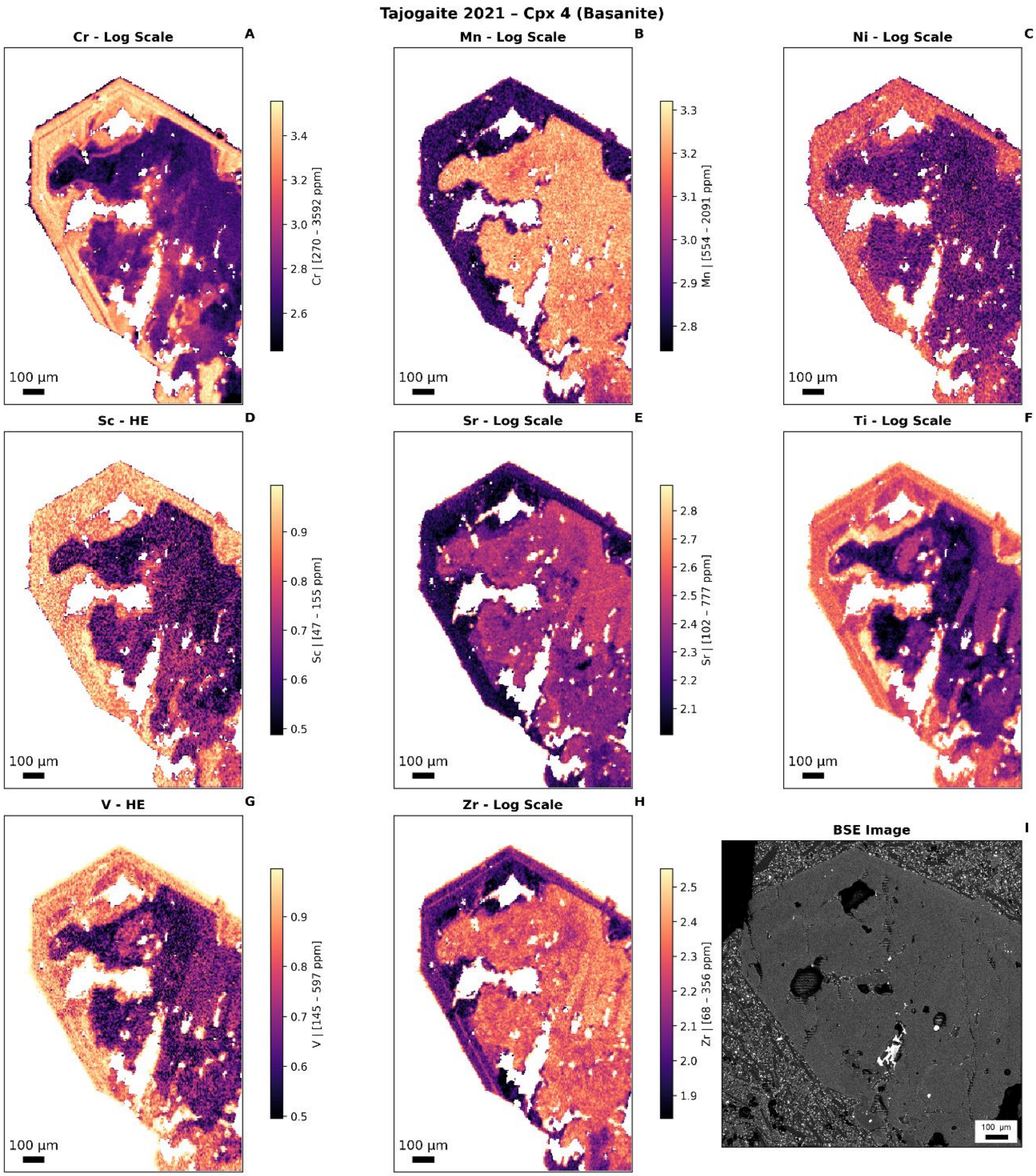
Tajogaite 2021 – Cpx 4 (Basanite)
Cr - Log Scale
A
Cr | [270 – 3592 ppm]
Mn - Log Scale
B
Mn | [554 – 2091 ppm]
Ni - Log Scale
C
Ni | [56 – 275 ppm]
Sc - HE
D
Sc | [47 – 155 ppm]
Sr - Log Scale
E
Sr | [102 – 777 ppm]
Ti - Log Scale
F
Ti | [3371 – 29672 ppm]
V - HE
G
V | [145 – 597 ppm]
Zr - Log Scale
H
Zr | [68 – 356 ppm]
BSE Image
I
100 µm

Tajogaite 2021 – Cpx 5 (Basanite)

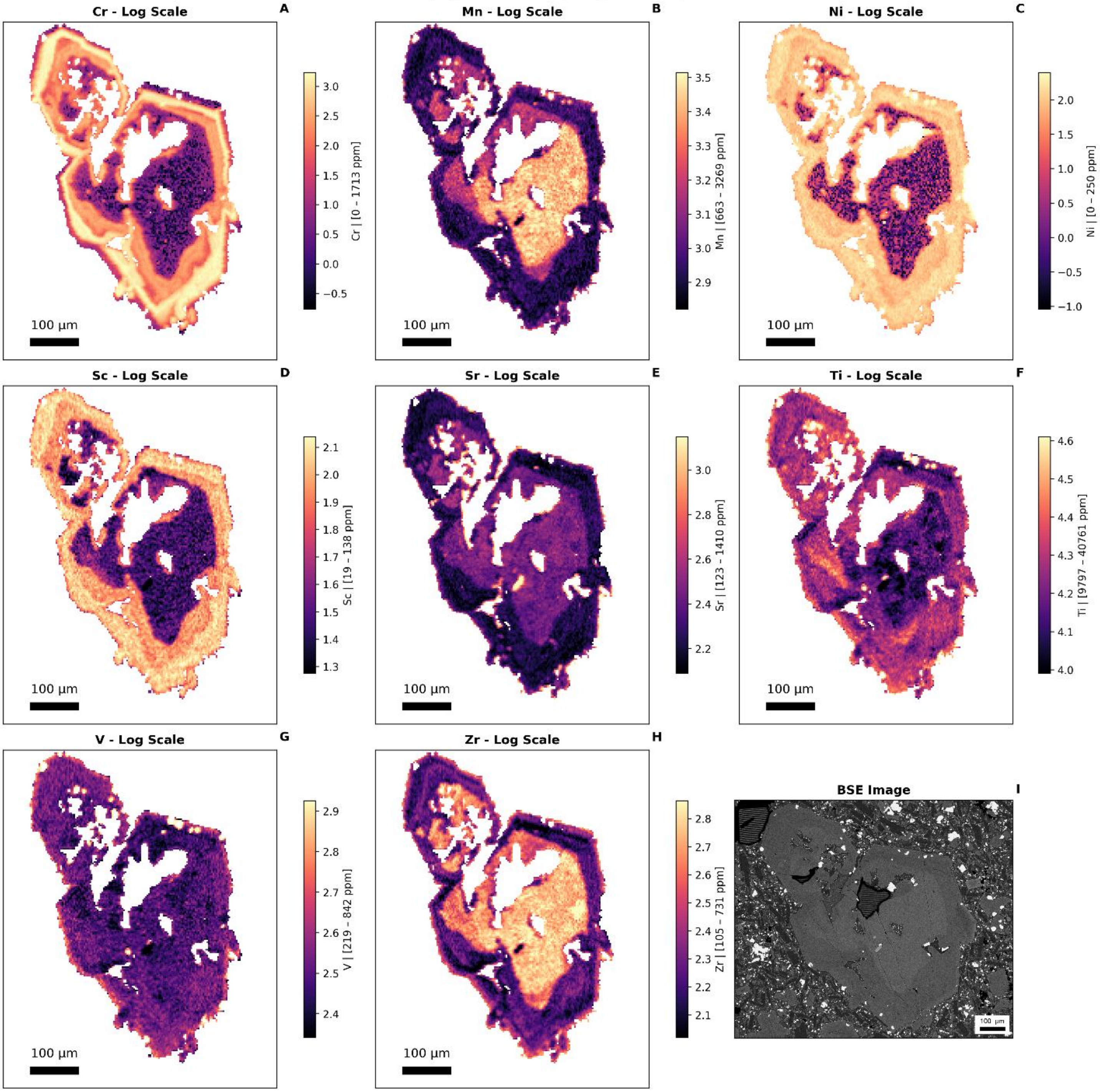

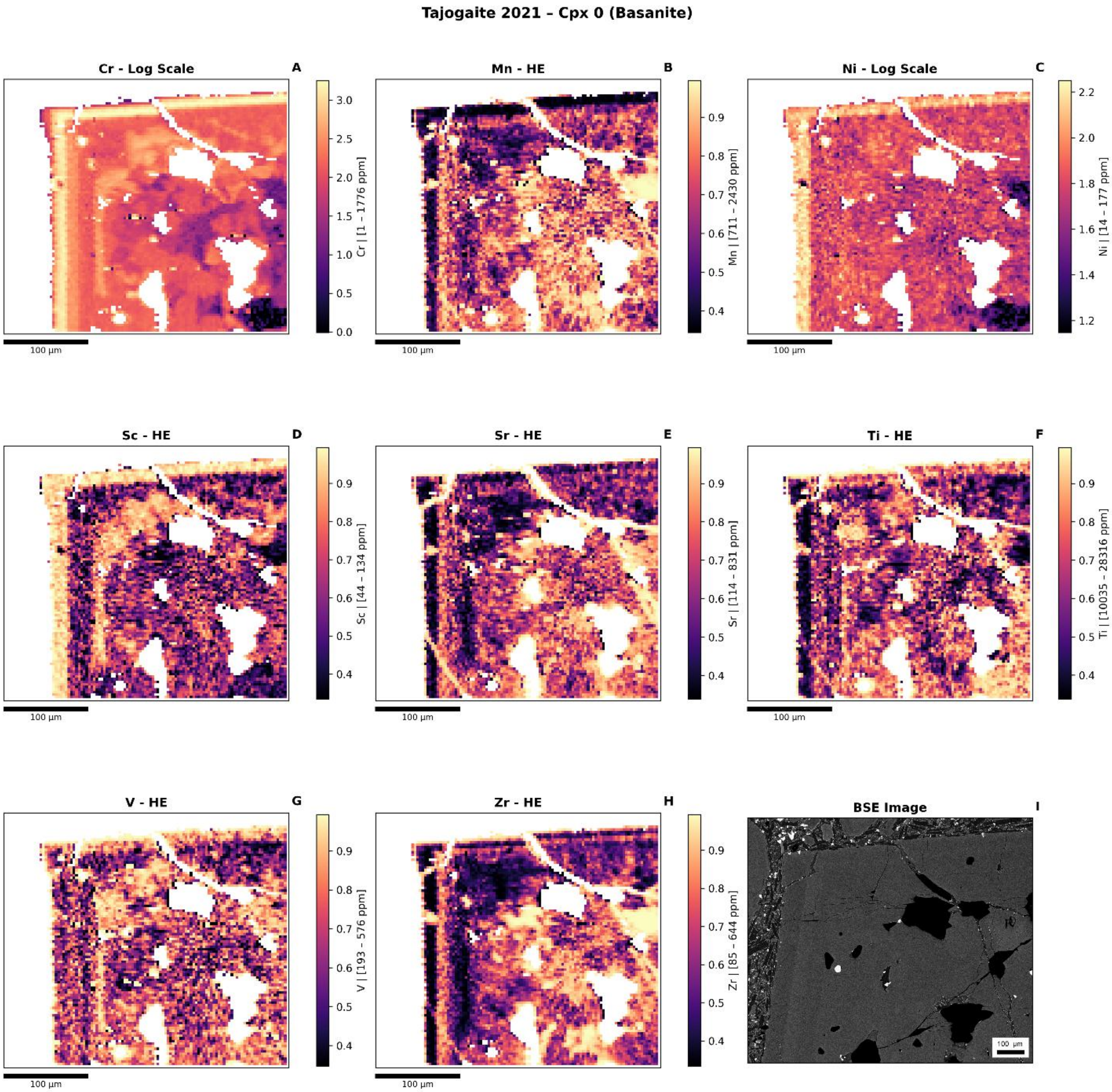
Tajogaite 2021 – Cpx 0 (Basanite)
Cr - Log Scale
A
Cr | [1 – 1776 ppm]
Mn - HE
B
Mn | [711 – 2430 ppm]
Ni - Log Scale
C
Ni | [14 – 177 ppm]
Sc - HE
D
Sc | [44 – 134 ppm]
Sr - HE
E
Sr | [114 – 831 ppm]
Ti - HE
F
Ti | [10035 – 28316 ppm]
V - HE
G
V | [193 – 576 ppm]
Zr - HE
H
Zr | [85 – 644 ppm]
BSE Image
I
100 µm

12. Supplementary References

1. Wilson, J. T. Mantle plumes and plate motions. *Tectonophysics* **19**, 149–164 (1973).
2. Anguita, F., Fernández, C., Márquez, Á., León, R. & Casillas, R. The Canary hotspot revisited: Refutation of the Hawaii paradigm and an alternative, plate-based hypothesis. *Earth-Science Reviews* vol. 261 Preprint at https://doi.org/10.1016/j.earscirev.2024.105038 (2025).
3. Carracedo, J. C. *et al.* The 2021 eruption of the Cumbre Vieja volcanic ridge on La Palma, Canary Islands. *Geology Today* **38**, 94–107 (2022).
4. Hernandez-Pacheco, A. & Valls, M. C. The historic eruptions of La Palma Island (Canaries). *Arquipélago. Série Ciências da Natureza* 83–94 (1984).
5. Longpré, M. A. & Felpeto, A. Historical volcanism in the Canary Islands; part 1: A review of precursory and eruptive activity, eruption parameter estimates, and implications for hazard assessment. *Journal of Volcanology and Geothermal Research* **419**, (2021).
6. Klugel, A., Schmincke, H.-U., White, J. D. L. & Hoernle, K. A. *Chronology and Volcanology of the 1949 Multi-Vent Rift-Zone ž / Eruption on La Palma Canary Islands*. *Journal of Volcanology and Geothermal Research* vol. 94 www.elsevier.comrlocaterjvolgeores (1999).
7. Klügel, A., Albers, E. & Hansteen, T. H. Mantle and Crustal Xenoliths in a Tephriphonolite From La Palma (Canary Islands): Implications for Phonolite Formation at Oceanic Island Volcanoes. *Front. Earth Sci. (Lausanne).* **10**, (2022).
8. Romero Ruiz, M. del C. Las manifestaciones volcánicas históricas del Archipiélago Canario. http://riull.ull.es/xmlui/handle/915/10113 (1990).
9. Chicharro, N. La erupción de El Charco de 1712, La Palma. Características petrológicas e implicaciones en la peligrosidad volcánica. (Universidad Complutense de Madrid, 2024). doi:https://docta.ucm.es/entities/publication/b8280.
10. Afonso, A., Aparicio, A., Hernández-Pacheco, A. & Badiola, E. R. Morphology evolution of Teneguía volcano area. *Estudios Geológicos, Vol. Teneguía* 19–26 (1974).
11. Bonadonna, C. *et al.* Physical Characterization of Long-Lasting Hybrid Eruptions: The 2021 Tajogaite Eruption of Cumbre Vieja (La Palma, Canary Islands). *J. Geophys. Res. Solid Earth* **127**, (2022).
12. Torres-González, P. A. *et al.* Unrest signals after 46 years of quiescence at Cumbre Vieja, La Palma, Canary Islands. *Journal of Volcanology and Geothermal Research* **392**, 106757 (2020).
13. Fernández, J. *et al.* Detection of volcanic unrest onset in La Palma, Canary Islands, evolution and implications. *Sci. Rep.* **11**, (2021).

14. Pankhurst, M. J. *et al.* Rapid response petrology for the opening eruptive phase of the 2021 Cumbre Vieja eruption, La Palma, Canary Islands. *Volcanica* (2022).

15. Ubide, T. *et al.* Discrete magma injections drive the 2021 La Palma eruption. *Sci. Adv.* https://www.science.org (2023).

16. Day, J. M. D. *et al.* Mantle source characteristics and magmatic processes during the 2021 La Palma eruption. *Earth Planet. Sci. Lett.* **597**, (2022).

17. Dayton, K. *et al.* Deep magma storage during the 2021 La Palma eruption. *Sci. Adv.* **9**, 1–8 (2023).

18. Bonechi, B. *et al.* Magma residence time, ascent rate and eruptive style of the November ash-laden activity during the 2021 Tajogaite eruption (La Palma, Spain). *Volcanica* **7**, 51–65 (2024).

19. Chamberlain, K. J. *et al.* Crystal cargo perspectives on magma assembly and dynamics during the 2021 Tajogaite eruption, La Palma, Canary Islands. *Volcanica* **8**, 399–425 (2025).

20. Fabbrizio, A. *et al.* Phase equilibrium experiments and thermodynamic simulations to constrain the pre-eruptive conditions of the 2021 Tajogaite eruption (Cumbre Vieja volcano, La Palma, Canary Islands). *Journal of Volcanology and Geothermal Research* **442**, (2023).

21. Scarrow, J. H. *et al.* Decoding links between magmatic processes and eruption dynamics: whole-rock time series petrology of the 2021 Tajogaite eruption, La Palma. *Volcanica* **7**, 953–980 (2024).

22. Barker, A. K., Troll, V. R., Carracedo, J. C. & Nicholls, P. A. The magma plumbing system for the 1971 Teneguía eruption on La Palma, Canary Islands. *Contributions to Mineralogy and Petrology* **170**, 1–21 (2015).

23. González-García, D., Boulesteix, T., Klügel, A. & Holtz, F. Bubble-enhanced basanite–tephrite mixing in the early stages of the Cumbre Vieja 2021 eruption, La Palma, Canary Islands. *Sci. Rep.* **13**, (2023).

24. Weis, F. A., Skogby, H., Troll, V. R., Deegan, F. M. & Dahren, B. Magmatic water contents determined through clinopyroxene: Examples from the Western Canary Islands, Spain. *Geochemistry, Geophysics, Geosystems* **16**, 2127–2146 (2015).

25. Putirka, K. D. Thermometers and barometers for volcanic systems. *Rev. Mineral. Geochem.* **69**, 61–120 (2008).

26. Palme, H. & O'Neill, H. Cosmochemical Estimates of Mantle Composition. in *Treatise on Geochemistry: Second Edition* vol. 3 1–39 (Elsevier Inc., 2014).

27. Carracedo, J. C., Badiola, E. R., Guillou, H., De La Nuez, J. & Pérez-Torrado, F. J. Geology and volcanology of La Palma and El Hierro, Western Canaries. *Estudios Geológicos* **57**, 175–273 (2001).

28. Day, J. M. D., Pearson, D. G., Macpherson, C. G., Lowry, D. & Carracedo, J. C. Evidence for distinct proportions of subducted oceanic crust and lithosphere in HIMU-type mantle beneath El Hierro and La Palma, Canary Islands. *Geochim. Cosmochim. Acta* **74**, 6565–6589 (2010).

29. Hernandez-Pacheco, A. & Valls, M. C. The historic eruptions of La Palma Island (Canaries). *Arquipélago. Série Ciências da Natureza* (1982).

30. Turner, S. *et al.* 238 U–230 Th–226 Ra disequilibria constraints on the magmatic evolution of the Cumbre Vieja volcanics on La Palma, Canary Islands. *Journal of Petrology* **56**, 1999–2024 (2015).

31. Ibarrola, E. Temporal modification of the basaltic materials from the 1971 eruption of the Teneguía volcano (La Palma, Canary Islands). *Estudios Geológicos* 49–58 (1974).

32. Lundstrom, C. C., Hoernle, K. & Gill, J. U-series disequilibria in volcanic rocks from the Canary Islands: Plume versus lithospheric melting. *Geochim. Cosmochim. Acta* **67**, 4153–4177 (2003).

33. Neave, D. A. *et al.* Clinopyroxene-Liquid Equilibria and Geothermobarometry in Natural and Experimental Tholeiites: The 2014-2015 Holuhraun Eruption, Iceland. *Journal of Petrology* **60**, 1653–1680 (2019).

34. Putirka, K. D., Mikaelian, H., Ryerson, F. & Shaw, H. New clinopyroxene-liquid thermobarometers for mafic, evolved, and volatile-bearing lava compositions, with applications to lavas from Tibet and the Snake River Plain, Idaho. *American Minerologist* **88**, 1542–1554 (2003).

35. Mollo, S. *et al.* An integrated P-T-H2O-lattice strain model to quantify the role of clinopyroxene fractionation on REE+Y and HFSE patterns of mafic alkaline magmas: Application to eruptions at Mt. Etna. *Earth-Science Reviews* vol. 185 32–56 Preprint at https://doi.org/10.1016/j.earscirev.2018.05.014 (2018).

36. Neave, D. A. & Putirka, K. D. A new clinopyroxene-liquid barometer , and implications for magma storage pressures under Icelandic rift zones. *American Mineralogist* **102**, 777–794 (2017).

37. D'Auria, L. *et al.* Rapid magma ascent beneath La Palma revealed by seismic tomography. *Sci. Rep.* **12**, (2022).

38. Ágreda-López, M. *et al.* Enhancing machine learning thermobarometry for clinopyroxene-bearing magmas. *Comput. Geosci.* **193**, (2024).

39. MacDonald, A., Ubide, T., Mollo, S., Pontesilli, A. & Masotta, M. The Influence of Undercooling and Sector Zoning on Clinopyroxene-Melt Equilibrium and Thermobarometry. *Journal of Petrology* **64**, 1–18 (2023).

40. Riel, N., Kaus, B. J. P., Green, E. C. R. & Berlie, N. MAGEMin, an Efficient Gibbs Energy Minimizer: Application to Igneous Systems. *Geochemistry, Geophysics, Geosystems* **23**, (2022).

41. Holland, T. J. B., Green, E. C. R. & Powell, R. Melting of peridotites through to granites: A simple thermodynamic model in the system KNCFMASHTOCr. *Journal of Petrology* **59**, 881–900 (2018).

42. Green, E. C. R., Holland, T. J. B., Powell, R., Weller, D. J. & Riel, N. Erratum: Melting of Peridotites through to Granites: a Simple Thermodynamic Model in the System KNCFMASHTOCr, and, a

Thermodynamic Model for the Subsolidus Evolution and Melting of Peridotite (Journal of Petrology (2018) 59:5 (881-900) DOI: 10.1093/petrology/egy048). *Journal of Petrology* vol. 66 Preprint at https://doi.org/10.1093/petrology/egae079 (2025).

43. Adam, J. & Green, T. Trace element partitioning between mica-and amphibole-bearing garnet lherzolite and hydrous basanitic melt: 1. Experimental results and the investigation of controls on partitioning behaviour. *Contributions to Mineralogy and Petrology* **152**, 1–17 (2006).

44. Schnetzler, C. C. & Philpotts, J. A. Partition coefficients of rare-earth elements between igneous matrix material and rock-forming mineral phenocrysts—II. *Geochim. Cosmochim. Acta* **34**, 331–340 (1970).

45. Villemant, B., Jaffrezic, H., Joron, J.-L. & Treuil, M. Distribution coefficients of major and trace elements; fractional crystallization in the alkali basalt series of Chaine des Puys (Massif Central, France). *Geochim. Cosmochim. Acta* **45**, 1997–2016 (1981).

46. McKenzie, D. A. N. & O'nions, R. K. Partial melt distributions from inversion of rare earth element concentrations. *Journal of petrology* **32**, 1021–1091 (1991).

47. Elkins, L. J., Gaetani, G. A. & Sims, K. W. W. Partitioning of U and Th during garnet pyroxenite partial melting: Constraints on the source of alkaline ocean island basalts. *Earth Planet. Sci. Lett.* **265**, 270–286 (2008).

48. Zack, T. & Brumm, R. Ilmenite/liquid partition coefficients of 26 trace elements determined through ilmenite/clinopyroxene partitioning in garnet pyroxenites. in *International Kimberlite Conference: Extended Abstracts* vol. 7 986–988 (1998).

49. Nielsen, R. L., Gallahan, W. E. & Newberger, F. Experimentally determined mineral-melt partition coefficients for Sc, Y and REE for olivine, orthopyroxene, pigeonite, magnetite and ilmenite. *Contributions to Mineralogy and Petrology* **110**, 488–499 (1992).

50. Larsen, L. M. Distribution of REE and other trace elements between phenocrysts and peralkaline undersaturated magmas, exemplified by rocks from the Gardar igneous province, south Greenland. *Lithos* **12**, 303–315 (1979).

51. Larsen, M. L. Clinopyroxenes and Coexisting Mafic Minerals from the Alkaline Dimaussaq Intrusion, South Greenland. **17**, 258–290 (1976).

52. Simpson, B., Ubide, T. & Spandler, C. Drivers of critical metal enrichment in peralkaline magmas recorded by clinopyroxene zoning. *Commun. Earth Environ.* **6**, (2025).

53. DIGIS Team. 2024-12-SGFTFN_CLINOPYROXENES.csv. *GEOROC Compilation: Minerals* Preprint at https://doi.org/doi/10.25625/SGFTFN/KPTLIE (2024).

54. Beloša, L. *et al.* Magmatic evolution and architecture of an oceanic intraplate volcano: Vesteris Seamount, Atlantic Ocean. *Front. Earth Sci. (Lausanne).* **12**, (2024).

55. Wei, X., Xu, Y. G., Zhang, Y., Yan, Q. S. & Shi, X. F. Deciphering magma plumbing processes beneath Pako seamount in the West Pacific: constraints from textural and compositional diversities of clinopyroxene phenocrysts. *Lithos* **514–515**, (2025).

56. Peretyazhko, I. S. & Savina, E. A. Chemistry and Crystallization Conditions of Minerals in Metasomatized Oceanic Lithosphere and Basaltic Rocks of Govorov Guyot, Magellan Seamounts, Pacific Ocean. *Minerals* **12**, (2022).

57. Wei, X., Zhang, Y., Shi, X. F., Castillo, P. R. & Xu, Y. G. Concurrent magma mixing and crystallization processes revealed by clinopyroxene macrocrysts from Lamont guyot lavas in NW Pacific. *Lithos* **428–429**, (2022).

58. Smith, W. H. F., Staudigel, H., Watts, A. B. & Pringle, M. S. The Magellan Seamounts: Early Cretaceous record of the South Pacific isotopic and thermal anomaly. *J. Geophys. Res. Solid Earth* **94**, 10501–10523 (1989).

59. Dorais, M. J. Exploring the mineralogical heterogeneities of the Louisville Seamount Trail. *Geochemistry, Geophysics, Geosystems* **16**, 2884–2899 (2015).

60. Hawkins, J. W., Lonsdale, P. F. & Batiza, R. Petrologic evolution of the Louisville seamount chain. *Seamounts, islands, and atolls* **43**, 235–254 (1987).

61. Dorais, M. J. & Buchs, D. M. Mineralogical characterization of rejuvenated magmatism at Burton Guyot, Louisville Seamount trail. *Contributions to Mineralogy and Petrology* **174**, 66 (2019).

62. Sandoval-Velasquez, A. *et al.* The noble gas signature of the 2021 Tajogaite eruption (La Palma, Canary Islands). *Journal of Volcanology and Geothermal Research* **443**, (2023).

63. Klügel, A., Hansteen, T. H. & Galipp, K. Magma storage and underplating beneath Cumbre Vieja volcano, La Palma (Canary Islands). *Earth Planet. Sci. Lett.* **236**, 211–226 (2005).

64. Brändle, J. L. MINERALOGY OF THE MATERIALS FROM TENEGUIA VOLCANO, LA PALMA, CANARY ISLANDS. (1974).

65. Lopez Ruiz, J. The pyroxenes of the alkali series rocks: the case of the pyroxenes of the Teneguia Volcano, La Palma, Canary Islands. *Boletín Geológico y Minero* 56–60 (1973).

66. Galipp, K., Klügel, A. & Hansteen, T. H. Changing depths of magma fractionation and stagnation during the evolution of an oceanic island volcano: La Palma (Canary Islands). *Journal of Volcanology and Geothermal Research* **155**, 285–306 (2006).

67. Martí, J. *et al.* Correlation of magma evolution and geophysical monitoring during the 2011-2012 El Hierro (Canary Islands) submarine eruption. *Journal of Petrology* **54**, 1349–1373 (2013).

68. Prieto-Torrell, C. *et al.* Mush system heterogeneities control magma composition and eruptive style on the Ocean Island of El Hierro, Canary Islands. *Contributions to Mineralogy and Petrology* **180**, (2025).

69. Longpré, M. A., Klügel, A., Diehl, A. & Stix, J. Mixing in mantle magma reservoirs prior to and during the 2011-2012 eruption at El Hierro, Canary Islands. *Geology* **42**, 315–318 (2014).

70. Sigmarsson, O. *et al.* Formation of U-depleted rhyolite from a basanite at El Hierro, Canary Islands. *Contributions to Mineralogy and Petrology* **165**, 601–622 (2013).

71. Del Moro, S. *et al.* Xenopumice erupted on 15 October 2011 offshore of El Hierro (Canary Islands): a subvolcanic snapshot of magmatic, hydrothermal and pyrometamorphic processes. *Bull. Volcanol.* **77**, 53 (2015).

72. Nazzareni, S., Barbarossa, V., Skogby, H., Zanon, V. & Petrelli, M. Magma water content of Pico Volcano (Azores Islands, Portugal): a clinopyroxene perspective. *Contributions to Mineralogy and Petrology* **175**, 87 (2020).

73. Zanon, V. & Frezzotti, M. L. Magma storage and ascent conditions beneath Pico and Faial islands (Azores archipelago): a study on fluid inclusions. *Geochemistry, Geophysics, Geosystems* **14**, 3494–3514 (2013).

74. Berthod, C. *et al.* The 2018-ongoing Mayotte submarine eruption: Magma migration imaged by petrological monitoring. *Earth Planet. Sci. Lett.* **571**, (2021).

75. Spath, A., Roex, A. P. & Duncan, R. A. The geochemistry of lavas from the gomores archipelago, Western Indian Ocean: petrogenesis and mantle source region characteristics. *Journal of Petrology* **37**, 961 (1996).

76. Stock, M. J. *et al.* Integrated Petrological and Geophysical Constraints on Magma System Architecture in the Western Galápagos Archipelago: Insights From Wolf Volcano. *Geochemistry, Geophysics, Geosystems* **19**, 4722–4743 (2018).

77. Nusbaum, R. L., Colgan, M. W., Lawton, D. E. & Glascock, M. D. Mineralogic constraints on the magmatic history of Volcán Darwin flank lava at Urvina Bay, Islá Isabela, Galápagos Islands. *Journal of volcanology and geothermal research* **47**, 359–366 (1991).

78. Reynolds, R. W. & Geist, D. J. Petrology of lavas from Sierra Negra volcano, Isabela Island, Galapagos archipelago. *Oceanographic Literature Review* **7**, 681 (1996).

79. Terry Richard Naumann. Geology and petrology of Cerro Azul volcano, Isabela Island, Galapagos Archipelago. (University of Idaho, 1998).

80. Lo Forte, F. M., Aiuppa, A., Rotolo, S. G. & Zanon, V. Temporal evolution of the Fogo Volcano magma storage system (Cape Verde Archipelago): a fluid inclusions perspective. *Journal of Volcanology and Geothermal Research* **433**, 107730 (2023).

81. Mata, J. *et al.* The 2014–15 eruption and the short-term geochemical evolution of the Fogo volcano (Cape Verde): Evidence for small-scale mantle heterogeneity. *Lithos* **288–289**, 91–107 (2017).

82. Hildner, E., Klügel, A. & Hansteen, T. H. Barometry of lavas from the 1951 eruption of Fogo, Cape Verde Islands: Implications for historic and prehistoric magma plumbing systems. *Journal of Volcanology and Geothermal Research* **217**, 73–90 (2012).

83. Klügel, A., Day, S., Schmid, M. & Faria, B. Magma Plumbing During the 2014–2015 Eruption of Fogo (Cape Verde Islands). *Front. Earth Sci. (Lausanne).* **8**, (2020).

84. Albert, H., Costa, F. & Martí, J. Timing of magmatic processes and unrest associated with mafic historical monogenetic eruptions in Tenerife Island. *Journal of Petrology* **56**, 1945–1966 (2015).

85. Di Roberto, A. *et al.* The forgotten eruption: the basaltic scoria cone of Montaña Grande, Tenerife. *Journal of Volcanology and Geothermal Research* **401**, 106918 (2020).

86. Di Roberto, A., Bertagnini, A., Del Carlo, P., Meletlidis, S. & Pompilio, M. The 1909 Chinyero eruption on Tenerife (Canary Islands): insights from historical accounts, and tephrostratigraphic and geochemical data. *Bull. Volcanol.* **78**, 88 (2016).

87. Scott, P. W. Crystallization trends of pyroxenes from the alkaline volcanic rocks of Tenerife, Canary Islands. *Mineral. Mag.* **40**, 805–816 (1976).

88. Neumann, E.-R. *et al.* Evidence for fractional crystallization of periodically refilled magma chambers in Tenerife, Canary Islands. *Journal of Petrology* **40**, 1089–1123 (1999).

89. Davila-Harris, P., Ellis, B. S., Branney, M. J. & Carrasco-Núñez, G. Lithostratigraphic analysis and geochemistry of a vitric spatter-bearing ignimbrite: the Quaternary Adeje Formation, Cañadas volcano, Tenerife. *Bull. Volcanol.* **75**, 722 (2013).

90. Olin, P. H. & Wolff, J. A. Rare earth and high field strength element partitioning between iron-rich clinopyroxenes and felsic liquids. *Contributions to Mineralogy and Petrology* **160**, 761–775 (2010).

91. Canhimbue, L. S., Mezhelovskaya, S. V & Erofeeva, K. G. Petrological features of picrobasaltic melts on Lanzarote, Canary Islands. in *E3S Web of Conferences* vol. 266 07006 (EDP Sciences, 2021).

92. Gómez-Ulla, A., Sigmarsson, O., Huertas, M. J., Devidal, J.-L. & Ancochea, E. The historical basanite-alkali basalt-tholeiite suite at Lanzarote, Canary Islands: Carbonated melts of heterogeneous mantle source? *Chem. Geol.* **494**, 56–68 (2018).

93. Asavin, A. M., Kogarko, L. N., Kryuchkova, O. I., Tyurin, D. A. & Kolesov, G. M. Grand Canary, Saint Helena, and Tristan da Cunha Oceanic Islands: variations of trace element partition coefficients in pyroxene-melt equilibria during alkaline magma evolution. *Geochemistry International* **35**, 415–423 (1997).

94. Aulinas, M. *et al.* The Plio-Quaternary magmatic feeding system beneath Gran Canaria (Canary Islands, Spain): constraints from thermobarometric studies. *J. Geol. Soc. London.* **167**, 785–801 (2010).

95. Crisp, J. A. & Spera, F. J. Pyroclastic flows and lavas of the Mogan and Fataga formations, Tejeda Volcano, Gran Canaria, Canary Islands: mineral chemistry, intensive parameters, and magma chamber evolution. *Contributions to Mineralogy and Petrology* **96**, 503–518 (1987).

96. Mangas, J., Pereztorrado, F., Massare, D. & Clocchiatti, R. Phonolitic origin of Roque-Nublo ignimbrites of Gran-Canaria (Canary-Islands, Spain) from clinopyroxene melt inclusion studies. *European journal of mineralogy* (1993).

97. Cortes-Calderon, E. A. *et al.* Generation and field relations of low-δ18O silica-Undersaturated and mildly saturated alkaline magmas: a case study from the Fataga group, gran Canaria. *Journal of Petrology* **63**, egac090 (2022).

98. Marques, A. F. A., Madureira, P., Zajacz, Z., Hu, S. & Ribeiro, L. P. Deep sourced magma and ore-metal mobility in the D. João de Castro submarine volcano (Azores): a mineral chemistry and melt inclusion study. *Contributions to Mineralogy and Petrology* **177**, 100 (2022).

99. Jeffery, A. J. *et al.* Petrogenesis of the peralkaline ignimbrites of Terceira, Azores. *Journal of Petrology* **58**, 2365–2402 (2017).

100. Pimentel, A. *et al.* Stress-induced comenditic trachyte effusion triggered by trachybasalt intrusion: multidisciplinary study of the AD 1761 eruption at Terceira Island (Azores). *Bull. Volcanol.* **78**, 22 (2016).

101. Madureira, P., Mata, J., Mattielli, N., Queiroz, G. & Silva, P. Mantle source heterogeneity, magma generation and magmatic evolution at Terceira Island (Azores archipelago): constraints from elemental and isotopic (Sr, Nd, Hf, and Pb) data. *Lithos* **126**, 402–418 (2011).

102. Laeger, K. *et al.* Pre-eruptive conditions and triggering mechanism of the~ 16 ka Santa Bárbara explosive eruption of Sete Cidades Volcano (São Miguel, Azores). *Contributions to Mineralogy and Petrology* **174**, 11 (2019).

103. Renzulli, A. & Santi, P. Two-stage fractionation history of the alkali basalt-trachyte series of Sete Cidades volcano (São Miguel Island, Azores). *European Journal of Mineralogy* **12**, 469–494 (2000).

104. Zaccarini, F. *et al.* Mineral Chemistry of Olivine, Oxy-Spinel, and Clinopyroxene in Lavas and Xenoliths from the Canary, Azores, and Cape Verde Islands (Macaronesia, North Atlantic Ocean): New Data and Comparisons with the Literature. *Minerals* **14**, (2024).

105. Costa, S. *et al.* A data driven approach to mineral chemistry unveils magmatic processes associated with long-lasting, low-intensity volcanic activity. *Sci. Rep.* **13**, 1–15 (2023).

106. Rykov, A., De Amorim, R. C., Makarenkov, V. & Mirkin, B. Inertia-Based Indices to Determine the Number of Clusters in K-Means: An Experimental Evaluation. *IEEE Access* **12**, 11761–11773 (2024).

107. Caracciolo, A. *et al. Supplementary Material for the Article 'Recurrent Evacuation of Mantle Mush in Ocean Islands Revealed by Clinopyroxene from La Palma'*. (2026). doi:10.5281/zenodo.21102987.
