# Supplementary material for: Recurrent evacuation of mantle mush in ocean islands revealed by clinopyroxene from La Palma
Source: Nat Commun. 2026 Sep 24;17:9362. doi: 10.1038/s41467-026-77213-9 (PMC13612566; doi:10.1038/s41467-026-77213-9)
Supplement: Supplementary file 2 [file 41467_2026_77213_MOESM3_ESM.pdf]

nature portfolio

Peer Review File

Recurrent evacuation of mantle mush in ocean islands revealed by clinopyroxene from La Palma

Corresponding Author: Dr Alberto Caracciolo

This file contains all reviewer reports in order by version, followed by all author rebuttals in order by version.

Attachments originally included by the reviewers as part of their assessment can be found at the end of this file.

Version 0:

Reviewer comments:

Reviewer #1

(Remarks to the Author)
This manuscript presents a comprehensive clinopyroxene-based investigation of the magma plumbing system beneath La Palma, integrating a large and diverse dataset that includes petrography, major and trace element geochemistry, high resolution trace-element maps, thermobarometry, and cluster analysis across three historical eruptions (1712, 1971 and 2021). The effort to combine mineral chemistry, spatially resolved datasets and quantitative petrological tools is evident and represents a substantial amount of work. The internal consistency of the clinopyroxene datasets across eruptions is one of the clear strengths of the study, as is the attempt to place the 2021 Tajogaite eruption into a longer-term historical framework.

The authors interpret these data as evidence for a temporally stable, upper-mantle plumbing system hosting an evolved, phonolite-related crystal mush that is repeatedly reactivated by injections of mafic basanite magma. This conceptual framework is internally coherent and broadly consistent with existing petrological and geophysical models proposed for La Palma. However, as currently developed, the manuscript largely consolidates and refines a conceptual model that is already established in the literature rather than clearly extending or challenging it.

While the dataset is rich and well-integrated, the main conclusions remain incremental in nature, and the manuscript would benefit from a more explicit articulation of its conceptual advance. This point is particularly important given the scope of Nature Communications, which typically emphasizes studies with a clear step-change in understanding or a strong methodological advance.

Although the authors repeatedly refer to low-flux OIB volcanoes in general, the manuscript does not convincingly demonstrate how insights from La Palma fundamentally alter our broader understanding of such systems. In addition, the manuscript is very long and, in places, conceptually fragmented between the main text and the Supplementary Material, which makes the narrative difficult to follow and obscures the key messages. For these reasons, in the Reviewer opinion, the current framing does not yet fully align with the expectations of a broad-scope journal such as Nature Communications, although the study addresses an important topic and could find a very appropriate audience in a more specialized journal within the fields of volcanology and igneous petrology.

Major conceptual issues:

1) Throughout the manuscript, clinopyroxene zoning patterns, particularly the occurrence of Cr-rich inner rims and the distribution of clusters, are interpreted as direct evidence for repeated mafic recharge events and progressive evacuation of a crystal mush. While this interpretation is plausible, the causal link between zoning style and specific plumbing-system dynamics is not demonstrated rigorously enough to exclude alternative explanations. For example, variations in compatible element concentrations and Mg# could also arise from local boundary-layer effects, changes in crystal growth rates during undercooling, or variations in melt composition unrelated to discrete recharge events. In addition, the possibility that part of the chemical variability observed in clinopyroxene cores reflects crystallographic effects (e.g. sector versus prism-sector zoning) is not systematically addressed. Given the central role assigned to zoning in the overall interpretation, a more critical evaluation of these alternative mechanisms is necessary to avoid over-interpreting the zoning record.

2) A key implicit assumption of the manuscript is that clinopyroxene zoning preserves a coherent temporal record that can be directly linked to eruption staging, particularly the transition from early tephrite to later basanite. However, no diffusion modelling or kinetic constraints are provided to demonstrate that, for examples, inner rims and outer rims formed within resolvable, eruption-relevant time windows. Without such constraints, it remains unclear whether the zoning patterns record short-term pre-eruptive processes or instead reflect longer-term crystal recycling within a persistent mush system. This ambiguity weakens the argument that the observed zoning can be used to infer eruption priming or the sequence of magmatic processes immediately preceding eruption. Explicitly addressing the timescales represented by the different textural domains would significantly strengthen the manuscript.

3) Although the manuscript presents a large and carefully assembled dataset, many of its main conclusions are already documented for La Palma. The present study refines these ideas rather than substantially modifying them. For the journal's scope, it would be important to clarify what aspects of the proposed model genuinely represent a new conceptual insight. At present, the manuscript would benefit from a clearer statement of whether the novelty lies in the temporal persistence of the plumbing system, the specific role assigned to recharge versus triggering, or the methodological integration itself. Without this clarification, the overall impact of the study remains limited.

4) The authors frequently extrapolate their findings to low-flux OIB volcanoes in general. However, the manuscript does not sufficiently discuss how representative La Palma is in terms of magma supply rate, lithospheric thickness etc. These factors can vary significantly among OIB systems and may strongly influence plumbing-system architecture. As a result, the broader applicability of the proposed model remains uncertain. A more explicit discussion of which aspects of the La Palma system are likely to be general and which are system-specific would improve the manuscript and better justify its relevance for a broad readership.

5) The manuscript argues that mafic recharge plays a key role in priming the system but does not act as the immediate trigger for eruption. While this distinction can be reasonable, it is not consistently maintained throughout the text. In some sections, recharge appears to be discussed in ways that verge on short-term eruption control, whereas in others it is framed strictly as a long-term conditioning process. This lack of conceptual clarity makes it difficult to assess the causal role of recharge in eruption dynamics. A clearer separation between long-term system evolution and short-term eruption initiation would strengthen the overall interpretation.

6) The interpretation of Cluster 4 as a "cryptic primitive seed for the phonolite lineage" is based on extremely limited data. Given its rarity and the lack of clear independent constraints, this interpretation appears speculative. While such hypotheses can be valuable, they should be more explicitly framed as tentative and not given the same weight as interpretations based on more robust datasets. The discussion of Cluster 4 is notably more speculative than that of other clusters, yet this speculative interpretation is still integrated into the main conceptual cartoon and evolutionary framework. This creates an imbalance in the strength of evidence supporting different parts of the proposed model. Alternatively, a reduction or simplification of the clustering scheme might help avoid over-interpretation (see later).

Methodological issues

1) The cluster analysis is central to the manuscript but is described in an overly simplified manner. Key methodological details are missing, including data preprocessing steps if applied (e.g. normalization, scaling), and the criteria used to select the number of clusters. The choice of six clusters is justified primarily by visual inspection, which is insufficient for a study so strongly reliant on clustering. Data-driven approaches such as silhouette analysis, elbow methods or dimensionality-reduction techniques (e.g. PCA) should be used to objectively justify the chosen number of clusters. Applying such methods might even reduce the number of clusters, potentially simplifying the interpretation and eliminating the need for poorly constrained groups such as Cluster 4. In addition, it is not entirely clear whether the clustering is based exclusively on LA-ICP-MS spot analyses or whether chemical map data are also included. From the text and figures, clustering appears to rely on spot analyses only, with chemical maps used primarily for visualization. This distinction is important and should be clarified, as it has implications for spatial coherence and the robustness of the clustering results. If both spot analyses and chemical map data are indeed combined in the clustering workflow, the authors should explicitly demonstrate how datasets characterized by different spatial resolutions, signal integration volumes and data-reduction procedures inherent to laser-based techniques can be made fully comparable within a single clustering framework. Without such justification, the mixing of differently reduced datasets may introduce additional noise into the chemical dataset, potentially biasing cluster structure and subsequent interpretations.

2) The care taken to assess equilibrium conditions is really appreciated. However, the use of different thermobarometric models for different crystal domains appears unnecessary and potentially confusing. The Reviewer is aware about the authors' expertise in global regression-based and machine-learning thermobarometers, and it is unclear why a single, internally consistent approach was not adopted. At the very least, the rationale for excluding such models should be explained more clearly.

3) While no major flaws are evident in the fractional crystallization modelling, the use of thermodynamically based models (e.g. MELTS, alphaMELTS or MCS for major and trace elements) would strengthen the manuscript. Such models would add rigour to the interpretation of the basanite-tephrite transition and reinforce the conclusion that phonolitic melts form after ~80% crystallization.

Minor comments (number of lines as in the PDF)

Lines ~99–106
Please clarify whether compositional variability in clinopyroxene cores could partly reflect sector zoning effects. When describing clinopyroxene cores, the manuscript attributes most of the observed chemical variability to complex magmatic histories involving antecryst recycling and mush processes. However, the potential contribution of crystallographic effects, such as sector versus prism-sector zoning, is not discussed, despite clear evidence of patchy and sector zoning in the presented BSE images. Because sector zoning can generate significant chemical heterogeneity without changes in melt composition, neglecting this possibility risks over-interpreting core compositions in terms of magmatic processes alone.

Line ~119 and throughout
Supplementary figures and tables are not cited in the order in which they appear, making the Supplementary Material difficult to navigate. This disrupts the logical flow of the manuscript and makes it unnecessarily challenging for the reader to follow the progression of the results and interpretations.

Lines ~166–178
Matrix compositions are treated as reliable proxies for equilibrium melts. However, the matrix is described as microcrystalline rather than glassy, implying crystallization during late ascent or emplacement. Although the methodology used here takes advantages from another published work, the manuscript does not sufficiently discuss how raster-based LA-ICP-MS analyses may integrate micro-scale heterogeneity or submicroscopic crystalline phases, potentially biasing melt compositions toward apparent homogeneity. This introduces a conceptual inconsistency when matrix compositions are subsequently used for equilibrium tests and trace-element modelling.

Line 350: Etna should not be classified as a low-flux volcano. This classification is incorrect and undermines the low-flux conceptual framework used elsewhere in the manuscript to contextualize La Palma and other similar systems. The analogy with Etna is used to reinforce interpretations of recharge-driven dynamics. However, given the fundamentally different magma supply rates and plumbing architectures of Etna and La Palma, this comparison weakens rather than strengthens the low-flux OIB narrative developed in the manuscript.

EPMA methods: The inter-instrument comparison of clinopyroxene analyses is appreciated, but the manuscript does not clearly explain how identical analytical spots were located across instruments or how Poisson statistics were applied to quantify inter-instrument reproducibility. Additional clarification would improve transparency and reproducibility.

LA-ICP-MS methods:
Lines 529 and 569: The Zenodo data repository referenced in the manuscript is inaccessible to the Reviewer, and its contents are not clearly described.

The raster method used for groundmass major-element analyses is only briefly mentioned. Although described in Ubide et al. (2023), a short summary would improve the self-contained nature of the manuscript and reduce reliance on external references.

Line 578: More information on the Python script used for data processing should be provided, or the script should be made publicly available.

Reviewer #2

(Remarks to the Author)
Please see attached document.

Reviewer #3

(Remarks to the Author)
This manuscript by Caracciolo et al. uses clinopyroxene zoning to reconstruct how the La Palma (Cumbre Vieja) plumbing system behaved across three eruptions (El Charco 1712, Teneguía 1971, Tajogaite 2021). There dataset comes primarily from the quantification of cpx zonations by LA-ICP-MS spot analyses and trace-element maps, combined with major-element EMPA. They integrate both single-spot trace elements and pixel-based trace-element map data into a large multivariate dataset and apply a clustering analysis (k-means) to identify recurring compositional clusters that are then mapped onto textural positions (cores/inner rims/outer rims/microlites). The results allowed them to interpret the clusters/cpx zones in terms of recycled evolved antecrysts (phonolitic mush), mafic recharge, post-recharge differentiation, and ascent/cooling signatures. They further estimate magma storage depths and temperatures using cpx-melt thermobarometry following the equilibrium/calibration framework of MacDonald et al. (2023), and the paper argues for temporally consistent upper-mantle storage with a cryptic shallow level that does not leave a cpx record.
Overall, the dataset is substantial, well-structured and well-written. The manuscript is clearly motivated by the need to understand plumbing system variability in low-flux basaltic systems, where monitoring/forecasting is a challenge. I found the manuscript interesting, and the use of k-means clustering to classify cpx rim domains from high-resolution trace-element data provides a novel and useful framework for objectively linking zoning patterns to magmatic processes. The data is certainly worthy of publication, although I suggest the significance/broadness of the results is emphasized more to meet the scope of the journal. I also appreciate the wealth of supplementary figures to support the results. Below I have listed my main concerns I would like to see addressed, mainly to ensure the robustness of the conclusions made.

Main comments
1) Clustering analysis. You identify 6 clusters from your analysis. I gather from your supplementary information that these 6 clusters were defined primarily from visual inspection of a map by varying k (2-10). Although I think this is a reasonable approach, I don't find it sufficient for the conclusions that rely on both the distinctiveness and temporal persistence of the cluster groups. Can you provide at least one quantitative basis for k choice, demonstrating that 6 was the best fit (e.g. bootstrapping or others)? It will demonstrate the robustness of your choice.

From what I understood, the clustering analysis of the trace element data was performed on combined spot (n=318) and pixel-based map data (n=3398776). This is a big class imbalance, meaning that the k-means clustering will be dominated by the pixel population. This is important because most petrological inferences comes from textural defined spot analyses and eruption to eruption comparisons. Could you test whether the cluster solutions are still valid when the clustering analysis runs with spots only, and pixels only. You could also downsample pixels per map so pixels and spots are roughly comparable weights.
Right now, it seems like the subjective k choice and pixel dominance needs clearer justification.

In regards the input data for the clustering, is the clusters analysis performed using the raw ppm concetration matrices or from post-processed data (scaled/transformed)? I see the methods section the cpx matrices underwent processing and enhancements. So I would like to see in the clustering methods section what filtering was applied prior to clustering, as this could have a significant effect on the boundary pixels of the different cpx zones.

2) What could be other explanations that the shallow reservoir is cryptic? Maybe cpx isn't the phase to best record this stage? What if the timescales or temperatures may prevent re-equilibration at in the cpx, or shallow storage was dominated by melt + other phases. I find in several places in the manuscript the wording is overly strong, for example line 252 "we do not observe evidence for shallow storage…" could be rephrased to "shallow storage was not recorded in the cpx".
The question on whether the recharge was a trigger vs. priming is certainly an important aspect of this study and what makes it novel and is a strong statement. Could the lack of cluster 5 in the earlier cpx crystals in the inner rim be a consequence of sampling, eruption frequency , or overprinting?

3) The fractional crystallization model is nice and the workflow in the supplementary info is clear. However, despite the mention of the uncertainties with using fixed partition coefficients, there is there a risk that it obscures the P-T-composition dependence? Your uncertainty treatment does address this partly.
In regards your fractional crystallization assemblage, you use the assemblage from Ubide et al. (2023), which excludes amphibole? But in line 305 you explicitly mention the MREE/HREE is partly through amphibole + cpx partitioning. This inconistiency should be addressed, either include amphibole into the FC model or explicitly state that it is not capturing the role of amphibole.

4) Significance of the study needs more emphasis. I think within the volcanology community this study is neat and potential very useful. However, I find the manuscript reads almost like a single volcano study, thus I have some concerns over the broader impact of the study, particularly for its publication in Nat. Comms. For a broader impact, I would recommend clearer statements upfront in the intro that gives a generalizable insight beyond "La Palma has persistent upper mantle storage and a recurrent mush system", which is system specific unless the authors demonstrate transferability to other systems. For example, better connections to hazard/monitoring by stating testable links between cluster-defined processes and geophysical/geochemical monitoring: this is stated in the intro and the very end of the discussion, but I would like to see a better link and more specific connections with your data. Also, do you think magma recharge not being the immediate trigger for an eruption is common at many other volcanoes? As this is a big conclusion of the study, I would like to see clarification which of your finds are likely common of low-flux OIB plumbing systems, and which are La Palma specific.

Specific comments
Line 26: "Interrogating" may be overly strong. Suggest something like "decoding".
Line 30: Be more specific on the type of cluster analysis, e.g., clustering analysis of cpx zoning.
Line 32: phonolitic *crystal* mush. Mush on its own could imply a physical state of something.
Line 37: This is rather interpretive and evidence needs to be watertight to make such a claim.
A more conservative option could be "does not appear to represent the immediate trigger". This feeds in to my main comments 1 & 2.
Line 38-40: This appears to be the main conclusion and novel part of the study. Most, if not all, studies relate magma recharge as the trigger of such eruptions. This is a interesting claim. It begs the question about sample/CPX retrospectivity.
Line 52: Can you expand or add an extra few sentences on the why low-flux means different to high-flux systems? E.g., does longer repose = more crystal mush development?
Line 53: magma plumbing *system*
Line 67: Sentence reads funny. "Magma plumbing" twice. Please rephrase.
Line 68-69: Sentence is fine, but I would consider tightening the citations up rather than clumping them all together.
Consider splitting the citation up in the sentence.
Line 87: Only basanites have olivine? Please clarify.
Line 90-91: Abit of an awkward sentence. Also, this may be confusing to call all phenocrysts "crystals". Why not call them just phenocrysts?
Line 93: inclusions
Line 104: returns *to lighter* BSE.
Line 108: All in mol%?
Line 146: trace elements*

Line 190: Missing closing parentheses
Line 220: thermobarometric*
Line 255-257: Code this shallow zone be recorded by other phases, like olivine diffusion, plagioclase, melt inclusion work?
Line 304: Check figure reference to Fig. 22? Should this be Fig. S22? Or maybe Fig. S21?
Line 347: associated *with*
Line 359: *likely* zoned
Line 426: …eruption *trigger*.
Line 447-452: The monitoring perspective is nice, but this could be expanded to highlight the broader significance of this petrological study.
Line 471: inner rims *show*
Line 506-507: state explicitly the beam size. A 5 um defocused vs a focused (1 um) beam.
Line 538: consisting *of* laser…
Line 545: *assessed*
Line 576-577 and Figure 8 caption: Inconsistency in the data trimming? 1st-99th vs 0.1-99.9? Check.
Line 600-602: The size of the dataset is impressive. Were any of these standardized (z-score) before k-means? Without standardizing elements like Ti and Zr would dominate purely by scale.
Line 649: * Python*
Figure 7: Caption - *Thermobarometry* results
Figure 8: The colors should be explained in the figure caption. I know in this figure that the colors are not important for reading the figure, which is mainly for the use in figure 9. But I would state something in the caption that these colors are used to distinguish each cluster for subsequent figures. In the captions, you state that 6 was chosen as it “best captured” the main textural and chemical features. I would like to see at least one quantitative metric that also supports 6 clusters.

Version 1:

Reviewer comments:

Reviewer #1

(Remarks to the Author)
Second-round review report of Caracciolo et al.

This revised manuscript presents a substantially improved version of the original submission. The authors have clearly engaged with the main concerns raised during the first round of review and have made significant efforts to strengthen the methodological transparency, broaden the conceptual framing, and clarify the role of mafic recharge in the La Palma plumbing system.

In particular, the revised manuscript now better articulates its broader relevance to low-flux OIB systems, provides a more detailed description of the clustering workflow, introduces additional quantitative support for the choice of six clusters, adds thermodynamic fractional crystallization modelling, and includes first-order crystallization timescale estimates for clinopyroxene rims.

Overall, the revision represents a meaningful improvement. Several of the major concerns raised previously have been addressed satisfactorily, especially those related to clustering methodology, preprocessing, cluster-number selection, and the broader significance beyond La Palma.

The original version of the manuscript presented a rich clinopyroxene dataset from three historical eruptions of Cumbre Vieja. The study risked appearing as a refinement of an already established model: upper-mantle magma storage, tephritic-to-basanitic evolution, evolved mush recycling, and recharge-driven remobilization had already been documented or proposed in previous studies of La Palma. The revised manuscript is more ambitious. The authors now frame the study around recurrent evacuation of evolved mantle mushes in ocean island systems more broadly. They also attempt to link the La Palma observations to a global OIB context using a compiled clinopyroxene database and PCA. This is a useful and really appreciated addition and helps justify the relevance of the work beyond the local case study.

The most important improvement concerns the clustering methodology. The first version did not sufficiently describe preprocessing, scaling, number-of-cluster selection, or whether single-spot analyses and pixel-level map data were combined. The revised version now states that the clustering dataset combines single-spot analyses and quantified pixels from compositional maps, that trace elements were log-transformed, standardized, and filtered for outliers, and that k=6 was selected using a combination of PCA, t-SNE, elbow method, and textural/geological inspection. This is a substantial improvement in transparency.

In my view, the manuscript is now closer to being suitable for publication, but a few points still require clarification before final acceptance. The revised manuscript is significantly stronger than the original submission. The authors have responded constructively to the review and have made substantial improvements. With modest additional clarification, the study would be suitable for publication.

Major comments:

- Conceptual advance and suitability for a broad-scope journal:

The revised manuscript now more clearly argues that the La Palma dataset reveals a recurring process potentially relevant to low-flux OIB systems worldwide: the storage of evolved tephri-phonolitic to phonolitic mushes in the upper mantle and their repeated recycling or evacuation by mafic recharge. The new global OIB comparison helps strengthen this argument and moves the manuscript beyond a purely local reconstruction. However, the broader generalization still depends strongly on whether the compiled clinopyroxene PCA genuinely captures comparable processes across different OIB systems. The manuscript should be careful not to imply that similar PCA trends necessarily require identical plumbing-system dynamics. Similar major-element trends in clinopyroxene can reflect related but not identical processes, including differentiation, melt composition, sector zoning, and crystallization kinetics. The global comparison is valuable, but it should be presented as supportive and hypothesis-generating rather than definitive evidence that the La Palma mechanism operates universally. The authors should maintain cautious wording when extrapolating to OIB systems globally.

- Clustering methodology and number of clusters:

This issue has been substantially improved. The revised manuscript now provide a much clearer description of the clustering workflow. The use of log transformation, standardization, outlier filtering, PCA, t-SNE, and elbow analysis is appropriate. The authors also correctly note that there is no uniquely correct number of clusters in unsupervised learning and that cluster-number selection should combine statistical diagnostics with geological and textural interpretability.
The six-cluster solution is now defensible. It is not necessarily uniquely correct, but it is methodologically justified. The combination of elbow analysis and textural coherence supports k=6 as a reasonable partition. The authors' argument that fewer clusters might obscure meaningful zoning domains is plausible.
However, I would avoid stating that k=6 is objectively "the" correct solution. It is better described as the most interpretable and geologically useful solution among the tested alternatives. This distinction matters because the clustering is not merely descriptive; it underpins several genetic interpretations. In particular, the preservation of Cluster 4 depends on the chosen partitioning. The authors should therefore avoid overstating the objectivity of k=6.
Furthermore, there are still aspects that should be improved. The Results section should explicitly report the main clustering results rather than leaving them implicit. Moreover, because clustering represents one of the central methodological advances of this study, its methodology should receive substantially greater emphasis in the main manuscript instead of being largely confined to the Supplementary Material. At present, the Methods section gives considerably more weight to thermobarometry than to clustering. In my opinion, the clustering workflow, including PCA and t-SNE analyses and their implementation, should be described in sufficient detail within the main manuscript.

- Thermobarometry:

The revised manuscript improves the thermobarometric framework by incorporating both classical clinopyroxene–liquid thermobarometry and the recently developed ML approach of Ágreda-López et al. (2024). This addition addresses the previous concern regarding the use of complementary thermobarometric methods and provides an interesting comparison between conventional and ML-based estimates of magma storage conditions.
However, one aspect remains unclear to me. The ML thermobarometers are ultimately trained on large compilations of experimental clinopyroxene datasets (following Jorgenson et al., 2022, on which Ágreda-López et al. 2024 is based), which substantially overlap with the experimental datasets that underpin many of the used clinopyroxene–liquid calibrations (e.g., Putirka, 2003; Putirka, 2008 etc..). Consequently, although the regression methodologies differ (ML vs. empirical exchange equations), the underlying calibration space is not entirely independent.
For this reason, I am not fully convinced that the agreement between the ML and exchange-based thermobarometers should be interpreted as an independent validation of the inferred P–T conditions. Rather, it may represent convergence between different predictive approaches trained on overlapping experimental constraints.
What additional information is gained by presenting both approaches if they are ultimately constrained by substantially overlapping experimental calibration datasets?
I encourage the authors to discuss this point explicitly and clarify the rationale for presenting both approaches together. A brief discussion of their degree of independence would strengthen the methodological framework of the study and help readers better appreciate the added value of the ML-based thermobarometric results.

- Supplementary codes and reproducibility:

The authors tried to improve reproducibility by providing the Jupyter code used for clustering, and thermobarometric calculations. However, the way the code is supplied is not optimal. The notebooks appear to be provided as raw text files rather than directly usable .ipynb notebooks or a structured code repository.
The files also contain executed outputs, hard-coded paths etc. This makes them bulky and difficult to inspect. The documentation is minimal. For example, the clustering notebook does not provide a complete step-by-step workflow. The notebooks are still hard-coded and do not function as a general reproducible pipeline. A better practice would be to provide: (1) clean .ipynb notebooks with cleared outputs, (2) equivalent .py scripts for batch execution, (3) a README.md or a .txt explaining the workflow. Ideally, these should be deposited as a version-controlled GitHub or Zenodo repository rather than embedded as large text supplements.

Reviewer #3

(Remarks to the Author)

I have read the revised manuscript and the authors rebuttal. Overall the paper is clearly improved, and I think most of my main concerns from the first round are adresssed now. In particular, the clustering workflow is explained much clearer, the choice of k is now backed up with quantitative tests, and they also provide the code/scripts, which makes the analysis alot more transparent and reproducable. I also appreciate the effort to strengthen the broader relevance beyond La Palma, which helps the manuscript fit better within the scope of Nat Comms. The discussion around the “cryptic” shallow storage also reads more balanced now.
I do have one remaining (minor) concern. Even though it is reassuring that the spot data and the pixel data overlap in PCA space, the k-means clustering is still based on a dataset that is dominated by map pixels compared to the much smaller number of spot analyses. A short stability test (for example running k-means using only the spots, or rerunning it on a downsampled pixel dataset with similar weight to the spots) would directly show that the cluster solution and the interpretations tied to the clusters are not sensitive to this pixel:spot imbalance.
Overall, I think the revision is much stronger, and the remaining points should be straightforward to address.
Paul A. Wallace, LMU Munich

Version 2:

Reviewer comments:

Reviewer #1

(Remarks to the Author)
I read the manuscript again with great interest after the second round of revisions. I have no further comments.
All of the comments and suggested revisions have been thoroughly addressed. The manuscript is now truly strong in every aspect and undoubtedly deserves publication in Nature Communications. I sincerely appreciated the opportunity to review this work.

Congratulations to the authors on an excellent study.

Simone Costa

Reviewer #3

(Remarks to the Author)
I have reviewed the latest revision and the authors response. The authors have now addressed my remaining concerns by repeating the clustering using a balanced, downsampled dataset and demonstrating that the cluster solution is not being driven by the much larger number of map pixels. This analysis is clearly presented in the revised methods and supplementary information. For both rounds of review, I am now satisfied that my concerns have been addressed and have no further comments. I therefore support publication of the manuscript in its present form.
Sincerely,
Paul A. Wallace, LMU Munich

Open Access This Peer Review File is licensed under a Creative Commons Attribution 4.0 International License, which permits use, sharing, adaptation, distribution and reproduction in any medium or format, as long as you give appropriate credit to the original author(s) and the source, provide a link to the Creative Commons license, and indicate if changes were made.
In cases where reviewers are anonymous, credit should be given to 'Anonymous Referee' and the source.
The images or other third party material in this Peer Review File are included in the article's Creative Commons license, unless indicated otherwise in a credit line to the material. If material is not included in the article's Creative Commons license and your intended use is not permitted by statutory regulation or exceeds the permitted use, you will need to obtain permission directly from the copyright holder.
To view a copy of this license, visit https://creativecommons.org/licenses/by/4.0/

Rebuttal letter

Point-by-point response to the reviewers' comments

Manuscript NCOMMS-25-099451

<u>Clustering methods</u>

We thank the reviewer for raising concerns about the clustering method and we apologize that the clustering methodology was not sufficiently explained in detail. Issues from both reviewers 1 and 3 include (1) the pre-processing of the dataset, (2) the selection of the appropriate cluster number and (3) the merging of single-spot data with pixel-level data from chemical maps.

(1) **<u>Pre-processing of the dataset</u>**. The clustering dataset consists of a single matrix combining single-spot analyses (n = 319) and quantified pixels from compositional maps (n = 398,776). Trace element concentrations (Sc, Ti, V, Cr, Mn, Ni, Sr, and Zr) were log-transformed and subsequently standardized by subtracting the mean and scaling to unit variance [i.e., using the StandardScaler function in scikit-learn, version 1.7.2] before clustering to ensure comparability across elements and to prevent variables with large absolute concentrations (e.g., Ti, Zr) from dominating Euclidean distances. Values outside the 0.1–99.9 percentile range were excluded to remove analytical outliers. These preprocessing steps are now fully described in the revised Methods section.

(2) **<u>Selection of cluster number.</u>** Cluster analysis is an unsupervised learning approach, and there is no a priori "correct" number of clusters for a given dataset, nor a universally accepted method that uniquely identifies the optimal cluster number. Instead, the choice of cluster number is typically guided by a combination of qualitative visual inspection methods, quantitative approaches, and geological or textural constraints (Musu et al., 2023; Templ et al., 2008). We evaluated the principal component analysis (PCA) and the T-distributed stochastic neighbor embedding (t-SNE) as visual approaches and the elbow method (Rykov et al., 2024) as a quantitative technique to assess the appropriate number of clusters (k). We evaluated solutions from k = 2 to 10. Projection of cluster assignments in PCA and t-SNE spaces confirms that clusters correspond to well-defined neighborhoods rather than arbitrary subdivisions of a continuous gradient when using k=6. The application of the elbow method using the total within-cluster sum of squares (WSS; e.g., Costa et al. 2023) further confirms k=6 as an optimal number of clusters. Indeed, the relative decrease in the WSS curve indicates that the marginal gain obtained by adding additional clusters drops sharply after k=6. This identifies six clusters as the highest-resolution partitioning that yields substantial improvements in cluster compactness, with further

subdivision providing only diminishing returns (see figure below, which is now included in the supplement as Fig. S24).

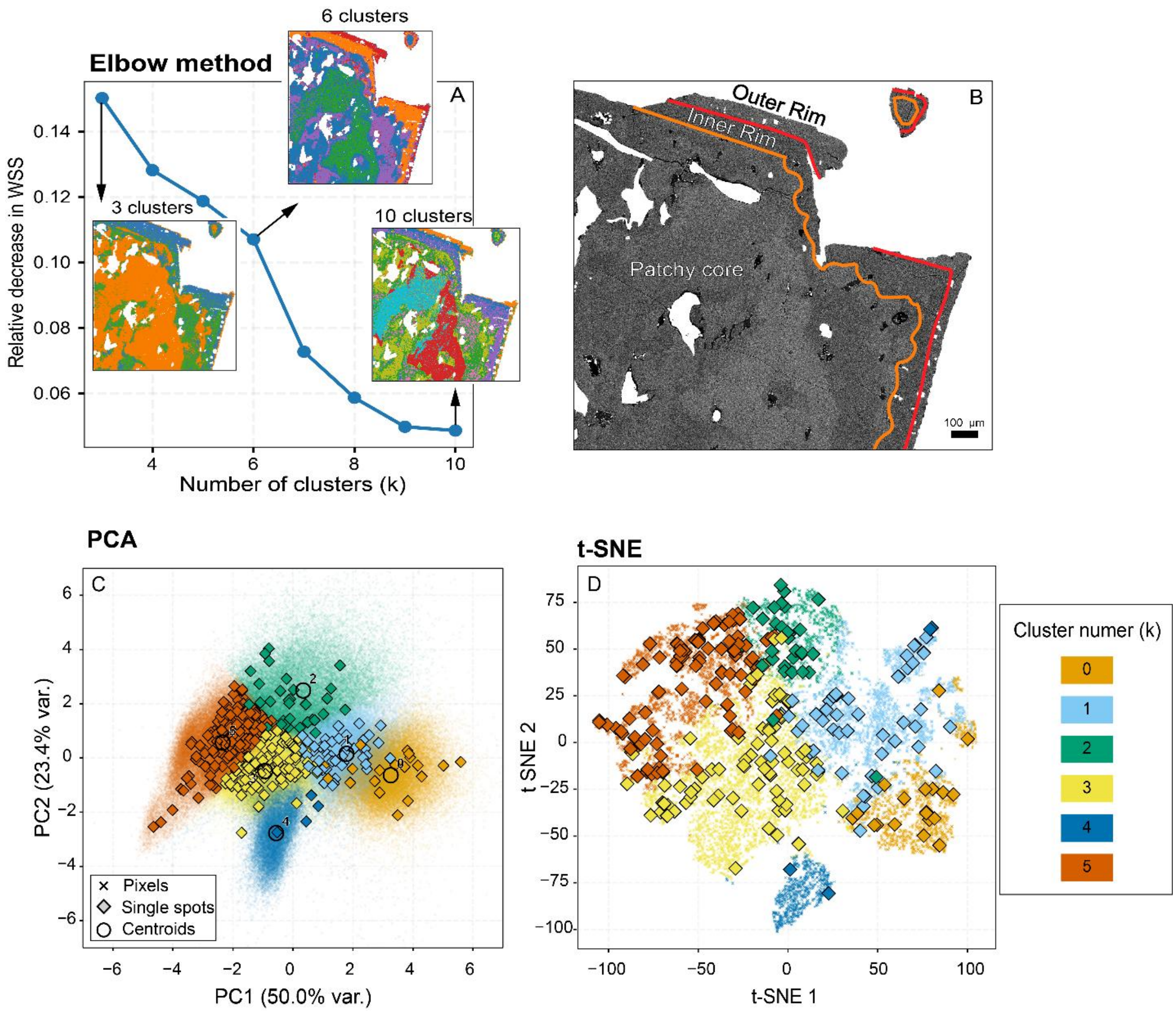

Importantly, the selection of cluster number in geochemical datasets should not rely solely on statistical criteria but must also consider the characteristics of the system under investigation (Musu et al., 2023; Templ et al., 2008). In our case, strong textural constraints exist, and clustering is expected to reflect texturally defined domains within crystals, including cores and their patchy regions, and concentric zoning such as inner and outer rims. For this reason, we systematically inspected cluster maps generated using cluster numbers ranging from 2 to 10 (Ágreda-López et al., 2025).

To summarize, by combining quantitative clustering approaches with visual inspection of cluster distributions in chemical maps, we selected a six-cluster configuration as it provides the most consistent and interpretable partitioning of the data from both

chemical and textural perspectives. These analyses are now included in supplementary figure S3. The Jupyter code used for clustering and evaluation of quantitative metrics is now provided as supplementary material.

(3) We clarify that clustering was performed on a combined dataset including both spot analyses and pixel-based map data. We agree that the very different number of spot analyses and pixel-based map data requires explicit clarification. However, we would like to note that spot and pixel-based map data do not represent different classes in the clustering analysis. Rather, they are complementary types of observations of the same crystal-chemical variability, each contributing different information to the dataset. Spot analyses were primarily included since they can provide a broader trace-element coverage compared with the maps, that are limited to a few elements. Maps, on the contrary, have the advantage of providing high-resolution spatial coverage of compositional domains.

To address the reviewers' concerns on the influence of using both data types for clustering, we projected both single spots and pixels into PCA and t-SNE spaces after clustering (See figure 2 c-d). These plots show that single-spot analyses occupy the same compositional regions as pixel data rather than forming distinct or isolated groups. This indicates that clustering reflects intrinsic geochemical populations rather than artefacts arising from differences in sampling density.

We now report all the relevant information about the clustering approach in the method section and supplementary material of the revised version of the manuscript.

Crystallization timescales of clinopyroxene rims

Two reviewers raised concerns that we did not constrain the timescales associated with the formation of inner and outer clinopyroxene rims, leaving it unclear whether these zones record short-lived pre-eruptive processes or longer-term crystal residence within a mush system. We thank the reviewers for this constructive suggestion, which prompted us to explicitly quantify the timescales of rim formation, which we did in a similar way as in Ubide and Kamber (2018).

To estimate timescales, we first measured rim thicknesses on backscattered electron images using ImageJ (Abràmoff et al., 2004). Measurements were performed on 20 clinopyroxenes per eruption. Inner rim thicknesses are consistent across eruptions, with mean values of 48 ± 10 µm (2021), 41 ± 8 µm (1971), and 48 ± 8 µm (1712). Outer rims are thinner, with average thicknesses of 11 ± 3 µm (2021), 13 ± 3 µm (1971), and 24 ± 7 µm (1712). Thus, inner rim thicknesses are broadly comparable across eruptions, whereas outer rims are slightly thicker in clinopyroxenes from El Charco 1712.

We then converted rim thickness into crystallization timescales using published clinopyroxene growth rates for mafic alkaline systems. Although no experimental growth-rate data are available specifically for La Palma magmas, experimentally constrained clinopyroxene growth rates from Mt. Etna provide a valid analogue for basaltic alkaline compositions. Under low undercooling conditions, clinopyroxene growth rates are typically on the order of 10^{-8} cm/s (Armienti et al., 2013; Orlando et al., 2008). Using this growth rate, the calculated formation times for inner rims are approximately 4–7 days across all eruptions, whereas outer rims formed within approximately 1–3 days.

Overall, our calculations indicate that inner and outer rims formed over the minimum timescales of days, and likely less than ~10 days in total, from the onset of recharge-related crystallization to eruption. However, it remains unclear whether outer rim growth followed immediately or was preceded by a period of near-equilibrium storage after inner rim crystallization. Nonetheless, inner rims and outer rims formed within a relatively short period of time and are consistent with the interpretation that they record pre-eruptive magmatic processes rather than prolonged crystal recycling within a mush system. These calculations have now been implemented in the discussion of the paper.

Broader implications beyond La Palma.

We thank the reviewers for highlighting the need to better articulate the broader significance and conceptual advance of our study. We agree that, in its original form, the manuscript primarily emphasized the La Palma dataset without sufficiently demonstrating how these findings may extend to low-flux OIB systems more generally.

In response, we have revised the Discussion to strengthen the global implications of our results. In particular, we now place the Cumbre Vieja observations within a worldwide OIB framework by compiling and analyzing clinopyroxene major element datasets from multiple low-flux OIB localities and comparing them in a unified principal component analysis (PCA) space. PCA space is used here to reduce the dimensionality of the dataset and highlight the main axes of compositional variability, allowing systematic geochemical trends to be more clearly resolved. This approach allows us to evaluate whether the processes identified at La Palma, namely the recycling of evolved tephri-phonolitic to phonolitic mushes by mafic recharge, may be applicable to low-flux OIB systems more broadly.

We show that similar geochemical signatures and trends are preserved across low-flux OIB systems worldwide and that these can be likely linked to the evolutionary stage of the volcanic islands and potentially to the flux regime. We find that OIB systems in their initial stage, characterized by peak flux regime, preserve clinopyroxene compositions in mafic lithologies consistent with recycling of an evolved mush, expressed as deviations

in the PCA space from the main recharge–differentiation trend (Fig. 10). This indicates that mafic recharge not only drives crystallization, but also remobilizes pre-existing evolved mushes stored at depth, mirroring our findings at Cumbre Vieja. Similar observations apply to well-characterized large submarine guyots. Although their precise evolutionary stage is unknown, these features represent flat-topped submarine mountains that were likely once emergent, subsequently eroded, and probably reached an initial evolutionary stage. In contrast, mature OIBs, during which magma flux was likely waning, lack this recycling signature in mafic rocks, suggesting that the response of the plumbing system to mafic recharge changes over time.

These observations imply that evolved melts can be generated and stored at depth during peak flux stages of OIB evolution, without requiring prolonged shallow crustal accumulation. Such melts may eventually contribute to felsic eruptions or be tapped directly from near-Moho reservoirs (Berthod et al., 2021). The cryptic nature of these deep reservoirs has important implications for hazard assessment, as eruptions sourcing them may occur with limited or no detectable precursory signals using current geophysical monitoring approaches.

We refer the reviewers to the revised manuscript and to the supplementary material where we explain data selection, filtering criteria and PCA approach.

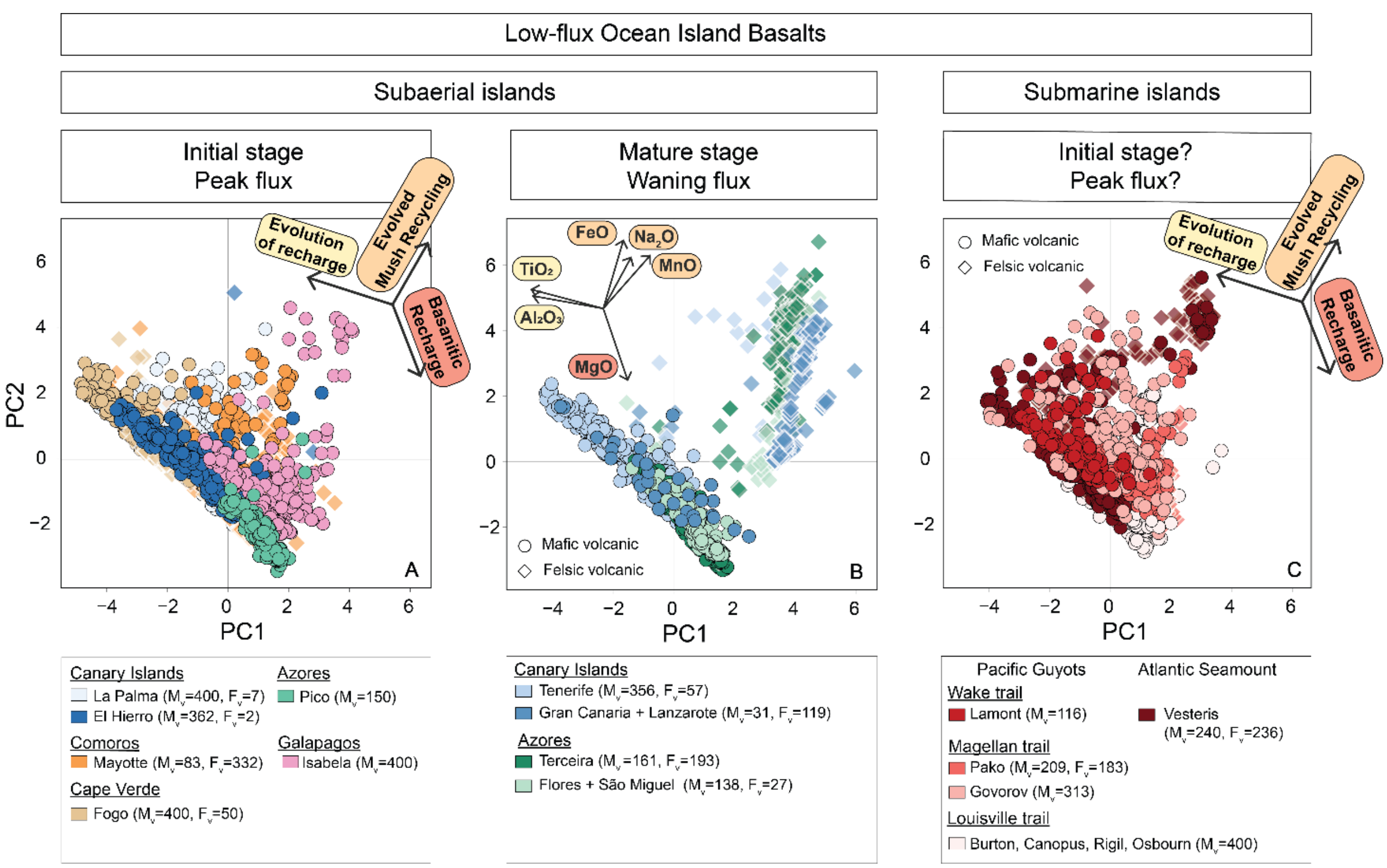

Reviewer #1 (Remarks to the Author):

This manuscript presents a comprehensive clinopyroxene-based investigation of the magma plumbing system beneath La Palma, integrating a large and diverse dataset that includes petrography, major and trace element geochemistry, high resolution trace-element maps, thermobarometry, and cluster analysis across three historical eruptions (1712, 1971 and 2021). The effort to combine mineral chemistry, spatially resolved datasets and quantitative petrological tools is evident and represents a substantial amount of work. The internal consistency of the clinopyroxene datasets across eruptions is one of the clear strengths of the study, as is the attempt to place the 2021 Tajogaite eruption into a longer-term historical framework.

The authors interpret these data as evidence for a temporally stable, upper-mantle plumbing system hosting an evolved, phonolite-related crystal mush that is repeatedly reactivated by injections of mafic basanite magma. This conceptual framework is internally coherent and broadly consistent with existing petrological and geophysical models proposed for La Palma. However, as currently developed, the manuscript largely consolidates and refines a conceptual model that is already established in the literature rather than clearly extending or challenging it.

While the dataset is rich and well-integrated, the main conclusions remain incremental in nature, and the manuscript would benefit from a more explicit articulation of its conceptual advance. This point is particularly important given the scope of Nature Communications, which typically emphasizes studies with a clear step-change in understanding or a strong methodological advance.

Although the authors repeatedly refer to low-flux OIB volcanoes in general, the manuscript does not convincingly demonstrate how insights from La Palma fundamentally alter our broader understanding of such systems. In addition, the manuscript is very long and, in places, conceptually fragmented between the main text and the Supplementary Material, which makes the narrative difficult to follow and obscures the key messages. For these reasons, in the Reviewer opinion, the current framing does not yet fully align with the expectations of a broad-scope journal such as Nature Communications, although the study addresses an important topic and could find a very appropriate audience in a more specialized journal within the fields of volcanology and igneous petrology.

We thank the reviewer for these thoughtful comments. They helped us better refine both the content and the presentation of the manuscript, with the aim of more clearly highlighting the broader implications of our results for the understanding of low-flux OIB volcanoes. We have revised the structure and presentation of the manuscript to reduce

fragmentation between the main text and the Supplementary Material, which is now follow the same structure as the manuscript. Also, we have improved the overall flow for a broader readership and better align the paper with the scope of Nature Communications.

Major conceptual issues:

1) Throughout the manuscript, clinopyroxene zoning patterns, particularly the occurrence of Cr-rich inner rims and the distribution of clusters, are interpreted as direct evidence for repeated mafic recharge events and progressive evacuation of a crystal mush. While this interpretation is plausible, the causal link between zoning style and specific plumbing-system dynamics is not demonstrated rigorously enough to exclude alternative explanations. For example, variations in compatible element concentrations and Mg# could also arise from local boundary-layer effects, changes in crystal growth rates during undercooling, or variations in melt composition unrelated to discrete recharge events. In addition, the possibility that part of the chemical variability observed in clinopyroxene cores reflects crystallographic effects (e.g. sector versus prism-sector zoning) is not systematically addressed. Given the central role assigned to zoning in the overall interpretation, a more critical evaluation of these alternative mechanisms is necessary to avoid over-interpreting the zoning record.

The Cr-rich inner rims identified in our dataset (Cluster 5) are systematically characterized by high Mg# and depletion in incompatible elements, notably Al_2O_3. This is illustrated in Fig. S3, where inner rims display distinctly lower Al_2O_3 contents compared to other textural positions. Importantly, slow-diffusing elements such as Al do not correlate with Cr, indicating that high-Cr growth zones are not generated by local enrichment of slowly diffusing species within a liquid boundary layer surrounding rapidly growing crystals. The observed decoupling between Cr and Al therefore argues against a kinetic or boundary-layer origin for the Cr-rich zones, consistent with findings from Ubide and Kamber (2018).

In contrast, Cr enrichment is accompanied by elevated Mg#, as shown in Figs. S22, supporting an origin linked to mafic magma recharge (e.g., Streck, 2008). While Mg enrichment may be partially attenuated by diffusion and annealing during antecryst storage, Cr zoning is preferentially preserved owing to its slower diffusion. Following Ubide and Kramer (2018), we interpret Cr-rich zoning as the most reliable recorder of mafic replenishment events.

2) A key implicit assumption of the manuscript is that clinopyroxene zoning preserves a coherent temporal record that can be directly linked to eruption staging, particularly the transition from early tephrite to later basanite. However, no diffusion modelling or kinetic

constraints are provided to demonstrate that, for examples, inner rims and outer rims formed within resolvable, eruption-relevant time windows. Without such constraints, it remains unclear whether the zoning patterns record short-term pre-eruptive processes or instead reflect longer-term crystal recycling within a persistent mush system. This ambiguity weakens the argument that the observed zoning can be used to infer eruption priming or the sequence of magmatic processes immediately preceding eruption. Explicitly addressing the timescales represented by the different textural domains would significantly strengthen the manuscript.

We refer the reviewer to our major reply titled “Timescale of the different textural domains”

3) Although the manuscript presents a large and carefully assembled dataset, many of its main conclusions are already documented for La Palma. The present study refines these ideas rather than substantially modifying them. For the journal’s scope, it would be important to clarify what aspects of the proposed model genuinely represent a new conceptual insight. At present, the manuscript would benefit from a clearer statement of whether the novelty lies in the temporal persistence of the plumbing system, the specific role assigned to recharge versus triggering, or the methodological integration itself. Without this clarification, the overall impact of the study remains limited.

We refer the reviewer to our major reply titled “Broader implications beyond La Palma”

4) The authors frequently extrapolate their findings to low-flux OIB volcanoes in general. However, the manuscript does not sufficiently discuss how representative La Palma is in terms of magma supply rate, lithospheric thickness etc. These factors can vary significantly among OIB systems and may strongly influence plumbing-system architecture. As a result, the broader applicability of the proposed model remains uncertain. A more explicit discussion of which aspects of the La Palma system are likely to be general and which are system-specific would improve the manuscript and better justify its relevance for a broad readership.

We thank the reviewer for the comment. We have now introduced magma flux more extensively throughout the manuscript and expanded our findings for La Palma to other low-flux OIB volcanoes. We refer the reviewer to our major reply titled “Broader implications beyond La Palma” and to the revised manuscript.

5) The manuscript argues that mafic recharge plays a key role in priming the system but does not act as the immediate trigger for eruption. While this distinction can be reasonable, it is not consistently maintained throughout the text. In some sections, recharge appears to be discussed in ways that verge on short-term eruption control, whereas in others it is framed strictly as a long-term conditioning process. This lack of conceptual clarity makes it difficult to assess the causal role of recharge in eruption

dynamics. A clearer separation between long-term system evolution and short-term eruption initiation would strengthen the overall interpretation.

We agree with the reviewer on this point and have improved the clarity of the text. We argue that mafic recharge was not the immediate eruption trigger, but rather a key mechanism that contributed to unlocking the mush and building up pressure within the system. Following mafic recharge, there is evidence for fractionation and cooling, either in the upper mantle or in the crust, in the days preceding the eruptions.

The interpretation of mafic recharge as a longer-term process was based on the observation that some clinopyroxene crystals record multiple recharge events in their innermost zoning patterns. We interpreted these features as evidence of repeated recharge episodes within the system, which may have progressively contributed to unlocking the mush over longer timescales. However, given the difficulty of constraining these longer-term timescales, and the fact that only three crystals from Teneguia 1971 and one crystal from Tajogaite 2021 display such features, we decided not to emphasize this aspect and instead focus on the short-term processes immediately preceding the eruptions preserved in inner rims.

6) The interpretation of Cluster 4 as a “cryptic primitive seed for the phonolite lineage” is based on extremely limited data. Given its rarity and the lack of clear independent constraints, this interpretation appears speculative. While such hypotheses can be valuable, they should be more explicitly framed as tentative and not given the same weight as interpretations based on more robust datasets. The discussion of Cluster 4 is notably more speculative than that of other clusters, yet this speculative interpretation is still integrated into the main conceptual cartoon and evolutionary framework. This creates an imbalance in the strength of evidence supporting different parts of the proposed model. Alternatively, a reduction or simplification of the clustering scheme might help avoid over-interpretation (see later).

We agree with the reviewer that the interpretation of Cluster 4 remains somewhat speculative, as it is based on fewer observations than the other clusters. For this reason, the discussion of Cluster 4 has been kept intentionally brief, and we explicitly state in the revised manuscript that the evidence supporting this cluster is more limited than for the other clusters. At the same time, this does not imply that Cluster 4 is chemically insignificant. As reported in the revised manuscript and discussed in our major reply entitled “Clustering methods,” Cluster 4 defines a well-constrained domain in the t-SNE space (Fig. S24D), and reducing the clustering scheme, for example to five or four clusters, would not simply eliminate Cluster 4. As further discussed in our major reply entitled “Clustering methods,” and now shown in the revised manuscript (Figs. S24), the six-cluster solution is fully supported by both the geochemical dataset and the textural

observations. In our view, a reduced number of clusters would therefore not strengthen the interpretation, but rather risk obscuring potentially meaningful structure in the data.

Methodological issues

1) The cluster analysis is central to the manuscript but is described in an overly simplified manner. Key methodological details are missing, including data preprocessing steps if applied (e.g. normalization, scaling), and the criteria used to select the number of clusters. The choice of six clusters is justified primarily by visual inspection, which is insufficient for a study so strongly reliant on clustering. Data-driven approaches such as silhouette analysis, elbow methods or dimensionality-reduction techniques (e.g. PCA) should be used to objectively justify the chosen number of clusters. Applying such methods might even reduce the number of clusters, potentially simplifying the interpretation and eliminating the need for poorly constrained groups such as Cluster 4. In addition, it is not entirely clear whether the clustering is based exclusively on LA-ICP-MS spot analyses or whether chemical map data are also included. From the text and figures, clustering appears to rely on spot analyses only, with chemical maps used primarily for visualization. This distinction is important and should be clarified, as it has implications for spatial coherence and the robustness of the clustering results. If both spot analyses and chemical map data are indeed combined in the clustering workflow, the authors should explicitly demonstrate how datasets characterized by different spatial resolutions, signal integration volumes and data-reduction procedures inherent to laser-based techniques can be made fully comparable within a single clustering framework. Without such justification, the mixing of differently reduced datasets may introduce additional noise into the chemical dataset, potentially biasing cluster structure and subsequent interpretations.

   We thank the reviewer for the comment, and we refer them to our major reply titled “Clustering methods,” above.

2) The care taken to assess equilibrium conditions is really appreciated. However, the use of different thermobarometric models for different crystal domains appears unnecessary and potentially confusing. The Reviewer is aware about the authors’ expertise in global regression-based and machine-learning thermobarometers, and it is unclear why a single, internally consistent approach was not adopted. At the very least, the rationale for excluding such models should be explained more clearly.

   We thank the Reviewer for this comment. This careful approach is motivated by recent experimental work demonstrating that clinopyroxene compositions record different crystallisation conditions depending on crystal domain, growth regime, and degree of

undercooling. In particular, experimental constraints show that the reliability of exchange-based clinopyroxene thermobarometers depends strongly on whether crystals grew under near-isothermal, low-undercooling conditions (e.g., sector-zoned domains) or under high undercooling and decompression-dominated regimes (e.g., outer rims and microlites).

As shown by MacDonald et al. (2023), exchange-based thermobarometers calibrated solely on isothermal–isobaric experiments perform best for low-ΔT, near-equilibrium clinopyroxene domains, whereas models incorporating decompression and undercooling more accurately reproduce crystallisation conditions for rapidly grown rims and microlites. Applying a single thermobarometric model across all crystal domains would therefore introduce systematic biases, either overestimating pressures and temperatures for low-ΔT domains or underestimating them for high-ΔT domains.

For this reason, we adopted a domain-specific approach in which equilibrium is first evaluated and thermobarometric models are selected according to the textural context of each clinopyroxene population. At the same time, in the Supplementary Material, we compare five individual thermobarometric approaches without applying a textural-specific subdivision, demonstrating that the choice of thermobarometer has a negligible impact on the overall conclusions (Fig. S13-S14).

Following the Reviewer's suggestion, we have also tested a new machine-learning (ML) based clinopyroxene barometer (Ágreda-López et al., 2024) and incorporated the results into the manuscript. We find that the ML-based barometer complement classical methods and allow to better resolve differences in storage depths of clinopyroxene rims, placing inner rims, outer rims, and microlites in the crust, at depths comparable to the crustal pre-seismicity preceding the 2021 Tajogaite eruption (del Fresno et al., 2023). These findings suggest that a crustal reservoir, also indicated by fluid inclusions (Zanon et al., 2024) and experimental studies (Andújar et al., 2025), cannot be confidently ruled out from a petrological perspective, despite not being resolved by exchange-based barometers. We now integrate this into the discussion and implement this possibility.

3) While no major flaws are evident in the fractional crystallization modelling, the use of thermodynamically based models (e.g. MELTS, alphaMELTS or MCS for major and trace elements) would strengthen the manuscript. Such models would add rigour to the interpretation of the basanite-tephrite transition and reinforce the conclusion that phonolitic melts form after ~80% crystallization.

We thank the reviewer for this suggestion. We have now used MAGEMin (Riel et al., 2022) to model fractional crystallization. MAGEMin is a Gibbs free-energy minimization solver that computes the thermodynamically stable phase assemblage for a given thermodynamic database, bulk-rock composition, pressure, and temperature conditions. Specifically, we performed the calculations using the web-based Julia interface MAGEMinApp.

Fractional crystallization of major elements was modeled using the Igneous thermodynamic database (Green et al., 2025), starting from a primitive matrix composition and assuming a constant pressure of 5 kbar, consistent with our clinopyroxene–melt barometry results. The crystallization path was simulated between 1250 °C and 900 °C. The model provides the stable mineral phase assemblage and phase proportions at each step of crystallization.

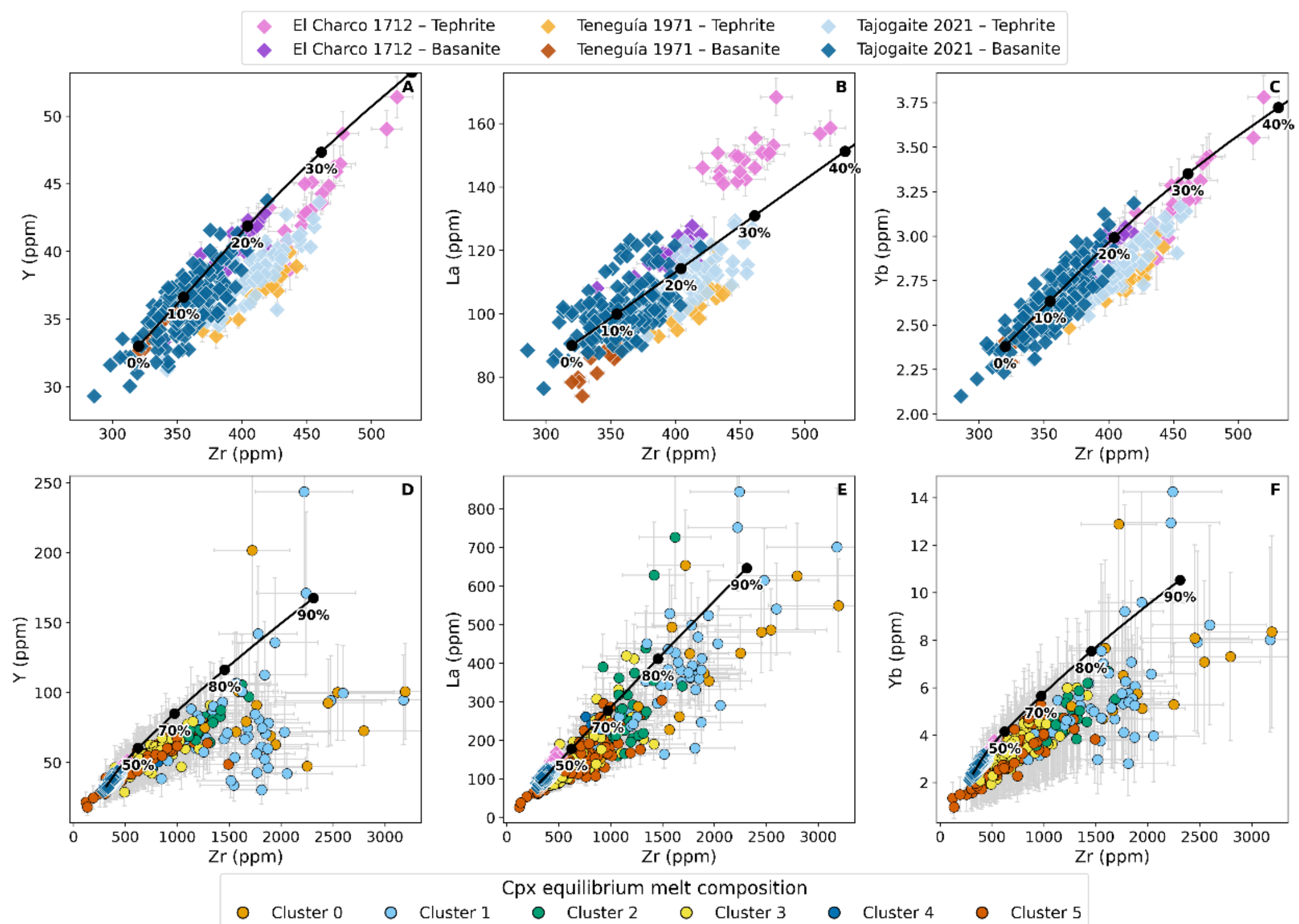

Trace element modeling was subsequently performed using the phase proportions obtained from the MAGEMin output. At each fractionation step, bulk partition coefficients were calculated based on the proportions of crystallizing phases, as detailed in the Supplementary Material. This more robust modelling confirms our previous interpretation that the most evolved Cluster 0 and 1 reconstructed equilibrium melt compositions can be reproduced after 80-90 % fractional crystallization (Fig. S19). Following 80-90 % fractional crystallization, composition would fall in the tephriphonolite to phonolite group (Fig. S21).

Minor comments (number of lines as in the PDF)

Lines ~99–106

Please clarify whether compositional variability in clinopyroxene cores could partly reflect sector zoning effects. When describing clinopyroxene cores, the manuscript attributes most of the observed chemical variability to complex magmatic histories involving antecryst recycling and mush processes. However, the potential contribution of crystallographic effects, such as sector versus prism-sector zoning, is not discussed, despite clear evidence of patchy and sector zoning in the presented BSE images. Because sector zoning can generate significant chemical heterogeneity without changes in melt composition, neglecting this possibility risks over-interpreting core compositions in terms of magmatic processes alone.

We thank the reviewer for this comment. Clinopyroxene crystals from our work do show complex zoning; however, the textures indicate that different zoning styles occur in different parts of the crystals. The cores are predominantly patchy-zoned, which is commonly interpreted as reflecting long-term open-system processes in crystal mush environments (Streck, 2008). In contrast, sector zoning is mainly observed in inner and outer rims, where it is associated with rapid crystallization during magma recharge and ascent.

This distinction between patchy cores and sector-zoned outer crystal domains has been reported in several basaltic systems, including La Palma (Ubide et al., 2023), Etna (MacDonald et al., 2024), Atlantic seamounts (Beloša et al., 2024), and Stromboli (Di Stefano et al., 2020; Petrone et al., 2022). Based on these textures, we interpret the core variability as reflecting mush processes, while sector zoning mainly characterizes the later recharge-related growth zones.

Line ~119 and throughout

Supplementary figures and tables are not cited in the order in which they appear, making the Supplementary Material difficult to navigate. This disrupts the logical flow of the manuscript and makes it unnecessarily challenging for the reader to follow the progression of the results and interpretations.

We thank the reviewer for the suggestion. Supplementary figures are now ordered according to their appearance in the manuscript, from Results to Discussion to Methods.

Lines ~166–178

Matrix compositions are treated as reliable proxies for equilibrium melts. However, the matrix is described as microcrystalline rather than glassy, implying crystallization during late ascent or emplacement. Although the methodology used here takes advantages from another published work, the manuscript does not sufficiently discuss how raster-based

LA-ICP-MS analyses may integrate micro-scale heterogeneity or submicroscopic crystalline phases, potentially biasing melt compositions toward apparent homogeneity. This introduces a conceptual inconsistency when matrix compositions are subsequently used for equilibrium tests and trace-element modelling.

The raster-based LA-ICP-MS approach adopted here follows an established methodology developed by Ubide et al. (2023), where rastered microcrystalline groundmass from lavas from Tajogaite (La Palma) were shown to return comparable results to glassy groundmass from tephra erupted on the same dates (cf. Fig. 2 in Ubide et al., 2023). Importantly, this approach does not employ a large stationary spot; instead, it uses relatively wide and elongated rasters (200–300 µm long, comprising five parallel lines), that provide protracted signals without overwhelming the mass spectrometer detector (as detailed recently also in Bennett et al., 2025 JPet). By integrating signals over large microcrystalline areas, this approach averages out micro-scale heterogeneities, thereby returning a composition representative of a carrier melt.

During data reduction, submicroscopic crystalline phases that could influence the apparent matrix composition can be identified and filtered by looking at the time-resolved signals of all trace elements during LA-ICP-MS analysis. Spikes or anomalous enrichments can be excluded, ensuring that the retained signal reflects a spatially averaged matrix composition. Further details on the technique and data reduction can be found in Ubide et al. (2023) and the method has been applied successfully to lavas with a range of magma compositions globally, to help investigate magma evolution as well as liquid-dependent thermobarometry (e.g., MacDonald et al., 2023 CMP, Jegal et al., 2025 CMP, Bennett et al., 2025 JPet, Cetina et al., 2025 Lithos, Moreno-Alfonso et al., 2026 JPet).

Line 350: Etna should not be classified as a low-flux volcano. This classification is incorrect and undermines the low-flux conceptual framework used elsewhere in the manuscript to contextualize La Palma and other similar systems. The analogy with Etna is used to reinforce interpretations of recharge-driven dynamics. However, given the fundamentally different magma supply rates and plumbing architectures of Etna and La Palma, this comparison weakens rather than strengthens the low-flux OIB narrative developed in the manuscript.

We agree that Etna is not a low-flux volcano and that its tectonic setting is more complex than that of La Palma or other OIB systems. We have revised the text accordingly. However, the chemical composition of Etna magmas is broadly similar to that of La Palma, and many studies at Etna focus on mafic recharge processes, which we use as a comparison in the recharge-related discussion. For the broader implications, we now focus on low-flux OIB systems, without including Etna in that context.

EPMA methods: The inter-instrument comparison of clinopyroxene analyses is appreciated, but the manuscript does not clearly explain how identical analytical spots were located across instruments or how Poisson statistics were applied to quantify inter-instrument reproducibility. Additional clarification would improve transparency and reproducibility.

Near-identical analytical spots were located across instruments based on visual inspection. During each analytical session, the position of each analysis was saved on backscattered electron (BSE) images, allowing the same spot to be revisited and re-analysed in subsequent sessions. Re-analyses were therefore conducted at nominally identical locations based on visual alignment. This has been added in the revised method. We acknowledge that minor spatial offsets are possible, potentially explaining small differences between the original measurements and the re-analyses.

EMPA uncertainties derived from the ZAF correction scheme represent counting-statistics uncertainties, reflecting the precision of the X-ray intensity measurements. These uncertainties were assumed to represent the analytical uncertainty within each session and were subsequently used to evaluate inter-instrument reproducibility during re-analyses as in Fig. S22-S23.

LA-ICP-MS methods:
Lines 529 and 569: The Zenodo data repository referenced in the manuscript is inaccessible to the Reviewer, and its contents are not clearly described.

We apologize for this; we have now removed the embargo and made it available publicly.

The raster method used for groundmass major-element analyses is only briefly mentioned. Although described in Ubide et al. (2023), a short summary would improve the self-contained nature of the manuscript and reduce reliance on external references.

See our response above regarding the raster method. We have now included additional analytical details related to the raster approach. Full methodological details are provided in Ubide et al. (2023).

Line 578: More information on the Python script used for data processing should be provided, or the script should be made publicly available.

We have now included in the Supplementary Material the script used for clustering and for evaluating quantitative methods to determine the optimal number of clusters. We have

also provided a script to visualize chemical maps and reproduce Fig. 5 and also for thermobarometry calculations.

Reviewer #2 (Remarks to the Author):

Review of: *Recurrent evacuation of mantle mush by mafic recharge in ocean island basalts, recorded by La Palma clinopyroxene*

By: Caracciolo et al.

Summary: This manuscript primarily presents detailed clinopyroxene major, minor, and trace element spot and map data and analysis to characterize the mineral histories of clinopyroxene crystals in three historical eruptions of La Palma: El Charco 1712, Teneguía 1971, and Tajogaite 2021. The new data are leveraged with a compilation of other literature data, as well as thermobarometry, to reconstruct the magmatic processes that led to each eruption and assess changes through time. The results convincingly show that the mantle mush region, magmatic recharge, and priming of the mantle plumbing system was remarkably consistent for the three eruptions in this study, implying a characteristic process by which mafic recharge mobilizes cold mush and prepares the system for eruption. However, mafic recharge is not the immediate trigger for these eruptions.

General Comments: The manuscript is well written, data-rich, and contains careful synthesis and interpretation of the geochemical datasets. The textural/petrographic analysis and clinopyroxene mapping, followed by cluster analysis, elevates this work above similar studies in the literature and strongly supports the conclusions derived by the authors. I leave below only very minor comments related to the geochemical work and its presentation, which was excellent.

My big concern is that the authors have not convinced me of the potential significance of the results – *whether these results will be important to the field and advance understanding in a way that will move the field forward (per Nature Communications review guidelines).* They lightly touch on the timeliness of the work in the introduction, and comment that low-flux OIB systems remain relatively understudied compared to high-flux OIB like Hawaii or Iceland. This notion is revisited in the end of the discussion section, in Lines 445-452, but the authors write that their model only *“slightly deviates from observations at other basaltic volcanoes worldwide, where mafic recharge is often identified as the immediate eruption-triggering mechanism.”* They conclude with a high-level basic statement about monitoring perspectives and hazard assessment, but don’t elaborate much on this topic before concluding.

Because of this weakness, my primary concern is that this contribution lacks a broad appeal to the volcanological community outside of those who study mineral histories or

La Palma. If the authors could make these implications clearer, with more robust discussion, I feel this contribution would be more suitable for publication in Nature Communications. At present, it seems to fall short of “moving the field forward” outside of their specialty.

We thank the reviewer for these comments and fully appreciate the concern regarding the broader appeal of the study and its potential to “move the field forward” beyond the immediate specialty of researchers working on La Palma. In response, we have worked to improve the manuscript and to more clearly explain how our results inform the general understanding of magma storage, mush remobilization, and pre-eruptive processes in low-flux OIB systems. We have also strengthened the discussion to better highlight the broader volcanological implications of these findings and to better align the manuscript with the requirements of Nature Communications.

Line-by-Line Comments:

Line 53 – “Magmatic processes occurring within a magma plumbing cannot be”→word or phrase missing in this sentence

We do not see a missing word or phrase in this sentence. The line reads ‘Magmatic processes occurring within a magma plumbing system cannot be directly observed, yet they….’

Line 63 – “There is a detailed record of historical eruptions” →this would benefit from further clarification; record via eyewitness accounts vs. monitoring data vs. what?

We have rephrased the sentence. The intended meaning was that there is a well-documented historical record of eruptive activity on the island, allowing inter-eruption intervals to be constrained based on the cited references. We have now specifically referred to a paleomagnetic study that refines the historical eruptive activity at La Palma.

Line 114 – “consistent with green cores” → do you mean the green observed in plane polarized light? If yes, can you clarify this, because you also have green symbols on your Fig3 leading to some initial confusion on my part.

Corrected

Lines 246-247 – “corroborated by melt and fluid inclusion studies during the 2021 Tajogaite eruption, which point out magma storage depth between 15-30 km” →here could you please clarify which phases the MI and FI work was conducted on? Implication is it was done on cpx, but I think they were olivine yes?

We now specified that this were olivine-hosted MI and FI.

Line 721 - extra “o” in thermobarometry in beginning of figure caption

Corrected

Lines 420-421 – should probably add “low-flux” before OIB at the end of this sentence

Corrected

Lines 450-452 – this would benefit from a comment about the timescales over which this process occurs, hinted at previously in the sentence as several years with the priming for 2021 beginning in 2017. When talking about hazard assessment and early warning, it’s important to recognize the time-delay between signals and any imminent activity. Which in the case of low-flux OIB could be a non-predictable number of years. On human time frames, for decision makers, policy makers, etc, several years of uncertainty is a big deal. I feel like this could be honed a bit more prominently in your message in this paper, to make the link between your detailed geochemical work and the implications for hazards assessments.

We have completely revised that section. Please see our major reply n.2 and the revised text.

Lines 466-467 – issue with tense in this sentence, you moved from past tense to active

Corrected

Figures:

Fig 10 – the reader would benefit from having the cluster numbers also associated with the color legend here, to make linking the schematic diagram back to the data (e.g. Fig 8 and 9) more straightforward. Especially since the cluster numbers aren’t aligned from deepest-to-shallowest process or anything intuitive like that.

Added

Reviewer #3 (Remarks to the Author):

This manuscript by Caracciolo et al. uses clinopyroxene zoning to reconstruct how the La Palma (Cumbre Vieja) plumbing system behaved across three eruptions (El Charco 1712, Teneguía 1971, Tajogaite 2021). There dataset comes primarily from the quantification of cpx zonations by LA-ICP-MS spot analyses and trace-element maps, combined with major-element EMPA. They integrate both single-spot trace elements and pixel-based trace-element map data into a large multivariate dataset and apply a clustering analysis (k-means) to identify recurring compositional clusters that are then mapped onto textural positions (cores/inner rims/outer rims/microlites). The results allowed them to interpret the clusters/cpx zones in terms of recycled evolved antecrysts (phonolitic mush), mafic recharge, post-recharge differentiation, and ascent/cooling signatures. They further estimate magma storage depths and temperatures using cpx-melt thermobarometry following the equilibrium/calibration framework of MacDonald et al. (2023), and the paper argues for temporally consistent upper-mantle storage with a cryptic shallow level that does not leave a cpx record.

Overall, the dataset is substantial, well-structured and well-written. The manuscript is clearly motivated by the need to understand plumbing system variability in low-flux basaltic systems, where monitoring/forecasting is a challenge. I found the manuscript interesting, and the use of k-means clustering to classify cpx rim domains from high-resolution trace-element data provides a novel and useful framework for objectively linking zoning patterns to magmatic processes. The data is certainly worthy of publication, although I suggest the significance/broadness of the results is emphasized more to meet the scope of the journal. I also appreciate the wealth of supplementary figures to support the results. Below I have listed my main concerns I would like to see addressed, mainly to ensure the robustness of the conclusions made.

We thank the reviewer for this positive and thoughtful assessment of our work. In response to the reviewer's concern regarding scope, we have revised the manuscript to more clearly emphasize the broader implications of our results, especially their relevance

for understanding magma storage, recharge, remobilization, and eruption triggering in low-flux basaltic OIB systems more generally, beyond the specific case of La Palma.

Main comments

1) Clustering analysis. You identify 6 clusters from your analysis. I gather from your supplementary information that these 6 clusters were defined primarily from visual inspection of a map by varying k (2-10). Although I think this is a reasonable approach, I don't find it sufficient for the conclusions that rely on both the distinctiveness and temporal persistence of the cluster groups. Can you provide at least one quantitative basis for k choice, demonstrating that 6 was the best fit (e.g. bootstrapping or others)? It will demonstrate the robustness of your choice.

From what I understood, the clustering analysis of the trace element data was performed on combined spot (n=318) and pixel-based map data (n=3398776). This is a big class imbalance, meaning that the k-means clustering will be dominated by the pixel population. This is important because most petrological inferences comes from textural defined spot analyses and eruption to eruption comparisons. Could you test whether the cluster solutions are still valid when the clustering analysis runs with spots only, and pixels only. You could also downsample pixels per map so pixels and spots are roughly comparable weights.
Right now, it seems like the subjective k choice and pixel dominance needs clearer justification.

In regards the input data for the clustering, is the clusters analysis performed using the raw ppm concetration matrices or from post-processed data (scaled/transformed)? I see the methods section the cpx matrices underwent processing and enhancements. So I would like to see in the clustering methods section what filtering was applied prior to clustering, as this could have a significant effect on the boundary pixels of the different cpx zones.

We thank the reviewer for their comments about the clustering method. We refer the reviewer to our major reply titled "Clustering methods" and to the revised clustering method.

2) What could be other explanations that the shallow reservoir is cryptic? Maybe cpx isn't the phase to best record this stage? What if the timescales or temperatures may prevent re-equilibration at in the cpx, or shallow storage was dominated by melt + other phases. I find in several places in the manuscript the wording is overly strong, for example line

252 “we do not observe evidence for shallow storage…” could be rephrased to “shallow storage was not recorded in the cpx”.

Our interpretation was that crustal storage is not recorded when using exchange-based clinopyroxene barometers in our study, nor in other barometry studies of La Palma (e.g. Scarrow et al., 2024; Ubide et al., 2023), or in barometry studies based on amphibole (González-García et al., 2023).

However, application of a machine-learning based barometer returns complementary results, possibly better resolving differences in storage depths among textural domains, placing inner rims, outer rims, and microlites in the crust, at depths comparable to the crustal pre-seismicity preceding the 2021 Tajogaite eruption (del Fresno et al., 2023). These findings suggest that a crustal reservoir, also proposed by fluid inclusions (Zanon et al., 2024) and experimental studies (Andújar et al., 2025), cannot be confidently ruled out from a petrological perspective, despite not being resolved by cpx – liquid exchange-based barometry. We now integrate this into the discussion.

The question on whether the recharge was a trigger vs. priming is certainly an important aspect of this study and what makes it novel and is a strong statement. Could the lack of cluster 5 in the earlier cpx crystals in the inner rim be a consequence of sampling, eruption frequency , or overprinting?

We do not consider sampling bias to be a plausible explanation for the absence of Cluster 5 in early-erupted clinopyroxene inner rims. Although only two crystals were mapped for the early-erupted products compared to three for the late-erupted products, we also analysed single-spots, that show that clinopyroxenes hosted in early-erupted tephrites are systematically lower in Cr compared to those hosted in later-erupted basanites, consistent with the clustering results (Fig. 4G-I).

With respect to eruption frequency, we acknowledge that we lack constraints on clinopyroxenes derived from eruptions outside those investigated here, and therefore cannot directly assess the role of longer-term eruptive cyclicity. However, we note that the pre-eruptive repose times of the three eruptions studied vary substantially (approximately 22–50 years), yet the observed geochemical patterns are remarkably consistent across eruptions. This argues against eruption frequency alone as the primary control on the presence or absence of Cluster 5 in inner rims.

Finally, regarding overprinting, we are not aware of a mechanism capable of modifying the trace-element composition of clinopyroxene inner rims after their crystallization, particularly in a way that would affect only early-erupted crystals while leaving later-erupted clinopyroxenes unaffected. Given the slow diffusion of Cr in clinopyroxene, post-crystallization overprinting is unlikely to be a viable mechanism here.

3) The fractional crystallization model is nice and the workflow in the supplementary info is clear. However, despite the mention of the uncertainties with using fixed partition coefficients, there is there a risk that it obscures the P-T-composition dependence? Your uncertainty treatment does address this partly. In regards your fractional crystallization assemblage, you use the assemblage from Ubide et al. (2023), which excludes amphibole? But in line 305 you explicitly mention the MREE/HREE is partly through amphibole + cpx partitioning. This inconsistency should be addressed, either include amphibole into the FC model or explicitly state that it is not capturing the role of amphibole.

We have revised the FC model by using MAGEMin (Riel et al., 2022), that computes thermodynamically stable phase assemblages for a given thermodinamic database, melt composition, pressure and temperature conditions.

Fractional crystallization of major elements was modeled using the Igneous thermodynamic database (Green et al., 2025), starting from a primitive matrix composition and assuming a constant pressure of 5 kbar, consistent with our clinopyroxene–melt barometry results. The crystallization path was simulated between 1250 °C and 900 °C. The model provides the stable mineral phase assemblage and phase proportions at each step of crystallization.

Trace element modeling was subsequently performed using the phase proportions obtained from the MAGEMin output. At each fractionation step, bulk partition coefficients were calculated based on the proportions of crystallizing phases, as detailed in the Supplementary Material.

4) Significance of the study needs more emphasis. I think within the volcanology community this study is neat and potential very useful. However, I find the manuscript reads almost like a single volcano study, thus I have some concerns over the broader impact of the study, particularly for its publication in Nat. Comms. For a broader impact, I would recommend clearer statements upfront in the intro that gives a generalizable insight beyond “La Palma has persistent upper mantle storage and a recurrent mush system”, which is system specific unless the authors demonstrate transferability to other systems. For example, better connections to hazard/monitoring by stating testable links between cluster-defined processes and geophysical/geochemical monitoring: this is stated in the intro and the very end of the discussion, but I would like to see a better link and more specific connections with your data. Also, do you think magma recharge not being the immediate trigger for an eruption is common at many other volcanoes? As this is a big conclusion of the study, I would like to see clarification which of your finds are likely common of low-flux OIB plumbing systems, and which are La Palma specific.

We thank the reviewer for the comment about the limited broader implications and refer them to our major reply titled “Broader implications beyond La Palma”. The finding that magma recharge was not the immediate eruption trigger, and that cooling followed recharge, possibly in the shallow crust, is indeed an interesting outcome of this study. However, we find it challenging to extend this conclusion to other OIB volcanoes, as doing so would require comparing our data with similarly resolved temporal datasets, which are currently not available. Hence, we decided to focus our new implication section on the link between recycling of phonolitic mushes and magma flux in OIBs worldwide.

Specific comments

Line 26: “Interrogating” may be overly strong. Suggest something like “decoding”.

Corrected

Line 30: Be more specific on the type of cluster analysis, e.g., clustering analysis of cpx zoning.

Corrected

Line 32: phonolitic *crystal* mush. Mush on its own could imply a physical state of something.

Corrected

Line 37: This is rather interpretive and evidence needs to be watertight to make such a claim.
A more conservative option could be “does not appear to represent the immediate trigger”.

Corrected

This feeds in to my main comments 1 & 2.

Line 38-40: This appears to be the main conclusion and novel part of the study. Most, if not all, studies relate magma recharge as the trigger of such eruptions. This is an interesting claim. It begs the question about sample/CPX retrospectivity.

We agree with the reviewer. It is a novel aspect that we were able to disentangle thanks to trace element chemical maps combined with temporally resolved sampling.

Line 52: Can you expand or add an extra few sentences on the why low-flux means different to high-flux systems? E.g., does longer repose = more crystal mush development?

We have now revised the initial part of the introduction and better introduced the concept of low-flux systems.

Line 53: magma plumbing *system*

Corrected

Line 67: Sentence reads funny. “Magma plumbing” twice. Please rephrase.

Corrected

Line 68-69: Sentence is fine, but I would consider tightening the citations up rather than clumping them all together. Consider splitting the citation up in the sentence.

We have now revised the sentence.

Line 87: Only basanites have olivine? Please clarify.

Corrected

Line 90-91: Abit of an awkward sentence. Also, this may be confusing to call all phenocrysts “crystals”. Why not call them just phenocrysts?

We revised the sentence. We avoid the term phenocrysts because it implies growth from the carrier melt, which is not applicable to the clinopyroxene cores. Instead, we use the term crystals, and reserve microlites for grains smaller than 100 µm.

Line 93: inclusions

Corrected

Line 104: returns *to lighter* BSE.

Corrected

Line 108: All in mol%?

Yes, specified

Line 146: trace elements*

Corrected

Line 190: Missing closing parentheses

Corrected

Line 220: thermobarometric*

Corrected

Line 255-257: Could this shallow zone be recorded by other phases, like olivine diffusion, plagioclase, melt inclusion work?

There is evidence of this shallow zone from melt, fluid inclusion and experimental studies. Additionally, we have evidence of this shallow zone using the ML-based barometer. This has been integrated into the main text.

Line 304: Check figure reference to Fig. 22? Should this be Fig. S22? Or maybe Fig. S21?

This was a mistake. Supplementary figures have now been revised and renumbered.

Line 347: associated *with*

Corrected

Line 359: *likely* zoned

Corrected

Line 426: …eruption *trigger*.

Corrected

Line 447-452: The monitoring perspective is nice, but this could be expanded to highlight the broader significance of this petrological study.

We have revised completely this part of the discussion. See revised text.

Line 471: inner rims *show*

Corrected

Line 506-507: state explicitly the beam size. A 5 um defocused vs a focused (1 um) beam.

Corrected

Line 538: consisting *of* laser…

Corrected

Line 545: *assessed*

Corrected

Line 576-577 and Figure 8 caption: Inconsistency in the data trimming? 1st-99th vs 0.1-99.9? Check.

Corrected

Line 600-602: The size of the dataset is impressive. Were any of these standardized (z-score) before k-means? Without standardizing elements like Ti and Zr would dominate purely by scale.

Yes, the data were standardized prior to clustering using the standard scaler (scikit-learn, version 1.7.2), but this was not described sufficiently clearly in the original text. We refer the reviewer to our major reply no.1 and to the revised Methods section for clarification.

Line 649: * Python*

Corrected

Figure 7: Caption - *Thermobarometry* results

Corrected

Figure 8: The colors should be explained in the figure caption. I know in this figure that the colors are not important for reading the figure, which is mainly for the use in figure 9. But I would state something in the caption that these colors are used to distinguish each cluster for subsequent figures. In the captions, you state that 6 was chosen as it "best captured" the main textural and chemical features. I would like to see at least one quantitative metric that also supports 6 clusters.

Corrected, figure has been updated. See our major reply n.1 about the clustering approach.

References

Abràmoff, M.D., Magalhães, P.J., Ram, S.J., 2004. Image processing with imageJ. Biophotonics International 11, 36–41. https://doi.org/10.1117/1.3589100

Ágreda-López, M., Musu, A., Jorgenson, C., Šala, M., Giordano, G., Caricchi, L., Stremtan, C., Petrelli, M., 2025. The crystal cargo provides a chronicle of pre-caldera dynamics in mafic volcanic systems: insights from Colli Albani. Bull. Volcanol. 87. https://doi.org/10.1007/s00445-025-01865-6

Ágreda-López, M., Parodi, V., Musu, A., Jorgenson, C., Carfì, A., Mastrogiovanni, F., Caricchi, L., Perugini, D., Petrelli, M., 2024. Enhancing machine learning thermobarometry for clinopyroxene-bearing magmas. Comput. Geosci. 193. https://doi.org/10.1016/j.cageo.2024.105707

Andújar, J., Scaillet, B., Frascerra, D., Di Carlo, I., Casillas, R., Suárez, E.D., Domínguez-Cerdeña, I., Meletlidis, S., López, C., Slodczyk, A., Martí, J., Núñez-Guerrero, E., 2025. Evolution of the crustal reservoir feeding La Palma 2021 eruption. Insights from phase equilibrium experiments and petrologically derived

time scales. Journal of Volcanology and Geothermal Research 463. https://doi.org/10.1016/j.jvolgeores.2025.108327

Armienti, P., Perinelli, C., Putirka, K.D., 2013. A new model to estimate deep-level magma ascent rates, with applications to Mt. Etna (Sicily, Italy). Journal of Petrology 54, 795–813. https://doi.org/10.1093/petrology/egs085

Beloša, L., Murphy, D.T., Ubide, T., Callegaro, S., Meyzen, C.M., Bizimis, M., Mazzini, A., 2024. Magmatic evolution and architecture of an oceanic intraplate volcano: Vesteris Seamount, Atlantic Ocean. Front. Earth Sci. (Lausanne). 12. https://doi.org/10.3389/feart.2024.1501694

Berthod, C., Médard, E., Di Muro, A., Hassen Ali, T., Gurioli, L., Chauvel, C., Komorowski, J.C., Bachèlery, P., Peltier, A., Benbakkar, M., Devidal, J.L., Besson, P., Le Friant, A., Deplus, C., Nowak, S., Thinon, I., Burckel, P., Hidalgo, S., Feuillet, N., Jorry, S., Fouquet, Y., 2021. Mantle xenolith-bearing phonolites and basanites feed the active volcanic ridge of Mayotte (Comoros archipelago, SW Indian Ocean). Contributions to Mineralogy and Petrology 176. https://doi.org/10.1007/s00410-021-01833-1

del Fresno, C., Cesca, S., Klügel, A., Domínguez Cerdeña, I., Díaz-Suárez, E.A., Dahm, T., García-Cañada, L., Meletlidis, S., Milkereit, C., Valenzuela-Malebrán, C., López-Díaz, R., López, C., 2023. Magmatic plumbing and dynamic evolution of the 2021 La Palma eruption. Nat. Commun. 14. https://doi.org/10.1038/s41467-023-35953-y

Di Stefano, F., Mollo, S., Ubide, T., Petrone, C.M., Caulfield, J., Scarlato, P., Nazzari, M., Andronico, D., Del Bello, E., 2020. Mush cannibalism and disruption recorded by clinopyroxene phenocrysts at Stromboli volcano: New insights from recent 2003–2017 activity. Lithos 360–361. https://doi.org/10.1016/j.lithos.2020.105440

González-García, D., Boulesteix, T., Klügel, A., Holtz, F., 2023. Bubble-enhanced basanite–tephrite mixing in the early stages of the Cumbre Vieja 2021 eruption, La Palma, Canary Islands. Sci. Rep. 13. https://doi.org/10.1038/s41598-023-41595-3

MacDonald, A., Ubide, T., Mollo, S., Taddeucci, J., 2024. Spatial and temporal mush heterogeneity during eruptions recorded in clinopyroxene from the 2021 paroxysms at Mt. Etna, Italy. Contributions to Mineralogy and Petrology 179. https://doi.org/10.1007/s00410-024-02174-5

Musu, A., Corsaro, R.A., Higgins, O., Jorgenson, C., Petrelli, M., Caricchi, L., 2023. The magmatic evolution of South-East Crater (Mt. Etna) during the February–April 2021 sequence of lava fountains from a mineral chemistry perspective. Bull. Volcanol. 85. https://doi.org/10.1007/s00445-023-01643-2

Orlando, A.D., Armienti, P., Borrini, D., 2008. Experimental determination of plagioclase and clinopyroxene crystal growth rates in an anhydrous trachybasalt from Mt Etna (Italy). European Journal of Mineralogy 20, 653–664. https://doi.org/10.1127/0935-1221/2008/0020-1841

Petrone, C.M., Mollo, S., Gertisser, R., Buret, Y., Scarlato, P., Del Bello, E., Andronico, D., Ellis, B., Pontesilli, A., De Astis, G., Giacomoni, P.P., Coltorti, M., Reagan, M., 2022. Magma recharge and mush rejuvenation drive paroxysmal activity at Stromboli volcano. Nat. Commun. 13. https://doi.org/10.1038/s41467-022-35405-z

Riel, N., Kaus, B.J.P., Green, E.C.R., Berlie, N., 2022. MAGEMin, an Efficient Gibbs Energy Minimizer: Application to Igneous Systems. Geochemistry, Geophysics, Geosystems 23. https://doi.org/10.1029/2022GC010427

Rykov, A., De Amorim, R.C., Makarenkov, V., Mirkin, B., 2024. Inertia-Based Indices to Determine the Number of Clusters in K-Means: An Experimental Evaluation. IEEE Access 12, 11761–11773. https://doi.org/10.1109/ACCESS.2024.3350791

Scarrow, J.H., Pankhurst, M.J., Barbee, O.A., Chamberlain, K.J., Morgan, D.J., Longpré, M.A., Tramontano, S., Hickey, J., Neave, D.A., Rollinson, G.K., Stewart, A.G., Wieser, P.E., Coldwell, B.C., Hernández, W., D'Auria, L., Pérez, N.M., 2024. Decoding links between magmatic processes and eruption dynamics: whole-rock time series petrology of the 2021 Tajogaite eruption, La Palma. Volcanica 7, 953–980. https://doi.org/10.30909/vol.07.02.953980

Streck, M.J., 2008. Mineral textures and zoning as evidence for open system processes. Rev. Mineral. Geochem. 69, 595–622. https://doi.org/10.2138/rmg.2008.69.15

Templ, M., Filzmoser, P., Reimann, C., 2008. Cluster analysis applied to regional geochemical data: Problems and possibilities. Applied Geochemistry 23, 2198–2213. https://doi.org/10.1016/j.apgeochem.2008.03.004

Ubide, T., Kamber, B.S., 2018. Volcanic crystals as time capsules of eruption history. Nat. Commun. 9, 326. https://doi.org/10.1038/s41467-017-02274-w

Ubide, T., Márquez, Á., Ancochea, E., Huertas, M.J., Herrera, R., Jesús Coello-Bravo, J., Sanz-Mangas, D., Mulder, J., Macdonald, A., Galindo, I., 2023. Discrete magma injections drive the 2021 La Palma eruption. Sci. Adv.

Zanon, V., Schiavi, F., Cyrzan, K., Pankhurst, M.J., 2024. Toward a near real-time magma ascent monitoring by combined fluid inclusion barometry and ongoing seismicity. Sci. Adv. 10, 4300.

Rebuttal letter

Point-by-point response to the reviewers' comments

Second round of review

Manuscript NCOMMS-25-099451

Reviewer #1 (Remarks to the Author):

Second-round review report of Caracciolo et al.

This revised manuscript presents a substantially improved version of the original submission. The authors have clearly engaged with the main concerns raised during the first round of review and have made significant efforts to strengthen the methodological transparency, broaden the conceptual framing, and clarify the role of mafic recharge in the La Palma plumbing system.

In particular, the revised manuscript now better articulates its broader relevance to low-flux OIB systems, provides a more detailed description of the clustering workflow, introduces additional quantitative support for the choice of six clusters, adds thermodynamic fractional crystallization modelling, and includes first-order crystallization timescale estimates for clinopyroxene rims.

Overall, the revision represents a meaningful improvement. Several of the major concerns raised previously have been addressed satisfactorily, especially those related to clustering methodology, preprocessing, cluster-number selection, and the broader significance beyond La Palma.

The original version of the manuscript presented a rich clinopyroxene dataset from three historical eruptions of Cumbre Vieja. The study risked appearing as a refinement of an already established model: upper-mantle magma storage, tephritic-to-basanitic evolution, evolved mush recycling, and recharge-driven remobilization had already been documented or proposed in previous studies of La Palma. The revised manuscript is more ambitious. The authors now frame the study around recurrent evacuation of evolved mantle mushes in ocean island systems more broadly. They also attempt to link the La Palma observations to a global OIB context using a compiled clinopyroxene database

and PCA. This is a useful and really appreciated addition and helps justify the relevance of the work beyond the local case study.

The most important improvement concerns the clustering methodology. The first version did not sufficiently describe preprocessing, scaling, number-of-cluster selection, or whether single-spot analyses and pixel-level map data were combined. The revised version now states that the clustering dataset combines single-spot analyses and quantified pixels from compositional maps, that trace elements were log-transformed, standardized, and filtered for outliers, and that k=6 was selected using a combination of PCA, t-SNE, elbow method, and textural/geological inspection. This is a substantial improvement in transparency.

In my view, the manuscript is now closer to being suitable for publication, but a few points still require clarification before final acceptance. The revised manuscript is significantly stronger than the original submission. The authors have responded constructively to the review and have made substantial improvements. With modest additional clarification, the study would be suitable for publication.

We thank the reviewer for their positive comments and constructive feedback. We are pleased to hear that the revisions have addressed their concerns and that they are satisfied with the changes made to the manuscript.

Major comments:

- Conceptual advance and suitability for a broad-scope journal:

The revised manuscript now more clearly argues that the La Palma dataset reveals a recurring process potentially relevant to low-flux OIB systems worldwide: the storage of evolved tephri-phonolitic to phonolitic mushes in the upper mantle and their repeated recycling or evacuation by mafic recharge. The new global OIB comparison helps strengthen this argument and moves the manuscript beyond a purely local reconstruction. However, the broader generalization still depends strongly on whether the compiled clinopyroxene PCA genuinely captures comparable processes across different OIB systems. The manuscript should be careful not to imply that similar PCA trends necessarily require identical plumbing-system dynamics. Similar major-element trends in clinopyroxene can reflect related but not identical processes, including differentiation, melt composition, sector zoning, and crystallization kinetics. The global comparison is

valuable, but it should be presented as supportive and hypothesis-generating rather than definitive evidence that the La Palma mechanism operates universally. The authors should maintain cautious wording when extrapolating to OIB systems globally.

We thank the reviewer for this constructive comment. We agree that similar clinopyroxene compositional trends in PCA space may arise from a range of magmatic processes, and that the global comparison should be treated as hypothesis-generating rather than as confirmation that the La Palma mechanism operates universally. We have revised the text accordingly. Specifically, we have clarified that similar compositional distributions may reflect variations in melt compositions, crystallization conditions, or crystal recycling, and that the PCA is used to explore whether the process documented at Cumbre Vieja may operate more broadly across OIB systems. We have also softened the language throughout the global comparison.

- Clustering methodology and number of clusters:

This issue has been substantially improved. The revised manuscript now provide a much clearer description of the clustering workflow. The use of log transformation, standardization, outlier filtering, PCA, t-SNE, and elbow analysis is appropriate. The authors also correctly note that there is no uniquely correct number of clusters in unsupervised learning and that cluster-number selection should combine statistical diagnostics with geological and textural interpretability.
The six-cluster solution is now defensible. It is not necessarily uniquely correct, but it is methodologically justified. The combination of elbow analysis and textural coherence supports k=6 as a reasonable partition. The authors' argument that fewer clusters might obscure meaningful zoning domains is plausible.

We are pleased that the reviewer finds the six-cluster solution to be a defensible and appropriate choice for the dataset.

However, I would avoid stating that k=6 is objectively "the" correct solution. It is better described as the most interpretable and geologically useful solution among the tested alternatives. This distinction matters because the clustering is not merely descriptive; it underpins several genetic interpretations. In particular, the preservation of Cluster 4 depends on the chosen partitioning. The authors should therefore avoid overstating the objectivity of k=6.

We thank the reviewer for this comment. We note that we never claim k=6 to be the objectively correct solution. Nevertheless, we acknowledge that the wording in the manuscript may have implied greater objectivity than intended, and we have expanded the methods section to clarify that k=6 is selected based on statistical and geological

observations rather than as a uniquely determined solution. In addition, we have clarified the link between Cluster 4 trace elements and the more abundant major element data (Discussion), which supports the petrogenetic significance of Cluster 4 as the start of the phonolite lineage (Fig. S20). We have clarified this in the Discussion.

Furthermore, there are still aspects that should be improved. The Results section should explicitly report the main clustering results rather than leaving them implicit. Moreover, because clustering represents one of the central methodological advances of this study, its methodology should receive substantially greater emphasis in the main manuscript instead of being largely confined to the Supplementary Material. At present, the Methods section gives considerably more weight to thermobarometry than to clustering. In my opinion, the clustering workflow, including PCA and t-SNE analyses and their implementation, should be described in sufficient detail within the main manuscript.

We thank the reviewer for this comment. We agree that, given the importance of clustering to the interpretation presented in this study, the methodology should be described in sufficient detail within the main manuscript. Accordingly, we have expanded the clustering section in the Methods to provide additional information on the PCA, t-SNE, and clustering workflow, as well as the rationale used to select the final clustering solution.

Regarding the presentation of the clustering results, our intention was not to treat the clustering output as a standalone result, but rather as an interpretative framework that is integrated with petrographic observations, mineral chemistry, and thermobarometric constraints throughout the discussion of the manuscript. For this reason, the main clustering results are presented immediately before the interpretation of individual clusters, rather than in a separate Results section. We believe that describing the geochemical features of each cluster alongside their textural occurrence and petrological significance facilitates reading. Nevertheless, to improve clarity, we have expanded the text in the main manuscript so that the principal clustering results are explicitly summarized before the detailed discussion of the individual clusters (lines 296-311).

- Thermobarometry:

The revised manuscript improves the thermobarometric framework by incorporating both classical clinopyroxene–liquid thermobarometry and the recently developed ML approach of Ágreda-López et al. (2024). This addition addresses the previous concern regarding the use of complementary thermobarometric methods and provides an interesting

comparison between conventional and ML-based estimates of magma storage conditions.

However, one aspect remains unclear to me. The ML thermobarometers are ultimately trained on large compilations of experimental clinopyroxene datasets (following Jorgenson et al., 2022, on which Ágreda-López et al. 2024 is based), which substantially overlap with the experimental datasets that underpin many of the used clinopyroxene–liquid calibrations (e.g., Putirka, 2003; Putirka, 2008 etc..). Consequently, although the regression methodologies differ (ML vs. empirical exchange equations), the underlying calibration space is not entirely independent.

For this reason, I am not fully convinced that the agreement between the ML and exchange-based thermobarometers should be interpreted as an independent validation of the inferred P–T conditions. Rather, it may represent convergence between different predictive approaches trained on overlapping experimental constraints. What additional information is gained by presenting both approaches if they are ultimately constrained by substantially overlapping experimental calibration datasets? I encourage the authors to discuss this point explicitly and clarify the rationale for presenting both approaches together. A brief discussion of their degree of independence would strengthen the methodological framework of the study and help readers better appreciate the added value of the ML-based thermobarometric results.

We thank the reviewer for this comment. However, we disagree with the comment that we interpret the agreement between the ML and exchange-based thermobarometers as an independent validation of the inferred P–T conditions. Our intention was not to present the two approaches as independent, but rather as complementary. Indeed, we explicitly state that the ML thermobarometer provides “a complementary perspective” (line 265) to the exchange-based thermobarometer on the crystallization conditions recorded by the different textural domains. One key advantage of the ML-based approach is that it does not require a liquid composition, allowing pressure estimates for individual textural domains where equilibrium melt compositions are unavailable (e.g. cpx cores). This avoids the bias that would otherwise be introduced by assuming a liquid composition for domains where it is not directly constrained, which is a limitation inherent to exchange-based thermobarometers. Additionally, exchange-based thermobarometers carry an internal interdependency because the barometer requires temperature as an input and the thermometer requires pressure as an input; P and T must be solved iteratively from one another, so uncertainty in one estimate propagates into the other. The ML cpx-only approach avoids this circularity, since P and T are estimated independently. We have added these points to the manuscript to strengthen the methodological framework as suggested by the reviewer (lines 794-799).

- Supplementary codes and reproducibility:

The authors tried to improve reproducibility by providing the Jupyter code used for clustering, and thermobarometric calculations. However, the way the code is supplied is not optimal. The notebooks appear to be provided as raw text files rather than directly usable .ipynb notebooks or a structured code repository. The files also contain executed outputs, hard-coded paths etc. This makes them bulky and difficult to inspect. The documentation is minimal. For example, the clustering notebook does not provide a complete step-by-step workflow. The notebooks are still hard-coded and do not function as a general reproducible pipeline. A better practice would be to provide: (1) clean .ipynb notebooks with cleared outputs, (2) equivalent .py scripts for batch execution, (3) a README.md or a .txt explaining the workflow. Ideally, these should be deposited as a version-controlled GitHub or Zenodo repository rather than embedded as large text supplements.

We thank the reviewer for this comment. We would like to clarify that the original files uploaded during submission were provided as Jupyter Notebook (.ipynb) files. We suspect that their conversion into plain text format occurred during the manuscript submission process or through the submission platform itself. In addition, the original .ipynb notebooks were publicly available through a Zenodo repository (10.5281/zenodo.17635256). This Zenodo repository has now been superseded; all supplementary code and data have been migrated to a new, actively maintained repository linked directly to our GitHub project, ensuring the deposited materials remain current and correctly versioned. The updated DOI is provided below and has been revised throughout the manuscript and the supplementary material.

We nevertheless agree that the documentation and readability of the code can be improved. In response, we have revised the notebooks to improve clarity and organization, removed unnecessary outputs, and added a README file describing the workflow, required inputs, and the purpose of each notebook. In addition, the revised notebooks and associated supplementary files have been deposited in a GitHub repository (https://github.com/AlbCaracc/Caracciolo_et_al_2026_CumbreVieja) and archived in Zenodo (10.5281/zenodo.21081067). We hope these additions will facilitate reproducibility and make the workflow easier for readers to follow.

Reviewer #3 (Remarks to the Author):

I have read the revised manuscript and the authors rebuttal. Overall the paper is clearly improved, and I think most of my main concerns from the first round are adresssed now.

In particular, the clustering workflow is explained much clearer, the choice of k is now backed up with quantitative tests, and they also provide the code/scripts, which makes the analysis alot more transparent and reproducable. I also appreciate the effort to strengthen the broader relevance beyond La Palma, which helps the manuscript fit better within the scope of Nat Comms. The discussion around the “cryptic” shallow storage also reads more balanced now.

I do have one remaining (minor) concern. Even though it is reassuring that the spot data and the pixel data overlap in PCA space, the k-means clustering is still based on a dataset that is dominated by map pixels compared to the much smaller number of spot analyses. A short stability test (for example running k-means using only the spots, or rerunning it on a downsampled pixel dataset with similar weight to the spots) would directly show that the cluster solution and the interpretations tied to the clusters are not sensitive to this pixel:spot imbalance.

Thank you for this suggestion. We performed an additional clustering stability analysis using a balanced dataset containing single-spot analyses and 1000 randomly downsampled map pixels, independently re-clustered, and compared against the six-cluster solution from the full dataset via nearest-centroid matching. The results show good agreement across elements, indicating that the identified clusters and their petrological interpretations are robust and not artifacts of the pixel imbalance. These results have been added to the Supplementary Material (Fig. S25) and are now briefly discussed in the Methods section.

Also, related to clustering, we would like to point out that we identified a minor inconsistency between the single-spot dataset used for clustering and the one used for the geochemical plots. Three analyses (two cores and one microlite from the Teneguía 1971 and El Charco 1712) were excluded during data reduction because their REE patterns suggested they were unlikely to represent clinopyroxene analyses, but were inadvertently retained in the clustering input dataset. To ensure consistency between datasets, these three analyses have now been removed from Fig. S24 relative to the previous version of the figure.

Overall, I think the revision is much stronger, and the remaining points should be straightforward to address.

Paul A. Wallace, LMU Munich

Review of: *Recurrent evacuation of mantle mush by mafic recharge in ocean island basalts, recorded by La Palma clinopyroxene*

By: *Caracciolo et al.*

Summary: This manuscript primarily presents detailed clinopyroxene major, minor, and trace element spot and map data and analysis to characterize the mineral histories of clinopyroxene crystals in three historical eruptions of La Palma: El Charco 1712, Teneguía 1971, and Tajogaite 2021. The new data are leveraged with a compilation of other literature data, as well as thermobarometry, to reconstruct the magmatic processes that led to each eruption and assess changes through time. The results convincingly show that the mantle mush region, magmatic recharge, and priming of the mantle plumbing system was remarkably consistent for the three eruptions in this study, implying a characteristic process by which mafic recharge mobilizes cold mush and prepares the system for eruption. However, mafic recharge is not the immediate trigger for these eruptions.

General Comments: The manuscript is well written, data-rich, and contains careful synthesis and interpretation of the geochemical datasets. The textural/petrographic analysis and clinopyroxene mapping, followed by cluster analysis, elevates this work above similar studies in the literature and strongly supports the conclusions derived by the authors. I leave below only very minor comments related to the geochemical work and its presentation, which was excellent.

My big concern is that the authors have not convinced me of the potential significance of the results – *whether these results will be important to the field and advance understanding in a way that will move the field forward (per Nature Communications review guidelines).* They lightly touch on the timeliness of the work in the introduction, and comment that low-flux OIB systems remain relatively understudied compared to high-flux OIB like Hawaii or Iceland. This notion is revisited in the end of the discussion section, in Lines 445-452, but the authors write that their model only *"slightly deviates from observations at other basaltic volcanoes worldwide, where mafic recharge is often identified as the immediate eruption-triggering mechanism."* They conclude with a high-level basic statement about monitoring perspectives and hazard assessment, but don't elaborate much on this topic before concluding.

Because of this weakness, my primary concern is that this contribution lacks a broad appeal to the volcanological community outside of those who study mineral histories or La Palma. If the authors could make these implications clearer, with more robust discussion, I feel this contribution would be more suitable for publication in Nature Communications. At present, it seems to fall short of "moving the field forward" outside of their specialty.

Line-by-Line Comments:

Line 53 – “Magmatic processes occurring within a magma plumbing cannot be” → word or phrase missing in this sentence

Line 63 – “There is a detailed record of historical eruptions” → this would benefit from further clarification; record via eyewitness accounts vs. monitoring data vs. what?

Line 114 – “consistent with green cores” → do you mean the green observed in plane polarized light? If yes, can you clarify this, because you also have green symbols on your Fig3 leading to some initial confusion on my part.

Lines 246-247 – “corroborated by melt and fluid inclusion studies during the 2021 Tajogaite eruption, which point out magma storage depth between 15-30 km” → here could you please clarify which phases the MI and FI work was conducted on? Implication is it was done on cpx, but I think they were olivine yes?

Line 721 - extra “o” in thermobarometry in beginning of figure caption

Lines 420-421 – should probably add “low-flux” before OIB at the end of this sentence

Lines 450-452 – this would benefit from a comment about the timescales over which this process occurs, hinted at previously in the sentence as several years with the priming for 2021 beginning in 2017. When talking about hazard assessment and early warning, it’s important to recognize the time-delay between signals and any imminent activity. Which in the case of low-flux OIB could be a non-predictable number of years. On human time frames, for decision makers, policy makers, etc, several years of uncertainty is a big deal. I feel like this could be honed a bit more prominently in your message in this paper, to make the link between your detailed geochemical work and the implications for hazards assessments.

Lines 466-467 – issue with tense in this sentence, you moved from past tense to active

Figures:

Fig 10 – the reader would benefit from having the cluster numbers also associated with the color legend here, to make linking the schematic diagram back to the data (e.g. Fig 8 and 9) more straightforward. Especially since the cluster numbers aren’t aligned from deepest-to-shallowest process or anything intuitive like that.
